# IGF2BP1 overexpression stabilizes PEG10 mRNA in an m6A-dependent manner and promotes endometrial cancer progression

**DOI:** 10.7150/thno.49345

**Published:** 2021-01-01

**Authors:** Lin Zhang, Yicong Wan, Zihan Zhang, Yi Jiang, Zhiyue Gu, Xiaoling Ma, Sipei Nie, Jing Yang, Jinghe Lang, Wenjun Cheng, Lan Zhu

**Affiliations:** 1 Department of Obstetrics and Gynecology, Peking Union Medical College Hospital, Peking Union Medical College, Chinese Academy of Medical Sciences, No. 1 Shuaifuyuan, Dongcheng District, Beijing 100010, China.; 2 Department of Gynecology, The First Affiliated Hospital of Nanjing Medical University, No. 300 Guangzhou Road, Nanjing, Jiangsu 210029, China.

**Keywords:** N6-methyladenosine, IGF2BP1, endometrial cancer, PEG10, cell cycle

## Abstract

**Rationale:** N6-methyladenosine (m^6^A) mRNA methylation is the most abundant chemical posttranscriptional modification in mRNA and is involved in the regulation of a number of biological processes. Insulin-like growth factor 2 mRNA-binding protein 1 (IGF2BP1) has recently been reported as having the capacity to recognize m^6^A sites in mRNA and plays a role in regulating mRNA metabolization. However, it is unclear which genes IGF2BP1 targets to identify m^6^A sites and what are their respective functions in endometrial cancer (EC).

**Methods:** Quantitative PCR, western blot and immunohistochemistry were used to measure IGF2BP1 expression in EC cell lines and tissues. Xenograft experiments were performed to examine the *in vivo* role of IGF2BP1 in EC cell growth. RNA-binding protein immunoprecipitation sequencing, methylated RNA-binding protein immunoprecipitation sequencing and RNA-sequencing were also conducted to identify potential IGF2BP1 targets involved in EC regulation. Co-immunoprecipitation and mass spectrometry were used to identify IGF2BP1-interacting proteins.

**Results:** IGF2BP1 expression increased in EC, and high expression of this protein correlated with poor prognosis. IGF2BP1 overexpression/knockdown can promote (and inhibit) cell proliferation and regulate the tumor cell cycle and cancer progression, both *in vivo* and *in vitro*. Mechanistically, IGF2BP1 can recognize m^6^A sites in the 3' untranslated region (3'UTR) of Paternally Expressed Gene 10 (PEG10) mRNA and recruits polyadenylate-binding protein 1 (PABPC1) to enhance PEG10 mRNA stability, which consequently promotes PEG10 protein expression. Additionally, it would appear that a large number of PEG10 proteins bind p16 and p18 gene promoter sequences, thereby repressing expression and accelerating the cell cycle.

**Conclusion:** This investigation found that IGF2BP1 has a crucial role in the m^6^A-dependent regulatory mechanism for endometrial cancer. This study provides new insights into our understanding of disease progression and provides another potential route for understanding biological functions.

## Introduction

Endometrial cancer (EC) is the most common gynecological cancer in developed countries and is a global threat to women's health and well-being 1. Estrogen is still considered a key factor in tumorigenesis and EC cell proliferation because continuous estrogen stimulation without progesterone antagonism, drives abnormal proliferation of endometrial epithelial cells 2. However, in some patients with an estrogen-independent subtype, the occurrence of tumors cannot be explained by abnormal levels of either estrogen or progesterone. Additionally, these subtypes are often more malignant and resistant to various endocrine therapies 3-5. As populations continue to grow, so too does the incidence of EC and therefore, it remains of the utmost importance to further study the mechanisms involved in EC progression, if we are to develop effective interventions.

N6-methyladenosine (m^6^A) in mRNA is the most abundant posttranscriptional modification in mRNA and is now thought to be involved in the regulation of gene expression in a number of processes, such as tumor proliferation, invasion and epithelial-mesenchymal transition 6-7. The effector proteins involved in m^6^A pathways include enzymes *i.e.* “writers” and “erasers” which effectively install and remove mRNA methylation. After m^6^A modification, mature mRNAs are exported from nuclei to cytoplasm where they are recognized by “readers” 8-9. Different readers are capable of recognizing different m^6^A sites and are consequently involved in RNA stabilization, translation, and degradation 10.

Previous studies have demonstrated that IGF2BP1 is an RNA binding protein (RBP) which regulates cellular proliferation, apoptosis and invasion 11. IGF2BP1 was discovered as a reader protein which binds to a specific m^6^A sites in mRNA 12. Although recently, Müller *et al.* found that IGF2BP1 stabilizes the SRF gene in an m^6^A-dependent manner and participates in the regulation of ovarian cancer cell stemness maintenance 13. IGF2BP1 has also been found to enhance c-Myc mRNA stability and promote tumor progression by recognizing m^6^A in c-Myc mRNA 14. This suggests that IGF2BP1 regulates multiple, targeted genes and may influence tumor progression by regulating multiple mRNAs through m^6^A modification.

To date, no team has specifically explored the potential function of IGF2BP1 in EC. Therefore, we designed this study to investigate the expression and function of IGF2BP1 in EC and to identify associations with prognoses. We also examine the functions and underlying mechanisms of IGF2BP1 in EC through interacting factors and by identifying the novel targets of IGF2BP1.

## Materials and methods

### Patients' sample

RNA samples from 20 normal endometria and 96 EC samples from EC patients were retained from our previous study 15. Of the 96 EC samples, there were grade 1 (n = 38), grade 2 (n = 27), grade 3 (n = 23), and UPSC (n = 8). Complete clinical data were available for all patients. This study was approved by the Institutional Review Board at the First Affiliated Hospital of Nanjing Medical University.

### Quantitative real-time PCR analysis

RNA samples were reverse transcribed to cDNA by reverse transcriptase (Takara). Gene expression was detected using a quantitative PCR kit (Takara) and with a 7900HT real-time instrument (ABI). Experimental procedures followed manufacturers' instructions. Primers are listed in Table S1. Transcript levels were analyzed according to the 2^-ΔΔCT^ method and β-actin expression was used as the internal control.

### Western blotting

Cells were washed twice in pre-chilled PBS and lysed in a buffer (100 mmol/L NaCl, 50 mmol/L Tris [pH 8.0], 1% NP-40, 0.1% SDS, 0.5 mM EDTA and 1× proteinase inhibitor cocktail). Lysates were centrifuged at 12500 g. Supernatants were collected and proteins were denatured using an LDS buffer (Thermo Fisher). Protein samples were separated by SDS-PAGE and transferred to a PVDF membrane. Membranes were blocked in 5% skimmed milk and sequentially incubated with primary and secondary antibodies. Antibody information is provided as supplementary materials, Table S2. The immunoblot was developed using Chemi-Doc (Bio-Rad) and ECL (Bio-Rad).

### Tissue microarray (TMA) and immunohistochemistry (IHC)

A total of 204 tissue array samples were immunochemically processed and consisted of comparatively normal endometria (n = 30), grade 1 (n = 75), grade 2 (n = 59), grade 3 (n = 31), and UPSC (n = 9). The IHC protocol is similar to that described in our previous related study 15.

After paraffin embedding, sectioning, dewaxing, antigen preparation and serum blocking, samples were stained with antibodies. Antibody details are also provided as supplementary materials, Table S2. IHC staining was semi-quantitatively assessed based upon stain intensity and the percentage of positive cells. Stain intensity was categorized as 0 (*i.e.*, negative), 1 (weak positive), 2 (moderately positive), or 3 (strongly positive). The percentage of positive cells was categorized, as follows; 1 (*i.e.*, 0%-10%), 2 (11%-50%), 3 (51%-80%), or 4 (81%-100%). Final scores for each section were then calculated by multiplying stain intensity scores by stain percentages. Immunohistochemical scores of ≥8 points were considered high expression, and ≤6 points were considered low expression. IHC staining was scored independently by two pathologists, then averages were calculated.

### Cells and cell culture

Human endometrial cancer cell lines: Ishikawa, HEC-1-A, HEC-1-B, RL-95-2, AN3CA and KLE were purchased directly from China Center for Type Culture Collection (CATCC). Cells were cultured as described in our previous study 15.

### Lentivirus construction and infection and siRNA

The lentivirus-mediated gene overexpression and the knockdown vector system were purchased from Han Biotechnology (Shanghai, China) with lentiviral vectors carrying FLAG and puromycin tags. Cells were seeded in 6-well culture plates before the lentivirus was applied. Empty lentiviruses served as the negative control and non-infected cells served as the control group. After 48 h, cells were cultured in 5 μg/mL of puromycin to screen for cell lines which stabilize differential gene expression *i.e.*, overexpression and down-expression. Gene expressions were verified through RT-PCR and western blotting. siRNAs were synthesized by Tsingke Biological Technology (Wuhan, China). Cells were transfected with siRNA using RNAimax (Invitrogen) according to the manufacturer's protocol. Oligo sequences are also provided in Table S1 of the supplementary materials.

### Cell proliferation assays

Cell proliferation activity was detected with Cell Counting Kit 8 (CCK8) and EdU assays. For CCK8 assays, cells were seeded (4×10^3^ cells/well) into 96-well plates. CCK8 solution, supplied by Beyotime (Shanghai, China), was added at the indicated time points and then absorbance was measured at 450 nanometers using a microplate reader. For EdU assays, cells (1×10^6^) were plated in 6-well plates. When cells reached 50% confluence, cells were treated with 10 mM EdU (Invitrogen) and phase S cells were detected with the supplied analysis kit (Invitrogen), according to the manufacturer's protocol.

### Cell cycle analysis

Cells were harvested by trypsinization, centrifuged and fixed in 75% ice cold ethanol at 4 °C overnight. Next, 50 μg/mL of propidium iodide (PI) and 1 mg/mL of RNase supplied by Beyotime (Shanghai, China) were added to each sample. After 30 minutes, cells were analyzed using a FACS flow cytometer (Becton-Dickson Bioscience), and the results were processed with the associated Modfit software.

### RNA-binding protein immunoprecipitation sequencing and quantitative PCR (RIP-Seq and RIP-qPCR)

EZ-Magna RIP™ RNA-Binding Protein Immunoprecipitation Kit (Millipore) was used according to the manufacturer's protocol, in order to detect the RNAs that bind IGF2BP1 proteins. AN3CA cells (1×10^7^) were washed in PBS twice and then lysed in 500 μL IP lysis buffer (containing 1× Protease Inhibitor Cocktail and 5 μL RNAse inhibitors). The supernatant was collected centrifugally and 10% was reserved as the input. To the 90% supernatant remaining, flag antibody (5 μg) and Magnetic Beads (40 μL) complex were added and samples were incubated at 4 °C overnight. Beads were washed in a buffer and eluted with an RNA purification kit (Qiagen), according to the manufacturer's protocol. The Ultra II RNA kit (NEB) was used to prepare the library according to the manufacturer's instructions. The samples were sequenced using the HiSeq PE150 platform. qPCR was performed with a 7900HT real-time instrument. All primers are listed in Table S1.

### Methylated RNA immunoprecipitation sequencing and quantitative PCR (MeRIP-seq and MeRIP-PCR)

Cells were washed in cold PBS twice, and the Oligotex Direct mRNA Midi/Maxi Kit (Thermo Fisher) was used to purify mRNA according to the manufacturer's instructions. The concentration of mRNA was quantified using a spectrophotometer, and 5 μg of mRNA was used for immunoprecipitation. The Magna MeRIP™ m^6^A Kit (Millipore) was then used for MeRIP according to the manufacturer's instructions. Samples were sequenced with the HiSeq PE150 platform and qPCR was performed with a 7900HT real-time instrument. Again, all primers are provided in the supplementary materials as Table S1.

### RNA-sequencing and quantitative PCR (RNA-seq and q-PCR)

Cells were washed twice with pre-chilled PBS. RNA was extracted using the RNeasy Mini kit (Qiagen), according to the manufacturer's instructions. The Ultra II RNA kit (NEB) was used to prepare the library, again according to the manufacturer's protocol. The library was sequenced with the HiSeq PE150 platform. q-PCR was performed with a 7900HT real-time instrument. Primers are listed in Table S1.

### RNA-pull down assays

The 3'-biotin RNA probe was synthesized by Tsingke. RNA probes contain an m^6^A base, an adenine base or a mutation to a guanine base, separately. We used RNAimax reagents (Invitrogen) to transfect RNA probes into cells. After 48 h, cells were washed twice in cold PBS and lysed in 500 μL IP lysis buffer (containing 1× Protease Inhibitor Cocktail and 5 μL RNAse inhibitors). One-tenth of the supernatant was saved as the input. To the remaining supernatant, 40 μL streptavidin beads (Invitrogen) were added and samples were incubated at room temperature for 2 h. The beads were eluted by 95% formamide at 95 °C for 2 mins after five washes with IP wash buffer (Thermo Fisher). Protein samples were denatured by LDS buffer (Thermo Fisher) and analyzed with western blotting.

### Co-immunoprecipitation and mass spectrometry (CoIP-MS)

The Pierce™ Classic Magnetic IP/Co-IP Kit (Thermo Fisher) was used (according to the manufacturer's instructions) to examine proteins coupled to IGF2BP1. Cells (1×10^7^) were washed in PBS twice and then 500 μL IP lysis buffer (containing 1× Protease Inhibitor Cocktail) was added to lyse cells. One tenth of the supernatant was saved as the input. To the remaining supernatant, 5 μg flag antibody-magnetic bead complex was added and then samples were incubated at 4 °C, overnight. Beads were washed five times with buffer containing 10 μg/mL RNAse A before being eluted by 100 μL elution buffer. Protein samples (20 μL) were boiled in LDS buffer for western blotting, and 80 μL protein samples were analyzed by label-free LC/MS. All related antibody messages are provided in Table S2.

### Cleavage under targets and tagmentation sequencing (CUT&Tag-seq)

The signal-to-noise ratio of CUT&Tag-seq is generally much higher than the ChIP-seq. Therefore, in order to detect PEG10 protein-coupled DNA fragments, the CUT&Tag Kit (Vazyme) was used in accordance with the manufacturer's instructions. 1×10^4^ cells were harvested with ConA beads and then cells were sequentially incubated with PEG10 antibody, a secondary antibody and pA-Tn5. PCR was used for library preparation after fragmenting and extracting DNA. The library was sequenced with HiSeq PE150 platform. Antibody messages are listed in the supplementary materials as Table S2.

### Chromatin immunoprecipitation and quantitative PCR (ChIP-PCR)

ChIP utilizes cell signaling technology *i.e.*, the Enzymatic Chromatin IP Kit, during experiments. All procedures were as per usual, carried out in accordance with predefined instructions. Cells were fixed with formaldehyde and lysed, before micrococcal nuclease enzyme was added to cut the chromatin into small fragments. PEG10 antibody and the protein G magnetic bead complex were added for chromatin immunoprecipitation. After protein-DNA complexes were de-crosslinked, a DNA purification spin column was used to purify and recover DNA, before performing qPCR analysis. Primers are listed in Table S1.

### Immunofluorescence (IF)

Cells were fixed in 4% paraformaldehyde for 20 minutes and washed three times with PBS. 0.5% NP-40 buffer was then added and the antigen was blocked with goat serum. Cells were sequentially incubated with primary and secondary antibodies. After three washes with PBS, 5 μg/mL DAPI and neutral balsam were added. Fluorescence imaging was performed using a confocal fluorescence microscope (Zeiss). The antibody messages have been provided in Table S2.

### Luciferase reporter assays

Luciferase assay was performed using a dual luciferase reporter assay system (Promega) according to the manufacturer's instructions. We cloned those containing either the wild-type or mutant sequences into the pmirGLO reporter vector (Promega). Detailed DNA sequences can be found in Table S1. Plasmids were verified by DNA sequencing. Cells seeded in 6-well plates were transfected with luciferase vectors using Lipofectamine 3000 (Invitrogen). After 48 h, firefly and Renilla luciferase activities in each well were assessed.

### RNA lifetime assays

Cells were treated according to our experimental design. Actinomycin D (Sigma) was added at a concentration of 5 mg/mL. Then, at the indicated times, cells were lysed and the total RNA was extracted (Qiagen). RNA quantities were determined through q-PCR analysis. RNA half-life was calculated according to a previous study 12.

### Tumorigenesis assay *in vivo*

Experimental animal procedures were approved by the Institution of Animal Care and a committee at Peking Union Medical College. All animals received care in compliance with the 'Guide for the Care and Use of Laboratory Animals'. Six-week-old, female SCID-Berge mice were purchased from Vitalriver. EC cells with IGF2BP1 overexpression or silencing or the appropriate controls (1×10^6^) were injected into lower abdominal cavity of each mouse (n = 5 mice/group). After four weeks, the mice were killed and tumors harvested, weighed and photographed. Tumors were fixed in 4% formaldehyde, paraffin-embedded and analyzed by IHC.

### Sequencing data analysis

RNA-seq/RIP-seq data sheets were analyzed with HiSAT2, feature counts and DESeq2. HiSAT2, MACS2 and HOMER were used to analyze CUT&Tag data. MeRIP-seq data were analyzed with HISAT2 and exomePeak software. Motif discoveries were analyzed by DREME. IGF2BP1 expression and overall EC survival were analyzed using TCGA data which is accessible via GEPIA (http://gepia.cancer-pku.cn) and ONCOLNC (http://www.oncolnc.org).

### Statistical analysis

SPSS (IBM, version 21.0) was used for all statistical analyses. Correlations between gene expression and clinicopathological data were analyzed using χ^2^ and Fisher's exact tests, respectively. Survival curves were generated using the Kaplan-Meier method. Data are expressed as means with corresponding standard deviations (SD). Differences between two groups were analyzed using a two-tailed Student's *t*-test. A *p* value < 0.05 was set as the threshold for statistical significance.

## Results

### IGF2BP1 is associated with clinical EC outcomes

**Figure 1:**
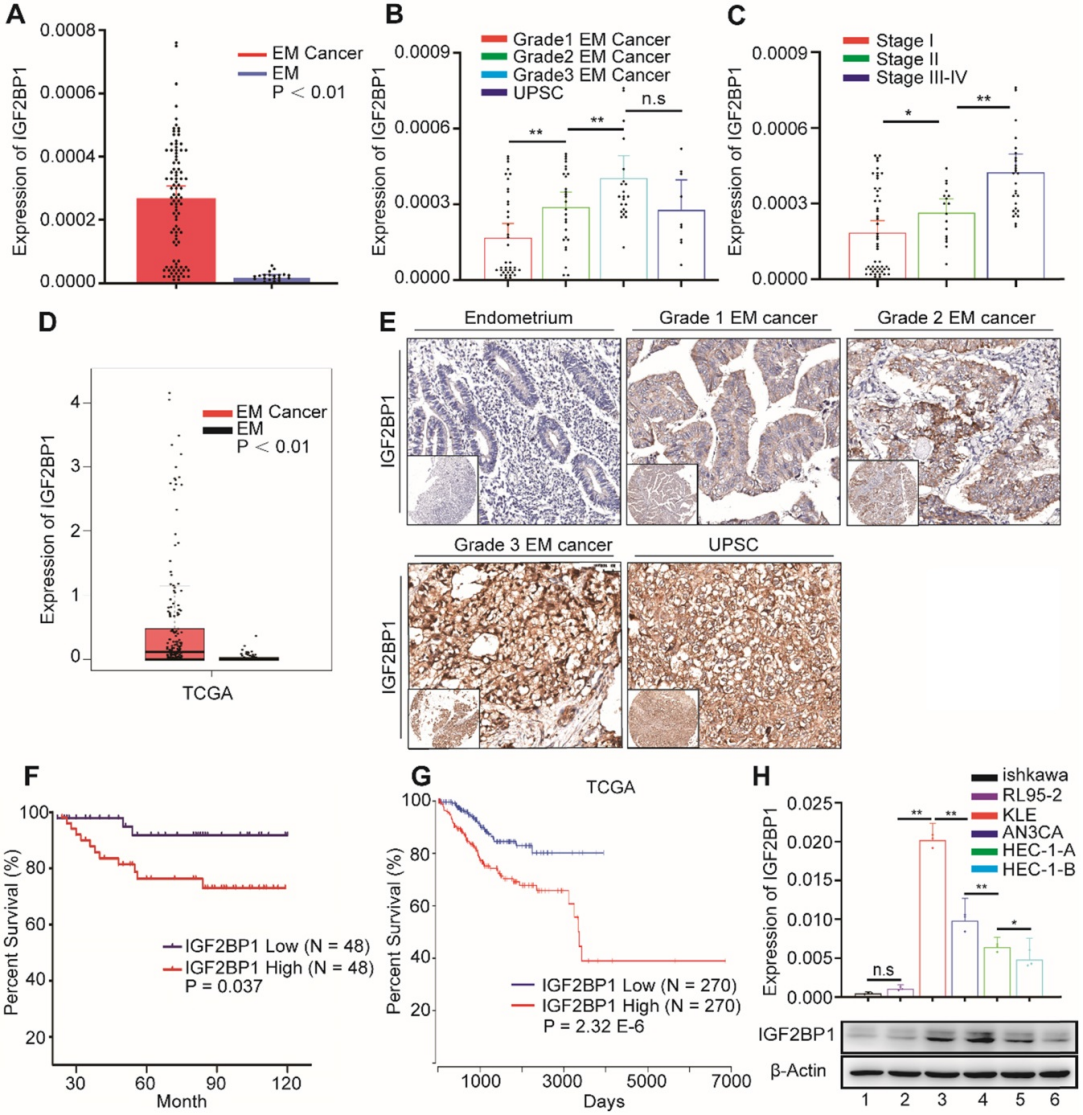
** IGF2BP1 is overexpressed in endometrial cancer and predicts overall survival.** (**A**) IGF2BP1 mRNA expression in normal endometrium (EM) (n = 20) and endometrial cancer tissues (n = 96); (**B**) Q-PCR analysis of IGF2BP1 mRNA across cancer cell grades; (**C**) Q-PCR analysis of IGF2BP1 mRNA at different stages of endometrial cancer; (**D**) Expression of IGF2BP1 in endometrial cancer from TCGA data; (**E**) Expression of IGF2BP1 protein in endometrial tissue and across different grades of endometrial cancer; (**F**) Kaplan-Meier analysis of overall survival in endometrial cancer according to IGF2BP1 expression. Define high and low expressions IGF2BP1 according to the median; (**G**) Overall survival in endometrial cancer cases from TCGA data according to IGF2BP1 expression; (**H**) IGF2BP1 mRNA and protein expressions in EC cell lines. (Top) IGF2BP1 mRNA expression in cell lines by PCR; (bottom) Western blot analysis of IGFBP1 protein expression in cells. (1-6): Ishikawa, RL95-2, KLE, AN3CA, HEC-1-A, and HEC-1-B. Error bars indicate means ± SD, *P < 0.05, **P < 0.01, n.s indicates not significant.

In order to explore IGF2BP1 expression in EC, we first used RT-PCR to detect the expression of IGF2BP1 mRNA in endometrial and EC tissues. IGF2BP1 mRNA expression in EC tissues increased compared with levels found in endometrial tissues (Figure 1A). Expression level positively correlated with both tumor cell grade (Figure 1B) and FIGO stage (Figure 1C). TCGA data analysis also consistently showed that IGF2BP1 expression increased in EC when compared with otherwise normal endometrial tissues (Figure 1D). Next, we used tissue array and IHC to analyze the level of protein in IGF2BP1 in tumor tissues compared with endometrial samples (Figure 1E). Similar to mRNA results, IGF2BP1 protein did not appear to express in normal endometria; however, expression significantly increased in EC. The intensity of IGF2BP1 protein expression also positively correlated with tumor cell grade (Table S3) and FIGO stage (Table S3). There was no significant correlation with depth of tumor invasion, lymphovascular invasion or lymph node metastasis (Table S3).

Survival analysis appears to suggest that patients with high IGF2BP1 expression encounter significantly poorer survival when compared with patients with low expression (Figure 1F). Analysis of TCGA data also suggests that high IGF2BP1 expression may be a negative prognostic indicator for EC patients (Figure 1G). However, IGF2BP1 appears to be expressed differently in EC cell lines. Expression was lower in the Ishikawa cell line which was derived from highly differentiated tumors. Conversely, expression was higher in more malignant cell lines *i.e.,* AN3CA and KLE cells (Figure 1H).

### IGF2BP1 promotes EC cell proliferation and accelerates cell cycle progression

To investigate IGF2BP1 functions in EC, we used lentivirus to upregulate IGFBPI expression in EC cell lines and constructed lentivirus expressing shRNA to silence IGF2BP1 expression in tumor cells (Figure S1A-B). CCK-8 assays showed that the proliferation rate of AN3CA and KLE cells increased after IGF2BP1 upregulation. Cell proliferation reduced after IGF2BP1 silencing (Figure S1C).

**Figure 2:**
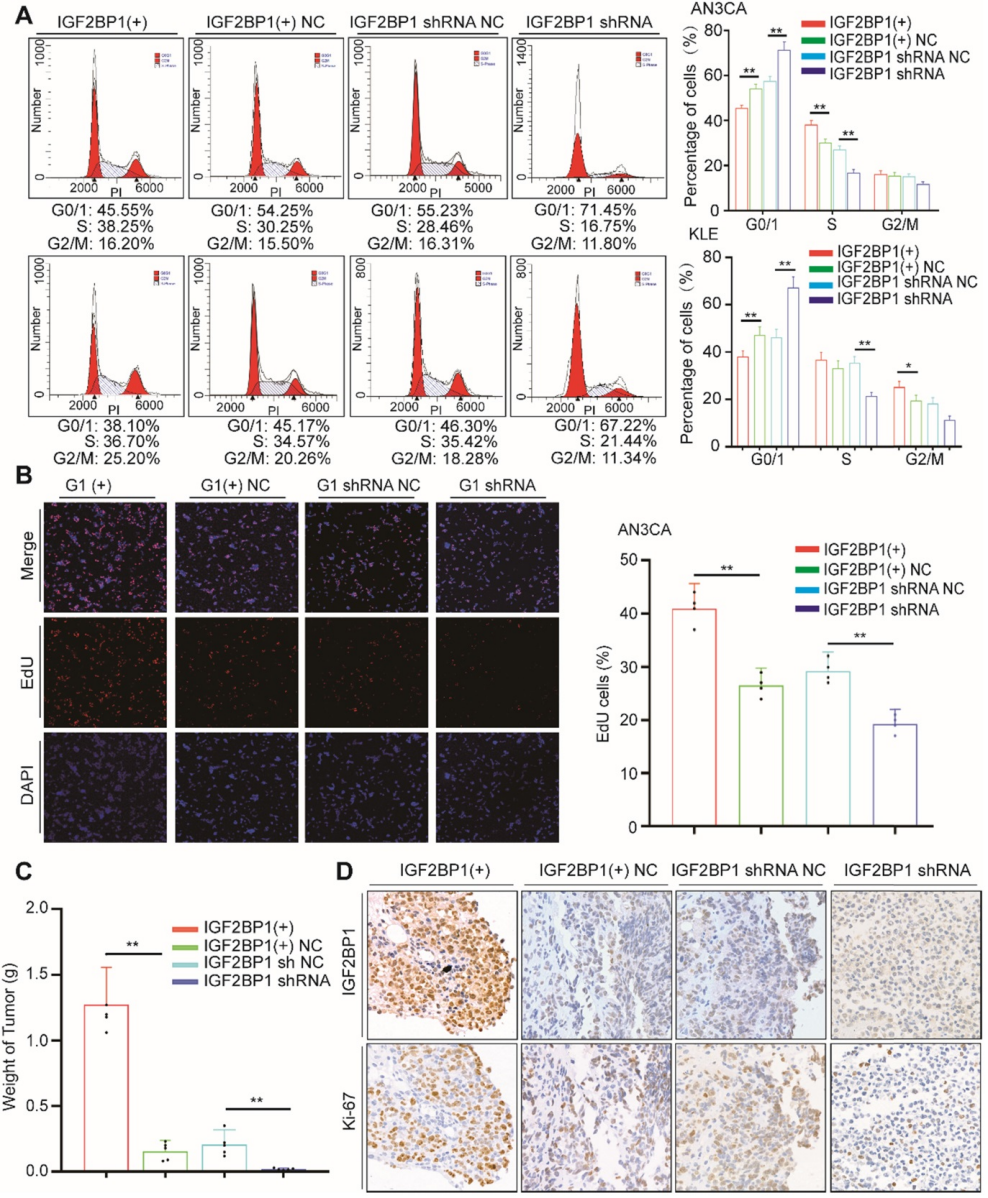
** IGF2BP1 promotes endometrial cancer cell proliferation.** (**A**) Flow cytometry analysis of cell cycle distribution after overexpression or silencing of IGF2BP1; (**B**) EdU assays evaluating cell proliferation; (**C**) Tumor growth assays based on Figure S2. Mice were injected with the AN3CA cell lines (n = 5 mice/group) and tumor weight after 4 weeks; (**D**) IHC staining of IGF2BP1 in tumor samples from (Figure S2). IGF2BP1(+): Overexpression of IGF2BP1 by lentivirus. IGF2BP1(+) NC: Negative control lentiviral vector. IGF2BP1 shRNA: knockdown of IGF2BP1 by shRNA lentivirus. IGF2BP1 shRNA NC: Negative control shRNA lentivirus. Error bars indicate means ± SD, *P < 0.05, **P < 0.01.

As previously described, we assessed the impact of IGF2BP1 on cell cycle distribution through flow cytometry. After IGF2BP1 overexpression, the number of cells in phase G0/1 decreased, while numbers in phase S cells increased (Figure 2A). However, when IGF2BP1 was silenced, the number of cells in phase S decreased (Figure 2A, S1D). EdU results were consistent with cell cycle analysis. After IGF2BP1 overexpression, phase S cells increased significantly, although after IGF2BP1 silencing, phase S cells significantly decreased in number (Figure 2B, S1E).

We examined the effect of IGF2BP1 on EC *in vivo* using SCID/Beige mice injected with AN3CA cells (either overexpressing or silenced IGF2BP1) into the abdominal cavity. In the mice injected with cells overexpressing IGF2BP1, the tumors were larger compared to those in the negative control group (Figure 2C and Figure S2). Ki67 expression also significantly increased, see Figure 2D for details. By contrast, after silencing IGF2BP1, tumor weight significantly reduced (Figure 2C and Figure S2) and in turn Ki67 significantly reduced (Figure 2D). These results demonstrate that IGF2BP1 promotes EC cell proliferation *in vitro*, accelerates the tumor cell cycle and promotes EC tumor growth *in vivo*.

### IGF2BP1 recognizes and binds PEG10 mRNA in an m^6^A-dependent manner

IGF2BP1 is a RBP that directly regulates the function of mRNA. To explore the molecular mechanism by which IGF2BP1 accelerates the cell cycle and increases tumor cell proliferation, we first performed RIP to identify mRNAs which interact with IGF2BP1. Sequencing results identified 902 mRNAs which interact with IGF2BP1 proteins (Table S5). GO and KEGG analysis showed that enriched mRNA is related to the molecular functions of RNA stability, cell proliferation and cell cycle regulation (Figure S3A-B).

**Figure 3:**
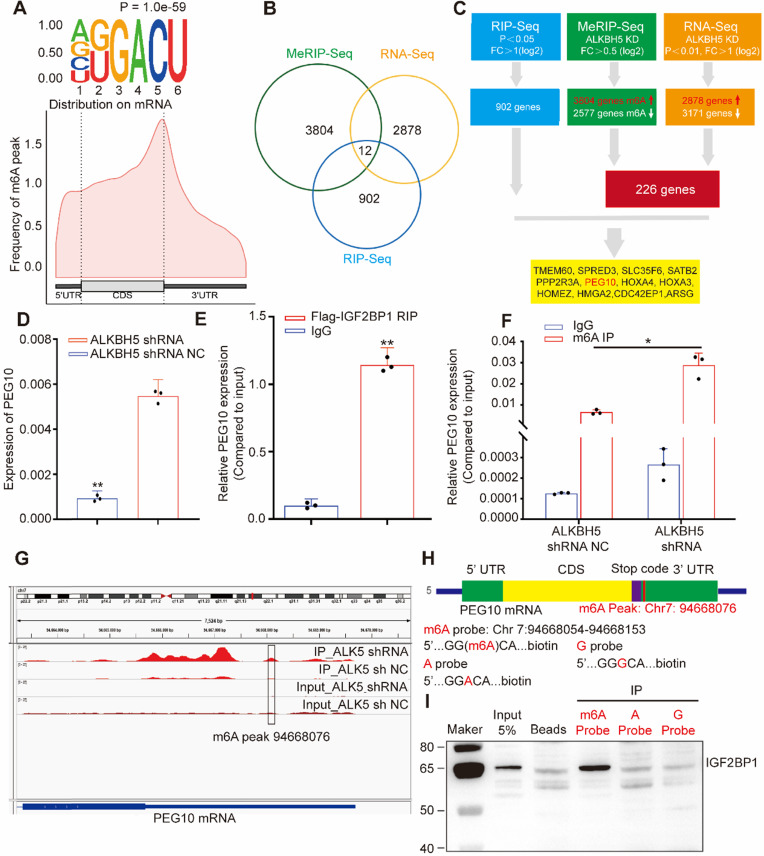
** IGF2BP1 and ALKBH5 recognize and regulate m^6^A in PEG10 mRNA** (**A,** upper) Top motif identified by HOMER with m^6^A-seq peaks, (A, down) Distribution of new m^6^A peaks in mRNA detected by MeRIP-seq after knocking down ALKBH5 expression; (**B**) Venn diagram shows the genes enriched by MeRIP-seq, RNA-seq and RIP-seq; (**C**) Schematic of downstream analysis for RIP-seq, MeRIP-Seq and mRNA-seq; (**D**) Quantitative PCR confirming elevated PEG10 mRNA expression after ALKBH5 knockdown; (**E**) RIP-PCR validating IGF2BP1 binding to PEG10 mRNA; (**F**) MeRIP-PCR confirmingthe m^6^A peak in the 3' untranslated region of PEG10 mRNA is regulated by ALKBH5; (**G**): m^6^A abundances in PEG10 mRNA transcripts in ALKBH5 knockdown (IP and input) and negative control (IP and input); (**H**) Position of m^6^A peak in PEG10 mRNA (top). RNA probe sequences for RNA pull down (bottom); (**I**) IGF2BP1 recognizes the m^6^A site in the 3′UTR of PEG10 mRNA as shown by RNA pull down assays. Error bars indicate means ± SD, *P < 0.05, **P < 0.01.

Considering that IGFBP1 recognizes m^6^A sites in mRNA and that m^6^A modification is regulated by methyltransferases and demethylases, we used lentivirus-mediated shRNA to silence demethylases ALKBH5 expression in cells (Figure S3C-D). We then analyzed m^6^A modifications across mRNAs in tumor cells using MeRIP-seq. The results showed that 3804 genes acquired a new m^6^A peak and 2577 genes lost an m^6^A peak after ALKBH5 knockdown. The most common motif of these emerging peaks was DRACH (Figure 3A, upper). The positions of these peaks occur mainly near the stop codon (Figure 3A, lower).

GO analysis showed that enriched mRNAs were related to RNA metabolization and RNA transport (Figure S3E). Due to the fact that the main molecular function of ALKBH5 is to remove m^6^A, we focused on genes that acquired an m^6^A peak. In order to detect the effect of RNA demethylase ALKBH5 on mRNA expression, we knocked down ALKBH5 expression in AN3CA and analyzed changes in mRNA expression using RNA-seq. Results showed that the expression of 2878 genes increased, while 3171 genes decreased (Figure S3F, Table S6). Previous studies have reported that IGF2BP1 is associated with mRNA stability 12 therefore, we screened for enhanced mRNAs. By combining RIP-seq, MeRIP-seq and RNA-seq results, we screened out 12 candidate genes (Figure 3B-C). RIP-PCR, q-PCR, MeRIP-PCR and western blotting were further used to verify candidates.

Results showed that mRNA and protein expression in PEG10 significantly increased after ALKBH5 silencing (see Figure 3D, and supplementary materials, Figures S3D, S4A-B). RIP-PCR analysis verified that IGF2BP1 interacts with PEG10 mRNA (Figure 3E). MeRIP-PCR and MeRIP-seq demonstrated that m^6^A modification in the 3'UTR region of PEG10 mRNA was regulated by ALKBH5 (Figure 3F-G, S3G). These results suggest that IGF2BP1 recognizes m^6^A in the PEG10 mRNA 3'UTR region and interacts with PEG10 mRNA.

RNA pull-down experiments were performed using an RNA probe for the PEG10 mRNA 3'UTR sequence (Figure 3H). We can confirm that IGF2BP1 binds to m^6^A sites, with a weaker binding ability to non-m^6^A sites (Figure 3I). These results indicate that IGF2BP1 recognizes the PEG10 mRNA 3'UTR region although this is m^6^A-dependent.

### IGF2BP1 promotes PEG10 mRNA stability and expression

**Figure 4:**
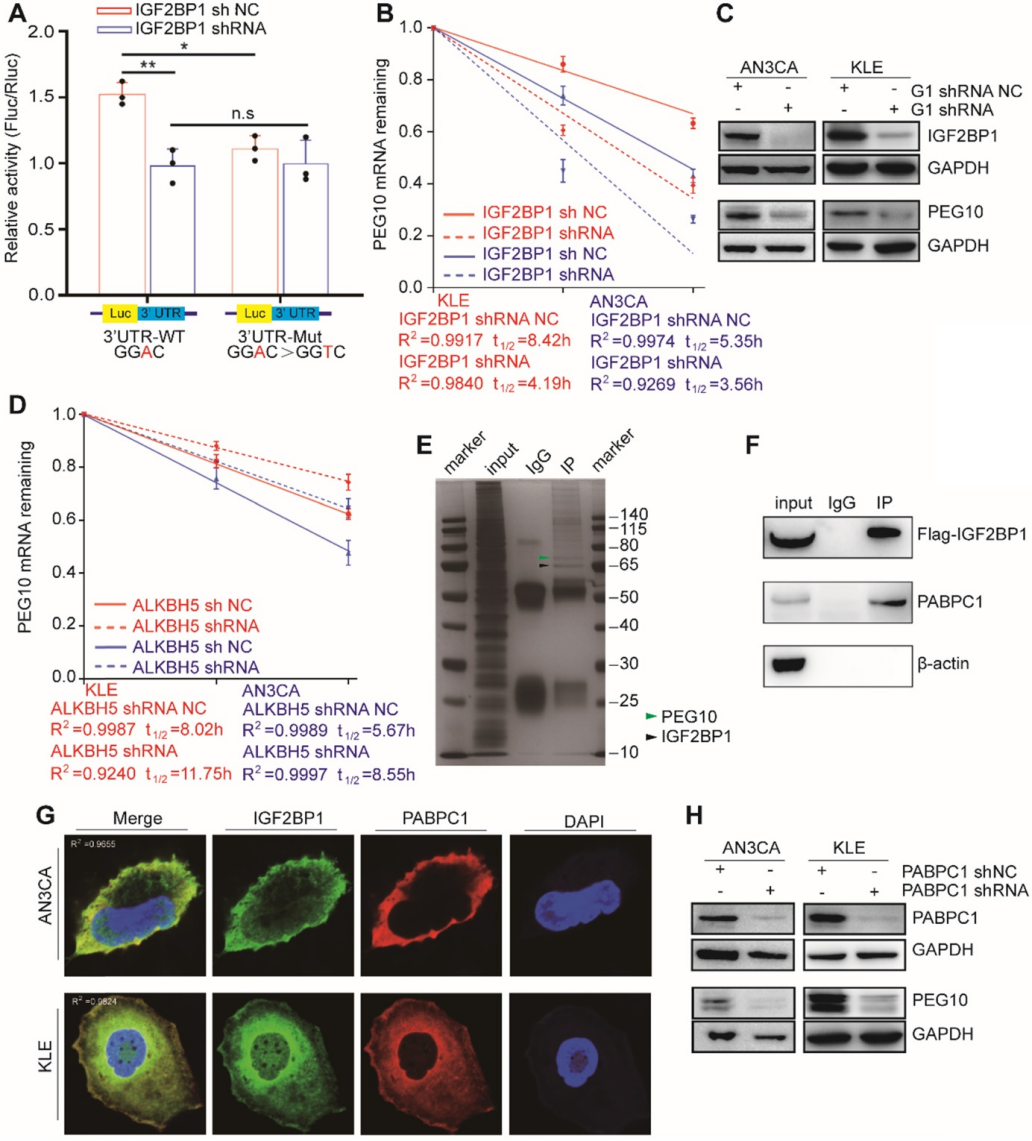
** IGF2BP1 regulates PEG10 mRNA stability and expression in an m^6^A-dependent manner.** (**A**) Relative activity of the wild-type or mutant PEG10 3′UTR luciferase reporter in AN3CA cells expressing IGF2BP1 shRNA or control; (**B**) Shortened RNA lifetime of PEG10 mRNA after IGF2BP1 expression knockdown; (**C**) Decreased PEG10 protein expression after IGF2BP1 knockdown; (**D**) Increased lifetime of PEG10 mRNA after ALKBH5 silencing; (**E**) Flag-IGF2BP1 co-immunoprecipitated proteins shown by silver staining. Lane 1: marker, Lane 2: input (50-fold dilution), lane 3: normal IgG control antibody, lane 4: IGF2BP1-IP (Flag-antibody). Flag-IGF2BP1 protein (black arrow, 65-70 kd) and PABPC1 (green arrow, 71kd) are displayed and verified by western blotting; (**F**) PABPC1 was co-immunoprecipitated with FLAG-IGF2BP1; (**G**) Co-localization of IGF2BP1 and PABPC1 in cells as shown by immunofluorescence analysis; (**H**) Decreased PEG10 protein expression after PABPC1 knockdown. Error bars indicate means ± SD, *P < 0.05, **P < 0.01, n.s indicates no significance.

To identify the biological function in the PEG10 3'UTR sequence, we performed luciferase assays using reporter constructs which contained either a wild-type or the mutant 3'UTR sequence. In AN3CA cells with normal IGF2BP1 protein expression, luciferase reporter activity significantly enhanced in cells transfected with the wild-type construct, indicating that this sequence enhances mRNA stability (Figure 4A). After silencing IGF2BP1 expression, luciferase activity of cells transfected with wild-type sequences decreased significantly, while cells transfected with mutant vector did not show significant changes. This result indicates that IGF2BP1 is important in the wild-type sequence in stabilizing mRNA (Figure 4A). We further confirmed these findings using an RNA decay assay. After IGF2BP1 was silenced, the stability of PEG10 mRNA significantly reduced (Figure 4B) and PEG10 protein expression significantly decreased (Figure 4C). This further suggests that IGF2BP1 regulates the stability and expression of PEG10 mRNA.

Our previous results show that upon ALKBH5 silencing, an m^6^A site appears in the PEG10 3'UTR region, which can be recognized by IGF2BP1. Therefore, we speculated that ALKBH5 could affect PEG10 mRNA stability and protein expression. Consistent with this hypothesis, the stability of PEG10 mRNA significantly enhanced after silencing ALKBH5 in cells, as can be seen through RNA decay analysis (Figures 4D, S4C), while PEG10 protein expression significantly increased (Figures S3D, S4A).

### PABPC1 collaborates with IGF2BP1 to stabilize PEG10 mRNA

Previous studies have shown that IGF2BP1 functions in mRNA stabilization by binding to mRNA stabilizers such as PABPC1, HuR and MATR3 12. In order to identify proteins which support IGF2BP1 in stabilizing PEG10 mRNA, we performed CoIP-MS and identified 381 potential IGF2BP1-interacting proteins (Figure 4E, Table S8). GO and KEGG analysis then revealed that these proteins were mainly related to RNA stability and translation (Figure S4D-E). We found that PABPC1 becomes highly enriched in FLAG-IGF2BP1 immunocomplexes from AN3CA cells (Figure 4F). Confocal fluorescence imaging analysis revealed that PABPC1 and IGF2BP1 co-localize in AN3CA and KLE cells (Figure 4G).

PABPC1 is a classical poly-A tail binding protein that can assist in mRNA stabilization 16. PABPC1 expression was silenced in cells to analyze the effect of PABPC1 on PEG10 mRNA. RNA decay assays again showed that PEG10 mRNA instability increases significantly upon PABPC1 silencing (Figure S4F). There was also a decrease in PEG10 protein expression after PABPC1 knockdown (Figure 4H). When we silenced the expression of both IGF2BP1 and PABPC1, PEG10 mRNA instability in cells further increased (Figure S4G), which suggests that PABPC1 and IGF2BP1 have a synergistic effect on maintaining PEG10 mRNA stability.

### PEG10 accelerates EC cell proliferation and is associated with poor prognosis in EC

**Figure 5:**
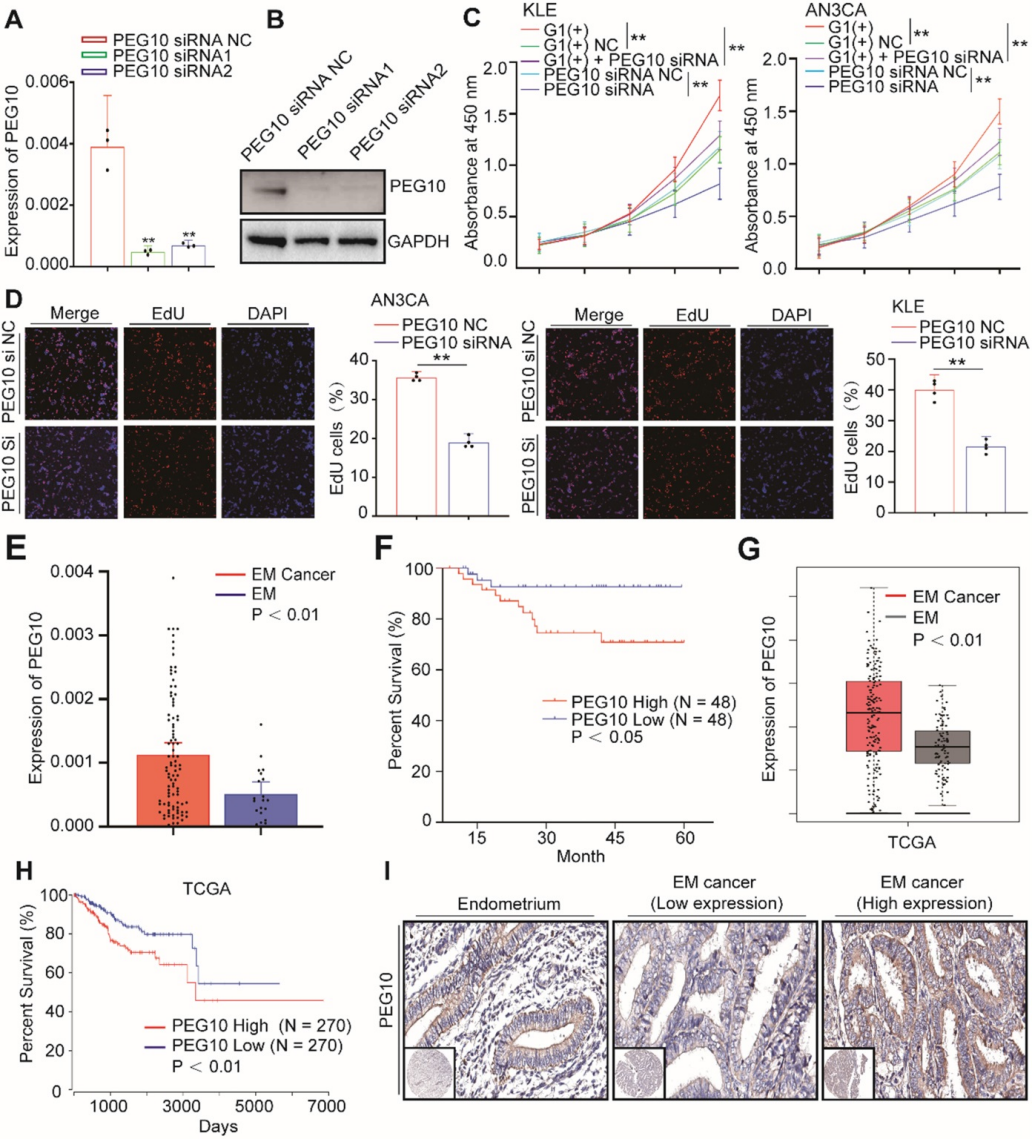
** PEG10 expression promotes cell proliferation and is associated with poor prognosis in EC.** (**A, B**) Real-Time PCR and WB validated siRNA-mediated silencing of PEG10 expression; (**C**) CCK-8 analysis showed that siRNA-mediated silencing of PEG10 expression inhibited the proliferation-promoting effect of IGF2BP1overexpression; (**D**) Silencing PEG10 expression reduced cell proliferation activity as shown by EdU assay; (**E**) Overexpression of PEG10 mRNA in endometrial cancer; (**F**) High expression of PEG10 relating to poor prognosis in endometrial cancer; (**G**) Analysis of TCGA data shows high expression of PEG10 in endometrial cancer; (**H**) TCGA data analysis of high PEG10 expression and prognosis in endometrial cancer; (**I**) Expression of PEG10 protein in endometrial tissue and endometrial cancer analyzed by tissue array and IHC. Error bars indicate means ± SD, **P < 0.01. Define high and low expressions PEG10 according to the median.

Previous studies have shown that PEG10 promotes tumor cell proliferation and invasion in a variety of tumors 17; however, their function in EC has not been reported. We therefore examined whether PEG10 expression promotes EC cell proliferation. We used PEG10 siRNA to silence PEG10 expression (Figure 5A-B) and found the cellular proliferation rate in these cells significantly reduced compared with controls (Figure 5C).

EdU analysis shows that cell proliferation did decrease significantly after PEG10 expression was silenced (Figure 5D). Furthermore, while IGF2BP1 overexpression accelerates cell proliferation, silencing PEG10 expression in cells with IGF2BP1 overexpression reduces the proliferation promoting effect (Figure 5C). Further EdU experiments show that silencing PEG10 can reduce proliferation activity of cells which stabilize IGF2BP1 overexpression (Figure S5A). Cell cycle analysis showed that cells in phase G0/1 increased, while in phase S decreased (Figure S5B). These results indicate that IGF2BP1-PEG10 axis promotes the proliferation of EC cells.

Consequently, we examined PEG10 mRNA expression in normal endometrial and EC tissues, and found it to be significantly greater in tumor samples (Figure 5E). We also found that the survival of patients negatively correlated with PEG10 expression (Figure 5F). TCGA data analysis further showed that PEG10 expression was significantly higher in EC tissue and is related to poorer prognoses (Figure 5G-H).

Tissue array and IHC were used to analyze PEG10 protein levels in tumor tissues compared with endometrial samples. Similar to the mRNA results, PEG10 protein showed negative/weak expression in normal endometria, but the expression significantly increased in EC (Figure 5I). The intensity of PEG10 protein expression positively correlated with FIGO stages and depth of invasion (Table S4), although there was no significant correlation with tumor cell grade, lymphovascular invasion or lymph node metastasis (Table S4). Additionally, we found that the expression level of IGF2BP1 positively correlated with PEG10 expression level in EC (Figure S5C-D). Together these results indicate that PEG10 is highly expressed and plays a role in EC promotion.

### PEG10 inhibits the gene expression of cell cycle-regulators

**Figure 6:**
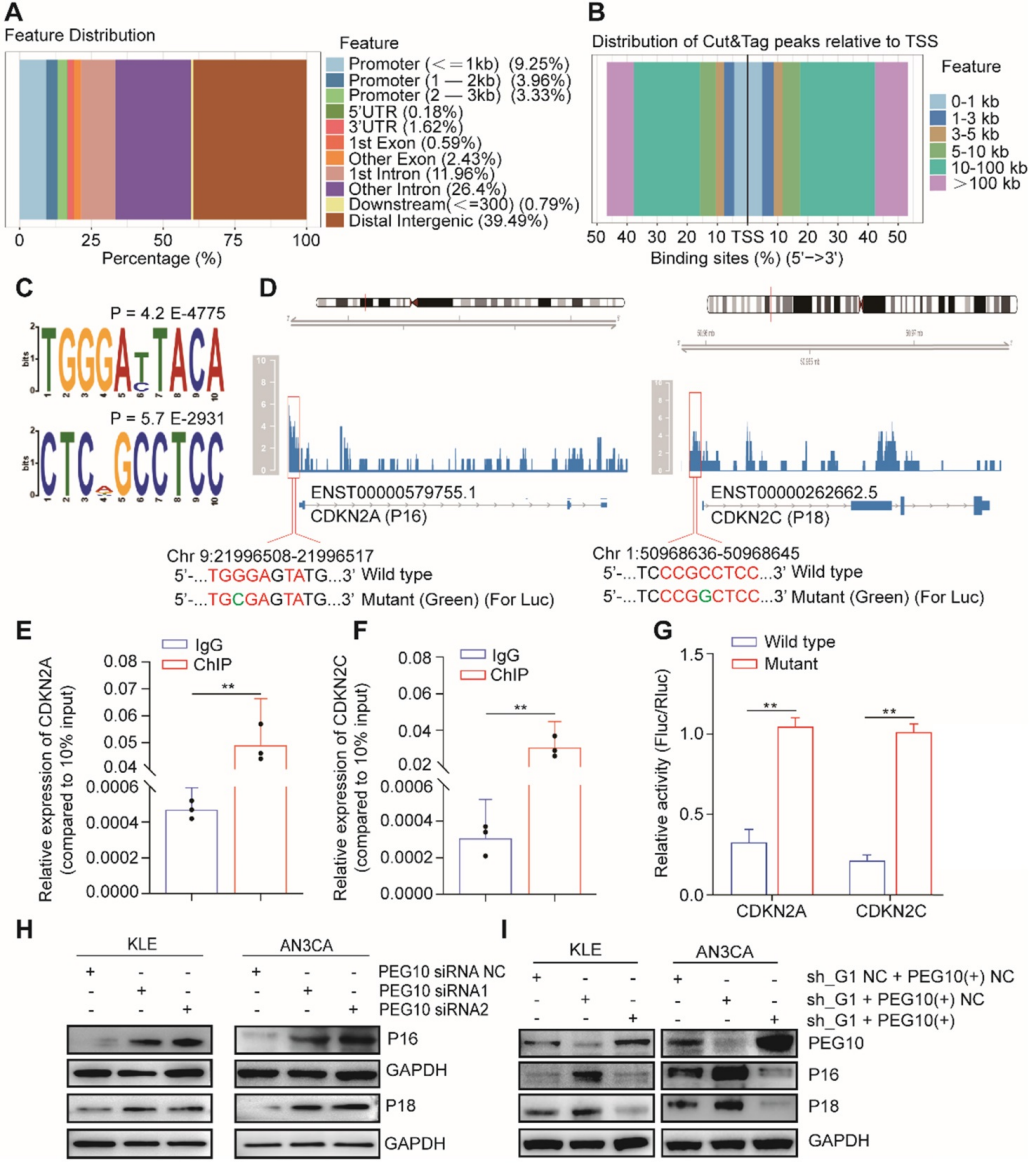
** PEG10 regulates the expression of cell cycle-related genes.** (**A**) The distribution of PEG10 protein-bound DNA fragments analyzed by CUT&Tag-seq; (**B**) Distribution of DNA fragments relative to transcription start site (TSS) analyzed by CUT&Tag-seq; (**C**) PEG10 binding motif identified by DREME; (**D**) PEG10 protein binding to the promoter regions of p16 and p18 genes (red frame); (**E-F**) ChIP-PCR confirming PEG10 binding to the sequence of the promoter region of p16 and p18 genes; (**G**) Relative activity of the wild-type or mutant promoter luciferase reporter in AN3CA cells expressing PEG10 protein; (**H-I**) Western blotting analysis of the change of expression of cell cycle-related proteins after PEG10 knockdown or overexpression. Error bars indicate means ± SD, **P < 0.01.

PEG10 contains a CCHC-type zinc finger domain that has DNA binding function 17. We used CUT&Tag-seq to detect DNA sequences that bind with PEG10 protein and found that PEG10 binds promoter regions (Figure 6A). PEG10 exhibits effects on cell proliferation and the range of promoter lengths varies substantially. Therefore, we focused on genes related to the cell cycle with a promoter range of less than 1 kb from the transcription start site (Figure 6B). Motif analysis shows that PEG10 prefers to combine TGGGAYTACA and CTCNGCCTCC motif (Figure 6C). By analyzing the cell cycle-related genes which bind to PEG10, we found that PEG10 binds promoter sequences for p16, p18, p19, p21, p57, CDK2, CDK6, CCND1, CCNE1, and CCNE2 genes (see Table S9). The promoter regions of p16 and p18 genes have high peak enrichment (>4-fold), and there are motifs that can bind to PEG10 protein near peaks, <350bp (Figure 6D).

ChIP-PCR confirmed that PEG10 can bind to the promoter sequences of p16 and p18 genes (Figure 6E-F). Luciferase report analysis also shows that PEG10 can inhibit gene expression after binding to wild-type sequences (Figure 6G). Silencing PEG10 expression by siRNA can also promote P16 and P18 protein expression (Figure 6H), while the rescue expression of PEG10 genes in IGF2BP1 silenced cells can inhibit P16 and P18 protein expression (Figure 6I). This suggests that PEG10 may be a transcriptional inhibitor which inhibits p16 and p18 gene expression.

In order to explore whether the IGF2BP1-PEG10 axis can regulate other cell cycle-related genes, we rescued PEG10 expression in IGF2BP1 stable silencing cells. PCR and WB showed that IGF2BP1 silencing can promote the expression of p19, p21, CDK2, CCND1, CCNE1 genes, while rescue PEG10 expression can reverse the effect of IGF2BP1 silencing (Figure S6A-B). On the other hand, silencing PEG10 mRNA expression could promote the expression of these genes (Figure S7A-B). However, this trend has not been observed in the regulation of p57, CDK6 and CCNE1 gene expression.

## Discussion

We found that IGF2BP1 enhances PEG10 expression and promotes EC cell proliferation by recognizing the m^6^A site in PEG10 mRNA. Previous research has shown that IGF2BP1 belongs to the IGF2BP family and contains two RNA recognition motifs and four hnRNP-K homology domains 18. Other researchers have also found that IGF2BP1 is not expressed in mature tissue though it can be detected in a variety of tumors 12, which suggests IGF2BP1 might be a oncogene. Early reports described IGF2BP1 as an RBP, involved in the regulation of mRNA and affecting tumor cell functions 19. In this study, we found high IGF2BP1 expression in EC and that IGF2BP1 is a factor which can influence patient survival. IGF2BP1 also appears to increase proliferation and we observed that IGF2BP1 overexpression promotes the formation of EC tumors. These findings combined indicate that increased IGF2BP1 expression plays a key role in promoting EC progression.

Reading, writing and erasing m^6^A sites, plays a regulatory role in the expression of some key proteins, which is more likely to correlate with the progression of malignancies 6. Liu *et al.* found that decreased METTL3 expression and METTL14 gene mutation decreases the m^6^A level and contributes to the progression of EC. YTHDF1/2 is involved in PHLPP2, PRR5, PRR5L and mTOR regulation 20; however, IGF2BP1 is also a m^6^A reader. To date, only a few target genes have been identified as proving relevant to m^6^A-dependent tumor formation. In this study, we found that the 3'UTR of PEG10 has a m^6^A modification which is removed by ALKBH5 and recognized by IGF2BP1. After IGF2BP1 reading, PEG10 mRNA is stable, leading to increased PEG10 protein expression. Therefore, we not only gained insight into the function of IGF2BP1 in EC cells, but also found that IGF2BP1 is m^6^A-dependent when regulating PEG10 expression. We are the first to report that PEG10 is a target gene which is regulated by IGF2BP1 via a m^6^A modification. PEG10 is likely not to be the only target gene regulated by IGF2BP1, although further research is required to identify other target genes and clarify our understanding of the functions and mechanism of IGF2BP1.

The regulation of reader proteins in recognizing m^6^A functions is undoubtedly complex although, our* in vitro* experiment suggests that IGF2BP1 binds the m^6^A site. The function of other proteins in the regulation of mRNA stability and expression cannot be ruled out, presently 6. For example, Wang *et al.* found that EIF binds YTHDF1 and participates in mRNA translation regulation 21. Likewise, PABPC1 is a poly-A binding protein which binds mRNA, preventing nuclease shortening the mRNA tail and affecting mRNA functions 22. However, we found that PABPC1 and IGF2BP1 bind to one another and their spatial expressions highly correlate. In terms of function, there may be an interaction between the two proteins in stabilizing mRNA, although we cannot describe the exact relationship between PABPC1 and IGF2BP1 in the m^6^A regulation pathway. We found that PABPC1 plays a synergetic role with IGF2BP1 in the regulation of PEG10 mRNA stabilization. Therefore, we hypothesize that IGF2BP1, after recognizing a m^6^A site, interacts with other mRNA binding proteins to form a large protein complex to complete the functional regulation of mRNAs. Of course, this protein complex may contain multiple factors; however, PABPC1 appears to be a key factor. This assertion even though evidence-based, is tentative and requires further research.

PEG10 is a paternally expressed imprinted gene. Paternally expressed imprinted genes are often thought to have a role in cell proliferation, while maternally expressed imprinted genes conversely, suppress proliferation 23. Paternally imprinted genes are expressed in a variety of human cancers and are regarded as oncogenes 17. PEG10 overexpression has been associated with several malignancies, such as liver cancer, pancreatic cancer and breast cancer 24-26. In this study, we found that PEG10 is also highly expressed in EC and is directly related to patient survival. A large number of studies have focused on PEG10 gene transcription and promoter methylation to investigate the relationship between PEG10 and tumor progression 26-27. However, we have provided a new perspective for understanding PEG10 expression regulation by demonstrating that IGF2BP1 is an m6A-dependent post-transcriptional regulator of PEG10 expression.

The most important molecular mechanism of cell proliferation is cell cycle acceleration, which is regulated by CDK-cyclin complexes and cyclin-dependent kinase inhibitors 28. Cyclin D-CDK4/6 and cyclin E-CDK2/4 complexes are also keys which regulate the G1/S checkpoint and promote cell cycle progression to phase S 29-30. P16, P18, and P19 inhibit the function of the cyclin D-CDK4/6 complex 31-33, while P21, P27, P57 and other proteins inhibit the cyclin E-CDK2/4 complex, leading to cell cycle inhibition 34. Numerous studies have provided a modicum of insight into the role of PEG10 in cell proliferation, though only a few have examined the molecular mechanism 35-36. For example, Akamatsu *et al.* recently reported that knockdown of PEG10 increased the expressions of p21 and p27. PEG10 is a key factor, and is directly involved in the regulation of key cell cycle proteins 37. However, while PEG10 was thought to directly bind DNA 38, the genes regulated by PEG10 remain unknown.

In this study, we found that PEG10 indeed binds a wide range of DNA sequences, including a large number of promoter regions of cell cycle regulatory genes. PEG10 protein can bind to the promoter regions of p16 and p18 genes, inhibiting RNA and protein expression. This indicates that PEG10 may accelerate cell cycle progression by regulating the expression of cyclin dependent kinase inhibitors. Notably, after PEG10 silencing, the expressions of CDK2, cyclin D1, and cyclin E2 were also significantly enhanced. This further suggests that PEG10 negatively regulates the expression of some components in cyclin-CDK complexes. These two molecular effects of PEG10 on cell cycle components appear to be countervailing. Therefore, it is worth noting that PEG10 showed no inhibitory effect on the expressions of CDK6 and cyclin E1, indicating that PEG10 does not regulate the expression of components in all cyclin-CDK complexes.

Nevertheless, our findings show that PEG10 inhibits p16, p18, p19 and p21, and these cyclin dependent kinase inhibitors exhibit potent effects on the functions of cyclin D-CDK4/6 and cyclin E-CDK2/4 complexes. Therefore, we speculate that the primary molecular mechanism in regulating cell proliferation is PEG10, which promotes cell cycle progression by inhibiting the expression of cyclin dependent kinase inhibitors. Of course, we cannot omit that the expression changes of CDK2, cyclin D1 and cyclin E2 are a negative feedback regulation mechanism which occur after cell cycle inhibition. Again, further research is required to understand the nuances involved in the molecular mechanism of PEG10 in cell cycle regulation.

In conclusion, we found that IGF2BP1 is highly expressed in EC and this overexpression of IGF2BP1 correlates with poor prognosis for EC patients. Mechanistically, IGF2BP1 recognizes and then stabilizes PEG10 mRNA in an m6A-dependent manner and enhances PEG10 expression, thereby accelerating the cell cycle, and promoting EC progression.

## Supplementary Material

Supplementary figures and tables.

## Abbreviations

EC: endometrial cancer
m^6^A: N6-methyladenosine
METTL3: Methyltransferase Like 3
METTL14: Methyltransferase Like 14
EMT: epithelial-mesenchymal transition
FTO: Fat mass-and obesity-associated protein
ALKBH5: AlkB Homolog 5
YTHDFs: YTH N6-Methyladenosine RNA Binding Protein
IGF2BPs: Insulin Like Growth Factor 2 mRNA Binding Protein
UTR: Untranslated regions
PEG10: Paternally Expressed 10
PABPC1: Poly(A) Binding Protein Cytoplasmic 1
p16: Cyclin Dependent Kinase Inhibitor 2A
p18: Cyclin Dependent Kinase Inhibitor 2C
p19: Cyclin Dependent Kinase Inhibitor 2D
p21: Cyclin Dependent Kinase Inhibitor 1A
CDK2: Cyclin Dependent Kinase 2
CDK6: Cyclin Dependent Kinase 6
