# Supplementary material for: IGF2BP1 overexpression stabilizes PEG10 mRNA in an m6A-dependent manner and promotes endometrial cancer progression
Source: Theranostics. 2021 Jan 1;11(3):1100–14. doi: 10.7150/thno.49345 (PMC7738899; doi:10.7150/thno.49345)
Supplement: Supplementary file 1 — Supplementary figures and tables. [file thnov11p1100s1.pdf]

SUPPLEMENTAL MATERIALS

Overexpression of IGF2BP1 stabilizes PEG10 mRNA in an m^6A-dependent manner and promotes endometrial cancer progression

Lin zhang, Yicong Wan, Zihan Zhang, Yi Jiang, Zhiyue Gu, Xiaoling Ma, Sipei Nie, Jing Yang, Jinghe Lang, Wenjun Cheng*, and Lan Zhu**

Supplementary Figures Legends

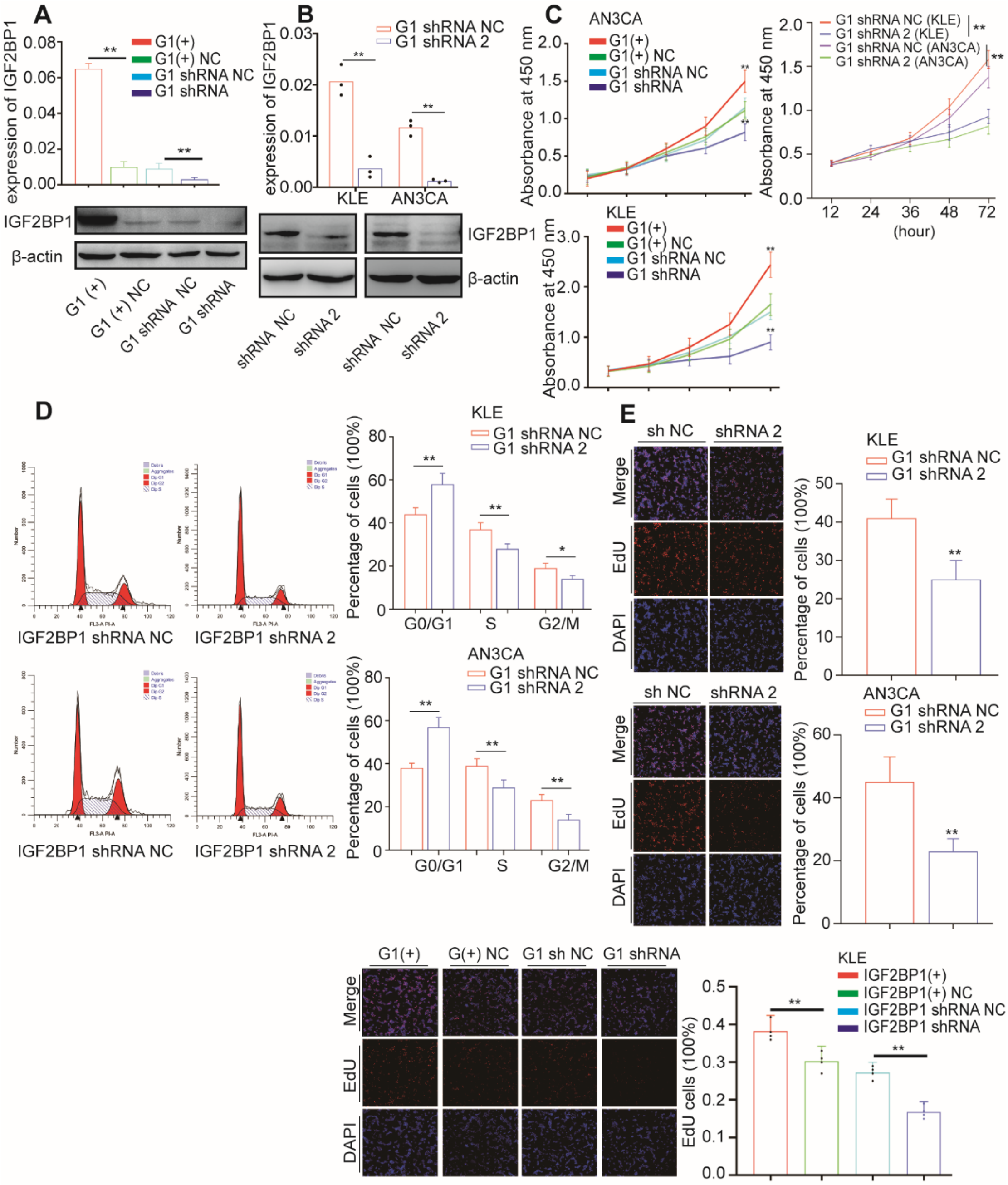

Figure S1: IGF2BP1 promotes the proliferation of EC cells. (a-b) Q-PCR and western blot analysis overexpression or knockdown of IGF2BP1 in EM cells. (c) IGF2BP1 promotes cell proliferation as shown by CCK-8 assays. (d) Flow cytometry analysis of cell cycle distribution after silencing of IGF2BP1. (e) EdU assays evaluated cell proliferation activity.

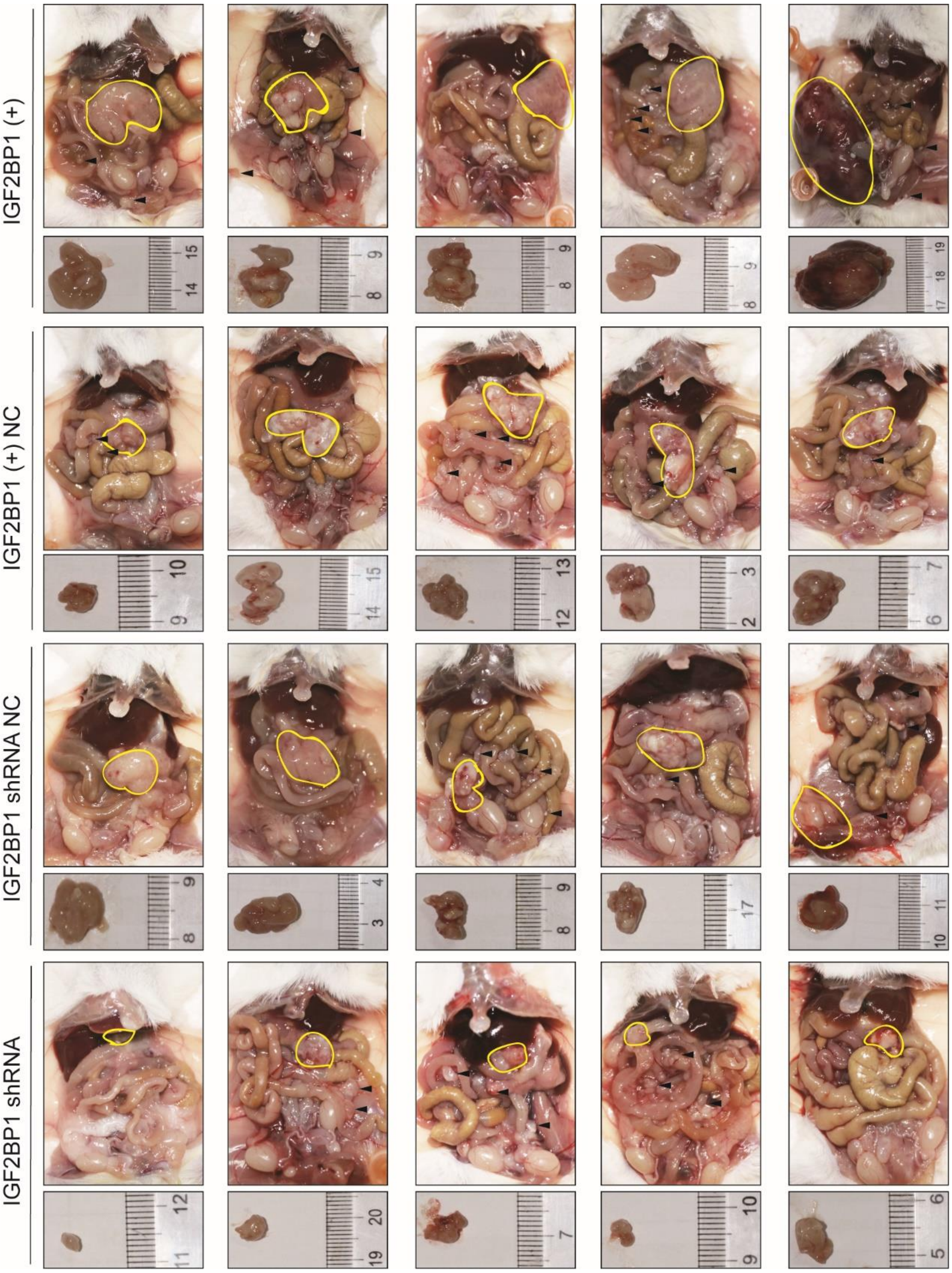

Figure S2: IGF2BP1 promotes endometrial cancer proliferation in a xenograft model. IGF2BP1 expression lentivirus (IGF2BP1 (+)) or IGF2BP1 shRNA lentivirus (IGF2BP1 shRNA) was transduced in AN3CA cells to enhance or silence gene expression. IGF2BP1 negative control (IGF2BP1 (+) NC) or shRNA negative control lentivirus (IGF2BP1 shRNA NC) served as controls. 5 mice in each group. The

yellow circle marks the main tumor in the abdominal cavity, The black arrow indicates a small tumor in the abdominal cavity.

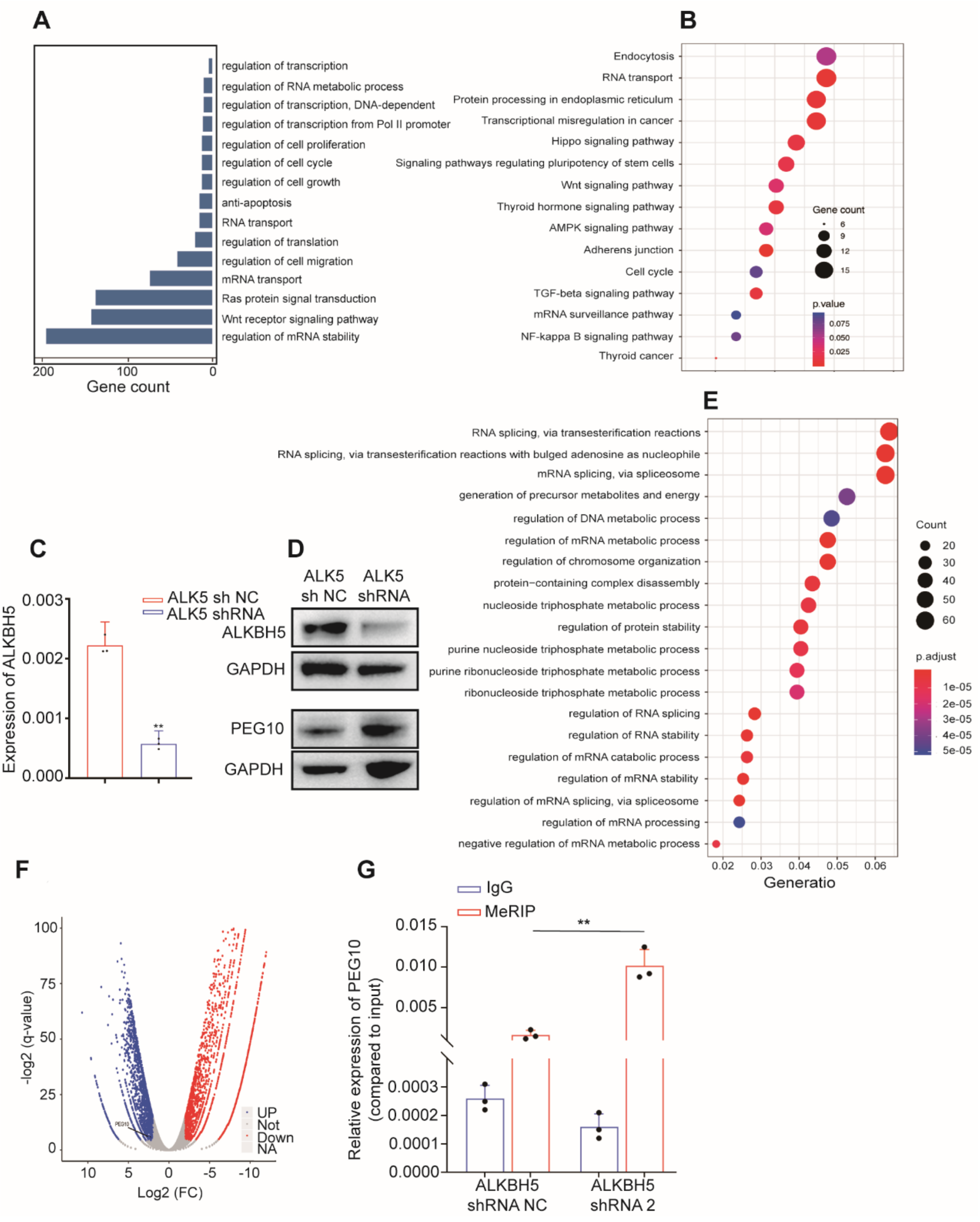

Figure S3: Bioinformatics analysis of the function of target genes. (a-b) GO and KEGG enrichment map of genes specifically enriched by IGF2BP1 RIP-seq. (c), (d, top) PCR and western blotting show that mRNA and protein expression of ALKBH5 was knocked down by shRNA compared with shRNA

negative control (NC). (d, bottom) PEG10 protein expression after knockdown of ALKBH5. (e) mRNAs that acquired m6A peak after ALKBH5 knockdown, analyzed by GO. (f) Volcano plots showing upregulation (blue) or downregulation (red) of genes after knockdown ALKBH5, as assessed by RNA-seq. (g) MeRIP-PCR confirmed that the m^{6}A peak in the 3' untranslated region of PEG10 mRNA is regulated by ALKBH5.

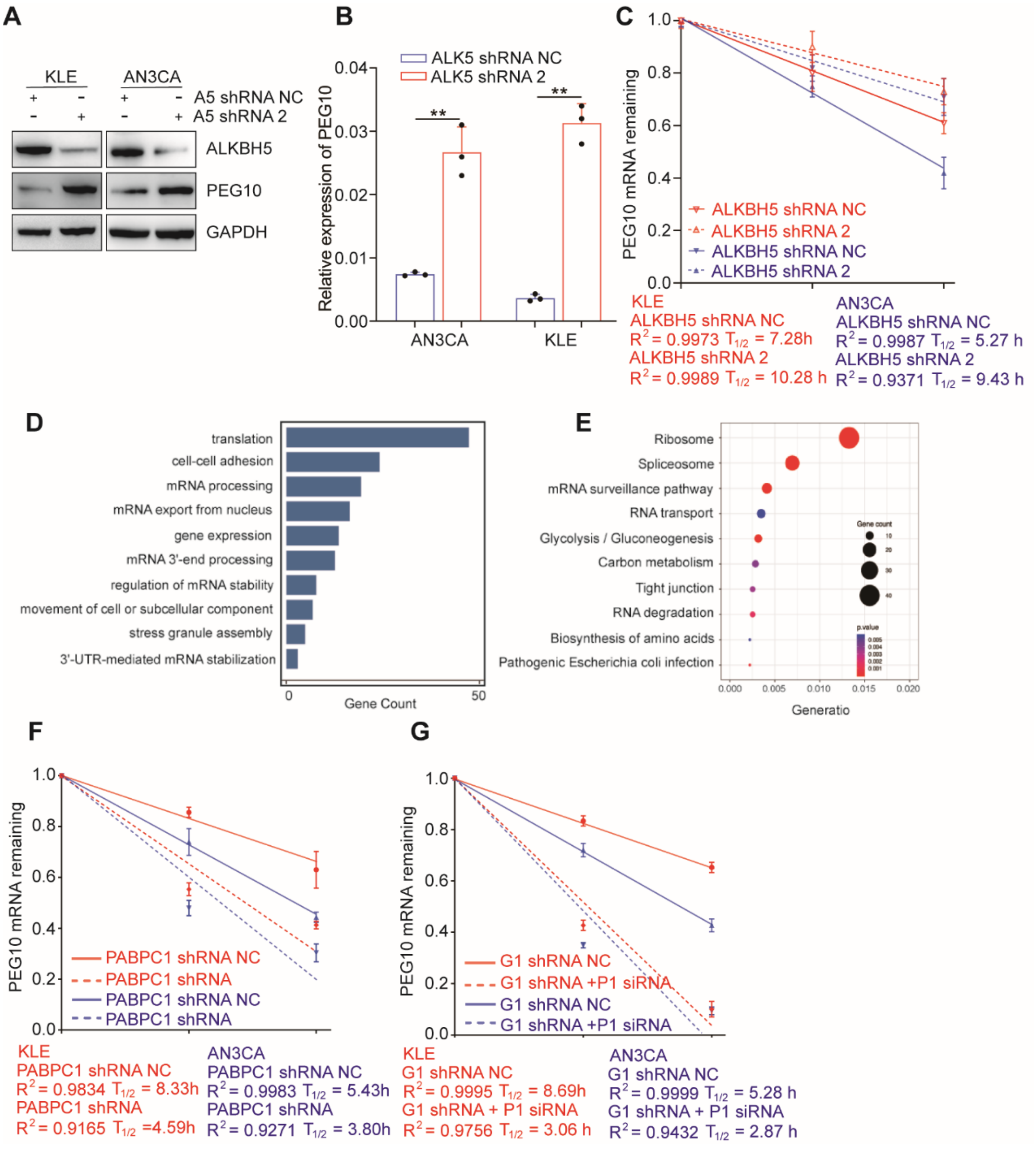

Figure S4: ALKBH5 regulates the m6A modification of the 3'UTR region in PEG10 mRNA. (a-b) PEG10 protein and mRNA expression after knockdown of ALKBH5. (c) Silencing ALKBH5 can

increase the half-life of PEG10 mRNA. (d-e) GO and KEGG analysis of proteins enriched by Flag-IGF2BP1 co-immunoprecipitation. (f) Shortened RNA lifetime of PEG10 mRNA after knockdown of PABPC1 expression. (g) Silencing IGF2BP1 and PABPC1 expression reduces the lifetime of PEG10 mRNA.

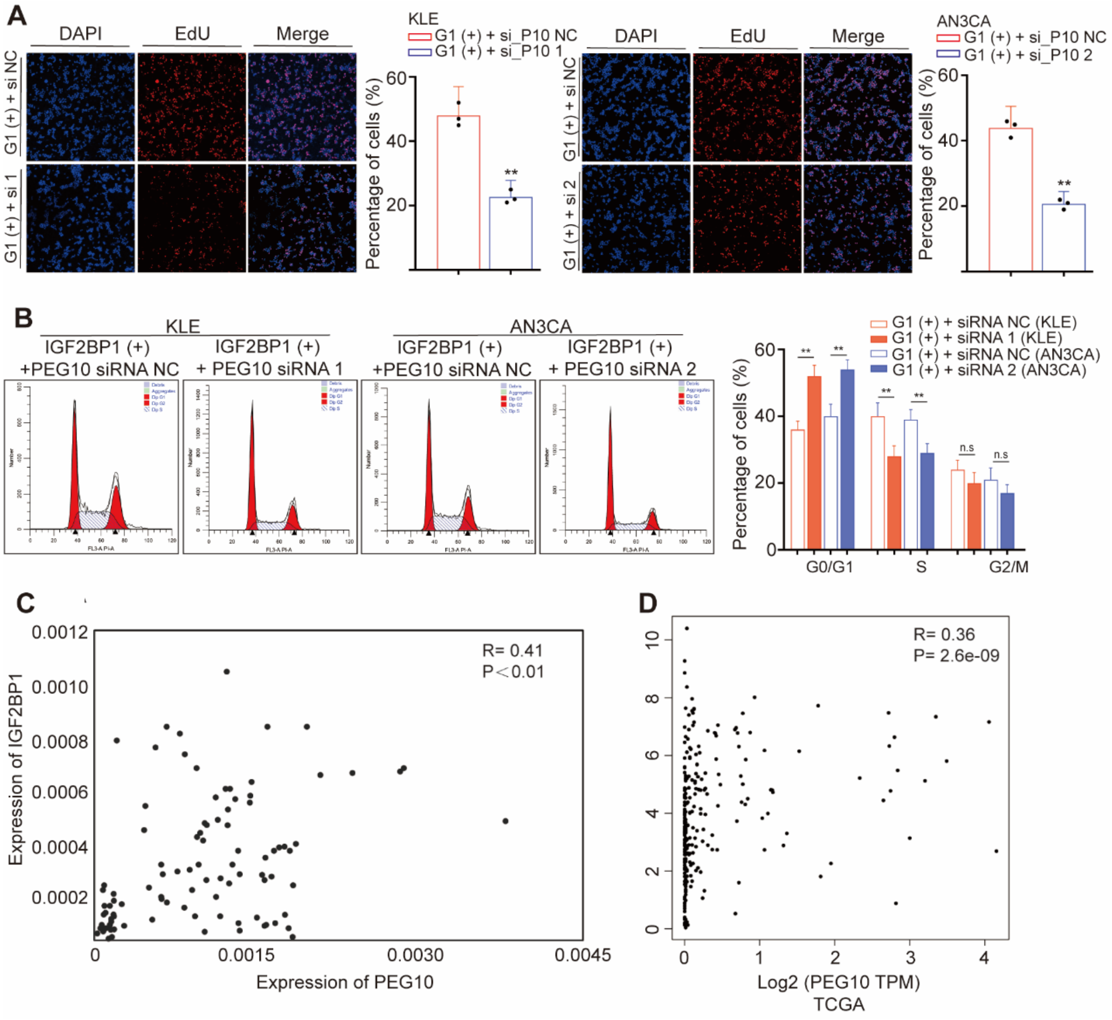

Figure S5: The IGF2BP1-PEG10 axis regulates cell proliferation activity and cell cycle. (a) EdU experiment confirmed that silencing PEG10 can inhibit the proliferation effect of IGF2BP1 over-expression. (b) Cell cycle analysis confirmed that knockdown PEG10 can reduce the ratio of cells in S phase and increase cells in G0/G1 phase. (c) Correlation analysis showed a positive correlation between IGF2BP1 and PEG10 expression. (d) TCGA data analysis showed that the expression of IGF2BP1 was positively correlated with PEG10 expression.

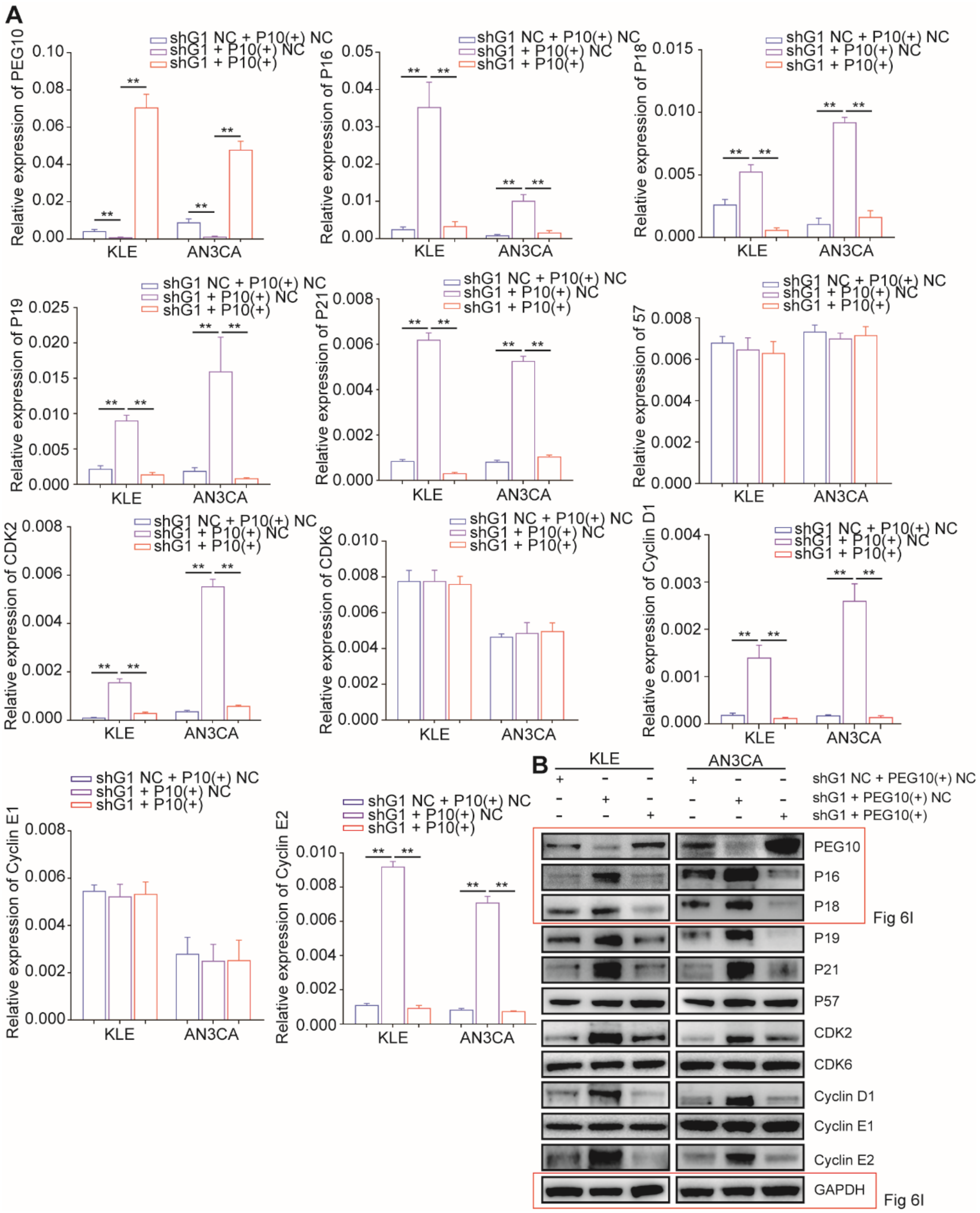

Figure S6: The IGF2BP1-PEG10 axis regulates the expression of PEG10. (a-b) q-PCR and WB confirmed the changes of genes related to cell cycle regulation after expression of PEG10 in knockdown IGF2BP1 cells.

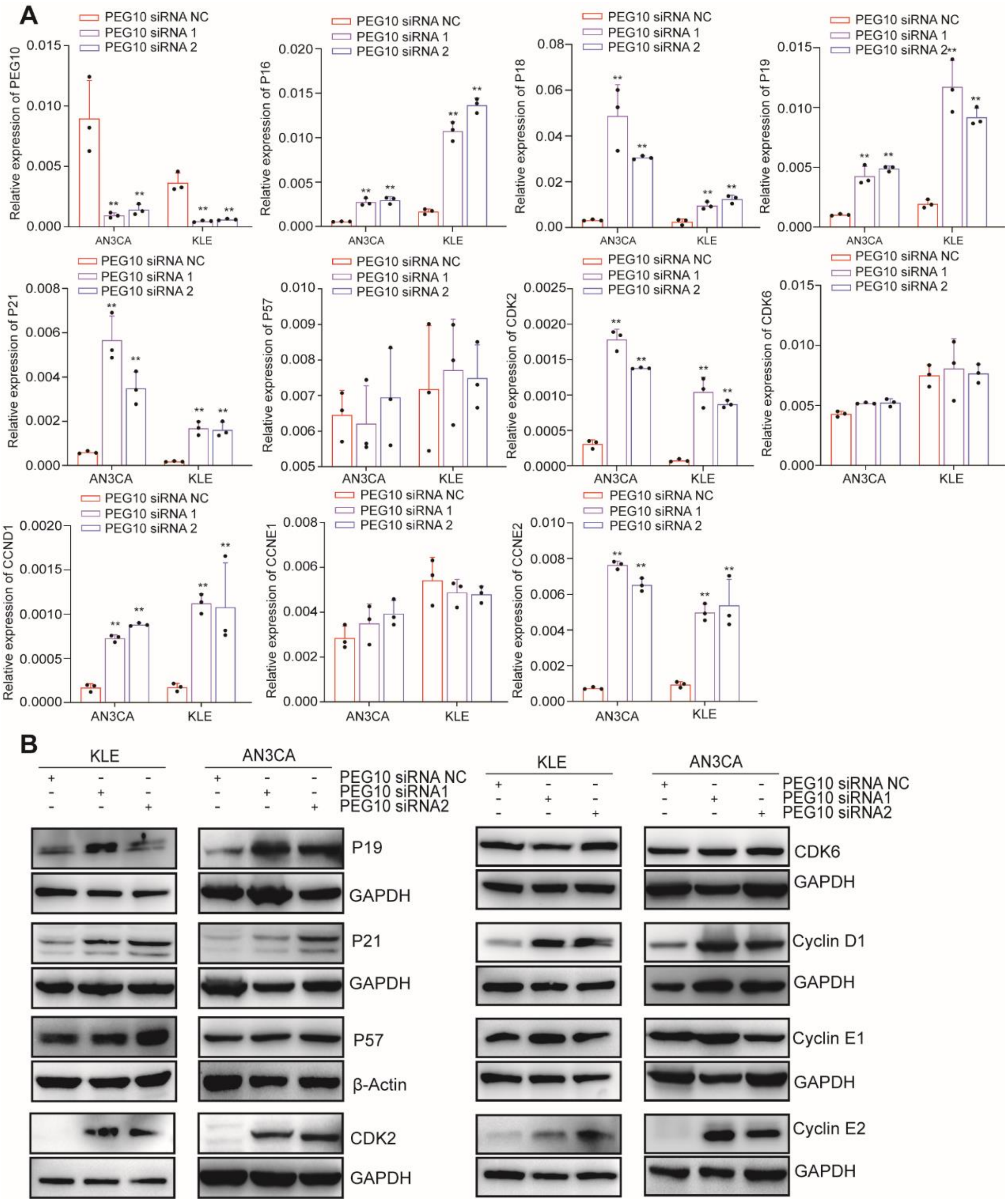

Figure S7: Silencing PEG10 promotes the expression of genes related to cell cycle regulation. (a-b) q-PCR and WB confirmed the expression changes of genes related to cell cycle regulation after knockdown PEG10.

Table S1. Primers used in this study.

P16	F: CAAGATCACGCAAAAACCTCT	R: CGACCCTATACACGTTGAACTG
P16 (for ChIP)	F: GGAAGGAGTCTTTACGAAGG	R:TAAGATGTGTTGTTTGTTCATTT
P18	F: GGGGACCTAGAGCAACTTACT	R: CAGCGCAGTCCTTCCAAAT
P18 (for ChIP)	F: GGCCGCGCTCTCTGAACT	R: TCAAAATTCCTCCGAGCCGT
P19	F: AGTCCAGTCCATGACGCAG	R: ATCAGGCACGTTGACATCAGC
P21	F: CCCAGGGAAGGGTGTCCTG	R: GGGCGGCCAGGGTATGTAC
P57	F: GCGGCGATCAAGAAGCTGTC	R: CCGGTTGCTGCTACATGAAC
CDK2	F: GTACCTCCCCTGGATGAAGAT	R: CGAAATCCGCTTGTTAGGGTC
CDK6	F: CCAGATGGCTCTAACCTCAGT	R: AACTTCCACGAAAAAGAGGCTT
CCND1	F: CAATGACCCCGCACGATTTC	R: CATGGAGGGCGGATTGGAA
CCNE1	F: ACTCAACGTGCAAGCCTCG	R: GCTCAAGAAAGTGCTGATCCC
CCNE2	F: GGAACCACAGATGAGGTCCA	R: CCATCAGTGACGTAAGCAAACT
IGF2BP1	F: GCGGCCAGTTCTTGGTCAA	R: TTGGGCACCGAATGTTCAATC
PEG10 (q-PCR)	F: AGCAGTCGGAGGAGAACAAC	R: CACTGGGCCATGAAAGGAG
PEG10 (MeRIP-PCR)	F: GCCTAGAAATGGGCCGTTGT	R: CGTTCTTGTCGTTGGTGAAC
β-actin	F: ACCGCGAGAAGATGACCCAG	R: TTAATGTCACGCACGATTTCCC
GAPDH	F: TGTGGGCATCAATGGATTTGG	R: ACACCATGTATTCCGGGTCAAT
ALKBH5 shRNA	CACCGCAUAUGCGUGUGAUUAUUUGCGAACAAAUAAUCACACGCAUAUGC (top)	
ALKBH5 shRNA	CACCGCTTCAGCTCTGAGAACTACTCGAAAGTAGTTCTCAGAGCTGAAGC (top)	
IGF2BP1 shRNA	CCGCCUUAAAGGAUGGUUCAUUUCGAAAAAUGAACCAUCCUUUAAGGC(top)	
IGF2BP1 shRNA2	CACCGGAGGCCUAUGAGAAUGAUGUCGAAACAUCAUUCUCAUAGGCCUCC(top)	
PEG10 siRNA1	GCGUGGUGAGUAUGCGAAA	
PEG10 siRNA2	GGUGAGUAUGCGAAAUAAG	
PABPC1 siRNA	Provided by thermo fisher (Cat: AM16708)	
PEG10 Luciferase reporter clone sequences (WT)	TAGCCCTGGCATGTGCACACGGACATTTGCCACCACTGCAAGCAAAAGTC	
PEG10 Luciferase reporter clone sequences (Mut)	TAGCCCTGGCATGTGCACACGGTCATTTGCCACCACTGCAAGCAAAAGTC	

P16 Luciferase reporter clone sequences (WT)	TTACGAAGGGAAAAGTCCGTGGGAGTATGGTGGCACTAAAAAAAGAAATA
P16 Luciferase reporter clone sequences (Mut)	TTACGAAGGGAAAAGTCCGTGCGAGTATGGTGGCACTAAAAAAAGAAATA
P18 Luciferase reporter clone sequences (WT)	TCTGACGCTCGCGCTCGCGGTCGTCCCGCCTCCCGCCCCGCACCCGCCGC
P18 Luciferase reporter clone sequences (Mut)	TCTGACGCTCGCGCTCGCGGTCGTCCCGGCTCCCGCCCCGCACCCGCCGC

Table S2: Antibody used in this study

Antibody	company	Catalogue number	Concentration
Anti_IGF2BP1	Proteintech	22803-1-AP	1:1000 (WB), 1:50 (IF),1:100 (IHC)
Anti_ALKBH5	Milllipore	ABE547	1:1000 (WB)
Anti_PEG10	Abcam	Ab215035	1:30 (Cut&Tag), 1:1000 (WB)
Anti_FLAG	cell signaling	14793	1:50 (IP), 1:1000 (WB)
Anti_PABPC1	Proteintech	10970-1-AP	1:1000 (WB), 1:50 (IF)
Anti_Ki-67	Proteintech	27309-1-AP	1:12000 (IHC)
Anti_β-actin	Proteintech	66009-1-Ig	1:10000 (WB)
Anti_GAPDH	Proteintech	60004-1-Ig	1:5000 (WB)
Anti_P16	ABclonal	A5025	1:1000 (WB)
Anti_P18	ABclonal	A2042	1:1000 (WB)
Anti_P19	SantaCruz	sc-56334	1:50 (WB)
Anti_P21	ABclonal	A19094	1:1000 (WB)
Anti_P57	Abcam	Ab75974	1:500 (WB)
Anti_CDK2	ABclonal	A0294	1:1000 (WB)
Anti_CDK6	ABclonal	A0705	1:1000 (WB)
Anti_CCND1	ABclonal	A19038	1:1000 (WB)
Anti_CCNE1	ABclonal	A14225	1:1000 (WB)

Anti_CCNE2	ABclonal	A4272	1:1000 (WB)

Table S3: Association between IGF2BP1 expression and clinicopathologic parameters

Clinical factors	patients	expression of IGF2BP1 (IHC score)				P-value
		(-)	(+)	(++)	(+++)	
**Patients**	204	39	63	64	38	**< 0.01**
Normal endometrium	30	26	4	0	0	
Endometrial cancer	174	13	59	64	38	
Age(Years)						ns
< 60	92	20	28	26	18	
> 60	112	19	35	38	20	
**Histology type**						**< 0.01**
Grade 1 endometrioid	75	10	45	18	2	
Grade 2 endometrioid	59	3	12	36	8	
Grade 3 endometrioid	31	0	1	7	23	
UPSC	9	0	1	3	5	
**FIGO stage**						**< 0.05**
I	89	11	41	27	10	
II	45	2	10	28	5	
III-IV	40	0	8	9	23	
Depth of invasion						ns
< 50%	70	8	27	20	15	
> 50%	104	5	32	44	23	
LVI						ns
Negative	103	8	39	36	20	
Positive	71	5	20	28	18	
Lymph node metastasis						ns
Negative	144	13	51	52	28	
Positive	30	0	8	12	10	

LVI: lymphovascular invasion

Table S4: Association between PEG10 expression and clinicopathologic parameters.

Clinical factors	patients	expression of PEG10 (IHC score)				P-value
		(-)	(+)	(++)	(+++)	
**Patients**	204	46	70	55	33	**< 0.01**
Normal endometrium	30	18	12	0	0	
Endometrial cancer	174	28	58	55	33	
Age(Years)						ns
< 60	92	25	30	20	17	
> 60	112	21	40	35	16	
Histology type						ns

Grade 1 endometrioid	75	12	27	22	14	
Grade 2 endometrioid	59	12	20	21	6	
Grade 3 endometrioid	31	3	9	10	9	
UPSC	9	1	2	2	4	
**FIGO stage**						**< 0.05**
I	89	23	39	24	3	
II	45	5	17	17	6	
III-IV	40	0	2	14	24	
**Depth of invasion**						**< 0.05**
< 50%	70	17	38	7	8	
> 50%	104	11	20	48	25	
LVI						ns
Negative	103	18	30	35	20	
Positive	71	10	28	20	13	
Lymph node metastasis						ns
Negative	144	25	48	46	25	
Positive	30	3	10	9	8	

LVI: lymphovascular invasion

Supplementary table legends

Table S5: data of Flag-IGF2BP1 RIP-Seq

Table S6: Data of mRNA-Seq.

Table S7: Data of MeRIP-Seq.

Table S8: Data of Flag-IGF2BP1 CoIP-MS.

Table S9: Data of PEG10 CUT&Tag-Seq.

Table S5: Data of Flag-IGF2BP1 RIP-Seq.

GeneID	RIP	Count	fpkm	Fold (log2)	p_value(-log10)
ENSG00000106484	MEST	8193	5196.977967	1.75295	82.73652
ENSG00000198804	MT-CO1	7511	4504.834424	5.52817	2084.29126
ENSG00000242265	PEG10	4663	1172.835844	2.7948	73.63866
ENSG00000167658	EEF2	3393	3648.80667	2.02204	46.20214
ENSG00000198938	MT-CO3	3224	3803.158411	4.26457	1075.99634
ENSG00000131711	MAP1B	2851	812.2956857	2.49197	13.64093
ENSG00000220884	MESTP1	2395	2228.356018	2.20855	191.0867
ENSG00000099194	SCD	2073	357.5509508	3.47333	56.00361
ENSG00000110321	EIF4G2	1948	1373.158161	1.47322	5.16832
ENSG00000198886	MT-ND4	1594	1069.804805	3.59262	553.87762
ENSG00000255248	MIR100HG	1357	746.1382444	1.70302	10.69824
ENSG00000167996	FTH1	1281	1528.666935	2.40722	117.20416
ENSG00000185650	ZFP36L1	1248	615.5718594	4.19669	92.18823
ENSG00000100852	ARHGAP5	1236	322.3629966	1.81072	9.14237
ENSG00000204469	PRRC2A	1120	628.1492011	4.79843	21.93465
ENSG00000072364	AFF4	1101	472.2848421	2.15761	7.41946
ENSG00000164924	YWHAZ	1098	687.9888936	1.58729	4.78956
ENSG00000187079	TEAD1	1021	329.6994125	2.66784	11.23862
ENSG00000111371	SLC38A1	1000	412.8738969	2.14427	9.71902
ENSG00000136158	SPRY2	995	381.831262	1.84051	16.75589
ENSG00000070756	PABPC1	971	933.4898553	3.11747	64.01558
ENSG00000147862	NFIB	970	382.7186021	2.7171	10.02561
ENSG00000113448	PDE4D	950	427.5404636	2.51879	10.14202
ENSG00000212802	RPL15P3	903	1357.932177	2.35124	99.34136
ENSG00000159217	IGF2BP1	900	411.2419843	3.71751	158.77702
ENSG00000167978	SRRM2	887	640.8835065	2.39524	8.35203
ENSG00000198899	MT-ATP6	865	1174.72021	7.18078	1197.92261
ENSG00000198712	MT-CO2	823	1112.779659	3.42247	275.93518
ENSG00000094916	CBX5	820	248.6448439	2.16057	5.80593
ENSG00000132510	KDM6B	819	313.6405534	1.8536	5.01241
ENSG00000141367	CLTC	764	318.4208528	2.41965	4.72862
ENSG00000102908	NFAT5	759	109.8344054	4.34654	26.35716
ENSG00000117713	ARID1A	740	203.3818043	3.61546	12.14945
ENSG00000198888	MT-ND1	719	695.5629534	3.27445	168.03987
ENSG00000087086	FTL	708	745.7687593	3.35963	93.8967
ENSG00000140836	ZFHX3	701	111.1263469	3.69245	11.00655
ENSG00000174748	RPL15	695	504.5228279	3.60375	149.6479
ENSG00000086758	HUWE1	691	155.9069853	2.25256	4.80778
ENSG00000225178	RPSAP58	673	700.9185328	1.58297	16.9271
ENSG00000004799	PDK4	673	420.835468	4.79418	41.76044
ENSG00000168003	SLC3A2	666	570.8450365	1.97901	15.98905
ENSG00000167548	KMT2D	649	201.0785784	3.57663	6.2444

ENSG00000134308	YWHAQ	645	485.3703876	2.17866	16.95424
ENSG00000151846	PABPC3	632	172.5708056	3.23092	131.80641
ENSG00000167460	TPM4	626	327.274332	1.65845	7.89335
ENSG00000140105	WARS	600	612.4751849	2.01844	10.46064
ENSG00000178343	SHISA3	598	280.5950494	2.3485	26.75506
ENSG00000136295	TTYH3	584	232.3032761	2.70218	7.28109
ENSG00000139514	SLC7A1	578	186.0619881	2.05768	5.26559
ENSG00000141867	BRD4	573	291.1713759	2.57398	12.38907
ENSG00000196230	TUBB	570	191.2068885	2.59662	58.00106
ENSG00000198763	MT-ND2	567	503.246525	4.88478	542.81793
ENSG00000117523	PRRC2C	538	124.3284834	3.61793	5.46828
ENSG00000173812	EIF1	520	364.6061525	1.76266	12.11346
ENSG00000204463	BAG6	516	358.2703942	2.74196	10.95322
ENSG00000123983	ACSL3	508	361.9549036	3.88393	28.40418
ENSG00000054598	FOXC1	504	120.1645049	2.59563	11.56771
ENSG00000095139	ARCN1	496	169.8960794	4.00294	12.42869
ENSG00000253341	AC115837.1	496	458.2611533	3.62463	54.44541
ENSG00000102225	CDK16	496	340.0440433	3.33876	27.49654
ENSG00000110395	CBL	493	99.68187623	4.9651	9.25272
ENSG00000064393	HIPK2	493	62.30457801	3.30182	7.12781
ENSG00000120738	EGR1	491	144.7084853	3.70896	24.88506
ENSG00000128283	CDC42EP1	486	443.7029015	1.91824	7.85509
ENSG00000174231	PRPF8	478	256.4224704	3.71745	9.3646
ENSG00000148730	EIF4EBP2	477	60.78923817	1.89265	5.07553
ENSG00000285976	AL135905.2	473	85.97644483	1.67761	22.03526
ENSG00000118058	KMT2A	464	128.9049605	2.93161	5.86828
ENSG00000226608	FTLP3	459	803.9780793	3.05899	111.42392
ENSG00000012660	ELOVL5	454	253.0899567	2.69634	23.46147
ENSG00000058262	SEC61A1	453	218.4313872	2.2892	10.17125
ENSG00000105887	MTPN	450	180.2411815	2.30217	11.06706
ENSG00000111676	ATN1	445	158.3504042	5.10959	19.26424
ENSG00000123066	MED13L	442	112.890966	4.16651	11.9912
ENSG00000055609	KMT2C	441	62.53501232	3.35408	9.03807
ENSG00000196182	STK40	440	174.497647	3.70908	20.85973
ENSG00000083312	TNPO1	434	242.0865426	1.88643	5.42553
ENSG00000171988	JMJD1C	431	113.8220945	2.80545	5.931
ENSG00000156639	ZFAND3	431	288.635029	3.69245	11.00655
ENSG00000168066	SF1	427	275.3874651	4.49906	19.11593
ENSG00000137309	HMGA1	426	229.4588162	2.30778	5.52475
ENSG00000115977	AAK1	424	148.4782705	4.9651	9.25272
ENSG00000125534	PPDPF	424	668.0257451	2.5277	13.81304
ENSG00000060749	QSER1	422	97.57035933	7.4	51.54924
ENSG00000135387	CAPRIN1	422	259.3232142	2.4501	11.13009
ENSG00000100644	HIF1A	422	198.8188677	3.25538	27.55095

ENSG00000145012	LPP	422	122.4604447	12.32264	27.18163
ENSG00000167552	TUBA1A	410	349.477776	2.35176	18.97977
ENSG00000090060	PAPOLA	397	292.3252381	1.8048	5.12618
ENSG00000060237	WNK1	394	115.4947659	5.42264	9.31879
ENSG00000168175	MAPK1IP1L	393	166.2676802	7.51761	116.59545
ENSG00000103994	ZNF106	393	106.7433624	2.7036	6.11664
ENSG00000187446	CHP1	386	353.1031516	3.5378	28.04435
ENSG00000250927	MESTP3	383	365.9222869	2.26652	46.30354
ENSG00000184588	PDE4B	379	244.601133	2.65972	11.18385
ENSG00000074657	ZNF532	371	199.6013516	4.11294	18.64918
ENSG00000112081	SRSF3	362	193.0745015	3.9695	41.25158
ENSG00000253352	TUG1	350	81.63761292	2.63765	8.71118
ENSG00000067225	PKM	348	224.1249722	2.42352	19.14338
ENSG00000152558	TMEM123	347	248.1969239	1.86168	4.78256
ENSG00000108256	NUFIP2	346	56.09005874	2.99937	11.63754
ENSG00000138685	FGF2	344	70.01410872	2.5376	11.8267
ENSG00000109133	TMEM33	339	130.7422529	4.52453	6.6833
ENSG00000076108	BAZ2A	338	153.3080357	3.52007	10.93404
ENSG00000154380	ENAH	337	105.6867573	5.49979	21.93163
ENSG00000181722	ZBTB20	337	116.6430566	4.88652	10.65463
ENSG00000104067	TJP1	335	91.90761562	2.37702	4.75251
ENSG00000158195	WASF2	332	90.84202949	2.94948	11.63754
ENSG00000095787	WAC	329	174.2677818	3.1934	14.83553
ENSG00000138336	TET1	329	32.75964116	2.97436	5.9866
ENSG00000161202	DVL3	326	171.7931821	6.94488	24.75457
ENSG00000086598	TMED2	324	216.5082077	1.94437	6.03376
ENSG00000182934	SRPRA	319	229.590017	4.09947	31.5096
ENSG00000136485	DCAF7	319	168.1043714	2.56849	5.38186
ENSG00000177311	ZBTB38	318	153.096478	3.65352	9.17341
ENSG00000233913	RPL10P9	318	455.9664097	4.55368	129.58667
ENSG00000132912	DCTN4	313	239.6309161	1.77281	4.81156
ENSG00000149948	HMGA2	309	125.5601039	5.86674	48.65232
ENSG00000047849	MAP4	309	109.9979971	5.12327	8.41215
ENSG00000128989	ARPP19	306	175.9952014	2.26531	5.33979
ENSG00000101126	ADNP	301	67.73141029	2.44501	6.04417
ENSG00000182481	KPNA2	297	264.1122559	2.19114	6.67569
ENSG00000168769	TET2	297	44.53254639	6.69728	31.14822
ENSG00000005339	CREBBP	296	174.0317283	4.12022	10.20195
ENSG00000115844	DLX2	296	127.1490519	2.32296	11.40715
ENSG00000055208	TAB2	296	108.7611874	3.3934	4.79185
ENSG00000109654	TRIM2	291	174.0800265	2.43618	6.89951
ENSG00000148143	ZNF462	291	123.1705817	3.35041	6.99766
ENSG00000108091	CCDC6	290	87.61936735	2.95988	8.24392
ENSG00000187605	TET3	290	56.8226448	3.75912	7.73977

ENSG00000122203	KIAA1191	290	190.4850025	3.28138	24.91142
ENSG00000205339	IPO7	289	232.6179686	2.56401	6.35161
ENSG00000125398	SOX9	287	67.55621554	4.88101	26.19258
ENSG00000146278	PNRC1	285	187.0679175	6.00744	91.10371
ENSG00000172757	CFL1	284	275.3185097	1.81953	15.54981
ENSG00000070770	CSNK2A2	279	148.4635619	2.65164	7.74107
ENSG00000132846	ZBED3	279	108.3247988	3.81076	27.77562
ENSG00000121644	DESI2	278	153.9549899	4.66234	20.57104
ENSG00000011405	PIK3C2A	277	100.6205796	3.12516	5.43154
ENSG00000131467	PSME3	277	174.2721058	1.944	4.98178
ENSG00000129422	MTUS1	275	137.7737381	4.27878	12.68071
ENSG00000100393	EP300	271	189.298316	3.03019	8.1324
ENSG00000146112	PPP1R18	271	94.0806946	2.0458	5.43924
ENSG00000130338	TULP4	271	83.10045438	4.06698	6.9063
ENSG00000075420	FNDC3B	270	111.4262083	5.01833	15.30598
ENSG00000197157	SND1	270	304.1487611	3.25359	5.31046
ENSG00000163191	S100A11	266	427.095109	7.69424	418.77039
ENSG00000136021	SCYL2	266	161.1046383	5.99636	9.40778
ENSG00000164054	SHISA5	266	176.6021412	2.34414	16.05119
ENSG00000115993	TRAK2	265	124.34396	4.16014	21.5187
ENSG00000114126	TFDP2	264	151.7446288	2.88651	4.75916
ENSG00000143442	POGZ	263	105.3409572	3.86599	13.45397
ENSG00000068654	POLR1A	259	98.77646188	4.32953	7.05133
ENSG00000049618	ARID1B	259	62.85303596	5.42264	9.31879
ENSG00000217128	FNIP1	258	54.47673117	3.44696	9.62294
ENSG00000136997	MYC	258	167.9156105	1.77727	5.64357
ENSG00000056586	RC3H2	258	82.30703088	7.88397	20.38453
ENSG00000250896	RNPS1P1	257	258.9142102	2.38541	17.36208
ENSG00000113552	GNPDA1	257	193.5531311	2.33917	8.5184
ENSG00000198911	SREBF2	255	90.60067996	3.69791	18.78286
ENSG00000005483	KMT2E	255	112.2482484	3.04387	6.41011
ENSG00000177200	CHD9	254	55.05243319	4.61557	14.0966
ENSG00000188994	ZNF292	254	84.52995049	2.70708	4.83877
ENSG00000006125	AP2B1	253	131.4516263	3.67873	22.06766
ENSG00000167232	ZNF91	251	151.8209417	2.63309	6.81026
ENSG00000170275	CRTAP	250	77.04411273	2.6475	8.65797
ENSG00000219507	FTH1P8	250	449.823701	3.63995	44.42178
ENSG00000196313	POM121	249	46.02929138	3.87948	18.97719
ENSG00000140262	TCF12	246	142.4608843	2.29074	7.26566
ENSG00000128918	ALDH1A2	245	189.4525039	3.04133	13.85682
ENSG00000183255	PTTG1IP	245	158.3404575	2.44694	9.72568
ENSG00000092421	SEMA6A	243	122.6722268	2.22486	5.30301
ENSG00000124789	NUP153	243	38.90850408	3.05059	6.81795
ENSG00000134352	IL6ST	242	84.3931682	3.15189	9.23779

ENSG00000134333	LDHA	241	200.6173218	4.47582	78.35112
ENSG00000107771	CCSER2	239	77.61101456	3.60095	12.6546
ENSG00000082701	GSK3B	239	90.14525672	3.25359	5.31046
ENSG00000152601	MBNL1	237	69.53886244	4.15298	17.53733
ENSG00000172380	GNG12	236	89.67200365	2.1192	4.93094
ENSG00000204217	BMPR2	236	34.44786251	4.56438	10.30564
ENSG00000136381	IREB2	235	120.341539	2.96797	6.56246
ENSG00000126247	CAPNS1	234	229.9808521	5.98387	18.00154
ENSG00000079432	CIC	233	79.89882992	4.29865	6.99678
ENSG00000185728	YTHDF3	233	93.6086639	2.0852	4.91801
ENSG00000185591	SP1	231	70.55398588	3.11806	8.45449
ENSG00000035403	VCL	230	59.96972983	8.71573	19.6141
ENSG00000107863	ARHGAP21	227	87.95061547	2.8813	7.14921
ENSG00000180398	MCFD2	227	116.3736802	2.77126	7.76376
ENSG00000204569	PPP1R10	224	192.5312328	2.97396	7.02967
ENSG00000108061	SHOC2	223	109.8768082	3.60134	13.75022
ENSG00000113384	GOLPH3	223	138.6945319	3.09513	14.20577
ENSG00000113328	CCNG1	222	212.9812567	2.02585	4.74966
ENSG00000005486	RHBDD2	221	193.1843988	3.37429	13.6723
ENSG00000103194	USP10	218	208.0645834	1.85554	5.3064
ENSG00000153317	ASAP1	218	103.9786392	3.2434	8.65647
ENSG00000120693	SMAD9	215	46.86308479	6.62013	12.18861
ENSG00000132466	ANKRD17	215	46.53406711	5.42264	9.31879
ENSG00000255642	PABPC1P4	214	105.55479	4.5176	55.80456
ENSG00000165997	ARL5B	213	27.37498523	3.81085	11.72706
ENSG00000088247	KHSRP	212	197.2490505	2.82088	8.92154
ENSG00000272391	POM121C	212	95.50197573	2.57332	6.92955
ENSG00000142599	RERE	210	95.01755437	3.73531	8.87152
ENSG00000006468	ETV1	210	82.92736171	2.71074	6.72949
ENSG00000235437	LINC01278	210	103.2513988	7.23309	58.35772
ENSG00000121089	NACA3P	209	298.2886475	3.45718	48.88113
ENSG00000105323	HNRNPUL1	208	115.5352589	5.03963	20.59565
ENSG00000158711	ELK4	207	59.80673806	4.25489	15.98026
ENSG00000029363	BCLAF1	207	67.86294525	3.35678	9.77814
ENSG00000127946	HIP1	206	65.2677393	3.45975	6.46191
ENSG00000111348	ARHGDIB	205	250.4513784	4.36179	75.52826
ENSG00000136240	KDELR2	205	154.6424906	2.01055	7.18951
ENSG00000162599	NFIA	204	88.86804331	3.69245	11.00655
ENSG00000273841	TAF9	204	187.9151952	3.01823	32.03793
ENSG00000125686	MED1	203	65.43813817	2.91897	6.27273
ENSG00000130175	PRKCSH	203	199.7255515	3.74959	10.35194
ENSG00000072062	PRKACA	201	126.2855593	5.12327	8.41215
ENSG00000009413	REV3L	201	36.65792612	2.9655	5.28566
ENSG00000151176	PLBD2	200	90.40445055	5.7683	14.29954

ENSG00000136451	VEZF1	200	78.40928607	3.72803	15.50603
ENSG00000168488	ATXN2L	198	98.45044665	4.74477	20.15921
ENSG00000178719	GRINA	196	183.8419429	2.48571	6.96926
ENSG00000164190	NIPBL	195	50.87258623	3.38066	8.13604
ENSG00000110851	PRDM4	194	114.1338935	2.70071	8.54503
ENSG00000181827	RFX7	194	35.62718043	3.1678	6.26799
ENSG00000138760	SCARB2	194	66.01121437	2.94854	7.60861
ENSG00000131023	LATS1	194	54.13955361	2.8545	6.29195
ENSG00000105220	GPI	193	121.0947375	2.55878	15.86857
ENSG00000172845	SP3	192	61.46376102	3.29293	15.50482
ENSG00000198315	ZKSCAN8	192	56.56859051	3.16038	5.2663
ENSG00000105993	DNAJB6	191	115.5290831	3.04761	9.28
ENSG00000062485	CS	190	183.422892	3.38912	21.96444
ENSG00000213699	SLC35F6	190	59.72778061	2.1192	4.93094
ENSG00000166454	ATMIN	187	55.91484577	2.97852	8.7635
ENSG00000179134	SAMD4B	187	103.4357763	8.10849	21.41391
ENSG00000244462	RBM12	187	53.42743835	2.66442	8.55606
ENSG00000085185	BCORL1	187	44.7579239	4.05425	11.31445
ENSG00000105438	KDELR1	186	168.3168106	5.59204	61.72049
ENSG00000106299	WASL	185	38.57834113	3.45126	11.47391
ENSG00000147548	NSD3	184	64.26363496	2.95822	9.33966
ENSG00000135870	RC3H1	181	36.96082861	3.74387	6.88384
ENSG00000136010	ALDH1L2	181	44.73425782	2.81934	5.34426
ENSG00000112335	SNX3	181	127.3939062	2.51642	22.53831
ENSG00000107341	UBE2R2	181	41.07867307	2.59064	10.99498
ENSG00000127589	TUBBP1	180	111.6504059	3.67203	65.48584
ENSG00000255717	SNHG1	176	193.315208	2.9688	32.76742
ENSG00000134970	TMED7	175	94.59179872	2.65989	9.8775
ENSG00000096070	BRPF3	175	49.34346573	4.36332	10.85701
ENSG00000198728	LDB1	174	114.6166169	6.68401	16.29781
ENSG00000168214	RBPJ	174	122.002828	2.22486	5.30301
ENSG00000163125	RPRD2	173	35.47602939	4.05849	9.09299
ENSG00000224550	AC114491.1	171	642.8748678	3.80454	65.75191
ENSG00000134910	STT3A	171	118.8183452	2.61472	5.53144
ENSG00000180008	SOCS4	171	35.43518205	3.21763	5.4874
ENSG00000120071	KANSL1	171	63.05710426	5.36629	18.8704
ENSG00000171467	ZNF318	171	46.67863562	4.1517	7.99138
ENSG00000224470	ATXN1L	170	53.84328081	4.28642	10.33359
ENSG00000128050	PAICS	170	63.78189856	2.74196	10.95322
ENSG00000171681	ATF7IP	169	74.18013404	4.5033	14.0966
ENSG00000111412	C12orf49	168	55.52991598	2.98384	4.87361
ENSG00000135018	UBQLN1	168	66.68356433	3.49293	8.13604
ENSG00000063245	EPN1	167	46.68919812	2.58139	7.05584
ENSG00000148737	TCF7L2	166	89.67466696	3.60944	7.09102

ENSG00000148484	RSU1	165	56.55974511	4.60708	38.29904
ENSG00000166987	MBD6	165	168.9902462	4.25085	11.91449
ENSG00000116127	ALMS1	165	24.13001143	4.74057	8.44714
ENSG00000159840	ZYX	165	130.7610902	2.94063	9.91508
ENSG00000188706	ZDHHC9	165	84.91830401	5.01007	7.07466
ENSG00000182463	TSHZ2	164	36.57423554	4.31977	10.07371
ENSG00000086232	EIF2AK1	164	140.5684474	3.90927	26.1753
ENSG00000204842	ATXN2	163	68.92936317	5.06463	17.06691
ENSG00000131844	MCCC2	162	100.1495185	3.53478	13.16268
ENSG00000162430	SELENON	161	44.90314903	2.02585	4.74966
ENSG00000120685	PROSER1	161	96.93935039	5.40324	16.87865
ENSG00000125648	SLC25A23	161	137.4873889	4.71434	19.28413
ENSG00000148516	ZEB1	160	61.63015605	3.2221	6.40804
ENSG00000115738	ID2	160	131.5324486	3.65368	30.3305
ENSG00000113569	NUP155	158	55.70885612	5.25209	7.4414
ENSG00000160691	SHC1	155	75.84646403	2.78886	7.55434
ENSG00000129315	CCNT1	155	48.92485223	3.60519	8.74288
ENSG00000107779	BMPR1A	154	29.9463792	2.80545	5.931
ENSG00000170689	HOXB9	154	55.18209202	2.51063	8.03104
ENSG00000141568	FOXK2	154	82.70904732	3.5261	12.05923
ENSG00000064666	CNN2	154	108.8044152	2.83351	17.80216
ENSG00000114784	EIF1B	154	180.7423598	3.43572	24.17037
ENSG00000213676	ATF6B	154	95.58723456	2.25094	5.84888
ENSG00000183283	DAZAP2	153	118.9076823	3.71745	9.3646
ENSG00000166783	MARF1	153	48.87742382	2.91552	5.16244
ENSG00000106609	TMEM248	152	109.4823243	2.93605	11.71423
ENSG00000273654	AC020904.2	151	90.97750287	2.88367	16.46737
ENSG00000132824	SERINC3	151	75.73235732	2.36882	7.19582
ENSG00000109184	DCUN1D4	151	96.84498398	3.2558	5.73058
ENSG00000237514	PTP4A1P7	150	269.8942206	5.66066	92.72927
ENSG00000116698	SMG7	150	64.19510846	8.78208	24.58018
ENSG00000172318	B3GALT1	150	27.23314279	5.01833	15.30598
ENSG00000138443	ABI2	150	54.14739632	3.37429	13.6723
ENSG00000214517	PPME1	149	71.95863804	2.10316	5.75491
ENSG00000056097	ZFR	149	66.82870604	8.41636	18.49883
ENSG00000137166	FOXP4	149	36.56163222	3.35041	6.99766
ENSG00000106511	MEOX2	149	55.12031674	3.14864	7.35925
ENSG00000204138	PHACTR4	148	58.39417846	5.84127	10.02177
ENSG00000120451	SNX19	147	81.06804817	4.13919	9.62239
ENSG00000139496	NUP58	147	82.5446975	4.74057	8.44714
ENSG00000107798	LIPA	146	94.29209445	4.70951	25.27414
ENSG00000111540	RAB5B	146	85.8399741	3.01732	11.57109
ENSG00000119547	ONECUT2	146	16.30949139	5.77656	12.51097
ENSG00000089693	MLF2	145	144.6617494	3.62949	25.88905

ENSG00000160014	CALM3	145	130.5758926	3.6061	12.92688
ENSG00000101266	CSNK2A1	145	52.22018759	3.01342	11.77457
ENSG00000198521	ZNF43	144	63.05710426	3.93959	9.73981
ENSG00000090924	PLEKHG2	144	86.53450565	2.70708	4.83877
ENSG00000163697	APBB2	144	82.20778037	4.08632	7.85212
ENSG00000013275	PSMC4	142	102.9207909	3.33321	9.22533
ENSG00000100225	FBXO7	142	65.46706338	1.97876	7.04352
ENSG00000116128	BCL9	140	52.37752997	2.80545	5.931
ENSG00000116044	NFE2L2	140	95.34407517	5.06865	31.59317
ENSG00000204619	PPP1R11	140	79.38519564	2.16827	6.1436
ENSG00000234975	FTH1P2	139	312.0204285	1.97705	15.30811
ENSG00000107862	GBF1	139	37.2291968	3.3934	4.79185
ENSG00000100889	PCK2	139	91.36632307	4.33132	8.71601
ENSG00000164244	PRRC1	139	50.83132327	6.18727	24.34908
ENSG00000171316	CHD7	139	54.4022076	3.21763	5.4874
ENSG00000011451	WIZ	138	36.2064054	6.5368	15.45243
ENSG00000126464	PRR12	138	32.72502026	8.97343	17.13777
ENSG00000218418	AL591135.1	138	21.58058489	3.31563	10.64726
ENSG00000075151	EIF4G3	137	46.82289043	3.94198	10.65083
ENSG00000198646	NCOA6	137	41.78850313	8.10803	21.98252
ENSG00000173402	DAG1	137	64.87595571	3.82972	10.00074
ENSG00000157764	BRAF	137	47.10139089	4.3945	7.79498
ENSG00000115806	GORASP2	136	110.2347975	2.70033	8.65797
ENSG00000085449	WDFY1	136	101.1076398	3.30182	7.12781
ENSG00000127947	PTPN12	136	132.119647	2.87906	11.79969
ENSG00000085719	CPNE3	136	108.2426024	3.60944	7.09102
ENSG00000116221	MRPL37	135	114.7546567	3.82547	13.87457
ENSG00000178464	RPL10P16	135	193.5706456	2.65886	63.05438
ENSG00000272886	DCP1A	135	71.38540105	4.39202	6.81489
ENSG00000109787	KLF3	135	49.88136893	5.91645	20.15932
ENSG00000170088	TMEM192	135	28.75473663	3.19148	6.82397
ENSG00000176087	SLC35A4	135	49.94122658	5.07136	22.36155
ENSG00000143157	POGK	134	28.68045103	3.99192	9.529
ENSG00000140931	CMTM3	134	107.0191959	4.25786	21.06458
ENSG00000108523	RNF167	134	131.4191187	3.88292	9.23266
ENSG00000177426	TGIF1	134	102.166718	4.44373	25.93205
ENSG00000141425	RPRD1A	134	77.60064428	2.93161	5.86828
ENSG00000189308	LIN54	134	40.04143099	4.16651	11.9912
ENSG00000112144	ICK	134	20.07910384	4.54234	8.80307
ENSG00000107651	SEC23IP	133	83.11039958	3.60095	12.6546
ENSG00000169118	CSNK1G1	133	67.47306165	3.20235	5.20503
ENSG00000145016	RUBCN	132	58.71984312	5.86321	12.68394
ENSG00000177683	THAP5	132	105.879058	4.39104	13.38262
ENSG00000214110	LDHAP4	132	122.3232003	3.59574	29.17969

ENSG00000103855	CD276	130	93.85548696	3.3044	8.09834
ENSG00000169375	SIN3A	130	65.95111289	2.88118	5.72783
ENSG00000124207	CSE1L	130	42.87763152	4.33132	8.71601
ENSG00000150712	MTMR12	130	57.63608763	3.71745	9.3646
ENSG00000105879	CBLL1	129	85.76854153	3.3934	4.79185
ENSG00000234130	AL359263.1	129	42.66954265	3.60172	11.52872
ENSG00000134779	TPGS2	128	97.51169995	3.78689	20.49831
ENSG00000226564	FTH1P20	128	216.0204448	3.97979	48.52698
ENSG00000147403	RPL10	128	103.2076754	4.30895	53.3915
ENSG00000143514	TP53BP2	127	84.01599872	2.93688	4.87191
ENSG00000175274	TP53I11	127	87.19700535	2.8157	6.91423
ENSG00000126773	PCNX4	127	39.82854059	5.99636	9.40778
ENSG00000069329	VPS35	127	65.47066121	3.2434	8.65647
ENSG00000113615	SEC24A	127	36.52188004	7.58437	30.94364
ENSG00000181472	ZBTB2	126	37.71182158	2.40176	6.18749
ENSG00000122515	ZMIZ2	126	48.67565943	6.03105	15.81479
ENSG00000049245	VAMP3	125	101.0530517	2.21844	4.95642
ENSG00000110660	SLC35F2	124	67.61783822	2.56078	6.78634
ENSG00000277258	PCGF2	124	64.06695733	2.69307	6.46897
ENSG00000137203	TFAP2A	124	79.5835209	3.89772	9.89464
ENSG00000179222	MAGED1	124	51.58787837	2.31671	5.20725
ENSG00000109084	TMEM97	123	83.27600006	3.35041	6.99766
ENSG00000173889	PHC3	123	58.57621838	5.04981	11.80429
ENSG00000112511	PHF1	123	90.13868153	2.40063	7.04633
ENSG00000135334	AKIRIN2	123	109.5905743	2.50917	6.48096
ENSG00000136802	LRRC8A	122	39.39601207	3.31607	7.68411
ENSG00000164402	8-Sep	121	55.18014844	2.70708	4.83877
ENSG00000025039	RRAGD	121	34.13829806	3.5515	6.67217
ENSG00000143569	UBAP2L	120	67.67103872	5.40566	15.15365
ENSG00000129562	DAD1	120	213.0144021	2.60484	16.71838
ENSG00000048828	FAM120A	120	43.77929132	5.99636	9.40778
ENSG00000135365	PHF21A	119	89.3308977	5.95164	18.43461
ENSG00000067182	TNFRSF1A	119	104.81492	3.84688	5.26464
ENSG00000105997	HOXA3	118	65.62286737	2.17974	5.63771
ENSG00000060339	CCAR1	117	56.15256404	5.12327	8.41215
ENSG00000233098	CCDC144NL-AS1	117	93.44213378	2.44414	6.04056
ENSG00000204576	PRR3	116	54.45743827	4.17709	19.30075
ENSG00000196247	ZNF107	116	21.91200028	4.58252	7.85357
ENSG00000087053	MTMR2	115	65.97786343	3.3584	9.31396
ENSG00000039523	RIPOR1	115	66.59756785	2.40346	4.90017
ENSG00000158985	CDC42SE2	115	74.84610546	3.06336	7.09064
ENSG00000105939	ZC3HAV1	115	25.71477656	4.00688	6.79294
ENSG00000067167	TRAM1	115	65.40978835	4.85537	14.05127

ENSG00000139793	MBNL2	114	29.36809981	7.65095	26.74579
ENSG00000108518	PFN1	113	104.4022386	1.71848	7.44054
ENSG00000125505	MBOAT7	113	73.80412486	2.70708	4.83877
ENSG00000225022	UBE2D3P1	113	235.3753171	2.2114	13.30914
ENSG00000147955	SIGMAR1	113	93.14317361	2.65815	10.61367
ENSG00000054965	FAM168A	111	44.26777306	3.60701	8.23007
ENSG00000213090	AC007256.1	111	37.12729321	4.16014	21.5187
ENSG00000130227	XPO7	111	64.64544442	4.06698	6.9063
ENSG00000155463	OXA1L	110	75.63727004	3.93	6.88384
ENSG00000171475	WIPF2	110	47.31726893	3.47871	7.60524
ENSG00000112290	WASF1	110	54.1704623	5.40373	21.57807
ENSG00000157741	UBN2	110	18.09214444	4.39202	6.81489
ENSG00000116237	ICMT	109	45.10393319	3.27513	9.77814
ENSG00000143079	CTTNBP2NL	109	44.72373145	4.54234	8.80307
ENSG00000108175	ZMIZ1	109	23.84842458	4.43991	9.30417
ENSG00000188938	FAM120AOS	109	52.88944946	5.99636	9.40778
ENSG00000182704	TSKU	108	49.37343211	5.52919	20.68931
ENSG00000111605	CPSF6	108	39.00134836	3.45126	11.47391
ENSG00000122778	KIAA1549	108	10.7043675	8.6013	15.96441
ENSG00000046653	GPM6B	108	67.71691739	3.87417	7.70332
ENSG00000180182	MED14	108	67.71691739	8.6013	15.96441
ENSG00000198464	ZNF480	107	40.58967007	3.35041	6.99766
ENSG00000157216	SSBP3	106	82.38048579	2.63653	6.92562
ENSG00000166272	WBP1L	106	40.66062965	3.31006	5.85335
ENSG00000102531	FNDC3A	106	21.76088304	5.04981	11.80429
ENSG00000124155	PIGT	106	53.83458434	3.90881	8.40735
ENSG00000113269	RNF130	106	42.66004269	2.12362	6.65219
ENSG00000185127	C6orf120	106	36.75769707	3.36639	9.86526
ENSG00000173065	FAM222B	105	71.40289747	4.28642	10.33359
ENSG00000017797	RALBP1	105	40.04451157	3.45975	6.46191
ENSG00000185800	DMWD	105	71.24573776	3.27513	9.77814
ENSG00000048405	ZNF800	105	47.4159866	3.12662	7.36388
ENSG00000137145	DENND4C	105	25.42758328	6.32076	11.20838
ENSG00000122034	GTF3A	104	95.04259193	2.08855	6.81709
ENSG00000160767	FAM189B	103	53.5459615	3.00503	6.98403
ENSG00000107611	CUBN	103	41.67028237	2.44501	6.04417
ENSG00000111206	FOXM1	103	47.89254173	2.65011	8.77722
ENSG00000128965	CHAC1	103	69.12791401	3.80336	11.12778
ENSG00000103202	NME4	103	84.37401728	3.41629	7.16949
ENSG00000068697	LAPTM4A	103	84.59881483	4.74057	8.44714
ENSG00000168906	MAT2A	103	71.24776777	2.69307	6.46897
ENSG00000082213	C5orf22	103	66.5676209	4.86719	16.03131
ENSG00000143324	XPR1	102	32.77742459	2.70708	4.83877
ENSG00000164294	GPX8	102	69.31185009	3.83178	8.52462

ENSG00000143418	CERS2	101	71.96347492	2.94391	13.43354
ENSG00000116731	PRDM2	100	35.97189923	4.67456	9.81576
ENSG00000136536	7-Mar	100	48.31962012	3.54913	7.23018
ENSG00000244509	APOBEC3C	100	47.74587141	3.07015	12.08649
ENSG00000156735	BAG4	100	48.3954751	3.99192	9.529
ENSG00000171492	LRRC8D	99	58.39216543	4.10273	12.42869
ENSG00000055483	USP36	99	59.29981567	3.0938	5.83711
ENSG00000141424	SLC39A6	99	40.56664395	5.40566	15.15365
ENSG00000048991	R3HDM1	99	52.8326113	3.45975	6.46191
ENSG00000145386	CCNA2	99	32.73468552	4.70045	8.43351
ENSG00000166340	TPP1	98	35.79544939	5.7319	21.43962
ENSG00000164329	TENT2	98	69.77219235	7.77616	15.16255
ENSG00000164638	SLC29A4	98	40.97381458	5.62423	8.40875
ENSG00000204272	NBDY	98	110.7996062	7.23491	73.79436
ENSG00000230673	PABPC1P3	97	195.444968	6.73949	89.99322
ENSG00000160352	ZNF714	96	43.56447635	4.01246	8.65803
ENSG00000010818	HIVEP2	96	14.71402101	4.50783	5.61193
ENSG00000146729	NIPSNAP2	96	75.36876299	2.69919	10.49449
ENSG00000182378	PLCXD1	96	57.16960901	3.37159	5.5509
ENSG00000142669	SH3BGRL3	95	72.3123994	1.93364	4.71371
ENSG00000116604	MEF2D	95	31.68393987	4.39104	13.38262
ENSG00000142089	IFITM3	95	137.0664045	5.95425	61.97791
ENSG00000171067	C11orf24	95	107.5392476	2.86911	9.49879
ENSG00000225674	IPO7P2	95	50.20546587	2.5765	9.73062
ENSG00000144369	FAM171B	95	25.97090312	3.60701	8.23007
ENSG00000063978	RNF4	95	83.12163223	3.00763	6.53583
ENSG00000145687	SSBP2	95	57.49971549	2.46164	6.18749
ENSG00000137509	PRCP	94	68.83193012	2.64458	8.08262
ENSG00000166831	RBPMS2	94	46.31578463	5.22665	33.40974
ENSG00000279800	BCLAF1P2	94	33.32109151	3.08652	10.37695
ENSG00000065427	KARS	94	78.95978905	5.33414	10.77257
ENSG00000196172	ZNF681	94	34.09205009	2.53231	7.18782
ENSG00000198538	ZNF28	94	49.19905362	4.00688	6.79294
ENSG00000105983	LMBR1	94	55.12665044	4.43991	9.30417
ENSG00000137075	RNF38	94	25.07491426	3.86361	9.529
ENSG00000214391	TUBAP2	93	53.32293256	3.92213	37.30744
ENSG00000166716	ZNF592	93	17.10618342	4.43991	9.30417
ENSG00000005893	LAMP2	93	37.99023419	5.99636	9.40778
ENSG00000133065	SLC41A1	92	28.74495023	4.87019	11.00531
ENSG00000174903	RAB1B	92	56.76120926	2.22456	10.02648
ENSG00000090487	SPG21	92	85.85777264	2.17203	4.93094
ENSG00000136715	SAP130	92	28.47558657	6.86444	31.26926
ENSG00000118705	RPN2	92	76.30946429	3.84245	6.17367
ENSG00000203950	RTL8A	92	101.2917294	8.88701	94.16211

ENSG00000179241	LDLRAD3	91	53.50299756	7.11241	16.08995
ENSG00000103507	BCKDK	91	56.33213866	2.70283	6.69973
ENSG00000249485	RBBP4P1	91	65.95628147	2.39982	7.90451
ENSG00000105866	SP4	91	25.16752846	5.63868	11.79668
ENSG00000084754	HADHA	89	34.80361103	2.46448	9.02166
ENSG00000166228	PCBD1	88	114.7894253	4.11723	38.79769
ENSG00000162129	CLPB	88	42.34427813	3.79245	6.60524
ENSG00000184635	ZNF93	88	81.79467595	4.49983	18.03066
ENSG00000167081	PBX3	88	55.97366064	5.99636	9.40778
ENSG00000104897	SF3A2	87	94.65984122	4.11267	13.11629
ENSG00000213096	ZNF254	87	30.41998678	3.97208	7.28745
ENSG00000126003	PLAGL2	87	14.10356968	2.8372	4.75456
ENSG00000103042	SLC38A7	86	54.4022076	3.45975	6.46191
ENSG00000171469	ZNF561	86	73.44046861	4.05849	9.09299
ENSG00000149308	NPAT	85	33.4515702	3.14047	5.71072
ENSG00000173456	RNF26	85	28.2063832	4.04879	18.00508
ENSG00000189079	ARID2	85	18.54912458	4.4493	8.3168
ENSG00000101294	HM13	85	65.40032444	6.90252	22.98327
ENSG00000156304	SCAF4	85	27.39065853	3.2684	7.54459
ENSG00000159346	ADIPOR1	84	52.91985862	3.66938	13.7787
ENSG00000122390	NAA60	84	65.28264912	5.22944	12.62006
ENSG00000183580	FBXL7	84	22.66890938	4.33132	8.71601
ENSG00000152926	ZNF117	84	17.64796739	5.39385	8.45488
ENSG00000258548	LINC00645	83	30.8403033	2.70708	4.83877
ENSG00000189067	LITAF	83	75.33024035	4.51961	13.06993
ENSG00000072736	NFATC3	83	23.22587441	3.89862	17.8589
ENSG00000010244	ZNF207	83	36.20826176	3.47871	7.60524
ENSG00000184117	NIPSNAP1	83	88.53692609	1.9849	5.10291
ENSG00000241889	AC079944.2	83	104.5797206	2.51811	12.10264
ENSG00000134590	RTL8C	83	96.9211047	3.719	16.02264
ENSG00000011114	BTBD7	82	21.2249307	4.11945	6.02411
ENSG00000109118	PHF12	82	36.81392106	4.48842	9.05212
ENSG00000233111	RAB1C	82	125.143032	2.90866	19.31668
ENSG00000177951	BET1L	81	59.50106423	2.35826	5.68436
ENSG00000158636	EMSY	81	29.79786788	7.4849	13.68189
ENSG00000265273	PGDP1	81	51.98600962	2.10045	5.61541
ENSG00000103148	NPRL3	80	43.70171431	2.55308	5.23431
ENSG00000163597	SNHG16	80	44.73216586	3.16917	16.59174
ENSG00000143319	ISG20L2	79	40.25463626	2.8372	4.75456
ENSG00000009844	VTA1	79	23.89214022	3.74308	10.6272
ENSG00000011007	ELOA	78	21.7740197	4.47343	10.80696
ENSG00000076685	NT5C2	78	63.05710426	2.62808	10.23671
ENSG00000082805	ERC1	78	22.43773788	6.43001	10.9893
ENSG00000279392	AL158801.5	78	155.1340372	2.95458	16.8614

ENSG00000156860	FBRS	78	37.82764933	6.3685	10.43636
ENSG00000084676	NCOA1	78	18.40707509	5.51879	9.06616
ENSG00000114354	TFG	78	46.87285723	3.97709	15.45485
ENSG00000126883	NUP214	78	43.90585957	4.80692	12.00287
ENSG00000196743	GM2A	77	41.96375353	5.40906	13.452
ENSG00000112320	SOBP	77	31.99123529	4.43366	11.06089
ENSG00000125354	6-Sep	77	30.32899904	2.52491	5.06283
ENSG00000151929	BAG3	76	37.80938796	6.98585	16.40858
ENSG00000198824	CHAMP1	76	44.99849694	6.85942	15.09668
ENSG00000156858	PRR14	76	60.64508388	3.30182	7.12781
ENSG00000108509	CAMTA2	76	33.67879838	1.81623	7.00486
ENSG00000154945	ANKRD40	76	37.6675521	3.07085	5.1683
ENSG00000196704	AMZ2	76	59.76841175	3.53558	13.71696
ENSG00000164168	TMEM184C	76	39.48744507	6.30717	16.93061
ENSG00000213462	ERV3-1	76	43.87493896	3.07085	5.1683
ENSG00000172954	LCLAT1	75	41.14045948	5.01891	10.14752
ENSG00000112640	PPP2R5D	75	35.44344133	5.31442	16.16375
ENSG00000179912	R3HDM2	74	29.72979025	2.50917	6.48096
ENSG00000186260	MRTFB	74	22.08389066	5.62423	8.40875
ENSG00000087263	OGFOD1	74	55.91338003	3.43285	7.96181
ENSG00000223501	VPS52	74	50.88325439	5.85791	18.62718
ENSG00000147533	GOLGA7	74	55.55030614	5.74195	32.79615
ENSG00000104472	CHRAC1	74	52.48311132	3.20461	7.53378
ENSG00000136930	PSMB7	74	102.7597255	4.73887	9.57653
ENSG00000065559	MAP2K4	73	47.88165931	2.52491	5.06283
ENSG00000136908	DPM2	73	60.11855711	2.15955	6.65219
ENSG00000168528	SERINC2	72	33.80116858	4.8239	7.53305
ENSG00000081059	TCF7	72	45.42176132	3.31607	7.68411
ENSG00000188459	WASF4P	72	44.57048333	2.3851	7.34756
ENSG00000119977	TCTN3	71	36.52028063	5.08805	13.10118
ENSG00000197111	PCBP2	71	47.10435048	3.48231	41.20639
ENSG00000132471	WBP2	71	53.16879722	4.16651	11.9912
ENSG00000073711	PPP2R3A	71	26.63832234	5.12327	8.41215
ENSG00000215114	UBXN2B	71	44.24761764	5.01891	10.14752
ENSG00000136854	STXBP1	71	27.34838175	2.80545	5.931
ENSG00000101447	FAM83D	70	26.77362574	4.6167	10.05901
ENSG00000185920	PTCH1	70	21.99749475	4.87996	6.50818
ENSG00000167535	CACNB3	69	50.8881894	2.95606	5.71192
ENSG00000140577	CRTC3	69	51.67108463	4.50783	5.61193
ENSG00000104853	CLPTM1	69	52.00797841	2.94854	7.60861
ENSG00000169564	PCBP1	69	36.46502258	3.30438	46.25417
ENSG00000138674	SEC31A	69	35.23676947	7.09715	19.23568
ENSG00000273749	CYFIP1	68	33.30982626	4.61557	14.0966
ENSG00000109686	SH3D19	68	22.50857265	8.20798	19.25553

ENSG00000037749	MFAP3	68	57.17177453	8.22917	14.8121
ENSG00000095261	PSMD5	68	64.56771251	3.87208	12.25296
ENSG00000123473	STIL	67	19.91774814	3.37159	5.5509
ENSG00000175376	EIF1AD	67	57.32110495	3.84473	8.25074
ENSG00000184384	MAML2	67	12.07877475	5.99636	9.40778
ENSG00000171792	RHNO1	67	56.58823238	3.0022	8.70635
ENSG00000003056	M6PR	67	62.15056615	2.4783	5.32932
ENSG00000100243	CYB5R3	67	32.37414548	3.11669	9.80723
ENSG00000144677	CTDSPL	67	36.64347395	5.48229	10.25345
ENSG00000148842	CNNM2	66	10.94678568	2.62808	10.23671
ENSG00000248930	AC020893.1	66	105.2401326	2.69798	13.5793
ENSG00000148337	CIZ1	66	39.20313226	5.88895	11.83284
ENSG00000157625	TAB3	66	14.18198813	5.20973	9.35571
ENSG00000154803	FLCN	65	36.74476736	3.45975	6.46191
ENSG00000198925	ATG9A	65	45.74919284	2.56035	4.8112
ENSG00000228412	AL022068.1	65	72.25293197	4.5033	14.0966
ENSG00000218586	AC006971.1	65	13.40045461	2.1192	4.93094
ENSG00000198039	ZNF273	65	30.78056293	3.37914	5.83198
ENSG00000183354	KIAA2026	65	16.74963483	4.87996	6.50818
ENSG00000172269	DPAGT1	64	42.55183456	5.28915	18.61682
ENSG00000173744	AGFG1	64	24.19852897	3.20461	7.53378
ENSG00000106290	TAF6	64	48.08253604	3.78567	6.06878
ENSG00000174718	KIAA1551	63	36.85310837	4.87996	6.50818
ENSG00000139579	NABP2	63	49.46075071	2.49492	5.90546
ENSG00000196531	NACA	63	51.19926567	2.29528	6.55834
ENSG00000138085	ATRAID	63	72.46861236	4.06698	6.9063
ENSG00000136699	SMPD4	63	28.71600017	3.84245	6.17367
ENSG00000135926	TMBIM1	63	61.07417645	3.93959	9.73981
ENSG00000196782	MAML3	62	20.88886223	4.8239	7.53305
ENSG00000112624	BICRAL	62	8.797165819	4.29151	7.66459
ENSG00000233225	AC004987.2	62	49.94767144	2.90059	8.00681
ENSG00000080802	CNOT4	61	24.54964721	4.89003	14.53762
ENSG00000258501	EIF3LP1	60	33.46818562	3.7	26.52257
ENSG00000136504	KAT7	60	32.22430415	5.99777	13.40795
ENSG00000172081	MOB3A	60	59.09504979	3.45975	6.46191
ENSG00000119042	SATB2	60	18.29550008	5.25209	7.4414
ENSG00000175470	PPP2R2D	59	20.73942008	3.2434	8.65647
ENSG00000204611	ZNF616	59	31.72407804	4.32953	7.05133
ENSG00000115207	GTF3C2	59	42.36445203	4.9651	9.25272
ENSG00000100167	3-Sep	59	23.09158452	4.50783	5.61193
ENSG00000172765	TMCC1	59	28.77922691	4.51604	8.44714
ENSG00000138802	SEC24B	59	12.66018891	5.40142	19.40708
ENSG00000151612	ZNF827	59	29.10155425	3.76356	4.75601
ENSG00000095951	HIVEP1	59	14.68391125	4.50783	5.61193

ENSG00000157450	RNF111	58	23.63021	3.43321	5.94583
ENSG00000141034	GID4	58	37.66894431	3.43321	5.94583
ENSG00000100129	EIF3L	58	44.81251186	2.19234	8.90042
ENSG00000109066	TMEM104	57	25.56534392	5.72202	10.25134
ENSG00000167553	TUBA1C	56	44.57048333	3.70357	10.4957
ENSG00000119242	CCDC92	56	32.38955699	3.50398	9.27005
ENSG00000163374	YY1AP1	55	25.66400813	3.40728	12.56717
ENSG00000143153	ATP1B1	55	29.30072817	2.70708	4.83877
ENSG00000132256	TRIM5	55	33.7531945	3.97823	7.05525
ENSG00000213250	RBMS2P1	55	41.4894487	3.75651	10.07656
ENSG00000141076	UTP4	55	46.11610526	3.97208	7.28745
ENSG00000141380	SS18	55	54.4022076	2.98384	4.87361
ENSG00000116120	FARSB	55	15.09826777	2.60587	4.94366
ENSG00000171552	BCL2L1	55	36.56798282	3.07377	6.25901
ENSG00000172936	MYD88	55	28.36924936	3.3044	8.09834
ENSG00000086589	RBM22	55	43.73694248	5.25209	7.4414
ENSG00000148110	MFSD14B	55	17.86027532	3.24419	5.44973
ENSG00000196268	ZNF493	54	24.04488521	3.56446	5.37183
ENSG00000010803	SCMH1	53	22.79832049	3.3044	8.09834
ENSG00000233328	PFN1P1	53	115.8779883	4.30771	33.61834
ENSG00000151414	NEK7	53	44.19872772	5.21955	9.72747
ENSG00000254598	CSNK2A3	53	34.61609396	1.96781	6.20825
ENSG00000211584	SLC48A1	53	49.26270256	3.35041	6.99766
ENSG00000078043	PIAS2	53	26.39547068	6.08774	13.58947
ENSG00000104833	TUBB4A	53	64.9223696	5.53469	30.44036
ENSG00000197372	ZNF675	53	37.79212725	3.02382	5.42841
ENSG00000039560	RAI14	53	38.414098	3.43321	5.94583
ENSG00000109846	CRYAB	52	62.05361486	3.87948	18.97719
ENSG00000215256	DHRS4-AS1	52	35.43961055	3.47871	7.60524
ENSG00000140157	NIPA2	52	33.14372951	4.61557	14.0966
ENSG00000006695	COX10	52	28.97081417	4.87382	7.23956
ENSG00000141030	COPS3	52	57.04810382	3.16038	5.2663
ENSG00000213015	ZNF580	52	50.41043136	5.69021	26.65576
ENSG00000124613	ZNF391	52	32.8046054	4.8239	7.53305
ENSG00000106603	COA1	52	40.85943204	6.76132	16.40858
ENSG00000143368	SF3B4	51	32.39472115	3.53121	12.59602
ENSG00000175592	FOSL1	51	39.24019467	5.39794	25.40605
ENSG00000196954	CASP4	51	39.43705183	3.97208	7.28745
ENSG00000012822	CALCOCO1	51	36.06017889	4.22809	7.54274
ENSG00000104313	EYA1	51	17.66543595	4.22809	7.54274
ENSG00000121931	LRIF1	50	19.31573787	3.75912	7.73977
ENSG00000149809	TM7SF2	50	49.56256855	5.65743	10.56715
ENSG00000167554	ZNF610	50	29.15629033	3.84473	8.25074
ENSG00000099917	MED15	50	33.33949276	5.45559	10.58385

ENSG00000163659	TIPARP	50	15.69116948	4.89172	9.11386
ENSG00000106049	HIBADH	50	38.15336341	4.05425	11.31445
ENSG00000132692	BCAN	49	28.66352873	3.05059	6.81795
ENSG00000033800	PIAS1	49	22.45641176	6.47588	15.36915
ENSG00000153815	CMIP	49	19.26744852	4.39202	6.81489
ENSG00000109099	PMP22	49	40.31765029	3.84666	13.37655
ENSG00000176809	LRRC37A3	49	22.74951753	4.50783	5.61193
ENSG00000214078	CPNE1	49	42.43168439	2.66442	8.55606
ENSG00000175054	ATR	49	23.7635233	4.05849	9.09299
ENSG00000178177	LCORL	49	9.678991655	3.3632	5.26923
ENSG00000150961	SEC24D	49	24.01538894	4.8239	7.53305
ENSG00000198225	FKBP1C	49	28.69983466	3.25991	18.67946
ENSG00000224280	AC005014.1	49	15.22749964	2.80545	5.931
ENSG00000161642	ZNF385A	48	32.21495022	5.32015	13.67571
ENSG00000205476	CCDC85C	48	13.22771198	3.60701	8.23007
ENSG00000104064	GABPB1	48	28.51136891	4.39202	6.81489
ENSG00000141664	ZCCHC2	48	18.89029847	3.84245	6.17367
ENSG00000105750	ZNF85	48	39.14656208	3.49293	8.13604
ENSG00000066322	ELOVL1	47	45.70700722	4.37682	11.59222
ENSG00000123416	TUBA1B	47	34.44323603	4.24825	90.77259
ENSG00000182117	NOP10	47	87.8128563	7.23253	77.1049
ENSG00000152518	ZFP36L2	47	11.7606504	3.57056	4.8162
ENSG00000164442	CITED2	47	25.44927627	5.15949	12.87247
ENSG00000188766	SPRED3	46	30.04415702	3.92579	5.08049
ENSG00000078081	LAMP3	46	30.56215973	8.01018	17.2107
ENSG00000197771	MCMBP	45	18.29348959	3.92579	5.08049
ENSG00000079387	SENP1	45	23.31523183	8.6013	15.96441
ENSG00000102710	SUPT20H	45	24.32360538	5.41415	10.92847
ENSG00000137764	MAP2K5	45	37.39235293	2.70708	4.83877
ENSG00000141627	DYM	45	45.73372397	5.70503	16.81004
ENSG00000197020	ZNF100	45	21.7779638	4.39202	6.81489
ENSG00000221923	ZNF880	45	47.2928282	5.22705	10.97272
ENSG00000232024	LSM12P1	45	70.77838233	2.87026	12.37331
ENSG00000179933	C14orf119	44	18.55579174	2.85252	7.67858
ENSG00000140382	HMG20A	44	28.10279785	6.35344	13.16358
ENSG00000128789	PSMG2	44	55.82009778	3.60944	7.09102
ENSG00000149679	CABLES2	44	17.84774179	4.03937	6.53309
ENSG00000226950	DANCR	44	57.80234557	4.16014	21.5187
ENSG00000170085	SIMC1	44	27.43954908	4.35616	9.81533
ENSG00000066136	NFYC	43	33.19533702	4.65724	11.25152
ENSG00000167182	SP2	43	42.30639761	3.82355	5.53074
ENSG00000125841	NRSN2	43	40.37361802	4.22769	8.82991
ENSG00000231007	CDC20P1	43	26.51200917	4.77475	23.70878
ENSG00000180628	PCGF5	42	8.449679405	4.24852	10.88985

ENSG00000003249	DBNDD1	42	22.9298561	4.69057	10.22378
ENSG00000108774	RAB5C	42	43.11118338	3.59731	20.37132
ENSG00000261373	VPS9D1-AS1	41	28.2550959	5.15505	19.02383
ENSG00000161921	CXCL16	41	28.81332728	4.03937	6.53309
ENSG00000134986	NREP	41	34.69198417	3.66388	14.90131
ENSG00000137216	TMEM63B	41	21.69241344	5.62423	8.40875
ENSG00000225968	ELFN1	41	9.994290642	5.65984	18.4972
ENSG00000248527	MTATP6P1	40	54.32232183	7.99363	125.06414
ENSG00000149260	CAPN5	40	14.58159289	3.61283	6.60524
ENSG00000070540	WIPI1	40	33.69171327	2.79434	6.0158
ENSG00000182141	ZNF708	40	18.99050368	3.61283	6.60524
ENSG00000241627	UBQLN4P1	40	20.54053368	3.12516	5.43154
ENSG00000151883	PARP8	40	22.55701291	3.01069	5.2663
ENSG00000235238	SUMO2P1	40	130.2588069	5.4656	30.44036
ENSG00000159596	TMEM69	39	22.81382899	3.00503	6.98403
ENSG00000132964	CDK8	39	19.26744852	4.76043	7.42871
ENSG00000166579	NDEL1	39	33.5834857	4.87019	11.00531
ENSG00000198816	ZNF358	39	27.57543092	3.92049	16.73981
ENSG00000152056	AP1S3	39	30.258946	5.4061	13.46123
ENSG00000145362	ANK2	39	11.07419823	4.21628	22.1705
ENSG00000107140	TESK1	39	24.3214185	5.01007	7.07466
ENSG00000163875	MEAF6	38	27.86980659	3.10482	6.98403
ENSG00000279088	AC022400.7	38	28.43351627	2.96054	15.06988
ENSG00000102699	PARP4	38	15.82342463	4.50783	5.61193
ENSG00000124399	NDUFB4P12	38	90.11237464	4.07051	24.20976
ENSG00000085231	AK6	38	37.82973747	3.01823	32.03793
ENSG00000056277	ZNF280C	38	10.64319385	7.4849	13.68189
ENSG00000177666	PNPLA2	37	25.84515754	4.56438	10.30564
ENSG00000140307	GTF2A2	37	48.74499798	3.40874	16.44339
ENSG00000165006	UBAP1	37	14.78142055	4.9651	9.25272
ENSG00000158169	FANCC	37	24.54733757	6.74063	11.49264
ENSG00000228532	AC005000.1	37	118.8159326	4.28524	23.93206
ENSG00000153071	DAB2	36	25.01438847	7.11276	12.57497
ENSG00000131459	GFPT2	36	34.71757148	4.65724	11.25152
ENSG00000242162	AC018639.1	36	75.3261336	4.23241	23.27717
ENSG00000160741	CRTC2	35	23.45602429	4.51095	9.46042
ENSG00000240809	AC026877.1	35	22.94068995	3.60264	11.00655
ENSG00000111328	CDK2AP1	34	26.53542278	2.09821	9.03724
ENSG00000121621	KIF18A	33	18.16645147	5.72202	10.25134
ENSG00000110104	CCDC86	33	18.37425555	3.94198	10.65083
ENSG00000188612	SUMO2	33	20.40082785	3.2705	17.02348
ENSG00000133740	E2F5	33	18.46318116	3.60944	7.09102
ENSG00000182195	LDOC1	33	28.18064493	6.79958	23.71396
ENSG00000168765	GSTM4	31	25.25987965	6.60315	21.16345

ENSG00000215271	HOMEZ	31	11.64972101	5.36506	8.00792
ENSG00000235043	TECRP1	31	64.42688405	3.39303	12.01866
ENSG00000164182	NDUFAF2	31	46.54214838	4.50783	5.61193
ENSG00000157107	FCHO2	31	17.66479569	2.69798	13.5793
ENSG00000235105	AL356968.2	30	22.07249473	4.55533	12.81357
ENSG00000119917	IFIT3	30	11.20562434	3.18545	4.92189
ENSG00000007372	PAX6	30	13.33259292	7.11276	12.57497
ENSG00000080561	MID2	30	16.87659725	5.62423	8.40875
ENSG00000197472	ZNF695	29	22.55701291	5.0885	9.53399
ENSG00000131462	TUBG1	29	24.58321572	6.68626	24.49225
ENSG00000206195	DUXAP8	29	14.51314304	6.35344	13.16358
ENSG00000136243	NUPL2	29	22.96257564	5.99777	13.40795
ENSG00000226783	TLK1P1	29	10.99192145	3.71745	9.3646
ENSG00000132122	SPATA6	28	15.73235165	4.55533	12.81357
ENSG00000259768	AC004943.2	28	5.348089801	3.69245	11.00655
ENSG00000112759	SLC29A1	28	21.45439173	4.50783	5.61193
ENSG00000168569	TMEM223	27	29.00187374	5.99378	19.47108
ENSG00000158483	FAM86C1	27	16.75880086	4.20818	9.79599
ENSG00000153443	UBALD1	27	21.32417873	3.73273	6.48293
ENSG00000196267	ZNF836	27	17.87445475	5.25209	7.4414
ENSG00000214160	ALG3	27	24.74788235	10.46197	22.02352
ENSG00000111907	TPD52L1	27	20.20276156	3.49293	8.13604
ENSG00000161654	LSM12	26	15.28657073	3.20461	7.53378
ENSG00000260747	AC022968.1	26	54.4022076	3.59925	14.83686
ENSG00000167461	RAB8A	26	21.4502906	5.12327	8.41215
ENSG00000233836	AC139769.1	26	19.29837541	1.58297	16.9271
ENSG00000236530	KPNA2P1	26	15.11362398	5.94793	15.16215
ENSG00000135211	TMEM60	26	23.50515714	5.95164	18.43461
ENSG00000077152	UBE2T	25	23.78697349	5.92766	12.3583
ENSG00000164543	STK17A	25	11.28401085	5.48229	10.25345
ENSG00000122140	MRPS2	25	24.10942464	3.49293	8.13604
ENSG00000117862	TXNDC12	24	20.88062154	3.87419	10.44264
ENSG00000236773	AC092809.3	24	96.08701602	3.7478	19.38061
ENSG00000177105	RHOG	24	19.47026377	3.27513	9.77814
ENSG00000111752	PHC1	24	12.76371518	2.4783	5.32932
ENSG00000258011	HMGA1P3	24	68.50648364	4.94812	23.38948
ENSG00000244026	FAM86DP	24	13.98620082	4.93539	10.36983
ENSG00000135454	B4GALNT1	23	13.20376361	4.50783	5.61193
ENSG00000228288	PCAT6	22	31.59382864	7.89078	27.25165
ENSG00000143476	DTL	22	8.3833645	5.25209	7.4414
ENSG00000213514	AL731556.1	22	5.482733937	3.04761	9.28
ENSG00000101079	NDRG3	22	8.698771117	3.45975	6.46191
ENSG00000237264	FTH1P11	22	36.92636233	4.35412	27.45618
ENSG00000212747	RTL8B	22	10.04265826	6.03105	15.81479

ENSG00000143457	GOLPH3L	21	13.15825753	3.76356	4.75601
ENSG00000143363	PRUNE1	21	10.97885139	4.9651	9.25272
ENSG00000198715	GLMP	21	18.51438333	4.35786	10.5157
ENSG00000126746	ZNF384	21	14.81433113	7.43491	18.36732
ENSG00000082146	STRADB	21	20.66126395	6.89215	17.24156
ENSG00000248049	UBA6-AS1	21	14.15567647	5.51879	9.06616
ENSG00000154122	ANKH	21	11.18755076	2.39982	7.90451
ENSG00000236081	ELFN1-AS1	21	30.48914931	5.65984	18.4972
ENSG00000183155	RABIF	19	7.23123994	4.65508	6.17547
ENSG00000119574	ZBTB45	19	10.68851159	3.76356	4.75601
ENSG00000224668	IPO8P1	18	5.354479101	2.91303	12.86091
ENSG00000175505	CLCF1	18	11.84002527	5.99636	9.40778
ENSG00000173588	CEP83	18	11.51250036	3.75651	10.07656
ENSG00000235423	AC068768.1	18	20.67959693	2.09821	9.03724
ENSG00000100564	PIGH	18	21.6195786	3.2684	7.54459
ENSG00000075399	VPS9D1	18	13.73521083	5.15505	19.02383
ENSG00000131238	PPT1	17	9.18891759	2.12362	6.65219
ENSG00000111203	ITFG2	17	12.08473328	2.65011	8.77722
ENSG00000248275	TRIM52-AS1	17	27.10730689	4.1517	7.99138
ENSG00000111640	GAPDH	16	11.74396862	1.74976	37.93309
ENSG00000129535	NRL	16	11.19319249	4.33132	8.71601
ENSG00000080603	SRCAP	16	2.606552839	2.93161	5.86828
ENSG00000268362	AC092279.1	15	7.244158192	3.97208	7.28745
ENSG00000236570	RAD23BP1	15	11.30608226	8.37736	20.22586
ENSG00000174939	ASPHD1	14	9.727817737	2.94854	7.60861
ENSG00000141337	ARSG	14	8.241709362	2.79434	6.0158
ENSG00000171425	ZNF581	14	12.010877	5.69021	26.65576
ENSG00000156973	PDE6D	14	14.4667323	6.02139	11.20838
ENSG00000203644	AC083799.1	14	14.22826968	4.51604	8.44714
ENSG00000090061	CCNK	13	7.233987894	3.60701	8.23007
ENSG00000227018	IL6STP1	13	11.78714498	7.61839	18.12652
ENSG00000127838	PNKD	13	9.572362961	3.93959	9.73981
ENSG00000131503	ANKHD1	13	4.816862131	3.71745	9.3646
ENSG00000233459	AC125238.2	12	7.70697941	5.12327	8.41215
ENSG00000236229	VEZF1P1	12	7.201849676	6.4868	17.83555
ENSG00000253886	AC026688.1	12	9.501755437	7.11276	12.57497
ENSG00000197576	HOXA4	12	7.335129114	2.60587	4.94366
ENSG00000078967	UBE2D4	12	10.43049845	3.84688	5.26464
ENSG00000271550	BNIP3P11	12	19.7123452	4.98351	8.893
ENSG00000230445	LRRC37A6P	11	4.130415274	8.75312	20.34323
ENSG00000182957	SPATA13	11	2.94193546	2.5765	9.73062
ENSG00000261971	MMP25-AS1	11	8.048427865	4.87996	6.50818
ENSG00000279738	AL022311.1	11	0.523960281	2.90254	19.88589
ENSG00000249572	AC034231.1	11	18.94453039	6.02139	11.20838

ENSG00000120509	PDZD11	11	14.16882008	6.82845	13.03533
ENSG00000131236	CAP1	10	7.624381939	2.12362	6.65219
ENSG00000251661	AC136475.1	10	10.88044152	5.95425	61.97791
ENSG00000261061	AC092718.4	10	12.91672527	4.06698	6.9063
ENSG00000111684	LPCAT3	9	7.815528415	3.3934	4.79185
ENSG00000102931	ARL2BP	9	7.692733607	4.20252	13.09687
ENSG00000277972	CISD3	9	4.828038145	2.69307	6.46897
ENSG00000270987	AL133338.2	9	11.30915457	7.20529	21.58403
ENSG00000224236	MRRFP1	9	4.969276276	4.74057	8.44714
ENSG00000247373	AC055713.1	8	0.893670761	1.94437	6.03376
ENSG00000116035	VAX2	8	5.292346376	3.10482	6.98403
ENSG00000112305	SMAP1	8	3.588118445	2.70708	4.83877
ENSG00000205578	POM121B	8	2.974949832	7.4849	13.68189
ENSG00000236810	ELOA-AS1	7	8.440498962	4.47343	10.80696
ENSG00000270872	SRGAP2D	7	6.34070784	6.16375	11.00335
ENSG00000110328	GALNT18	7	3.21443034	1.96781	6.20825
ENSG00000241749	RPSAP52	6	5.467019877	5.86674	48.65232
ENSG00000234494	SP2-AS1	6	5.186004836	3.82355	5.53074
ENSG00000229950	TFAP2A-AS1	6	4.464219771	7.20189	24.84468
ENSG00000185010	F8	6	1.88549955	4.22516	5.86499
ENSG00000230221	AL157384.1	5	1.869869651	6.74063	11.49264
ENSG00000277203	F8A1	5	2.699467394	4.22516	5.86499
ENSG00000135747	ZNF670-ZNF695	4	1.950105491	5.0885	9.53399
ENSG00000137804	NUSAP1	4	2.779376496	4.27878	12.68071
ENSG00000157045	NTAN1	4	4.276705337	9.0151	20.74566
ENSG00000102934	PLLP	4	1.744153756	4.20252	13.09687
ENSG00000285278	TFAP2A-AS2	4	1.079471875	3.66938	13.7787
ENSG00000112245	PTP4A1	4	2.319341766	1.67761	22.03526
ENSG00000205596	AC068533.1	4	8.914096667	4.51604	8.44714
ENSG00000284707	AC079781.5	4	1.231474739	4.50076	16.95153
ENSG00000258136	AC007622.2	3	2.468427569	4.58148	18.73171
ENSG00000246859	STARD4-AS1	3	0.917497549	3.66388	14.90131
ENSG00000218803	GSTM2P1	3	4.242373987	7.51824	15.25735
ENSG00000281207	SLFNL1-AS1	2	0.38534897	3.3044	8.09834
ENSG00000008516	MMP25	2	1.220907629	4.87996	6.50818
ENSG00000267009	AC007780.1	2	2.832580487	2.79434	6.0158
ENSG00000284526	AC015802.6	2	0.32714451	2.92284	14.3616
ENSG00000269386	RAB11B-AS1	2	2.917468546	7.4849	13.68189
ENSG00000258881	AC007040.2	2	0.805256882	3.10482	6.98403
ENSG00000244687	UBE2V1	2	1.40339534	2.75684	9.01715
ENSG00000285804	AC093218.1	2	0.7003692	2.49197	13.64093
ENSG00000254996	ANKHD1-EIF4EBP3	2	0.348206901	3.71745	9.3646

ENSG00000241370	RPP21	2	2.80679068	8.30562	18.30048
ENSG00000188107	EYS	2	0.54772729	5.08805	13.10118
ENSG00000112309	B3GAT2	2	0.314249925	2.70708	4.83877
ENSG00000271551	AL355297.4	2	1.480924786	5.42264	9.31879
ENSG00000285725	AC004967.2	2	0.968416261	4.50076	16.95153
ENSG00000236986	AL157938.2	2	2.37137828	4.80692	12.00287
ENSG00000229021	AL450992.1	1	0.22507606	7.69424	418.77039
ENSG00000262412	AL160291.1	1	0.922991546	8.75312	20.34323
ENSG00000170324	FRMPD2	1	0.392879154	2.37702	4.75251
ENSG00000185885	IFITM1	1	1.475019983	9.34557	18.33121
ENSG00000285827	AP001267.5	1	0.282048652	2.93161	5.86828
ENSG00000126749	EMG1	1	0.583862076	3.3934	4.79185
ENSG00000173867	AC013489.1	1	0.478199343	3.92579	5.08049
ENSG00000275807	AC145285.6	1	0.600934067	4.74477	20.15921
ENSG00000267924	AC139769.2	1	0.403682902	1.58297	16.9271
ENSG00000228925	AC016722.2	1	0.503998653	2.77126	7.76376
ENSG00000204929	AC007389.1	1	1.420641366	5.25209	7.4414
ENSG00000116039	ATP6V1B1	1	0.912968933	3.10482	6.98403
ENSG00000256458	AC080075.1	1	2.165895853	3.37429	13.6723
ENSG00000101353	MROH8	1	0.501266953	3.84245	6.17367
ENSG00000178075	GRAMD1C	1	0.604864309	2.51811	12.10264
ENSG00000270228	AC079880.1	1	0.452022253	5.51879	9.06616
ENSG00000272540	AL662797.1	1	1.196426299	2.59662	58.00106
ENSG00000196569	LAMA2	1	0.210285932	2.20855	191.0867
ENSG00000183444	OR7E38P	1	0.938921349	4.50076	16.95153
ENSG00000137101	CD72	1	0.854748179	3.55284	6.39257
ENSG00000255054	AL020996.2	0	0	2.02585	4.74966

Table S6: Data of mRNA-Seq.

Gene_ID	Gene_name	A5_NC_fpkm	A5_shRNA_fpkm	log2 FoldChange	p value
ENSG00000123243	ITIH5	5.23603577	0	13.79349392	6.99E-43
ENSG00000198930	CSAG1	60.19094693	0	13.5728226	2.91E-41
ENSG00000135457	TFCP2	16.70544543	0	13.53283891	5.76E-41
ENSG00000130881	LRP3	12.53973586	0	13.53189574	5.86E-41
ENSG00000162344	FGF19	26.00704598	0	13.52622376	6.47E-41
ENSG00000213401	MAGEA12	30.00036223	0	13.39958416	5.50E-40
ENSG00000163817	SLC6A20	7.51396769	0	13.17755864	2.28E-38
ENSG00000176720	BOK	17.20124052	0	13.07768944	1.23E-37
ENSG00000139629	GALNT6	6.295970327	0	13.06600899	1.48E-37
ENSG00000159228	CBR1	18.28326662	0	13.01966417	3.22E-37
ENSG00000137507	LRRC32	9.867701199	0	12.99386783	5.02E-37
ENSG00000120457	KCNJ5	7.195661188	0	12.99249727	5.14E-37
ENSG00000141750	STAC2	12.92589791	0	12.97594814	6.69E-37
ENSG00000011677	GABRA3	8.789593983	0	12.73824725	3.51E-35
ENSG00000054983	GALC	5.093845426	0	12.65406481	1.40E-34
ENSG00000003400	CASP10	4.399626297	0	12.59749023	3.58E-34
ENSG00000118495	PLAGL1	3.602936496	0	12.51015463	1.54E-33
ENSG00000164742	ADCY1	2.259899487	0	12.49861499	1.87E-33
ENSG00000197937	ZNF347	3.313182165	0	12.45350437	3.90E-33
ENSG00000274173	AL035661.1	26.18895821	0	12.4394903	4.95E-33
ENSG00000102935	ZNF423	3.065288152	0	12.32000563	3.50E-32
ENSG00000226057	PHF2P2	6.93784209	0	12.31343427	3.91E-32
ENSG00000172478	MAB21L4	9.34595219	0	12.22982603	1.55E-31
ENSG00000184860	SDR42E1	2.09786748	0	12.17771435	3.58E-31
ENSG00000178538	CA8	4.291986469	0	12.16803389	4.21E-31
ENSG00000088882	CPXM1	10.17111348	0	12.13859658	6.63E-31
ENSG00000145284	SCD5	5.656418194	0	12.13611614	6.91E-31
ENSG00000233836	AC139769.1	7.320831595	0	12.10347624	1.19E-30
ENSG00000142700	DMRTA2	7.359422025	0	12.10347624	1.19E-30
ENSG00000154930	ACSS1	2.711655072	0	12.0932828	1.42E-30
ENSG00000163053	SLC16A14	4.731065998	0	12.08301682	1.68E-30
ENSG00000170315	UBB	131.878079	0.027614511	12.06143071	1.80E-52
ENSG00000164403	SHROOM1	5.105009821	0	12.03056172	3.88E-30
ENSG00000275896	PRSS2	17.62356978	0	12.00632002	5.82E-30
ENSG00000049323	LTBP1	2.981824362	0	11.98442446	8.01E-30
ENSG00000258998	LINC02302	7.194464662	0	11.91955799	2.36E-29
ENSG00000204291	COL15A1	3.108943052	0	11.90506218	3.00E-29
ENSG00000147180	ZNF711	3.732633191	0	11.75794888	3.10E-28
ENSG00000166770	ZNF667-AS1	5.054784481	0	11.73190258	4.76E-28
ENSG00000198453	ZNF568	1.939390424	0	11.72861348	5.03E-28
ENSG00000104689	TNFRSF10A	6.187814972	0	11.72531686	5.31E-28
ENSG00000184867	ARMCX2	5.064550315	0	11.71870094	5.92E-28

ENSG00000196172	ZNF681	2.797702183	0	11.71870094	5.92E-28
ENSG00000113209	PCDHB5	5.309008268	0	11.71205455	6.60E-28
ENSG00000186891	TNFRSF18	13.17614214	0	11.70871983	6.97E-28
ENSG00000204983	PRSS1	9.421772746	0	11.67835535	1.09E-27
ENSG00000135097	MSI1	5.273891843	0	11.56120906	6.96E-27
ENSG00000170954	ZNF415	4.278034244	0	11.5500722	8.35E-27
ENSG00000263711	AC079062.1	2.255049122	0	11.48528601	2.40E-26
ENSG00000170231	FABP6	13.17462236	0	11.47354492	2.72E-26
ENSG00000175785	PRIMA1	3.907991798	0	11.46170749	3.29E-26
ENSG00000185869	ZNF829	1.964596238	0	11.46170749	3.29E-26
ENSG00000169255	B3GALNT1	3.426347026	0	11.37600893	1.31E-25
ENSG00000016402	IL20RA	2.985937073	0	11.35483163	1.85E-25
ENSG00000130038	CRACR2A	1.119552427	0	11.33766308	2.27E-25
ENSG00000130701	RBBP8NL	4.952017258	0	11.32465125	2.80E-25
ENSG00000130508	PXDN	1.202672652	0	11.293826	4.59E-25
ENSG00000165474	GJB2	5.169841708	0	11.25320043	8.79E-25
ENSG00000070190	DAPP1	2.884923381	0	11.24861504	9.46E-25
ENSG00000125850	OVOL2	5.917204061	0	11.22079238	1.48E-24
ENSG00000160233	LRRC3	2.185744315	0	11.21610266	1.59E-24
ENSG00000102409	BEX4	9.502414254	0	11.19242259	2.15E-24
ENSG00000188171	ZNF626	1.798896957	0	11.12897127	5.87E-24
ENSG00000170516	COX7B2	14.70516483	0	11.10380405	8.74E-24
ENSG00000164438	TLX3	7.948471544	0	11.10380405	8.74E-24
ENSG00000046774	MAGEC2	5.876406937	0	11.08334931	1.21E-23
ENSG00000197928	ZNF677	1.251596728	0	11.08334931	1.21E-23
ENSG00000265843	LINC01029	5.327944294	0	11.0147946	3.26E-23
ENSG00000104313	EYA1	1.131375021	0	10.94848491	9.18E-23
ENSG00000256229	ZNF486	2.500654763	0	10.94848491	9.18E-23
ENSG00000111981	ULBP1	3.364646417	0	10.93713082	1.10E-22
ENSG00000079215	SLC1A3	15.60701593	0.007144952	10.93296616	4.42E-44
ENSG00000197647	ZNF433	3.365635527	0	10.90834839	1.71E-22
ENSG00000144908	ALDH1L1	25.69537054	0.013058087	10.90396994	6.55E-55
ENSG00000157765	SLC34A2	83.18990434	0.044363847	10.84291539	5.04E-56
ENSG00000087510	TFAP2C	3.253842089	0	10.82456171	5.71E-22
ENSG00000170006	TMEM154	0.843859107	0	10.82456171	5.71E-22
ENSG00000269067	ZNF728	4.422547708	0	10.81218484	6.90E-22
ENSG00000213967	ZNF726	1.521524602	0	10.79341815	9.21E-22
ENSG00000170801	HTRA3	26.48550854	0.013478808	10.78028675	5.99E-43
ENSG00000181143	MUC16	0.21545772	0	10.77440411	1.23E-21
ENSG00000198046	ZNF667	1.062422358	0	10.76158743	1.50E-21
ENSG00000124939	SCGB2A1	17.80493295	0	10.74865586	1.83E-21
ENSG00000115138	POMC	5.977112592	0	10.73560733	2.23E-21
ENSG00000080007	DDX43	3.245182852	0	10.7224397	2.73E-21

ENSG00000176845	METRNL	32.0630934	0.017046124	10.71725031	1.75E-42
ENSG00000150510	FAM124A	1.352945809	0	10.71581054	3.02E-21
ENSG00000197360	ZNF98	2.755224192	0	10.71581054	3.02E-21
ENSG00000018869	ZNF582	2.669279541	0	10.69573832	4.10E-21
ENSG00000121068	TBX2	1.476248302	0	10.68898504	4.54E-21
ENSG00000196263	ZNF471	1.299827526	0	10.67538289	5.03E-21
ENSG00000268119	AC010615.2	1.680837215	0	10.64778771	7.64E-21
ENSG00000184012	TMPRSS2	24.68053516	0.014635646	10.63960576	1.32E-47
ENSG00000083307	GRHL2	13.14931594	0.007386654	10.63778055	6.68E-42
ENSG00000259020	AL049872.1	26.60253853	0	10.63378962	9.43E-21
ENSG00000184735	DDX53	2.360434667	0	10.63378962	9.43E-21
ENSG00000251247	ZNF345	1.737385774	0	10.57639822	2.24E-20
ENSG00000128815	WDFY4	0.595713035	0	10.5616864	2.79E-20
ENSG00000198947	DMD	0.428682044	0	10.55427385	3.11E-20
ENSG00000268902	CSAG2	7.330780835	0	10.52423682	4.88E-20
ENSG00000196653	ZNF502	2.425675418	0	10.52423682	4.88E-20
ENSG00000139219	COL2A1	1.185091566	0	10.51662881	5.47E-20
ENSG00000175984	DENND2C	1.048441096	0	10.49356109	7.71E-20
ENSG00000226278	PSPHP1	29.4579423	0	10.47797511	9.72E-20
ENSG00000261183	SPINT1-AS1	6.104250676	0	10.4542758	1.38E-19
ENSG00000131771	PPP1R1B	179.3177567	0.127782298	10.44582642	3.28E-58
ENSG00000188511	C22orf34	1.074112636	0	10.39741478	2.83E-19
ENSG00000197134	ZNF257	1.404203853	0	10.35538237	5.24E-19
ENSG00000229676	ZNF492	1.663543269	0	10.35538237	5.24E-19
ENSG00000134463	ECHDC3	3.212794383	0	10.34682691	5.94E-19
ENSG00000196109	ZNF676	2.273301725	0	10.34682691	5.94E-19
ENSG00000205212	CCDC144NL	2.018738308	0	10.32956227	7.64E-19
ENSG00000276644	DACH1	1.287137656	0	10.32956227	7.64E-19
ENSG00000272259	LINC01749	1.762292893	0	10.32956227	7.64E-19
ENSG00000229689	AC009237.3	1.385013936	0	10.32085184	8.67E-19
ENSG00000163220	S100A9	11.94904571	0	10.32085184	8.67E-19
ENSG00000177432	NAP1L5	3.578492911	0	10.31208851	9.85E-19
ENSG00000165152	TMEM246	1.230749627	0	10.31208851	9.85E-19
ENSG00000221867	MAGEA3	98.48092254	0.075105909	10.3043052	2.71E-48
ENSG00000130224	LRCH2	1.373350177	0	10.29440052	1.27E-18
ENSG00000100558	PLEK2	4.222859029	0	10.29440052	1.27E-18
ENSG00000113494	PRLR	0.516420737	0	10.29440052	1.27E-18
ENSG00000187372	PCDHB13	1.321018005	0	10.27649297	1.65E-18
ENSG00000174599	TRAM1L1	3.263520128	0	10.25836035	2.14E-18
ENSG00000227121	AC073174.1	15.8079898	0	10.24920785	2.45E-18
ENSG00000122012	SV2C	0.541946318	0	10.23999692	2.80E-18
ENSG00000151743	AMN1	2.014071805	0	10.22139673	3.65E-18
ENSG00000186998	EMID1	1.31352509	0	10.22139673	3.65E-18

ENSG00000251629	LINC02241	3.341100424	0	10.22139673	3.65E-18
ENSG00000010704	HFE	1.119187404	0	10.20255361	4.79E-18
ENSG00000267795	SMIM22	3.53350293	0	10.17381927	6.30E-18
ENSG00000246082	NUDT16P1	2.674847194	0	10.16411255	7.23E-18
ENSG00000145103	ILDR1	2.043400287	0	10.15434008	8.31E-18
ENSG00000150556	LYPD6B	1.628868009	0	10.15434008	8.31E-18
ENSG00000197172	MAGEA6	84.80450312	0.072082322	10.1478878	3.96E-47
ENSG00000132746	ALDH3B2	1.872918195	0	10.13459429	1.10E-17
ENSG00000007264	MATK	13.3600427	0.010809737	10.11142183	4.85E-38
ENSG00000106031	HOXA13	1.166640583	0	10.10445943	1.68E-17
ENSG00000248663	LINC00992	5.186655601	0	10.10445943	1.68E-17
ENSG00000175745	NR2F1	1.356041517	0	10.08401405	2.24E-17
ENSG00000006042	TMEM98	1.119756633	0	10.07368167	2.59E-17
ENSG00000185477	GPRIN3	6.515837142	0.005887948	10.03193118	4.23E-43
ENSG00000168143	FAM83B	1.781192249	0	10.03159586	4.67E-17
ENSG00000159556	ISL2	2.521049258	0	10.02087974	5.43E-17
ENSG00000005001	PRSS22	2.345727022	0	10.02087974	5.43E-17
ENSG00000183087	GAS6	53.43890231	0.052171471	9.980290746	9.27E-52
ENSG00000134762	DSC3	0.769856661	0	9.977201088	9.96E-17
ENSG00000225899	FRG2B	2.570492679	0	9.966071626	1.16E-16
ENSG00000233757	AC092835.1	2.321413931	0	9.954855639	1.36E-16
ENSG00000226321	CROCC2	0.894703529	0	9.954855639	1.36E-16
ENSG00000147246	HTR2C	1.095983529	0	9.943551772	1.59E-16
ENSG00000171517	LPAR3	1.474225612	0	9.920674811	2.17E-16
ENSG00000187566	NHLRC1	2.323479246	0	9.920674811	2.17E-16
ENSG00000184785	SMIM10	3.400508688	0	9.920674811	2.17E-16
ENSG00000183844	FAM3B	1.439676547	0	9.909098841	2.55E-16
ENSG00000130294	KIF1A	18.02318244	0.018768605	9.887195479	4.59E-51
ENSG00000160179	ABCG1	0.832841855	0	9.885664467	3.51E-16
ENSG00000197506	SLC28A3	1.006878332	0	9.885664467	3.51E-16
ENSG00000280202	AC005831.1	2.979398669	0	9.87380297	4.13E-16
ENSG00000124785	NRN1	2.117991061	0	9.837621875	6.77E-16
ENSG00000174469	CNTNAP2	0.293345598	0	9.825357022	6.77E-16
ENSG00000177465	ACOT4	2.052122052	0	9.812987006	7.99E-16
ENSG00000014257	ACPP	1.146035497	0	9.800510008	9.45E-16
ENSG00000174080	CTSF	1.659297941	0	9.800510008	9.45E-16
ENSG00000177519	RPRM	3.266707571	0	9.800510008	9.45E-16
ENSG00000155066	PROM2	7.305389709	0.007382999	9.790618006	1.06E-35
ENSG00000128714	HOXD13	2.126580976	0	9.787924162	1.12E-15
ENSG00000184454	NCMAP	1.155638376	0	9.787924162	1.12E-15
ENSG00000196267	ZNF836	1.122417857	0	9.787924162	1.12E-15
ENSG00000101443	WFDC2	456.4954671	0.518241644	9.776469056	3.39E-54
ENSG00000119782	FKBP1B	2.790680074	0	9.762418213	1.57E-15

ENSG00000268940	CT45A1	32.67149206	0.033783488	9.757581815	1.83E-35
ENSG00000138769	CDKL2	1.226708552	0	9.749494122	1.87E-15
ENSG00000141579	ZNF750	1.249174531	0	9.749494122	1.87E-15
ENSG00000269586	CT45A10	79.27674452	0.088464668	9.727560275	7.51E-41
ENSG00000166402	TUB	0.677568303	0	9.723293333	2.65E-15
ENSG00000237187	NR2F1-AS1	0.584563301	0	9.710012313	3.16E-15
ENSG00000167355	OR51B5	1.127627549	0	9.696607896	3.77E-15
ENSG00000088386	SLC15A1	1.066565656	0	9.696607896	3.77E-15
ENSG00000168907	PLA2G4F	0.725750906	0	9.683077766	4.50E-15
ENSG00000181433	SAGE1	13.79641534	0.015158524	9.670043182	7.84E-35
ENSG00000162373	BEND5	2.013050247	0	9.641708954	7.75E-15
ENSG00000183305	MAGEA2B	1.777003067	0	9.641708954	7.75E-15
ENSG00000102109	PCSK1N	3.157667959	0	9.641708954	7.75E-15
ENSG00000267640	AC016582.3	0.610181242	0	9.627651476	9.32E-15
ENSG00000118402	ELOVL4	1.401090065	0	9.627651476	9.32E-15
ENSG00000141744	PNMT	2.896589243	0	9.613455673	1.12E-14
ENSG00000260220	CCDC187	0.359352002	0	9.599118798	1.35E-14
ENSG00000260922	AC009139.1	2.414920972	0	9.584638017	1.63E-14
ENSG00000176490	DIRAS1	1.175028457	0	9.570010414	1.97E-14
ENSG00000224807	DUX4L9	3.252560901	0	9.570010414	1.97E-14
ENSG00000262179	MYMX	4.85762061	0	9.570010414	1.97E-14
ENSG00000064655	EYA2	1.120284335	0	9.55523298	2.38E-14
ENSG00000206069	TMEM211	3.609250867	0	9.55523298	2.38E-14
ENSG00000148798	INA	11.36781529	0.013854262	9.520524989	9.41E-34
ENSG00000178882	RFLNA	1.237892333	0	9.509970189	4.26E-14
ENSG00000176438	SYNE3	0.216242698	0	9.509970189	4.26E-14
ENSG00000153064	BANK1	5.889681048	0.007285664	9.499032029	1.36E-33
ENSG00000035664	DAPK2	0.285486844	0	9.494561425	5.19E-14
ENSG00000085552	IGSF9	0.586307622	0	9.494561425	5.19E-14
ENSG00000272913	AC009237.14	2.411707321	0	9.478986309	6.32E-14
ENSG00000198788	MUC2	0.300187576	0	9.463241209	7.72E-14
ENSG00000154856	APCDD1	0.632793298	0	9.447322375	9.45E-14
ENSG00000185156	MFSD6L	1.718779967	0	9.447322375	9.45E-14
ENSG00000137561	TTPA	1.574169346	0	9.447322375	9.45E-14
ENSG00000155966	AFF2	0.265522285	0	9.431225929	1.16E-13
ENSG00000214814	FER1L6	0.614593475	0	9.431225929	1.16E-13
ENSG00000188227	ZNF793	2.850194401	0.003728085	9.418540797	5.07E-33
ENSG00000248498	ASNSP1	1.297043974	0	9.414947863	1.42E-13
ENSG00000136014	USP44	0.682718096	0	9.414947863	1.42E-13
ENSG00000113248	PCDHB15	0.904987358	0	9.398484033	1.75E-13
ENSG00000135074	ADAM19	0.466452332	0	9.381830149	2.15E-13
ENSG00000278384	AL354822.1	1.187165102	0	9.381830149	2.15E-13
ENSG00000165556	CDX2	0.871797371	0	9.381830149	2.15E-13

ENSG00000166341	DCHS1	0.333817814	0	9.381830149	2.15E-13
ENSG00000224141	MIR548XHG	2.471491585	0	9.381830149	2.15E-13
ENSG00000229807	XIST	1.318199978	0.001771815	9.379263346	9.61E-33
ENSG00000159885	ZNF222	1.773221824	0	9.364981771	2.15E-13
ENSG00000165025	SYK	17.41996143	0.02577531	9.348180543	3.34E-41
ENSG00000133433	GSTT2B	3.193792415	0	9.347934305	2.65E-13
ENSG00000133477	FAM83F	1.981041872	0.002731291	9.342604235	1.80E-32
ENSG00000115339	GALNT3	6.928573461	0.009552523	9.342604235	1.80E-32
ENSG00000169851	PCDH7	71.23551169	0.109632922	9.342535352	2.23E-53
ENSG00000240771	ARHGEF25	0.990063492	0	9.330682987	3.28E-13
ENSG00000169752	NRG4	0.681058828	0	9.313222884	4.06E-13
ENSG00000180720	CHRM4	2.239987764	0	9.29554888	5.04E-13
ENSG00000120471	TP53AIP1	0.982473588	0	9.29554888	5.04E-13
ENSG00000230061	TRPM2-AS	1.476710956	0	9.29554888	5.04E-13
ENSG00000229375	USP24P1	4.344828641	0	9.29554888	5.04E-13
ENSG00000183850	ZNF730	1.02844774	0	9.29554888	5.04E-13
ENSG00000228623	ZNF883	0.601603539	0	9.29554888	5.04E-13
ENSG00000099284	H2AFY2	15.80671849	0.022607637	9.289664315	4.28E-32
ENSG00000183317	EPHA10	0.410063305	0	9.27765567	6.26E-13
ENSG00000281162	LINC01127	0.870303583	0	9.27765567	6.26E-13
ENSG00000238113	LINC01410	1.100341034	0	9.27765567	6.26E-13
ENSG00000099994	SUSD2	63.13331223	0.10175364	9.267295498	5.62E-49
ENSG00000104413	ESRP1	23.21128219	0.036728715	9.265369502	1.05E-42
ENSG00000196458	ZNF605	5.377599123	0.008271087	9.264685919	1.87E-37
ENSG00000260581	AC011374.1	2.106605367	0	9.259537746	7.79E-13
ENSG00000196814	MVB12B	3.842523059	0.005677717	9.242687822	9.19E-32
ENSG00000241399	CD302	0.799819671	0	9.241189394	9.72E-13
ENSG00000250682	LINC00491	2.656287823	0	9.241189394	9.72E-13
ENSG00000188001	TPRG1	0.341821202	0	9.241189394	9.72E-13
ENSG00000258927	AL133467.2	1.877176026	0	9.222604676	1.21E-12
ENSG00000237289	CKMT1B	0.927227582	0	9.222604676	1.21E-12
ENSG00000056998	GYG2	8.15130681	0.012247092	9.218613226	1.38E-31
ENSG00000157554	ERG	0.418515321	0	9.203777423	1.52E-12
ENSG00000127928	GNGT1	1.345063218	0	9.203777423	1.52E-12
ENSG00000082556	OPRK1	0.553449679	0	9.203777423	1.52E-12
ENSG00000132698	RAB25	32.43967602	0.052693493	9.185942727	6.93E-37
ENSG00000120318	ARAP3	4.708124665	0.007236198	9.185875801	2.32E-31
ENSG00000071909	MYO3B	0.380513454	0	9.184701221	1.91E-12
ENSG00000186026	ZNF284	0.720273226	0	9.184701221	1.91E-12
ENSG00000204516	MICB	1.090185098	0	9.14577501	3.03E-12
ENSG00000137491	SLCO2B1	5.151022623	0.008649043	9.138131249	1.56E-36
ENSG00000129675	ARHGEF6	0.467602184	0	9.125910826	3.82E-12
ENSG00000187808	SOWAHD	1.938500293	0	9.125910826	3.82E-12

ENSG00000176293	ZNF135	0.468767911	0	9.125910826	3.82E-12
ENSG00000221994	ZNF630	0.818431027	0	9.125910826	3.82E-12
ENSG00000164237	CMBL	8.284179317	0.013466643	9.105011016	8.79E-31
ENSG00000112276	BVES	0.503612526	0	9.085342619	6.14E-12
ENSG00000175287	PHYHD1	1.003424203	0	9.085342619	6.14E-12
ENSG00000272077	AC124045.1	1.476290887	0	9.06462255	7.81E-12
ENSG00000250790	AC127070.2	0.883602851	0	9.06462255	7.81E-12
ENSG00000203907	OOEP	1.96536885	0	9.06462255	7.81E-12
ENSG00000142185	TRPM2	3.969773622	0.006676081	9.056035683	1.93E-30
ENSG00000179455	MKRN3	0.488164813	0	9.022267709	1.27E-11
ENSG00000125878	TCF15	1.800402058	0	9.022267709	1.27E-11
ENSG00000143416	SELENBP1	7.417394512	0.012774864	9.021663941	3.45E-30
ENSG00000189057	FAM111B	0.713911403	0	9.000614677	1.63E-11
ENSG00000186188	FFAR4	0.679270874	0	9.000614677	1.63E-11
ENSG00000243955	GSTA1	2.706417812	0	9.000614677	1.63E-11
ENSG00000254101	LINC02055	0.187850947	0	9.000614677	1.63E-11
ENSG00000092850	TEKT2	1.394256699	0	9.000614677	1.63E-11
ENSG00000253930	TNFRSF10A-AS1	4.32941876	0	9.000614677	1.63E-11
ENSG00000115226	FNDC4	1.370360393	0	8.978631702	2.09E-11
ENSG00000267280	TBX2-AS1	2.252117993	0	8.978631702	2.09E-11
ENSG00000099864	PALM	60.14108072	0.118565726	8.977748559	3.16E-47
ENSG00000126838	PZP	4.04635086	0.007188553	8.976916796	7.02E-30
ENSG00000262714	AC007342.5	1.248491526	0	8.956308572	2.69E-11
ENSG00000101463	SYNDIG1	0.939328714	0	8.956308572	2.69E-11
ENSG00000250874	AC010595.1	1.811757328	0	8.933634597	3.48E-11
ENSG00000280241	AC079298.3	1.913142004	0	8.933634597	3.48E-11
ENSG00000231358	AL355516.1	2.430732593	0	8.933634597	3.48E-11
ENSG00000198598	MMP17	0.657627629	0	8.933634597	3.48E-11
ENSG00000140092	FBLN5	0.520952734	0	8.91059857	4.50E-11
ENSG00000213468	FIRRE	0.279712583	0	8.91059857	4.50E-11
ENSG00000115041	KCNIP3	0.329186524	0	8.91059857	4.50E-11
ENSG00000163032	VSNL1	6.268362689	0.011678352	8.908335788	2.13E-29
ENSG00000242599	CSAG4	3.376043041	0	8.887188742	5.84E-11
ENSG00000171631	P2RY6	0.641074571	0	8.887188742	5.84E-11
ENSG00000174740	PABPC5	0.738815216	0	8.887188742	5.84E-11
ENSG00000073756	PTGS2	0.483756405	0	8.887188742	5.84E-11
ENSG00000151917	BEND6	0.448662678	0	8.863392782	7.60E-11
ENSG00000116014	KISS1R	1.492337527	0	8.863392782	7.60E-11
ENSG00000142319	SLC6A3	0.562010098	0	8.863392782	7.60E-11
ENSG00000259129	LINC00648	0.810168122	0	8.839197737	9.92E-11
ENSG00000062524	LTK	0.706401603	0	8.839197737	9.92E-11
ENSG00000109193	SULT1E1	1.179025153	0	8.839197737	9.92E-11

ENSG00000146833	TRIM4	4.639030824	0.009152141	8.825745115	8.09E-29
ENSG00000106123	EPHB6	0.455127914	0	8.814589992	1.30E-10
ENSG00000173930	SLCO4C1	0.454359982	0	8.814589992	1.30E-10
ENSG00000133710	SPINK5	0.55319702	0	8.814589992	1.30E-10
ENSG00000264956	AC138761.3	0.815674895	0	8.789555224	1.70E-10
ENSG00000128833	MYO5C	11.91374126	0.026737554	8.775384779	7.96E-42
ENSG00000157021	FAM92A1P1	2.629196902	0	8.76407835	2.24E-10
ENSG00000075388	FGF4	0.723781393	0	8.76407835	2.24E-10
ENSG00000215182	MUC5AC	0.134111946	0	8.76407835	2.24E-10
ENSG00000134532	SOX5	2.080271364	0.004285603	8.763319708	2.18E-28
ENSG00000196562	SULF2	9.013655894	0.020172655	8.751256137	8.00E-37
ENSG00000104549	SQLE	18.38365708	0.041591751	8.749607653	6.72E-39
ENSG00000267374	AC016205.1	0.826096259	0	8.738143474	2.96E-10
ENSG00000264334	AC134978.1	2.916497198	0	8.738143474	2.96E-10
ENSG00000088756	ARHGAP28	0.192963878	0	8.738143474	2.96E-10
ENSG00000183690	EFHC2	0.692437418	0	8.738143474	2.96E-10
ENSG00000144010	TRIM43B	1.377817621	0	8.738143474	2.96E-10
ENSG00000204219	TCEA3	6.038978374	0.012734885	8.729645041	3.82E-28
ENSG00000261804	AC007342.4	2.040157632	0	8.711733827	3.92E-10
ENSG00000278626	AC023310.4	3.471406678	0	8.711733827	3.92E-10
ENSG00000124343	XG	0.673757641	0	8.711733827	3.92E-10
ENSG00000108551	RASD1	86.51118209	0.204865547	8.705034805	4.60E-43
ENSG00000233695	GAS6-AS1	0.308917407	0	8.684831702	3.92E-10
ENSG00000228223	HCG11	0.388804229	0	8.684831702	3.92E-10
ENSG00000127954	STEAP4	0.196943432	0	8.684831702	3.92E-10
ENSG00000279924	AL356585.4	1.767976761	0	8.657418382	5.21E-10
ENSG00000270685	IGHV1OR15-6	6.334820523	0	8.657418382	5.21E-10
ENSG00000180155	LYNX1	0.432664962	0	8.657418382	5.21E-10
ENSG00000144115	THNSL2	0.337870228	0	8.657418382	5.21E-10
ENSG00000184292	TACSTD2	54.10247219	0.133280244	8.645001893	8.02E-42
ENSG00000039068	CDH1	40.818101	0.10145438	8.644417027	4.12E-45
ENSG00000254535	PABPC4L	4.03148463	0.00905403	8.638829632	1.63E-27
ENSG00000110492	MDK	186.22687	0.468830512	8.630791544	3.87E-47
ENSG00000272695	GAS6-DT	1.091730527	0	8.629474063	6.95E-10
ENSG00000169116	PARM1	0.423332934	0	8.629474063	6.95E-10
ENSG00000177599	ZNF491	0.702855537	0	8.629474063	6.95E-10
ENSG00000212747	RTL8B	9.652315378	0.022094335	8.611356403	2.57E-27
ENSG00000280027	AC007342.9	1.478330027	0	8.571907246	1.25E-09
ENSG00000228636	AL358214.1	2.652185346	0	8.571907246	1.25E-09
ENSG00000250305	TRMT9B	0.139588702	0	8.571907246	1.25E-09
ENSG00000178386	ZNF223	0.615045686	0	8.571907246	1.25E-09
ENSG00000049089	COL9A2	8.521576394	0.021229842	8.568982085	2.00E-32
ENSG00000258590	NBEAP1	5.131149144	0.012134216	8.564372098	5.29E-27

ENSG00000156968	MPV17L	5.910739415	0.014898693	8.552110814	2.67E-32
ENSG00000261116	AL049555.1	1.037610779	0	8.542238883	1.68E-09
ENSG00000050767	COL23A1	0.532581422	0	8.542238883	1.68E-09
ENSG00000183160	TMEM119	0.628943756	0	8.542238883	1.68E-09
ENSG00000182107	TMEM30B	7.859889082	0.020023763	8.536756117	3.47E-32
ENSG00000204186	ZDBF2	3.208963319	0.008204385	8.531601354	3.79E-32
ENSG00000131620	ANO1	68.38628663	0.185862489	8.520943574	1.27E-46
ENSG00000259126	AL161752.1	7.328045469	0	8.511947571	2.27E-09
ENSG00000078081	LAMP3	0.514653088	0	8.511947571	2.27E-09
ENSG00000242732	RTL5	0.410947099	0	8.511947571	2.27E-09
ENSG00000159445	THEM4	7.776472271	0.020393222	8.4949929	6.86E-32
ENSG00000178202	KDELC2	13.23945185	0.035860703	8.489940315	5.38E-37
ENSG00000052344	PRSS8	43.21531834	0.118971753	8.48775764	1.87E-41
ENSG00000204681	GABBR1	0.226933971	0	8.481006587	3.09E-09
ENSG00000115461	IGFBP5	0.298374842	0	8.481006587	3.09E-09
ENSG00000101746	NOL4	0.337986765	0	8.481006587	3.09E-09
ENSG00000006555	TTC22	0.317445208	0	8.481006587	3.09E-09
ENSG00000233098	CCDC144NL-AS1	6.411870146	0.017511246	8.478037043	6.52E-37
ENSG00000196867	ZFP28	2.522465553	0.006418572	8.458712524	2.83E-26
ENSG00000235984	GPC5-AS1	4.078840096	0	8.449387452	4.20E-09
ENSG00000220008	LINGO3	0.839065276	0	8.449387452	4.20E-09
ENSG00000157873	TNFRSF14	0.302354926	0	8.449387452	4.20E-09
ENSG00000230099	TRBV5-4	3.456517066	0	8.449387452	4.20E-09
ENSG00000214652	ZNF727	1.119919764	0	8.449387452	4.20E-09
ENSG00000182580	EPHB3	27.8631139	0.079016985	8.444957525	3.88E-41
ENSG00000168806	LCMT2	9.19556204	0.025737026	8.442668919	1.19E-36
ENSG00000281566	AL157778.1	0.696688076	0	8.417059771	5.75E-09
ENSG00000184254	ALDH1A3	0.31853081	0	8.417059771	5.75E-09
ENSG00000152785	BMP3	0.320641757	0	8.417059771	5.75E-09
ENSG00000230798	FOXD3-AS1	1.521986617	0	8.417059771	5.75E-09
ENSG00000261594	TPBGL	0.658274197	0	8.417059771	5.75E-09
ENSG00000173320	STOX2	4.194792512	0.011867212	8.413197472	2.28E-34
ENSG00000205097	FRG2	109.1428821	0.31928047	8.41018767	1.07E-43
ENSG00000233392	AC104809.2	0.676496379	0	8.38399106	7.89E-09
ENSG00000186081	KRT5	0.41863336	0	8.38399106	7.89E-09
ENSG00000233515	LINC01518	3.46199303	0	8.38399106	7.89E-09
ENSG00000198681	MAGEA1	1.050745253	0	8.38399106	7.89E-09
ENSG00000053328	METTL24	1.012837871	0	8.38399106	7.89E-09
ENSG00000242419	PCDHGC4	0.36123329	0	8.38399106	7.89E-09
ENSG00000077942	FBLN1	4.307601032	0.012289121	8.373489761	5.08E-31
ENSG00000140563	MCTP2	1.262127962	0.003449154	8.355778664	1.43E-25
ENSG00000261437	AC108860.2	0.803934463	0	8.350146548	1.09E-08

ENSG00000161905	ALOX15	0.515566666	0	8.350146548	1.09E-08
ENSG00000236081	ELFN1-AS1	1.774508526	0	8.350146548	1.09E-08
ENSG00000230316	FEZF1-AS1	0.52972802	0	8.350146548	1.09E-08
ENSG00000268089	GABRQ	0.284346878	0	8.350146548	1.09E-08
ENSG00000165731	RET	0.253941388	0	8.350146548	1.09E-08
ENSG00000183578	TNFAIP8L3	0.740189343	0	8.350146548	1.09E-08
ENSG00000141655	TNFRSF11A	0.195020439	0	8.350146548	1.09E-08
ENSG00000149418	ST14	37.10740144	0.113194102	8.345599942	1.17E-41
ENSG00000141934	PLPP2	22.73071694	0.069754121	8.336984865	1.36E-41
ENSG00000154065	ANKRD29	4.772864818	0.014008175	8.332580451	9.82E-31
ENSG00000261324	AC010168.2	0.315623338	0	8.315488957	1.51E-08
ENSG00000246022	ALDH1L1-AS2	0.63008587	0	8.315488957	1.51E-08
ENSG00000135905	DOCK10	0.152081978	0	8.315488957	1.51E-08
ENSG00000279516	FAM230C	0.869646437	0	8.315488957	1.51E-08
ENSG00000274750	HIST1H3E	0.639493647	0	8.315488957	1.51E-08
ENSG00000151704	KCNJ1	0.369463766	0	8.315488957	1.51E-08
ENSG00000153012	LGI2	0.26652201	0	8.315488957	1.51E-08
ENSG00000237687	LINC00686	5.160251449	0	8.315488957	1.51E-08
ENSG00000186462	NAP1L2	0.671844502	0	8.315488957	1.51E-08
ENSG00000107014	RLN2	1.340534805	0	8.315488957	1.51E-08
ENSG00000168803	ADAL	2.754123869	0.007805252	8.303328837	3.34E-25
ENSG00000223547	ZNF844	6.570793692	0.020285403	8.287219667	1.86E-33
ENSG00000086570	FAT2	17.30401139	0.05542658	8.281243244	1.48E-43
ENSG00000265972	TXNIP	8.115022713	0.024696895	8.280268837	2.30E-30
ENSG00000278704	BX004987.1	0.747169437	0	8.279978258	2.10E-08
ENSG00000109424	UCP1	1.143240787	0	8.279978258	2.10E-08
ENSG00000204789	ZNF204P	6.467418536	0.018674644	8.276371083	5.16E-25
ENSG00000198440	ZNF583	1.892269172	0.005553737	8.25285661	7.08E-25
ENSG00000145428	RNF175	0.371214711	0	8.243571388	2.93E-08
ENSG00000183072	NKX2-5	7.163790268	0.021376849	8.228952515	1.04E-24
ENSG00000166025	AMOTL1	5.488114011	0.017842799	8.226564666	4.44E-35
ENSG00000266602	AC008109.1	1.922333086	0	8.206221942	4.11E-08
ENSG00000163909	HEYL	0.433719511	0	8.206221942	4.11E-08
ENSG00000143171	RXRG	0.588964069	0	8.206221942	4.11E-08
ENSG00000230953	AC099677.1	8.83463816	0	8.167879813	5.79E-08
ENSG00000228933	AC107419.1	0.548248822	0	8.167879813	5.79E-08
ENSG00000242021	AC112493.1	1.743023312	0	8.167879813	5.79E-08
ENSG00000162687	KCNT2	0.217755166	0	8.167879813	5.79E-08
ENSG00000226686	LINC01535	0.706932637	0	8.167879813	5.79E-08
ENSG00000225285	LINC01770	1.816758729	0	8.167879813	5.79E-08
ENSG00000157502	MUM1L1	0.355989334	0	8.167879813	5.79E-08
ENSG00000144476	ACKR3	6.310380296	0.019824235	8.154767787	3.42E-24
ENSG00000198521	ZNF43	4.541371686	0.015526577	8.140001482	2.15E-32

ENSG00000162543	UBXN10	7.020495766	0.024105074	8.133850102	2.39E-32
ENSG00000274286	ADRA2B	0.446240352	0	8.128490788	8.20E-08
ENSG00000058866	DGKG	0.137026437	0	8.128490788	8.20E-08
ENSG00000111913	RIPOR2	0.157747088	0	8.128490788	8.20E-08
ENSG00000145626	UGT3A1	0.177286532	0	8.128490788	8.20E-08
ENSG00000060982	BCAT1	5.429447105	0.019007695	8.128288206	7.00E-36
ENSG00000162639	HENMT1	5.701147651	0.018451411	8.11184376	6.34E-24
ENSG00000165929	TC2N	8.272798694	0.029128435	8.111555191	3.05E-34
ENSG00000142173	COL6A2	9.340824863	0.033244056	8.096063067	3.91E-34
ENSG00000135439	AGAP2	0.217373778	0	8.087996091	1.17E-07
ENSG00000101938	CHRDL1	0.373084382	0	8.087996091	1.17E-07
ENSG00000116157	GPX7	21.84407952	0.079414173	8.051399646	9.24E-32
ENSG00000260211	AC139426.1	0.561765649	0	8.04633185	1.67E-07
ENSG00000230707	AL589987.1	1.734682937	0	8.04633185	1.67E-07
ENSG00000239911	PRKAG2-AS1	1.221586695	0	8.04633185	1.67E-07
ENSG00000265790	RNASEH1P1	1.914697205	0	8.04633185	1.67E-07
ENSG00000242675	RPS16P9	3.327178749	0	8.04633185	1.67E-07
ENSG00000144868	TMEM108	0.168629712	0	8.04633185	1.67E-07
ENSG00000233922	LINC01694	10.58442035	0.039438874	8.043992587	1.91E-36
ENSG00000240225	ZNF542P	5.318958231	0.019088751	8.042471749	1.12E-28
ENSG00000062038	CDH3	11.44476426	0.043069072	8.029705274	2.44E-36
ENSG00000145703	IQGAP2	6.037320947	0.022653402	8.019798723	1.39E-33
ENSG00000246582	AC100861.1	0.563975409	0	8.003428494	2.40E-07
ENSG00000234147	AL035446.1	3.043973234	0	8.003428494	2.40E-07
ENSG00000184574	LPAR5	0.423241214	0	8.003428494	2.40E-07
ENSG00000268606	MAGEA2	0.606918959	0	8.003428494	2.40E-07
ENSG00000280109	PLAC4	0.130455996	0	8.003428494	2.40E-07
ENSG00000102445	RUBCNL	0.225867301	0	8.003428494	2.40E-07
ENSG00000170542	SERPINB9	0.334851839	0	8.003428494	2.40E-07
ENSG00000196972	SMIM10L2B	0.503807042	0	8.003428494	2.40E-07
ENSG00000185633	NDUFA4L2	8.637049693	0.03190529	8.00080286	2.15E-28
ENSG00000152229	PSTPIP2	5.821565227	0.021729671	7.985804053	2.76E-28
ENSG00000178467	P4HTM	6.031848212	0.023433122	7.96967463	3.22E-33
ENSG00000263567	AC007923.1	2.647790939	0	7.959210057	3.48E-07
ENSG00000248268	AC010275.1	2.3666096	0	7.959210057	3.48E-07
ENSG00000267383	AC011447.3	0.362957227	0	7.959210057	3.48E-07
ENSG00000260711	AL121839.2	0.192587415	0	7.959210057	3.48E-07
ENSG00000270038	AL133467.4	1.844323344	0	7.959210057	3.48E-07
ENSG00000164743	C8orf48	0.925352543	0	7.959210057	3.48E-07
ENSG00000179776	CDH5	0.219851106	0	7.959210057	3.48E-07
ENSG00000171812	COL8A2	0.286324288	0	7.959210057	3.48E-07
ENSG00000132704	FCRL2	0.264360305	0	7.959210057	3.48E-07
ENSG00000134460	IL2RA	0.337489759	0	7.959210057	3.48E-07

ENSG00000137033	IL33	0.411046549	0	7.959210057	3.48E-07
ENSG00000225255	LINC01297	1.563899911	0	7.959210057	3.48E-07
ENSG00000253873	PCDHGA11	0.229985281	0	7.959210057	3.48E-07
ENSG00000159247	TUBBP5	0.475172148	0	7.959210057	3.48E-07
ENSG00000088881	EBF4	3.338789044	0.012042809	7.955539865	7.02E-23
ENSG00000007062	PROM1	6.109871533	0.024085618	7.948596264	4.63E-33
ENSG00000129422	MTUS1	2.832086338	0.011181463	7.932400187	6.61E-31
ENSG00000182272	B4GALNT4	3.233067315	0.01190192	7.926109012	1.12E-22
ENSG00000160216	AGPAT3	8.75955625	0.035664669	7.925603336	1.94E-37
ENSG00000119411	BSPRY	9.807700926	0.038275436	7.921574238	7.69E-28
ENSG00000173467	AGR3	1.11668015	0	7.913593359	5.06E-07
ENSG00000205667	ARSH	0.690852786	0	7.913593359	5.06E-07
ENSG00000142609	CFAP74	0.124289865	0	7.913593359	5.06E-07
ENSG00000254349	MIR2052HG	0.445749819	0	7.913593359	5.06E-07
ENSG00000160321	ZNF208	0.188633897	0	7.913593359	5.06E-07
ENSG00000139714	MORN3	3.088395608	0.011608693	7.896065247	1.79E-22
ENSG00000205890	AC108134.1	0.409527449	0	7.866487076	7.40E-07
ENSG00000187721	GTF2IP3	2.537577981	0	7.866487076	7.40E-07
ENSG00000138795	LEF1	0.233830167	0	7.866487076	7.40E-07
ENSG00000122378	PRXL2A	7.940517406	0.034327548	7.823961329	1.15E-33
ENSG00000168824	NSG1	2.590601999	0.010278558	7.818099026	5.57E-22
ENSG00000279628	AC131280.1	0.199601066	0	7.817790631	1.09E-06
ENSG00000236304	AP001189.1	0.849774244	0	7.817790631	1.09E-06
ENSG00000282870	FRG1DP	1.571696591	0	7.817790631	1.09E-06
ENSG00000248131	LINC01194	0.64766332	0	7.817790631	1.09E-06
ENSG00000266916	ZNF793-AS1	0.917318752	0	7.817790631	1.09E-06
ENSG00000165215	CLDN3	71.9929862	0.316222997	7.816161393	1.22E-36
ENSG00000268916	CSAG3	28.86012665	0.123997568	7.810429849	4.90E-30
ENSG00000177932	ZNF354C	3.907491791	0.016545231	7.803934298	5.16E-27
ENSG00000154342	WNT3A	3.776652268	0.015267095	7.791144988	8.47E-22
ENSG00000160588	MPZL3	5.254773167	0.022562057	7.783841891	7.19E-27
ENSG00000260807	AC009041.2	0.299613988	0	7.767392904	1.61E-06
ENSG00000273305	AC009237.15	2.335314613	0	7.767392904	1.61E-06
ENSG00000280079	AC011447.7	0.735844416	0	7.767392904	1.61E-06
ENSG00000228836	CT45A5	1.007745582	0	7.767392904	1.61E-06
ENSG00000128610	FEZF1	0.447929794	0	7.767392904	1.61E-06
ENSG00000053108	FSTL4	0.173744078	0	7.767392904	1.61E-06
ENSG00000157404	KIT	0.210316847	0	7.767392904	1.61E-06
ENSG00000229544	NKX1-2	0.347798044	0	7.767392904	1.61E-06
ENSG00000132329	RAMP1	8.770204008	0.036274815	7.758121	1.41E-21
ENSG00000134202	GSTM3	4.301702194	0.01884763	7.754644172	1.11E-26
ENSG00000020633	RUNX3	10.75354072	0.049483885	7.733885885	5.10E-33
ENSG00000234476	AC092811.1	1.137305615	0	7.715170703	2.40E-06

ENSG00000138316	ADAMTS14	0.214121687	0	7.715170703	2.40E-06
ENSG00000134326	CMPK2	0.262251783	0	7.715170703	2.40E-06
ENSG00000235436	DPY19L2P4	0.559071938	0	7.715170703	2.40E-06
ENSG00000198417	MT1F	0.597251017	0	7.715170703	2.40E-06
ENSG00000158352	SHROOM4	0.109184861	0	7.715170703	2.40E-06
ENSG00000100170	SLC5A1	0.212468394	0	7.715170703	2.40E-06
ENSG00000145335	SNCA	0.281068054	0	7.715170703	2.40E-06
ENSG00000278318	ZNF229	0.204942265	0	7.715170703	2.40E-06
ENSG00000159495	TGM7	4.457114748	0.019252956	7.695540904	3.72E-21
ENSG00000167741	GGT6	6.199739451	0.028322127	7.694413867	2.98E-26
ENSG00000119715	ESRRB	1.719333893	0.007548587	7.672094237	5.34E-21
ENSG00000274835	AC100757.2	3.88007757	0	7.660986956	3.61E-06
ENSG00000261888	AC144831.1	0.409044322	0	7.660986956	3.61E-06
ENSG00000279029	AL353583.1	0.585672086	0	7.660986956	3.61E-06
ENSG00000151640	DPYSL4	0.34207233	0	7.660986956	3.61E-06
ENSG00000087258	GNAO1	0.079318224	0	7.660986956	3.61E-06
ENSG00000153885	KCTD15	0.170072279	0	7.660986956	3.61E-06
ENSG00000182170	MRGPRG	1.248760597	0	7.660986956	3.61E-06
ENSG00000230873	STMND1	0.37696798	0	7.660986956	3.61E-06
ENSG00000103528	SYT17	0.144125991	0	7.660986956	3.61E-06
ENSG00000156049	GNA14	10.07396387	0.049352946	7.621122309	1.07E-28
ENSG00000134769	DTNA	0.77723452	0.003543068	7.617903065	1.12E-20
ENSG00000172005	MAL	6.710909731	0.030722802	7.611754223	1.23E-20
ENSG00000267886	AC074135.1	1.080285697	0	7.604688532	5.45E-06
ENSG00000277400	AC145212.1	0.479410862	0	7.604688532	5.45E-06
ENSG00000285621	AL138999.2	0.796216668	0	7.604688532	5.45E-06
ENSG00000257869	AL139023.1	2.056370608	0	7.604688532	5.45E-06
ENSG00000186377	CYP4X1	0.409982837	0	7.604688532	5.45E-06
ENSG00000165023	DIRAS2	0.222927074	0	7.604688532	5.45E-06
ENSG00000117228	GBP1	0.209262073	0	7.604688532	5.45E-06
ENSG00000184350	MRGPRE	0.741402604	0	7.604688532	5.45E-06
ENSG00000196119	OR8A1	0.1715894	0	7.604688532	5.45E-06
ENSG00000198754	OXCT2	0.572089961	0	7.604688532	5.45E-06
ENSG00000070526	ST6GALNAC1	7.70752514	0.038947032	7.576462186	2.18E-28
ENSG00000213949	ITGA1	3.709060802	0.019288214	7.57022149	1.03E-34
ENSG00000124749	COL21A1	0.14168562	0	7.546103643	8.28E-06
ENSG00000187140	FOXD3	0.480753029	0	7.546103643	8.28E-06
ENSG00000213626	LBH	0.213508797	0	7.546103643	8.28E-06
ENSG00000168671	UGT3A2	0.372253459	0	7.546103643	8.28E-06
ENSG00000247746	USP51	0.227558615	0	7.546103643	8.28E-06
ENSG00000245680	ZNF585B	3.392504094	0.017814986	7.520978556	5.30E-28
ENSG00000187172	BAGE2	4.209269672	0.021942707	7.504020366	6.01E-25
ENSG00000219159	AC011298.1	0.282832657	0	7.485038676	1.27E-05

ENSG00000248783	AC106771.1	2.583509052	0	7.485038676	1.27E-05
ENSG00000234626	AL021937.3	2.662230935	0	7.485038676	1.27E-05
ENSG00000248713	C4orf54	0.095287068	0	7.485038676	1.27E-05
ENSG00000133083	DCLK1	0.066856721	0	7.485038676	1.27E-05
ENSG00000251165	F11-AS1	0.358205504	0	7.485038676	1.27E-05
ENSG00000124391	IL17C	0.6969292	0	7.485038676	1.27E-05
ENSG00000236301	MRGPRG-AS1	0.477666684	0	7.485038676	1.27E-05
ENSG00000113396	SLC27A6	0.275140386	0	7.485038676	1.27E-05
ENSG00000258484	SPESP1	10.60424223	0.056376728	7.475670804	9.51E-25
ENSG00000178498	DTX3	6.062826596	0.032857687	7.475488757	1.13E-27
ENSG00000078328	RBFOX1	1.64730707	0.008845593	7.461284343	1.20E-24
ENSG00000117122	MFAP2	2.981603347	0.015356131	7.441928659	1.45E-19
ENSG00000166145	SPINT1	50.51533036	0.290669623	7.438234106	2.76E-38
ENSG00000151322	NPAS3	4.277571304	0.024219847	7.434743785	7.22E-31
ENSG00000236431	AC009237.8	2.425540677	0	7.421274334	1.96E-05
ENSG00000275038	AC091980.2	0.300811491	0	7.421274334	1.96E-05
ENSG00000277693	AP003900.1	0.991671971	0	7.421274334	1.96E-05
ENSG00000242173	ARHGDIG	0.378460237	0	7.421274334	1.96E-05
ENSG00000279973	CU104787.1	3.191944155	0	7.421274334	1.96E-05
ENSG00000188816	HMX2	0.564668253	0	7.421274334	1.96E-05
ENSG00000142149	HUNK	0.110836739	0	7.421274334	1.96E-05
ENSG00000187537	POTEG	0.366392952	0	7.421274334	1.96E-05
ENSG00000230082	PRRT3-AS1	1.497198561	0	7.421274334	1.96E-05
ENSG00000267454	ZNF582-AS1	0.451291073	0	7.421274334	1.96E-05
ENSG00000283023	FRG1GP	11.06720595	0.057833491	7.420982137	1.98E-19
ENSG00000185352	HS6ST3	1.092010243	0.005734451	7.41393183	2.20E-19
ENSG00000136167	LCP1	159.9009731	0.955229633	7.390067889	3.47E-41
ENSG00000104081	BMF	4.280573072	0.024653822	7.387741471	4.66E-27
ENSG00000138449	SLC40A1	3.62848256	0.020675807	7.375656804	4.51E-24
ENSG00000104892	KLC3	2.241365175	0.012250444	7.356253575	5.19E-19
ENSG00000279377	AC003973.3	0.31094772	0	7.354560884	3.05E-05
ENSG00000279741	AC007342.8	1.294239625	0	7.354560884	3.05E-05
ENSG00000254810	AP001189.3	1.347917766	0	7.354560884	3.05E-05
ENSG00000108556	CHRNE	0.306387733	0	7.354560884	3.05E-05
ENSG00000258754	LINC01579	0.129138258	0	7.354560884	3.05E-05
ENSG00000198062	POTEH	0.382684024	0	7.354560884	3.05E-05
ENSG00000135643	KCNMB4	3.163486116	0.018368126	7.348554352	6.97E-24
ENSG00000069812	HES2	2.797546361	0.016557471	7.32093299	1.02E-23
ENSG00000215845	TSTD1	18.19911781	0.110982947	7.305298941	1.75E-26
ENSG00000215548	FRG1JP	8.48276068	0.04808069	7.303821327	1.13E-18
ENSG00000261241	LINC02128	1.968344962	0.011216017	7.296172993	1.26E-18
ENSG00000114654	EFCC1	0.283772161	0	7.284612248	4.78E-05
ENSG00000164616	FBXL21	0.21583394	0	7.284612248	4.78E-05

ENSG00000275714	HIST1H3A	2.033355268	0	7.284612248	4.78E-05
ENSG00000205293	LINC01602	0.343206988	0	7.284612248	4.78E-05
ENSG00000248238	LINC02438	1.303758214	0	7.284612248	4.78E-05
ENSG00000205847	OR7E91P	0.697586824	0	7.284612248	4.78E-05
ENSG00000214842	RAD51AP2	0.224411658	0	7.284612248	4.78E-05
ENSG00000183145	RIPPLY3	0.360374737	0	7.284612248	4.78E-05
ENSG00000101276	SLC52A3	3.895253884	0.023709281	7.280533334	1.94E-23
ENSG00000148734	NPFFR1	0.866529357	0.005017725	7.272981657	1.78E-18
ENSG00000164684	ZNF704	4.187454554	0.026843557	7.270784004	1.16E-32
ENSG00000005379	TSPOAP1	0.677164254	0.003942498	7.26516762	1.99E-18
ENSG00000007372	PAX6	1.019940851	0.006452497	7.252337691	4.00E-26
ENSG00000140832	MARVELD3	3.286568963	0.020832976	7.249495665	4.19E-26
ENSG00000120708	TGFBI	23.05957654	0.15475087	7.217429368	3.22E-37
ENSG00000285416	AC137630.5	1.040528918	0	7.21109859	4.78E-05
ENSG00000253217	AP001574.1	1.454072462	0	7.21109859	4.78E-05
ENSG00000129993	CBFA2T3	0.132940985	0	7.21109859	4.78E-05
ENSG00000187821	HELT	0.642332981	0	7.21109859	4.78E-05
ENSG00000158748	HTR6	0.400163087	0	7.21109859	4.78E-05
ENSG00000225174	OSTM1-AS1	0.229989445	0	7.21109859	4.78E-05
ENSG00000253767	PCDHGA8	0.091666501	0	7.21109859	4.78E-05
ENSG00000175093	SPSB4	0.212279028	0	7.21109859	4.78E-05
ENSG00000176933	TOB2P1	0.800326174	0	7.21109859	4.78E-05
ENSG00000169059	VCX3A	0.79791313	0	7.21109859	4.78E-05
ENSG00000258405	ZNF578	0.110435883	0	7.21109859	4.78E-05
ENSG00000073150	PANX2	2.384276204	0.014430407	7.209251219	4.02E-18
ENSG00000081923	ATP8B1	41.40493442	0.280247206	7.207583998	2.37E-38
ENSG00000109265	KIAA1211	3.023172716	0.019909469	7.194405365	9.87E-26
ENSG00000182632	CCNYL2	5.311802276	0.035053345	7.191446773	1.04E-25
ENSG00000003989	SLC7A2	1.733792264	0.011325268	7.178692423	9.18E-23
ENSG00000101098	RIMS4	1.380541208	0.008598371	7.167940991	7.32E-18
ENSG00000165140	FBP1	4.084834174	0.025740726	7.151079902	9.35E-18
ENSG00000184160	ADRA2C	6.098106168	0.041085931	7.134041223	1.86E-22
ENSG00000224513	AC109309.1	1.729053135	0	7.133636911	7.55E-05
ENSG00000279602	AC109326.1	0.56937026	0	7.133636911	7.55E-05
ENSG00000266995	AP001542.1	3.760690568	0	7.133636911	7.55E-05
ENSG00000223658	C1GALT1C1L	0.64175607	0	7.133636911	7.55E-05
ENSG00000110848	CD69	0.265585492	0	7.133636911	7.55E-05
ENSG00000149972	CNTN5	0.082625301	0	7.133636911	7.55E-05
ENSG00000121361	KCNJ8	0.302793121	0	7.133636911	7.55E-05
ENSG00000205628	LINC01446	0.2158835	0	7.133636911	7.55E-05
ENSG00000250584	LINC01511	0.444526072	0	7.133636911	7.55E-05
ENSG00000253417	LINC02159	0.323778783	0	7.133636911	7.55E-05
ENSG00000103710	RASL12	0.26842902	0	7.133636911	7.55E-05

ENSG00000104055	TGM5	0.207143518	0	7.133636911	7.55E-05
ENSG00000183833	MAATS1	0.982011016	0.006299342	7.125412925	1.35E-17
ENSG00000149054	ZNF215	1.202354797	0.007759252	7.116754778	1.53E-17
ENSG00000099822	HCN2	30.88543147	0.223290221	7.106269963	1.22E-34
ENSG00000151388	ADAMTS12	1.3204852	0.009155885	7.092638348	3.57E-22
ENSG00000175701	MTLN	3.320832995	0.021825023	7.090464135	2.24E-17
ENSG00000267191	AC006213.3	1.373989677	0	7.051778972	0.00012
ENSG00000277806	AC006213.4	1.120430068	0	7.051778972	0.00012
ENSG00000279864	AC124864.2	3.819100338	0	7.051778972	0.00012
ENSG00000277493	AC139769.3	4.059158074	0	7.051778972	0.00012
ENSG00000283766	AC141930.2	0.932221342	0	7.051778972	0.00012
ENSG00000285771	AL139095.5	0.599453724	0	7.051778972	0.00012
ENSG00000244731	C4A	0.098783572	0	7.051778972	0.00012
ENSG00000102678	FGF9	0.156499816	0	7.051778972	0.00012
ENSG00000134363	FST	0.239256539	0	7.051778972	0.00012
ENSG00000128713	HOXD11	0.33666003	0	7.051778972	0.00012
ENSG00000066735	KIF26A	0.10514397	0	7.051778972	0.00012
ENSG00000164344	KLKB1	0.240552883	0	7.051778972	0.00012
ENSG00000139648	KRT71	0.313621485	0	7.051778972	0.00012
ENSG00000248307	LINC00616	0.300360534	0	7.051778972	0.00012
ENSG00000258819	LINC02289	0.254333213	0	7.051778972	0.00012
ENSG00000141506	PIK3R5	0.094637978	0	7.051778972	0.00012
ENSG00000204434	POTEKP	0.264268104	0	7.051778972	0.00012
ENSG00000185924	RTN4RL1	0.195959355	0	7.051778972	0.00012
ENSG00000241697	TMEFF1	0.272061533	0	7.051778972	0.00012
ENSG00000206077	ZDHHC11B	0.146767079	0	7.051778972	0.00012
ENSG00000152284	TCF7L1	1.495718446	0.010141169	7.045552861	4.26E-17
ENSG00000103534	TMC5	1.026171714	0.007001896	7.036400364	4.85E-17
ENSG00000182240	BACE2	9.205049398	0.069804224	7.036030954	1.42E-33
ENSG00000090661	CERS4	27.5378371	0.2095342	7.033561502	1.42E-34
ENSG00000136378	ADAMTS7	8.173299036	0.063699139	6.99241137	9.62E-32
ENSG00000162738	VANGL2	1.189398598	0.008382607	6.989746121	9.41E-17
ENSG00000106541	AGR2	6.139303486	0.047201886	6.971091058	3.38E-24
ENSG00000269696	AC005498.3	0.326927732	0	6.964995578	0.000194
ENSG00000283355	AC074194.2	0.284739017	0	6.964995578	0.000194
ENSG00000253983	AC087627.1	0.673280173	0	6.964995578	0.000194
ENSG00000075340	ADD2	0.052755244	0	6.964995578	0.000194
ENSG00000279081	AL356585.1	0.745336914	0	6.964995578	0.000194
ENSG00000228955	AL591623.2	3.011564019	0	6.964995578	0.000194
ENSG00000225678	AP000619.1	0.668567212	0	6.964995578	0.000194
ENSG00000126947	ARMCX1	0.312268665	0	6.964995578	0.000194
ENSG00000249082	C5orf66-AS1	0.793080916	0	6.964995578	0.000194
ENSG00000133878	DUSP26	0.325812482	0	6.964995578	0.000194

ENSG00000139515	PDX1	0.267319957	0	6.964995578	0.000194
ENSG00000250591	PRSS3P1	0.901033979	0	6.964995578	0.000194
ENSG00000007350	TKTL1	0.183169099	0	6.964995578	0.000194
ENSG00000163013	FBXO41	2.169859882	0.016763121	6.964173785	3.78E-24
ENSG00000169783	LINGO1	2.395290017	0.018263208	6.955661677	2.83E-21
ENSG00000137462	TLR2	2.250663535	0.017160484	6.955661677	2.83E-21
ENSG00000183117	CSMD1	0.625497258	0.00478622	6.95052074	3.06E-21
ENSG00000168952	STXBP6	20.53790904	0.166467542	6.945706284	1.47E-35
ENSG00000258949	AL049870.3	61.98175196	0.497368022	6.923333385	8.47E-26
ENSG00000159374	M1AP	7.437810235	0.059684163	6.923333385	8.47E-26
ENSG00000165521	EML5	0.550808215	0.004069375	6.921786802	2.14E-16
ENSG00000255363	AP001189.5	9.713603336	0.077848908	6.911209236	8.88E-24
ENSG00000180353	HCLS1	4.340173464	0.035156585	6.909764662	1.06E-25
ENSG00000124507	PACSIN1	1.225660195	0.00931207	6.881465472	3.75E-16
ENSG00000138207	RBP4	4.066079059	0.030892424	6.881465472	3.75E-16
ENSG00000267327	AC009271.1	0.660465502	0	6.872655826	0.000315
ENSG00000269397	AC011503.2	0.417854508	0	6.872655826	0.000315
ENSG00000275155	AC027348.1	1.019157336	0	6.872655826	0.000315
ENSG00000266369	AC090774.2	0.456838018	0	6.872655826	0.000315
ENSG00000274310	AC091076.1	1.012571505	0	6.872655826	0.000315
ENSG00000257829	AC121757.1	1.004457951	0	6.872655826	0.000315
ENSG00000277702	AC239859.6	2.942637377	0	6.872655826	0.000315
ENSG00000251002	AC244502.1	0.190859245	0	6.872655826	0.000315
ENSG00000280604	AJ239328.1	0.130389382	0	6.872655826	0.000315
ENSG00000284668	AL805961.1	0.342503695	0	6.872655826	0.000315
ENSG00000133169	BEX1	0.725441853	0	6.872655826	0.000315
ENSG00000184492	FOXD4L1	0.251618531	0	6.872655826	0.000315
ENSG00000282995	FRG1EP	0.814002287	0	6.872655826	0.000315
ENSG00000278828	HIST1H3H	0.506695037	0	6.872655826	0.000315
ENSG00000139679	LPAR6	0.144154039	0	6.872655826	0.000315
ENSG00000120289	MAGEB4	0.292615201	0	6.872655826	0.000315
ENSG00000185155	MIXL1	0.308911662	0	6.872655826	0.000315
ENSG00000196341	OR8D1	0.073678354	0	6.872655826	0.000315
ENSG00000120328	PCDHB12	0.161750132	0	6.872655826	0.000315
ENSG00000168016	TRANK1	0.051485277	0	6.872655826	0.000315
ENSG00000182583	VCX	0.594105935	0	6.872655826	0.000315
ENSG00000149582	TMEM25	5.751262078	0.048576367	6.863501345	5.99E-28
ENSG00000136010	ALDH1L2	11.67065135	0.09997641	6.862566208	2.50E-33
ENSG00000100604	CHGA	5.172667018	0.04329122	6.848733867	2.29E-23
ENSG00000279114	Z99129.3	1.663619812	0.013923211	6.821300056	2.08E-20
ENSG00000049283	EPN3	6.643184545	0.057858408	6.819233334	1.22E-27
ENSG00000071967	CYBRD1	3.955732394	0.03459283	6.799306705	5.90E-25
ENSG00000176597	B3GNT5	3.110808742	0.027495775	6.792331842	2.50E-26

ENSG00000279400	AC008957.3	0.260926098	0	6.773999253	0.000517
ENSG00000255000	AC139103.1	0.567957583	0	6.773999253	0.000517
ENSG00000077522	ACTN2	0.105785951	0	6.773999253	0.000517
ENSG00000285407	AL033530.1	0.20818374	0	6.773999253	0.000517
ENSG00000277386	AL138999.1	1.167657307	0	6.773999253	0.000517
ENSG00000258932	AL390334.1	0.351138242	0	6.773999253	0.000517
ENSG00000270025	BMS1P7	0.556080143	0	6.773999253	0.000517
ENSG00000176753	C15orf56	0.253684437	0	6.773999253	0.000517
ENSG00000180044	C3orf80	0.215230431	0	6.773999253	0.000517
ENSG00000249035	CLMAT3	0.266999685	0	6.773999253	0.000517
ENSG00000186417	GLDN	0.081886382	0	6.773999253	0.000517
ENSG00000147255	IGSF1	0.082966432	0	6.773999253	0.000517
ENSG00000224559	LINC01087	0.166381203	0	6.773999253	0.000517
ENSG00000257084	MIR200CHG	1.67141803	0	6.773999253	0.000517
ENSG00000230937	MIR205HG	0.166286615	0	6.773999253	0.000517
ENSG00000166819	PLIN1	0.15406803	0	6.773999253	0.000517
ENSG00000176381	PRR18	0.18577209	0	6.773999253	0.000517
ENSG00000128276	RFPL3	0.389737715	0	6.773999253	0.000517
ENSG00000138083	SIX3	0.231865363	0	6.773999253	0.000517
ENSG00000050438	SLC4A8	0.032535946	0	6.773999253	0.000517
ENSG00000213231	TCL1B	0.310837572	0	6.773999253	0.000517
ENSG00000274391	TPTE	0.225345266	0	6.773999253	0.000517
ENSG00000112494	UNC93A	0.216745576	0	6.773999253	0.000517
ENSG00000114200	BCHE	8.080508972	0.07262025	6.768331535	3.72E-26
ENSG00000121316	PLBD1	5.280174876	0.047268344	6.751645166	1.01E-22
ENSG00000138821	SLC39A8	11.15568361	0.103618338	6.743439167	1.87E-31
ENSG00000105707	HPN	1.582407603	0.013243527	6.742048152	2.55E-15
ENSG00000226363	HAGLROS	4.733944912	0.03993142	6.730744285	2.98E-15
ENSG00000116194	ANGPTL1	4.334488427	0.039462053	6.72733832	1.49E-22
ENSG00000104213	PDGFRL	2.150849271	0.01858162	6.696291354	4.75E-15
ENSG00000129538	RNASE1	3.440501922	0.029723189	6.696291354	4.75E-15
ENSG00000273274	ZBTB8B	0.403724162	0.003487854	6.696291354	4.75E-15
ENSG00000115705	TPO	0.608022056	0.005295531	6.684621748	5.57E-15
ENSG00000197863	ZNF790	2.506476013	0.023247532	6.673127796	1.81E-19
ENSG00000010361	FUZ	1.728076269	0.01517394	6.672856979	6.52E-15
ENSG00000214754	AC004870.1	0.615885329	0	6.668097731	0.000858
ENSG00000276900	AC023157.3	0.260158458	0	6.668097731	0.000858
ENSG00000249753	AC084357.2	1.975312218	0	6.668097731	0.000858
ENSG00000250385	AC106772.2	1.228983846	0	6.668097731	0.000858
ENSG00000251468	AC135352.1	0.383082412	0	6.668097731	0.000858
ENSG00000254912	AC135983.3	0.443075742	0	6.668097731	0.000858
ENSG00000250687	AC146944.2	0.492038822	0	6.668097731	0.000858
ENSG00000166743	ACSM1	0.189206151	0	6.668097731	0.000858

ENSG00000163286	ALPG	0.217894448	0	6.668097731	0.000858
ENSG00000241838	AP000529.1	0.483714034	0	6.668097731	0.000858
ENSG00000179071	CCDC89	0.384710241	0	6.668097731	0.000858
ENSG00000278530	CHMP1B2P	0.077094928	0	6.668097731	0.000858
ENSG00000259726	CSPG4P11	0.110633576	0	6.668097731	0.000858
ENSG00000271449	CT45A2	0.411211855	0	6.668097731	0.000858
ENSG00000235857	CTBP2P1	2.217187183	0	6.668097731	0.000858
ENSG00000236417	CTSLP1	0.542126607	0	6.668097731	0.000858
ENSG00000099958	DERL3	0.143100859	0	6.668097731	0.000858
ENSG00000189269	DRICH1	0.417212642	0	6.668097731	0.000858
ENSG00000264859	DSG2-AS1	0.268384812	0	6.668097731	0.000858
ENSG00000164116	GUCY1A1	0.094209306	0	6.668097731	0.000858
ENSG00000240990	HOXA11-AS	0.173660761	0	6.668097731	0.000858
ENSG00000183960	KCNH8	0.088355703	0	6.668097731	0.000858
ENSG00000170298	LGALS9B	0.20344976	0	6.668097731	0.000858
ENSG00000225826	LINC00626	0.568807183	0	6.668097731	0.000858
ENSG00000205054	LINC01121	0.125308157	0	6.668097731	0.000858
ENSG00000258710	LINC01193	0.133993799	0	6.668097731	0.000858
ENSG00000250986	LINC02600	0.120046599	0	6.668097731	0.000858
ENSG00000184709	LRRC26	0.449305922	0	6.668097731	0.000858
ENSG00000155495	MAGEC1	0.127215658	0	6.668097731	0.000858
ENSG00000166670	MMP10	0.308993663	0	6.668097731	0.000858
ENSG00000253159	PCDHGA12	0.112816378	0	6.668097731	0.000858
ENSG00000107593	PKD2L1	0.177230297	0	6.668097731	0.000858
ENSG00000148123	PLPPR1	0.1480542	0	6.668097731	0.000858
ENSG00000248483	POU5F2	0.064529682	0	6.668097731	0.000858
ENSG00000160181	TFF2	0.654470915	0	6.668097731	0.000858
ENSG00000232354	VIPR1-AS1	0.104523929	0	6.668097731	0.000858
ENSG00000150051	MKX	5.637881081	0.054509403	6.662928867	1.95E-25
ENSG00000005102	MEOX1	8.536148603	0.082680314	6.660322979	2.03E-25
ENSG00000131668	BARX1	12.98006124	0.125951384	6.657712376	2.12E-25
ENSG00000112655	PTK7	12.44209953	0.124011309	6.646280839	8.90E-33
ENSG00000137558	PI15	0.694575868	0.006252706	6.636975852	1.06E-14
ENSG00000145248	SLC10A4	2.777915441	0.025007331	6.636975852	1.06E-14
ENSG00000174370	C11orf45	3.677119667	0.035810498	6.630172922	6.49E-22
ENSG00000237440	ZNF737	3.872740279	0.037945568	6.621406153	7.46E-22
ENSG00000189221	MAOA	2.4490749	0.024292577	6.603711159	9.85E-22
ENSG00000137343	ATAT1	2.278264973	0.022187421	6.602778761	5.23E-19
ENSG00000145526	CDH18	10.77139891	0.110685651	6.594837405	2.53E-29
ENSG00000170579	DLGAP1	0.396937621	0.003697598	6.587702521	1.73E-14
ENSG00000165816	VWA2	1.455718521	0.013560473	6.587702521	1.73E-14
ENSG00000228613	AC141930.1	6.060485224	0.056950537	6.575116675	2.04E-14
ENSG00000255983	AC007848.1	0.356886412	0	6.553802243	0.001437

ENSG00000284526	AC015802.6	0.088685074	0	6.553802243	0.001437
ENSG00000259363	AC090825.1	0.388400782	0	6.553802243	0.001437
ENSG00000259536	AC091045.1	0.216038522	0	6.553802243	0.001437
ENSG00000231482	AC105450.1	0.19518311	0	6.553802243	0.001437
ENSG00000268081	AC123912.1	1.121757067	0	6.553802243	0.001437
ENSG00000172482	AGXT	0.112452435	0	6.553802243	0.001437
ENSG00000260691	ANKRD20A1	0.12900062	0	6.553802243	0.001437
ENSG00000123685	BATF3	0.184823225	0	6.553802243	0.001437
ENSG00000251079	BMS1P2	0.232896149	0	6.553802243	0.001437
ENSG00000240240	BX664727.3	0.223054008	0	6.553802243	0.001437
ENSG00000064195	DLX3	0.184687075	0	6.553802243	0.001437
ENSG00000188573	FBLL1	0.33010231	0	6.553802243	0.001437
ENSG00000163534	FCRL1	0.127427042	0	6.553802243	0.001437
ENSG00000237009	GLIS3-AS1	0.683140884	0	6.553802243	0.001437
ENSG00000099984	GSTT2	0.42350119	0	6.553802243	0.001437
ENSG00000109758	HGFAC	0.176124134	0	6.553802243	0.001437
ENSG00000164120	HPGD	0.090690072	0	6.553802243	0.001437
ENSG00000267313	KC6	0.096800272	0	6.553802243	0.001437
ENSG00000162975	KCNF1	0.21905872	0	6.553802243	0.001437
ENSG00000251273	LINC02228	0.129400106	0	6.553802243	0.001437
ENSG00000258487	LINC02277	0.742852458	0	6.553802243	0.001437
ENSG00000259823	LYPD8	0.224150831	0	6.553802243	0.001437
ENSG00000120729	MYOT	0.173323681	0	6.553802243	0.001437
ENSG00000183251	OR51B4	0.53743345	0	6.553802243	0.001437
ENSG00000175426	PCSK1	0.094483778	0	6.553802243	0.001437
ENSG00000185668	POU3F1	0.229590389	0	6.553802243	0.001437
ENSG00000180739	S1PR5	0.17869758	0	6.553802243	0.001437
ENSG00000198022	SAGE2P	0.235853908	0	6.553802243	0.001437
ENSG00000157150	TIMP4	0.303894187	0	6.553802243	0.001437
ENSG00000124731	TREM1	0.115829385	0	6.553802243	0.001437
ENSG00000150630	VEGFC	0.207200582	0	6.553802243	0.001437
ENSG00000183066	WBP2NL	0.081136798	0	6.553802243	0.001437
ENSG00000102383	ZDHHC15	0.073522787	0	6.553802243	0.001437
ENSG00000236869	ZKSCAN7-AS1	1.099617125	0	6.553802243	0.001437
ENSG00000118156	ZNF541	0.107717596	0	6.553802243	0.001437
ENSG00000269421	ZNF92P3	0.525603154	0	6.553802243	0.001437
ENSG00000110042	DTX4	2.842126344	0.029488223	6.552737586	2.90E-23
ENSG00000186446	ZNF501	1.186236582	0.011448369	6.536686634	3.38E-14
ENSG00000253953	PCDHGB4	1.246423814	0.012963545	6.507977077	1.95E-18
ENSG00000115648	MLPH	0.595028378	0.005957296	6.483800409	6.74E-14
ENSG00000162069	BICDL2	2.655024377	0.028633127	6.483079812	6.04E-21
ENSG00000134198	TSPAN2	1.298902575	0.013127015	6.470270279	8.03E-14
ENSG00000153363	LINC00467	3.66374231	0.040254606	6.470096516	1.03E-22

ENSG00000065320	NTN1	1.924631446	0.021038597	6.463592136	8.18E-21
ENSG00000104044	OCA2	1.329940082	0.013568694	6.456612056	9.58E-14
ENSG00000151468	CCDC3	1.179931273	0.012153984	6.442823292	1.15E-13
ENSG00000121900	TMEM54	24.91792935	0.284310584	6.439099253	1.03E-26
ENSG00000224189	HAGLR	1.602729618	0.017519813	6.436232495	5.61E-18
ENSG00000267776	AC006116.10	0.791118689	0	6.429666703	0.002435
ENSG00000278917	AC006213.6	0.109988025	0	6.429666703	0.002435
ENSG00000236750	AC009237.9	1.324610831	0	6.429666703	0.002435
ENSG00000225868	AC016582.1	0.092112216	0	6.429666703	0.002435
ENSG00000189229	AC069277.1	0.152047621	0	6.429666703	0.002435
ENSG00000259683	AC243562.2	0.473855627	0	6.429666703	0.002435
ENSG00000284700	AL049637.2	0.494236514	0	6.429666703	0.002435
ENSG00000285933	AP003498.2	0.401432278	0	6.429666703	0.002435
ENSG00000215196	BASP1-AS1	0.156286963	0	6.429666703	0.002435
ENSG00000165617	DACT1	0.102369701	0	6.429666703	0.002435
ENSG00000140274	DUOXA2	0.17996866	0	6.429666703	0.002435
ENSG00000151617	EDNRA	0.108456809	0	6.429666703	0.002435
ENSG00000172031	EPHX4	0.204193673	0	6.429666703	0.002435
ENSG00000101448	EPPIN	0.073683866	0	6.429666703	0.002435
ENSG00000139055	ERP27	0.189464121	0	6.429666703	0.002435
ENSG00000267259	ERVE-1	0.170742927	0	6.429666703	0.002435
ENSG00000142449	FBN3	0.045450406	0	6.429666703	0.002435
ENSG00000150201	FXYD4	0.555791969	0	6.429666703	0.002435
ENSG00000100626	GALNT16	0.042551376	0	6.429666703	0.002435
ENSG00000274997	HIST1H2AH	1.187700151	0	6.429666703	0.002435
ENSG00000276903	HIST1H2AL	1.169567324	0	6.429666703	0.002435
ENSG00000146151	HMGCLL1	0.095758325	0	6.429666703	0.002435
ENSG00000232656	IDI2-AS1	0.360219403	0	6.429666703	0.002435
ENSG00000198885	ITPRIPL1	0.098804806	0	6.429666703	0.002435
ENSG00000232685	LINC00442	0.21103763	0	6.429666703	0.002435
ENSG00000258700	LINC00871	0.340726433	0	6.429666703	0.002435
ENSG00000237476	LINC01637	0.671988243	0	6.429666703	0.002435
ENSG00000260265	LINC02562	0.444956397	0	6.429666703	0.002435
ENSG00000135324	MRAP2	0.213488137	0	6.429666703	0.002435
ENSG00000156006	NAT2	0.347685294	0	6.429666703	0.002435
ENSG00000138650	PCDH10	0.045235701	0	6.429666703	0.002435
ENSG00000121440	PDZRN3	0.074908729	0	6.429666703	0.002435
ENSG00000107317	PTGDS	0.169546278	0	6.429666703	0.002435
ENSG00000250053	RARRES2P4	0.949669335	0	6.429666703	0.002435
ENSG00000164651	SP8	0.123625594	0	6.429666703	0.002435
ENSG00000258986	TMEM179	0.094323817	0	6.429666703	0.002435
ENSG00000225986	UBXN10-AS1	0.90125482	0	6.429666703	0.002435
ENSG00000182168	UNC5C	0.04301731	0	6.429666703	0.002435

ENSG00000114812	VIPR1	1.097830098	0.012124356	6.421445283	6.97E-18
ENSG00000075213	SEMA3A	0.454819761	0.004776771	6.414843988	1.64E-13
ENSG00000171827	ZNF570	2.508732634	0.028666745	6.413532085	2.54E-22
ENSG00000172889	EGFL7	162.8372305	1.928689156	6.402657047	6.37E-34
ENSG00000197696	NMB	7.809218292	0.087599065	6.398976339	9.69E-18
ENSG00000044524	EPHA3	0.626093059	0.00670709	6.38631131	2.36E-13
ENSG00000184185	KCNJ12	2.707834089	0.031876889	6.370594944	4.72E-22
ENSG00000168461	RAB31	2.670498733	0.032053793	6.342590598	7.35E-22
ENSG00000183049	CAMK1D	0.462587163	0.005108779	6.342425492	4.13E-13
ENSG00000123095	BHLHE41	1.936863599	0.022676354	6.33728995	2.38E-17
ENSG00000164292	RHOBTB3	2.89986203	0.035474001	6.333235139	3.84E-25
ENSG00000186205	1-Mar	6.260144761	0.077361196	6.332273242	8.79E-29
ENSG00000230392	AC004835.1	1.379911687	0.015398391	6.327495126	4.98E-13
ENSG00000179299	NSUN7	1.573140913	0.018935331	6.297352112	3.79E-17
ENSG00000278492	AC006213.5	1.450883707	0	6.293835811	0.004174
ENSG00000260070	AC006960.3	0.592701429	0	6.293835811	0.004174
ENSG00000235920	AC073109.1	0.427254098	0	6.293835811	0.004174
ENSG00000245748	AC097382.2	0.097175467	0	6.293835811	0.004174
ENSG00000250712	AC106872.9	0.299967342	0	6.293835811	0.004174
ENSG00000272744	AC107214.2	0.65907651	0	6.293835811	0.004174
ENSG00000226791	AC109826.1	0.19888363	0	6.293835811	0.004174
ENSG00000260978	AC126407.1	0.738258847	0	6.293835811	0.004174
ENSG00000260418	AL023284.4	1.117258042	0	6.293835811	0.004174
ENSG00000231720	AL353747.3	0.721683087	0	6.293835811	0.004174
ENSG00000267292	AP001542.2	1.147951944	0	6.293835811	0.004174
ENSG00000102010	BMX	0.14891465	0	6.293835811	0.004174
ENSG00000268079	BNIP3P30	0.727969525	0	6.293835811	0.004174
ENSG00000078898	BPIFB2	0.220155167	0	6.293835811	0.004174
ENSG00000187013	C17orf82	0.273107521	0	6.293835811	0.004174
ENSG00000224462	C4BPAP1	0.220736665	0	6.293835811	0.004174
ENSG00000179058	C9orf50	0.257934881	0	6.293835811	0.004174
ENSG00000231752	EMBP1	0.072733596	0	6.293835811	0.004174
ENSG00000153303	FRMD1	0.047478072	0	6.293835811	0.004174
ENSG00000154451	GBP5	0.051314566	0	6.293835811	0.004174
ENSG00000196565	HBG2	0.480845233	0	6.293835811	0.004174
ENSG00000232977	LINC00327	0.127940756	0	6.293835811	0.004174
ENSG00000243629	LINC00880	0.207063681	0	6.293835811	0.004174
ENSG00000260468	LINC01290	0.832379497	0	6.293835811	0.004174
ENSG00000280081	LINC01667	0.168150707	0	6.293835811	0.004174
ENSG00000223783	LINC01983	0.443111885	0	6.293835811	0.004174
ENSG00000267440	LINC02594	0.761119322	0	6.293835811	0.004174
ENSG00000259240	MIR4713HG	0.744838694	0	6.293835811	0.004174
ENSG00000253537	PCDHGA7	0.076769154	0	6.293835811	0.004174

ENSG00000176732	PFN4	0.395695556	0	6.293835811	0.004174
ENSG00000119608	PROX2	0.091514347	0	6.293835811	0.004174
ENSG00000274520	RF02246	4.802925375	0	6.293835811	0.004174
ENSG00000199572	RNA5SP174	3.541139895	0	6.293835811	0.004174
ENSG00000202474	RNA5SP283	3.511382417	0	6.293835811	0.004174
ENSG00000169413	RNASE6	0.393830827	0	6.293835811	0.004174
ENSG00000228956	SATB1-AS1	0.030940726	0	6.293835811	0.004174
ENSG00000145423	SFRP2	0.205637061	0	6.293835811	0.004174
ENSG00000198574	SH2D1B	0.149393818	0	6.293835811	0.004174
ENSG00000259096	SHLD2P2	0.169171865	0	6.293835811	0.004174
ENSG00000224397	SMIM25	0.170483275	0	6.293835811	0.004174
ENSG00000074317	SNCB	0.208302347	0	6.293835811	0.004174
ENSG00000179046	TRIML2	0.137001478	0	6.293835811	0.004174
ENSG00000233608	TWIST2	0.222499738	0	6.293835811	0.004174
ENSG00000171016	PYGO1	2.463959255	0.031175709	6.280555257	6.81E-24
ENSG00000145908	ZNF300	6.2995522	0.081184533	6.261098536	2.28E-25
ENSG00000132429	POPDC3	3.843106316	0.047595026	6.256277129	6.84E-17
ENSG00000068615	REEP1	3.566702006	0.045942308	6.254773186	1.04E-23
ENSG00000184305	CCSER1	0.686184603	0.008547474	6.247919911	7.71E-17
ENSG00000162777	DENND2D	4.955155901	0.064932906	6.237039541	3.40E-25
ENSG00000224717	AC098936.1	1.303532259	0.015515814	6.234514887	1.59E-12
ENSG00000197497	ZNF665	0.950304566	0.011976755	6.231058822	9.81E-17
ENSG00000103740	ACSBG1	0.858048746	0.010878037	6.2225538	1.11E-16
ENSG00000163888	CAMK2N2	2.730473655	0.032865728	6.218418441	1.95E-12
ENSG00000079931	MOXD1	1.326271628	0.016914083	6.213998342	1.25E-16
ENSG00000099256	PRTFDC1	1.578840561	0.019219889	6.202140376	2.38E-12
ENSG00000005108	THSD7A	2.133432292	0.028670679	6.197643129	3.42E-24
ENSG00000078804	TP53INP2	3.963095022	0.052936521	6.196773369	3.07E-22
ENSG00000184979	USP18	3.238421501	0.042050843	6.188023276	1.81E-16
ENSG00000197971	MBP	2.262677108	0.031260843	6.168917255	1.10E-26
ENSG00000247134	AC090204.1	21.13852215	0.292569425	6.145521496	6.55E-22
ENSG00000253966	AC008514.2	0.281278277	0	6.143874414	0.007243
ENSG00000280000	AC009135.2	0.212708743	0	6.143874414	0.007243
ENSG00000235959	AC009237.6	0.886955323	0	6.143874414	0.007243
ENSG00000283897	AC011416.3	0.162028891	0	6.143874414	0.007243
ENSG00000267682	AC016590.2	1.002850818	0	6.143874414	0.007243
ENSG00000275056	AC020663.3	0.344701244	0	6.143874414	0.007243
ENSG00000261002	AC036103.1	0.380636697	0	6.143874414	0.007243
ENSG00000276925	AC099778.2	1.782317805	0	6.143874414	0.007243
ENSG00000259692	AC104041.1	0.490311678	0	6.143874414	0.007243
ENSG00000250771	AC106865.1	0.114760164	0	6.143874414	0.007243
ENSG00000272154	AC244517.2	0.355117145	0	6.143874414	0.007243
ENSG00000224387	AL139246.1	0.8265254	0	6.143874414	0.007243

ENSG00000228648	AL353747.2	1.834483204	0	6.143874414	0.007243
ENSG00000229162	AL445471.1	0.458620801	0	6.143874414	0.007243
ENSG00000278849	AL513478.2	0.253415806	0	6.143874414	0.007243
ENSG00000263551	AP005328.1	0.277337063	0	6.143874414	0.007243
ENSG00000235062	BCRP5	1.48643896	0	6.143874414	0.007243
ENSG00000183784	C9orf66	0.122498064	0	6.143874414	0.007243
ENSG00000119865	CNRIP1	0.105994661	0	6.143874414	0.007243
ENSG00000158270	COLEC12	0.051235566	0	6.143874414	0.007243
ENSG00000273696	CT45A7	0.376822702	0	6.143874414	0.007243
ENSG00000095596	CYP26A1	0.126537368	0	6.143874414	0.007243
ENSG00000275945	EIF3FP1	0.466586919	0	6.143874414	0.007243
ENSG00000145242	EPHA5	0.038245607	0	6.143874414	0.007243
ENSG00000037280	FLT4	0.042484078	0	6.143874414	0.007243
ENSG00000260459	FTLP14	0.777002184	0	6.143874414	0.007243
ENSG00000236296	GUSBP5	0.189074438	0	6.143874414	0.007243
ENSG00000269466	H3.Y	0.384528688	0	6.143874414	0.007243
ENSG00000253898	LINC01419	0.247251188	0	6.143874414	0.007243
ENSG00000267123	LINC02081	0.082182923	0	6.143874414	0.007243
ENSG00000177363	LRRN4CL	0.14548126	0	6.143874414	0.007243
ENSG00000187601	MAGEH1	0.257229177	0	6.143874414	0.007243
ENSG00000107954	NEURL1	0.075805091	0	6.143874414	0.007243
ENSG00000184478	OR56A3	0.077828861	0	6.143874414	0.007243
ENSG00000231940	RPS7P3	0.648394926	0	6.143874414	0.007243
ENSG00000132026	RTBDN	0.140586563	0	6.143874414	0.007243
ENSG00000139155	SLCO1C1	0.077380464	0	6.143874414	0.007243
ENSG00000184564	SLITRK6	0.084056562	0	6.143874414	0.007243
ENSG00000101955	SRPX	0.189264749	0	6.143874414	0.007243
ENSG00000147041	SYTL5	0.078494898	0	6.143874414	0.007243
ENSG00000205116	TMEM88B	0.764368002	0	6.143874414	0.007243
ENSG00000211728	TRBV5-6	0.979346502	0	6.143874414	0.007243
ENSG00000156587	UBE2L6	0.171173899	0	6.143874414	0.007243
ENSG00000282914	Z83818.2	0.868519762	0	6.143874414	0.007243
ENSG00000235111	Z97192.3	0.126409767	0	6.143874414	0.007243
ENSG00000198153	ZNF849P	0.240146269	0	6.143874414	0.007243
ENSG00000204103	MAFB	1.970430923	0.026385571	6.143664404	3.40E-16
ENSG00000163814	CDCP1	9.253547648	0.130705289	6.141152568	4.01E-28
ENSG00000069188	SDK2	0.838805034	0.011569688	6.128271713	1.16E-18
ENSG00000166828	SCNN1G	0.988934249	0.012763936	6.1178755	5.40E-12
ENSG00000138696	BMPR1B	1.032720266	0.014183499	6.107168353	5.69E-16
ENSG00000148408	CACNA1B	0.658636993	0.009045796	6.107168353	5.69E-16
ENSG00000140479	PCSK6	3.740143451	0.054098493	6.105215402	3.67E-27
ENSG00000134569	LRP4	0.398558446	0.005206831	6.100415397	6.65E-12
ENSG00000138829	FBN2	8.844101543	0.128902594	6.100403366	4.53E-30

ENSG00000144488	ESPNL	0.631223706	0.008348214	6.082741393	8.21E-12
ENSG00000164736	SOX17	33.72096581	0.496943284	6.08238352	7.89E-29
ENSG00000242715	CCDC169	0.37827725	0.005065421	6.064848182	1.02E-11
ENSG00000183570	PCBP3	0.48345791	0.006555818	6.046730259	1.26E-11
ENSG00000100473	COCH	2.975049459	0.044418876	6.027844459	8.69E-20
ENSG00000159921	GNE	4.89766979	0.075600611	6.005041979	3.01E-24
ENSG00000124249	KCNK15	2.331239077	0.034446419	6.001765722	2.16E-15
ENSG00000135636	DYSF	1.734001534	0.026689197	5.992329154	6.92E-21
ENSG00000180318	ALX1	2.197712289	0.030977939	5.990969935	2.42E-11
ENSG00000104490	NCALD	20.13412356	0.317271568	5.98932891	5.00E-30
ENSG00000187017	ESPN	12.95276847	0.20604429	5.973365291	1.09E-28
ENSG00000123689	G0S2	9.98797726	0.155068552	5.957672872	1.33E-17
ENSG00000154277	UCHL1	2.563524586	0.039231483	5.951185483	4.34E-15
ENSG00000175591	P2RY2	11.59652146	0.187751648	5.93905221	1.07E-24
ENSG00000143318	CASQ1	1.401809699	0.020571288	5.932967522	4.74E-11
ENSG00000170837	GPR27	4.559344239	0.073172247	5.923682054	3.95E-19
ENSG00000136490	LIMD2	3.469645437	0.056602472	5.914028444	2.00E-21
ENSG00000161405	IKZF3	0.822515368	0.013418202	5.88628338	3.77E-17
ENSG00000127533	F2RL3	0.842206033	0.012888892	5.872535132	9.47E-11
ENSG00000099769	IGFALS	1.237825456	0.018943344	5.872535132	9.47E-11
ENSG00000104883	PEX11G	1.995212519	0.03053419	5.872535132	9.47E-11
ENSG00000215915	ATAD3C	2.017808487	0.033774991	5.849218666	6.46E-17
ENSG00000187621	TCL6	0.514112114	0.00847305	5.84437252	1.85E-14
ENSG00000165238	WNK2	4.473620391	0.078008168	5.838799089	1.11E-26
ENSG00000021300	PLEKHB1	3.676238725	0.063519532	5.835168394	9.94E-22
ENSG00000187815	ZFP69	3.229109649	0.055126997	5.834574122	1.52E-18
ENSG00000101335	MYL9	30.54292785	0.539505901	5.822582259	8.83E-28
ENSG00000009694	TENM1	0.803566361	0.013884343	5.817235851	1.98E-18
ENSG00000083814	ZNF671	2.93397276	0.050694362	5.817235851	1.98E-18
ENSG00000188883	KLRG2	1.274294584	0.020374657	5.809460222	1.93E-10
ENSG00000260401	AP002761.4	5.992438936	0.106547151	5.796883657	3.46E-22
ENSG00000203635	AC144450.1	3.182925865	0.054555907	5.787846244	3.95E-14
ENSG00000146090	RASGEF1C	0.93138796	0.015117569	5.787807189	2.45E-10
ENSG00000175414	ARL10	1.958180213	0.035157352	5.7851964	3.40E-22
ENSG00000116771	AGMAT	4.044831633	0.071620995	5.781921388	3.08E-18
ENSG00000112208	BAG2	8.845875006	0.161526827	5.772599236	2.34E-26
ENSG00000166394	CYB5R2	1.16664823	0.020829745	5.769955157	3.68E-18
ENSG00000166669	ATF7IP2	1.183408948	0.021012262	5.764160967	2.21E-16
ENSG00000184584	TMEM173	2.593953956	0.04650714	5.763934615	4.03E-18
ENSG00000100078	PLA2G3	8.986118443	0.165973756	5.746211957	1.77E-22
ENSG00000255690	TRIL	1.972849043	0.036282166	5.727274009	6.96E-18
ENSG00000157227	MMP14	5.232541885	0.100275811	5.693019401	4.19E-22
ENSG00000127863	TNFRSF19	11.18800745	0.218869167	5.673473695	8.60E-26

ENSG00000251322	SHANK3	1.552591531	0.030022215	5.663264953	9.74E-19
ENSG00000170500	LONRF2	2.4172162	0.048191117	5.641642284	1.23E-23
ENSG00000167476	JSRP1	11.52849872	0.229554472	5.637790293	9.77E-22
ENSG00000168389	MFSD2A	22.77499986	0.458020464	5.636289573	9.63E-27
ENSG00000162804	SNED1	3.603042105	0.072453552	5.632824779	4.47E-25
ENSG00000130707	ASS1	179.8829405	3.654431924	5.625138219	7.44E-29
ENSG00000140931	CMTM3	3.527257462	0.070760547	5.610243891	2.01E-18
ENSG00000113140	SPARC	0.501667593	0.009265809	5.601782504	1.84E-09
ENSG00000162444	RBP7	7.765008271	0.155969066	5.586378134	2.44E-15
ENSG00000163132	MSX1	216.4959392	4.518541556	5.586098048	1.58E-28
ENSG00000074211	PPP2R2C	0.490631516	0.009733229	5.577183746	5.29E-13
ENSG00000131711	MAP1B	0.350693411	0.00708838	5.550249199	7.46E-13
ENSG00000137843	PAK6	1.326624966	0.027729981	5.542677083	9.56E-17
ENSG00000092758	COL9A3	10.33199433	0.221920043	5.53839837	1.04E-24
ENSG00000105523	FAM83E	4.196719847	0.088499648	5.538268332	5.93E-18
ENSG00000169071	ROR2	1.465148153	0.030775608	5.535626777	1.06E-16
ENSG00000100311	PDGFB	1.710970332	0.035705761	5.531281898	5.25E-15
ENSG00000173898	SPTBN2	6.630547569	0.14391794	5.524872917	2.08E-25
ENSG00000227825	SLC9A7P1	1.312499812	0.027039035	5.522802212	1.06E-12
ENSG00000149639	SOGA1	6.767210098	0.147947918	5.516548706	2.33E-26
ENSG00000123892	RAB38	15.48771315	0.336105353	5.515197127	2.23E-21
ENSG00000151136	BTBD11	0.801538177	0.016947169	5.512438771	6.81E-15
ENSG00000187889	FYB2	0.928966421	0.019323601	5.508880387	1.26E-12
ENSG00000204161	TMEM273	0.547274883	0.01085693	5.498926339	5.39E-09
ENSG00000261150	EPPK1	0.88782985	0.019581418	5.483126709	2.12E-19
ENSG00000171310	CHST11	8.946669217	0.199958493	5.482267257	6.45E-25
ENSG00000106624	AEBP1	2.049215849	0.045169649	5.474429326	1.54E-17
ENSG00000114115	RBP1	1.583673934	0.034389082	5.473997713	1.01E-14
ENSG00000181885	CLDN7	63.71778206	1.437018362	5.472973025	8.25E-27
ENSG00000176771	NCKAP5	0.231849758	0.004686256	5.472024214	5.39E-09
ENSG00000205078	SYCE1L	1.780248304	0.035983217	5.472024214	5.39E-09
ENSG00000123612	ACVR1C	0.231762696	0.004868202	5.416666575	9.38E-09
ENSG00000204175	GPRIN2	1.082854669	0.022745489	5.416666575	9.38E-09
ENSG00000073282	TP63	0.285894552	0.006005248	5.416666575	9.38E-09
ENSG00000146904	EPHA1	3.602041815	0.085118973	5.388953098	1.51E-19
ENSG00000136574	GATA4	2.153695301	0.050374884	5.388867444	5.00E-17
ENSG00000182759	MAFA	0.834374017	0.017876646	5.388170279	1.24E-08
ENSG00000120278	PLEKHG1	2.196029615	0.052181607	5.385651477	6.52E-21
ENSG00000166582	CENPV	11.72314035	0.279769512	5.384106513	1.33E-22
ENSG00000140873	ADAMTS18	3.929509296	0.093843023	5.380404484	1.48E-21
ENSG00000132561	MATN2	4.032088065	0.096847949	5.37291597	8.39E-22
ENSG00000154237	LRRK1	0.949669335	0.022890233	5.360393073	2.36E-19
ENSG00000148346	LCN2	12.65591875	0.308131628	5.349293779	2.86E-20

ENSG00000131127	ZNF141	5.361399236	0.132481385	5.333334127	4.83E-22
ENSG00000121552	CSTA	1.460816924	0.032602419	5.329431396	2.21E-08
ENSG00000171291	ZNF439	0.385193324	0.00859672	5.329431396	2.21E-08
ENSG00000151692	RNF144A	3.072744409	0.076068954	5.325254771	4.18E-20
ENSG00000180787	ZFP3	2.785161624	0.068786972	5.322936464	4.86E-19
ENSG00000161267	BDH1	10.98507132	0.275042224	5.321270743	3.25E-25
ENSG00000162390	ACOT11	1.296170309	0.032078404	5.316976624	2.36E-18
ENSG00000158156	XKR8	4.600512624	0.114233268	5.312209414	2.54E-18
ENSG00000160352	ZNF714	9.27987603	0.234040971	5.309787028	1.70E-24
ENSG00000228477	AL663070.1	2.708849911	0.061742237	5.299140083	2.95E-08
ENSG00000196415	PRTN3	1.713713949	0.039060316	5.299140083	2.95E-08
ENSG00000076706	MCAM	1.199178255	0.030155701	5.284434337	2.31E-16
ENSG00000274209	ANTXRL	1.185785123	0.028870121	5.282119296	2.08E-11
ENSG00000138744	NAAA	4.713631247	0.120465903	5.280607286	3.32E-20
ENSG00000196440	ARMCX4	0.143969046	0.003352792	5.268199099	3.96E-08
ENSG00000185271	KLHL33	0.349414785	0.00813727	5.268199099	3.96E-08
ENSG00000156966	B3GNT7	1.855423326	0.047044794	5.264269827	4.54E-15
ENSG00000187699	C2orf88	2.034575892	0.052249767	5.263650346	5.30E-18
ENSG00000175352	NRIP3	1.672228808	0.04342772	5.229739302	7.34E-15
ENSG00000156711	MAPK13	10.60747791	0.286105823	5.213261176	4.96E-24
ENSG00000066056	TIE1	0.274657878	0.006687051	5.204252284	7.22E-08
ENSG00000069482	GAL	6.46067354	0.172165854	5.200828581	6.99E-16
ENSG00000134330	IAH1	4.187032303	0.113650445	5.19654314	1.32E-20
ENSG00000124097	HMGB1P1	5.38743233	0.140764534	5.180394317	5.69E-11
ENSG00000144857	BOC	0.796876457	0.021666564	5.171847513	1.06E-15
ENSG00000111319	SCNN1A	32.8537751	0.914154975	5.170621082	3.87E-25
ENSG00000137198	GMPR	1.516407487	0.040110324	5.162720313	6.99E-11
ENSG00000116663	FBXO6	7.983305335	0.222456156	5.151237758	5.28E-18
ENSG00000236830	CBR3-AS1	0.207470024	0.005291775	5.137339061	1.33E-07
ENSG00000149527	PLCH2	12.68175446	0.361227308	5.136314029	1.49E-24
ENSG00000143375	CGN	3.309488543	0.094722447	5.119265588	8.47E-20
ENSG00000158014	SLC30A2	0.931751046	0.025593552	5.108360826	1.31E-10
ENSG00000168918	INPP5D	0.197350937	0.005156448	5.10268147	1.83E-07
ENSG00000168404	MLKL	0.300984449	0.007864217	5.10268147	1.83E-07
ENSG00000128408	RIBC2	6.814596928	0.197303017	5.093690422	1.40E-17
ENSG00000125458	NT5C	3.989699309	0.113973576	5.092348569	4.87E-14
ENSG00000196507	TCEAL3	2.71922673	0.077680038	5.092348569	4.87E-14
ENSG00000154548	SRSF12	1.359772804	0.0393695	5.073000009	5.55E-14
ENSG00000100321	SYNGR1	0.673086507	0.019313874	5.072303375	1.77E-12
ENSG00000158258	CLSTN2	0.11451206	0.003066807	5.067170771	2.51E-07
ENSG00000134874	DZIP1	0.197333888	0.005284902	5.067170771	2.51E-07
ENSG00000181444	ZNF467	12.07406953	0.360200689	5.063853603	3.81E-21
ENSG00000142694	EVA1B	13.59493608	0.406601438	5.05257024	2.51E-18

ENSG00000152578	GRIA4	1.18937066	0.035515717	5.049163707	2.73E-17
ENSG00000161921	CXCL16	5.309175025	0.160211603	5.038181556	8.65E-18
ENSG00000107731	UNC5B	0.451998736	0.013086719	5.03254083	3.11E-10
ENSG00000188959	C9orf152	0.552980176	0.015189386	5.030763901	3.45E-07
ENSG00000175538	KCNE3	174.0170268	5.356479103	5.025972402	8.49E-25
ENSG00000138646	HERC5	2.156781554	0.065745248	5.016459511	1.96E-16
ENSG00000123609	NMI	1.705038508	0.051451864	5.013346891	1.24E-13
ENSG00000144369	FAM171B	2.001054027	0.061466697	5.008396692	5.02E-17
ENSG00000114646	CSPG5	13.7338804	0.430611801	4.995747539	2.62E-22
ENSG00000258982	AL133523.1	9.08928234	0.270472036	4.993082259	4.85E-10
ENSG00000111490	TBC1D30	2.271873837	0.072005022	4.972984446	4.01E-19
ENSG00000074181	NOTCH3	2.522072661	0.081006288	4.955094	1.72E-19
ENSG00000018236	CNTN1	0.390413982	0.011949579	4.952514051	7.62E-10
ENSG00000139626	ITGB7	0.652751965	0.019979077	4.952514051	7.62E-10
ENSG00000129116	PALLD	9.812185639	0.318661734	4.946691761	6.35E-23
ENSG00000172572	PDE3A	0.671711819	0.021320849	4.940483318	3.27E-13
ENSG00000072609	CHFR	2.878894838	0.094089589	4.93521415	1.05E-21
ENSG00000112619	PRPH2	1.418121817	0.045124115	4.923340836	1.14E-11
ENSG00000188681	TEKT4P2	0.651483858	0.019386367	4.9156833	9.27E-07
ENSG00000268357	VN1R81P	7.638200676	0.240661946	4.910771989	1.21E-09
ENSG00000212864	RNF208	5.95896863	0.194622269	4.907537807	3.85E-14
ENSG00000122691	TWIST1	4.686218777	0.154127818	4.906894761	8.85E-16
ENSG00000180066	C10orf91	13.78510214	0.460796844	4.899387218	6.94E-20
ENSG00000145808	ADAMTS19	1.012184551	0.033108818	4.897132809	5.79E-13
ENSG00000279662	AC131649.2	0.630688263	0.020168111	4.889439142	1.52E-09
ENSG00000204967	PCDHA4	0.208305446	0.006661179	4.889439142	1.52E-09
ENSG00000100053	CRYBB3	1.686840573	0.051629898	4.875188603	1.30E-06
ENSG00000197837	HIST4H4	0.567296655	0.017363507	4.875188603	1.30E-06
ENSG00000205363	INSYN1	0.222499738	0.006810151	4.875188603	1.30E-06
ENSG00000184988	TMEM106A	0.408859596	0.012514152	4.875188603	1.30E-06
ENSG00000213976	AC010615.1	3.055738746	0.101503678	4.874959085	7.74E-13
ENSG00000142156	COL6A1	18.77725384	0.645098333	4.86569431	1.80E-22
ENSG00000078900	TP73	0.599318161	0.019856479	4.865118143	2.33E-11
ENSG00000158220	ESYT3	0.800291844	0.027361321	4.847083209	1.14E-14
ENSG00000135083	CCNJL	0.29493477	0.009721603	4.845803134	2.44E-09
ENSG00000188060	RAB42	1.215576749	0.040693747	4.823480005	3.10E-09
ENSG00000145087	STXBP5L	0.241251137	0.008076341	4.823480005	3.10E-09
ENSG00000185442	FAM174B	3.822332408	0.134568114	4.820633955	7.34E-18
ENSG00000133401	PDZD2	1.621516981	0.057234525	4.820483801	3.43E-19
ENSG00000137648	TMPRSS4	3.028479091	0.107993057	4.807149096	1.07E-19
ENSG00000198553	KCNRG	1.619989783	0.055093073	4.800806029	3.95E-09
ENSG00000170577	SIX2	3.44895784	0.121804413	4.800321636	2.19E-14
ENSG00000138395	CDK15	0.224543214	0.007289224	4.790621007	2.59E-06

ENSG00000054219	LY75	0.156731061	0.005087875	4.790621007	2.59E-06
ENSG00000005513	SOX8	0.311128131	0.010099982	4.790621007	2.59E-06
ENSG00000058404	CAMK2B	0.471168215	0.016459047	4.788871471	5.87E-11
ENSG00000176788	BASP1	41.65351654	1.514314004	4.784019523	7.30E-22
ENSG00000140511	HAPLN3	38.34680697	1.397259766	4.781464073	2.65E-22
ENSG00000255725	TDGP1	2.107972292	0.072844788	4.777770003	5.04E-09
ENSG00000160183	TMPRSS3	0.877691947	0.031341237	4.770730602	2.99E-12
ENSG00000109255	NMU	13.71419922	0.505019838	4.752555795	2.09E-16
ENSG00000159164	SV2A	1.767046425	0.064954779	4.746526139	7.98E-15
ENSG00000262920	AC129507.4	3.095218575	0.103618338	4.746402569	3.68E-06
ENSG00000236155	AL355877.1	0.419033038	0.014027929	4.746402569	3.68E-06
ENSG00000254851	AP005018.2	1.626684214	0.054456353	4.746402569	3.68E-06
ENSG00000163071	SPATA18	0.270620203	0.009059527	4.746402569	3.68E-06
ENSG00000101972	STAG2	44.00563777	1.645743216	4.7448764	1.04E-22
ENSG00000129654	FOXJ1	4.113675576	0.15254377	4.739155324	1.98E-15
ENSG00000160097	FNDC5	0.798702468	0.028520626	4.730564214	8.28E-09
ENSG00000256802	AC022613.1	1.771605368	0.065443161	4.721874465	4.77E-12
ENSG00000250182	EEF1A1P13	18.6515331	0.708991133	4.7143338	8.44E-19
ENSG00000276573	AL442067.1	5.808739792	0.200731489	4.700785872	5.25E-06
ENSG00000131746	TNS4	0.26724757	0.009235222	4.700785872	5.25E-06
ENSG00000100593	ISM2	1.992673396	0.075851332	4.696185038	1.62E-14
ENSG00000177359	AC024940.1	0.330709621	0.012430748	4.656726656	1.77E-08
ENSG00000182379	NXPH4	25.16844592	0.999362723	4.655535553	2.76E-20
ENSG00000170324	FRMPD2	0.142596237	0.005091926	4.653679589	7.53E-06
ENSG00000168675	LDLRAD4	0.074799423	0.00267099	4.653679589	7.53E-06
ENSG00000196659	TTC30B	3.91120973	0.156007893	4.640571309	1.07E-16
ENSG00000112852	PCDHB2	4.324378932	0.173584574	4.632915281	3.57E-17
ENSG00000128274	A4GALT	2.293477925	0.091970019	4.621078205	4.63E-14
ENSG00000163520	FBLN2	0.937828072	0.037209578	4.618899772	1.73E-11
ENSG00000131459	GFPT2	5.481062265	0.224752077	4.605654001	2.31E-18
ENSG00000172379	ARNT2	0.437233111	0.017347806	4.605434043	4.13E-10
ENSG00000196872	KIAA1211L	0.488459042	0.019027895	4.605314906	2.98E-08
ENSG00000257446	ZNF878	1.325374736	0.051629898	4.605314906	2.98E-08
ENSG00000205642	VCX3B	1.031300487	0.038096274	4.604983144	1.09E-05
ENSG00000176532	PRR15	4.975462351	0.20326342	4.599503374	1.35E-14
ENSG00000122592	HOXA7	3.63954569	0.148529646	4.59874013	1.49E-14
ENSG00000140807	NKD1	0.810529834	0.033395729	4.592882951	4.28E-16
ENSG00000121690	DEPDC7	1.33170709	0.053834182	4.591965225	2.41E-11
ENSG00000111058	ACSS3	2.331640424	0.09703215	4.58591357	5.72E-19
ENSG00000167363	FN3K	3.906594312	0.161850131	4.585847495	2.30E-16
ENSG00000144015	TRIM43	1.341506743	0.053226067	4.578905259	3.88E-08
ENSG00000275183	LENG9	2.835129749	0.116813993	4.572650384	2.94E-12
ENSG00000248144	ADH1C	0.629028291	0.024066195	4.554585416	1.58E-05

ENSG00000204128	C2orf72	0.303815274	0.011623766	4.554585416	1.58E-05
ENSG00000108375	RNF43	0.851855292	0.03578664	4.550108663	5.95E-13
ENSG00000115457	IGFBP2	45.19525574	1.934382866	4.549972678	3.54E-21
ENSG00000163577	EIF5A2	5.065355912	0.216092311	4.549330152	2.46E-18
ENSG00000174307	PHLDA3	2.024024383	0.085589143	4.540656336	6.75E-13
ENSG00000121207	LRAT	0.563622848	0.023677928	4.536538935	4.75E-11
ENSG00000143217	NECTIN4	0.918754914	0.038346478	4.532489839	9.42E-10
ENSG00000106633	GCK	0.527133294	0.021719128	4.524589814	5.07E-08
ENSG00000235863	B3GALT4	1.791311768	0.075998509	4.522343132	5.64E-11
ENSG00000186469	GNG2	0.661597512	0.028069053	4.522343132	5.64E-11
ENSG00000166813	KIF7	1.044427383	0.044754171	4.516124107	6.09E-12
ENSG00000100197	CYP2D6	1.299915783	0.05496904	4.513662586	1.16E-09
ENSG00000170745	KCNS3	1.988512348	0.085780496	4.511921994	9.89E-13
ENSG00000125841	NRSN2	2.122752186	0.091571337	4.511921994	9.89E-13
ENSG00000254165	AC090739.1	1.593512953	0.063224749	4.502363216	2.30E-05
ENSG00000123119	NECAB1	0.176062293	0.006985506	4.502363216	2.30E-05
ENSG00000156535	CD109	2.486110442	0.109825693	4.498230675	1.14E-17
ENSG00000165591	FAAH2	10.03761447	0.443418742	4.4968492	4.40E-17
ENSG00000198435	NRARP	55.78475714	2.478410142	4.495241191	3.48E-20
ENSG00000169981	ZNF35	5.319289451	0.235329969	4.491177967	9.40E-16
ENSG00000128641	MYO1B	7.670674376	0.342778549	4.486280749	8.73E-20
ENSG00000106605	BLVRA	13.53839426	0.614540389	4.459317716	1.58E-17
ENSG00000162782	TDRD5	0.255268261	0.010517651	4.448179468	3.37E-05
ENSG00000182040	USH1G	0.305346183	0.012580979	4.448179468	3.37E-05
ENSG00000127831	VIL1	0.156997358	0.006468659	4.448179468	3.37E-05
ENSG00000129757	CDKN1C	4.36740587	0.198706353	4.447573012	1.55E-14
ENSG00000159184	HOXB13	2.832299085	0.129111976	4.443291081	4.03E-14
ENSG00000183196	CHST6	3.945865072	0.181752815	4.440102981	2.64E-18
ENSG00000180535	BHLHA15	4.974458424	0.224376551	4.434103576	1.63E-10
ENSG00000047457	CP	1.592153963	0.073623556	4.425433357	9.61E-15
ENSG00000198626	RYR2	0.597035748	0.027698394	4.41946356	2.31E-14
ENSG00000050030	NEXMIF	0.168986573	0.007542863	4.409410316	1.52E-07
ENSG00000085563	ABCB1	0.669315694	0.030839216	4.403448884	2.34E-10
ENSG00000147036	LANCL3	0.101440694	0.004346778	4.391881045	4.96E-05
ENSG00000204710	SPDYC	1.054123379	0.045169649	4.391881045	4.96E-05
ENSG00000168032	ENTPD3	0.797785308	0.037158098	4.374507713	5.33E-09
ENSG00000184828	ZBTB7C	0.376986938	0.017558756	4.374507713	5.33E-09
ENSG00000196268	ZNF493	1.736402716	0.083826071	4.369233576	2.05E-16
ENSG00000110723	EXPH5	4.077193295	0.198158245	4.363955298	2.51E-18
ENSG00000165568	AKR1E2	1.175593981	0.05556035	4.353485719	6.68E-09
ENSG00000136859	ANGPTL2	0.730627578	0.034530565	4.353485719	6.68E-09
ENSG00000182218	HHIPL1	0.362609833	0.017137489	4.353485719	6.68E-09
ENSG00000094755	GABRP	0.41595558	0.019373781	4.348178019	2.67E-07

ENSG00000109472	CPE	64.69823004	3.193042168	4.344493946	8.62E-20
ENSG00000167608	TMC4	6.222784306	0.306997244	4.337946208	3.14E-16
ENSG00000279970	AC023024.2	0.503186562	0.022460172	4.333296156	7.35E-05
ENSG00000174990	CA5A	0.123200346	0.005499155	4.333296156	7.35E-05
ENSG00000226453	LINC02542	1.220013161	0.054456353	4.333296156	7.35E-05
ENSG00000122574	WIPF3	0.165350506	0.007380564	4.333296156	7.35E-05
ENSG00000091428	RAPGEF4	0.392709384	0.018837055	4.332152872	8.38E-09
ENSG00000157992	KRTCAP3	9.619886383	0.478579351	4.322742072	5.51E-15
ENSG00000105810	CDK6	9.669276003	0.485603199	4.31886981	2.74E-19
ENSG00000185306	C12orf56	0.827509835	0.040664173	4.318742628	6.16E-11
ENSG00000259803	SLC22A31	0.720438806	0.034301243	4.316558884	3.56E-07
ENSG00000169964	TMEM42	3.161487742	0.158530052	4.307355077	1.04E-13
ENSG00000189334	S100A14	37.17170874	1.892880647	4.297044202	4.04E-18
ENSG00000162676	GFI1	2.683585447	0.135604732	4.294780527	3.20E-13
ENSG00000005961	ITGA2B	0.647369621	0.032255898	4.290717608	8.72E-10
ENSG00000230445	LRRC37A6P	0.326855081	0.015915777	4.284231203	4.77E-07
ENSG00000196549	MME	0.191118486	0.009306262	4.284231203	4.77E-07
ENSG00000101417	PXMP4	14.90199284	0.766518601	4.283910179	9.33E-19
ENSG00000226054	MEMO1P1	5.976072349	0.302453527	4.2816545	1.71E-11
ENSG00000164099	PRSS12	0.773669481	0.03909443	4.278527062	1.00E-10
ENSG00000223572	CKMT1A	0.309123631	0.014397916	4.272231189	0.00011
ENSG00000169429	CXCL8	0.422632088	0.01968475	4.272231189	0.00011
ENSG00000197061	HIST1H4C	2.209345672	0.102903729	4.272231189	0.00011
ENSG00000189431	RASSF10	0.379867734	0.017692934	4.272231189	0.00011
ENSG00000257591	ZNF625	0.436055067	0.020309946	4.272231189	0.00011
ENSG00000140848	CPNE2	7.652521184	0.396948389	4.271073847	3.01E-18
ENSG00000167895	TMC8	4.722773973	0.245996274	4.261982681	9.39E-17
ENSG00000171243	SOSTDC1	5.221069262	0.271511051	4.258840861	1.39E-14
ENSG00000249790	AC092490.1	0.788404731	0.039283126	4.251162493	6.40E-07
ENSG00000248774	AC097534.1	3.44870323	0.171835401	4.251162493	6.40E-07
ENSG00000166446	CDYL2	0.209977139	0.01046234	4.251162493	6.40E-07
ENSG00000157833	GAREM2	0.302538202	0.015074296	4.251162493	6.40E-07
ENSG00000188906	LRRK2	0.133183188	0.006635998	4.251162493	6.40E-07
ENSG00000065989	PDE4A	2.187983278	0.114500914	4.251039235	5.55E-15
ENSG00000171124	FUT3	0.846729945	0.043193749	4.24351976	2.12E-08
ENSG00000223652	AC106786.1	5.858156332	0.307197896	4.234361307	6.54E-12
ENSG00000197479	PCDHB11	2.209711784	0.117292602	4.225300383	3.24E-13
ENSG00000164932	CTHRC1	1.458726322	0.075613382	4.220483733	2.69E-08
ENSG00000139597	N4BP2L1	0.289937045	0.014790392	4.21731798	8.61E-07
ENSG00000197744	PTMAP2	96.22154257	5.201830687	4.209490208	8.38E-17
ENSG00000107099	DOCK8	0.066691811	0.00324747	4.208466847	0.000164
ENSG00000162669	HFM1	0.157762127	0.007682019	4.208466847	0.000164
ENSG00000249839	AC011330.1	1.033334176	0.055348528	4.186543129	2.33E-09

ENSG00000186889	TMEM17	0.840753536	0.045033322	4.173277945	4.35E-08
ENSG00000176170	SPHK1	0.747520519	0.040546306	4.168425205	2.84E-09
ENSG00000126778	SIX1	2.655724828	0.148004444	4.160830975	1.28E-14
ENSG00000183779	ZNF703	35.65924702	2.004917377	4.156121337	2.55E-18
ENSG00000283429	MIR1244-3	38.34429599	2.106499857	4.150076853	3.48E-09
ENSG00000198944	SOWAHA	1.015031193	0.05576222	4.150076853	3.48E-09
ENSG00000166444	ST5	6.114586715	0.345249153	4.149606646	5.21E-18
ENSG00000213145	CRIP1	0.992089173	0.054039986	4.149082899	5.56E-08
ENSG00000186019	AC021092.1	0.479190949	0.025666928	4.14714969	1.58E-06
ENSG00000165309	ARMC3	0.125374263	0.006395645	4.141753396	0.000248
ENSG00000183813	CCR4	0.283520021	0.014463044	4.141753396	0.000248
ENSG00000232046	LINC01798	0.546045094	0.027855085	4.141753396	0.000248
ENSG00000136274	NACAD	0.178461352	0.009103747	4.141753396	0.000248
ENSG00000189212	DPY19L2P1	0.388960313	0.02164561	4.131492135	4.27E-09
ENSG00000132386	SERPINF1	11.72279997	0.670936334	4.127984052	1.06E-16
ENSG00000259439	LINC01833	1.21946176	0.069364342	4.113330281	1.19E-10
ENSG00000187391	MAGI2	0.051686783	0.002839489	4.110742821	2.15E-06
ENSG00000120324	PCDHB10	0.495329052	0.027211624	4.110742821	2.15E-06
ENSG00000197935	ZNF311	0.513108495	0.028188364	4.110742821	2.15E-06
ENSG00000165810	BTNL9	0.42799114	0.024131063	4.099440386	9.12E-08
ENSG00000188613	NANOS1	0.780161515	0.044573684	4.09358868	6.45E-09
ENSG00000121413	ZSCAN18	0.925547483	0.053886026	4.090555636	4.61E-12
ENSG00000189409	MMP23B	1.709655486	0.098467052	4.09001072	9.37E-10
ENSG00000131979	GCH1	13.15441672	0.773608682	4.089140998	1.20E-16
ENSG00000128683	GAD1	0.562749943	0.032586597	4.08759685	1.63E-10
ENSG00000178078	STAP2	12.35737481	0.728250357	4.085707851	2.22E-16
ENSG00000124126	PREX1	14.65571132	0.865836121	4.084634768	8.46E-18
ENSG00000113580	NR3C1	1.73118963	0.102488613	4.074993468	1.39E-14
ENSG00000225067	RPL23AP2	6.635457846	0.384232807	4.074256857	7.95E-09
ENSG00000273523	AL139082.1	0.681615276	0.039117205	4.073963512	1.17E-07
ENSG00000165028	NIPSNAP3B	0.729039086	0.041104795	4.073393375	2.93E-06
ENSG00000138075	ABCG5	0.224652961	0.012033097	4.07180476	0.000377
ENSG00000138670	RASGEF1B	0.816586929	0.048354546	4.067606046	2.53E-12
ENSG00000184226	PCDH9	3.315126733	0.198182126	4.067525507	1.22E-17
ENSG00000231806	PCAT7	2.675288006	0.159218422	4.067382877	1.55E-14
ENSG00000198121	LPAR1	3.586777091	0.215259062	4.054926177	2.56E-14
ENSG00000111859	NEDD9	4.719468808	0.285197997	4.050664586	7.52E-17
ENSG00000275496	CU633906.1	2.07620551	0.124428414	4.044781192	2.04E-11
ENSG00000274267	HIST1H3B	3.761707246	0.217825411	4.035051245	4.01E-06
ENSG00000167676	PLIN4	1.021822004	0.061960643	4.030125223	9.28E-12
ENSG00000008735	MAPK8IP2	2.726215649	0.166885051	4.02710735	1.97E-14
ENSG00000121310	ECHDC2	4.578633906	0.282380565	4.021521026	1.07E-16
ENSG00000100100	PIK3IP1	4.95275645	0.30587153	4.015511006	7.83E-15

ENSG00000272320	AL445309.1	0.399157147	0.02250534	3.998291102	0.000575
ENSG00000238186	AL603839.2	1.454072462	0.08198374	3.998291102	0.000575
ENSG00000212712	AP002414.1	0.692173988	0.039026262	3.998291102	0.000575
ENSG00000018625	ATP1A2	0.126059632	0.007107514	3.998291102	0.000575
ENSG00000204882	GPR20	0.524041957	0.029546615	3.998291102	0.000575
ENSG00000174498	IGDCC3	0.14299776	0.008062522	3.998291102	0.000575
ENSG00000170965	PLAC1	0.414365117	0.023362799	3.998291102	0.000575
ENSG00000253731	PCDHGA6	0.279978368	0.016977164	3.994716865	2.53E-07
ENSG00000172818	OVOL1	0.759932853	0.046519223	3.994230078	1.87E-08
ENSG00000175318	GRAMD2A	1.441166539	0.089759619	3.993329007	1.49E-11
ENSG00000188921	HACD4	1.931298697	0.122276885	3.979385026	1.66E-14
ENSG00000134824	FADS2	34.13779616	2.176041481	3.975844199	1.27E-17
ENSG00000100767	PAPLN	0.830390176	0.052327328	3.97467601	1.90E-11
ENSG00000135378	PRRG4	7.238781524	0.461392035	3.973376504	4.21E-16
ENSG00000030419	IKZF2	1.891269585	0.120684888	3.969655476	3.92E-15
ENSG00000115850	LCT	0.32991853	0.020390125	3.967303545	2.53E-07
ENSG00000124191	TOX2	0.674167992	0.041665953	3.967303545	2.53E-07
ENSG00000010030	ETV7	0.616044977	0.037711139	3.955167523	7.63E-06
ENSG00000054179	ENTPD2	3.967475385	0.255012089	3.953304305	9.00E-13
ENSG00000158050	DUSP2	2.16280801	0.139015907	3.937221526	9.72E-10
ENSG00000253846	PCDHGA10	1.648018681	0.107814233	3.928520619	7.41E-13
ENSG00000267260	AC020928.1	0.366896641	0.021835669	3.920829424	0.000575
ENSG00000183831	ANKRD45	0.258377916	0.015377232	3.920829424	0.000575
ENSG00000163508	EOMES	0.205727055	0.012243742	3.920829424	0.000575
ENSG00000140795	MYLK3	0.07898951	0.004701021	3.920829424	0.000575
ENSG00000095627	TDRD1	0.143100859	0.008516576	3.920829424	0.000575
ENSG00000206538	VGLL3	0.05915826	0.003520774	3.920829424	0.000575
ENSG00000219665	ZNF433-AS1	1.837424745	0.121597615	3.911914135	9.32E-13
ENSG00000135547	HEY2	0.762230039	0.048992837	3.910862929	4.27E-07
ENSG00000160111	CPAMD8	0.331858173	0.021944858	3.890998176	7.31E-09
ENSG00000128606	LRRC17	1.015727113	0.066960541	3.887519161	5.64E-08
ENSG00000181392	SYNE4	3.921505237	0.2647661	3.882375328	2.19E-12
ENSG00000105376	ICAM5	1.198149126	0.080220649	3.882205034	4.51E-10
ENSG00000229167	AC114488.1	3.464445156	0.227223969	3.881792409	5.57E-07
ENSG00000114698	PLSCR4	0.484383035	0.031769427	3.881792409	5.57E-07
ENSG00000100290	BIK	5.568470353	0.375765977	3.873758479	1.73E-10
ENSG00000183971	NPW	13.48725594	0.919790177	3.871950017	9.88E-14
ENSG00000204335	SP5	0.66937858	0.043459342	3.870599927	1.47E-05
ENSG00000151882	CCL28	1.02523984	0.069262125	3.869280943	5.27E-10
ENSG00000267128	RNF157-AS1	2.87355049	0.196071493	3.86178838	8.06E-11
ENSG00000234773	AC012618.3	0.638758483	0.042767314	3.852124046	7.30E-07
ENSG00000245848	CEBPA	0.762334335	0.051041188	3.852124046	7.30E-07
ENSG00000183307	TMEM121B	0.395679944	0.02649228	3.852124046	7.30E-07

ENSG00000131242	RAB11FIP4	8.208778014	0.57034206	3.850997094	1.90E-16
ENSG00000221923	ZNF880	2.606675067	0.180544993	3.845558789	3.61E-12
ENSG00000152217	SETBP1	1.328792231	0.092558327	3.842581554	4.76E-14
ENSG00000125780	TGM3	0.977528183	0.066488113	3.842522056	8.90E-08
ENSG00000198515	CNGA1	0.198367122	0.012500174	3.838971485	0.000885
ENSG00000109832	DDX25	0.063611772	0.004008518	3.838971485	0.000885
ENSG00000185269	NOTUM	0.235294025	0.014827135	3.838971485	0.000885
ENSG00000114638	UPK1B	0.313483082	0.019754246	3.838971485	0.000885
ENSG00000184669	OR7E14P	1.801694394	0.123723389	3.836638689	1.32E-08
ENSG00000127084	FGD3	0.986108261	0.068398658	3.836351744	9.71E-11
ENSG00000118596	SLC16A7	0.795039678	0.055687748	3.830644625	1.75E-12
ENSG00000124613	ZNF391	2.247459081	0.157420992	3.830644625	1.75E-12
ENSG00000261104	AC093904.4	0.408534807	0.027352962	3.826381489	2.06E-05
ENSG00000174460	ZCCHC12	2.865609129	0.200641515	3.824586464	1.29E-10
ENSG00000112559	MDFI	14.30940525	1.011295264	3.82440331	3.74E-15
ENSG00000175985	PLEKHD1	1.723643393	0.121623896	3.822170851	3.28E-13
ENSG00000260400	AL513534.1	0.8557369	0.058513885	3.821832734	9.57E-07
ENSG00000211445	GPX3	6.975250711	0.495323828	3.815736033	2.59E-14
ENSG00000144407	PTH2R	1.589560427	0.11216272	3.814864937	6.24E-11
ENSG00000196387	ZNF140	7.08346588	0.508103915	3.80316485	3.89E-15
ENSG00000267254	ZNF790-AS1	2.941074223	0.20806232	3.802864588	1.15E-09
ENSG00000176920	FUT2	6.613969277	0.476766979	3.794228352	3.02E-14
ENSG00000112562	SMOC2	4.155745475	0.299753049	3.791836832	1.49E-13
ENSG00000181104	F2R	0.472732596	0.03302739	3.79089175	1.26E-06
ENSG00000177614	PGBD5	0.661676901	0.047519238	3.790678395	4.01E-11
ENSG00000214546	AC087491.1	2.102839243	0.145334812	3.780764792	2.88E-05
ENSG00000158292	GPR153	1.218145184	0.087727824	3.779990046	5.43E-10
ENSG00000181350	LRRC75A	2.657532051	0.193832193	3.771696199	5.80E-12
ENSG00000226887	ERVMER34-1	2.705753986	0.197629678	3.770763998	2.56E-12
ENSG00000127129	EDN2	1.147251546	0.081933719	3.759272614	1.66E-06
ENSG00000197977	ELOVL2	0.47043915	0.03359754	3.759272614	1.66E-06
ENSG00000197619	ZNF615	5.169861813	0.383840572	3.752264033	3.84E-14
ENSG00000267980	AC007292.1	0.896202697	0.060004185	3.75218809	0.00137
ENSG00000259408	AC010809.2	0.693534452	0.046434774	3.75218809	0.00137
ENSG00000090539	CHRD	0.100992026	0.006761801	3.75218809	0.00137
ENSG00000149970	CNKSR2	0.02098124	0.001404774	3.75218809	0.00137
ENSG00000223839	FAM95B1	0.084383089	0.005649769	3.75218809	0.00137
ENSG00000171126	KCNG3	0.174834522	0.011705837	3.75218809	0.00137
ENSG00000130822	PNCK	0.131323357	0.008792599	3.75218809	0.00137
ENSG00000171056	SOX7	0.766586317	0.055675525	3.748085196	2.27E-07
ENSG00000117461	PIK3R3	19.80438638	1.480022251	3.746027463	6.79E-16
ENSG00000183508	TENT5C	0.632122103	0.046701223	3.736586761	9.53E-09
ENSG00000256417	AC006206.2	0.781523393	0.055814367	3.733658509	4.06E-05

ENSG00000260001	TGFBR3L	0.826888867	0.059054251	3.733658509	4.06E-05
ENSG00000139946	PELI2	2.369483987	0.17825391	3.730687446	5.26E-13
ENSG00000019582	CD74	2.427860675	0.182764003	3.723840468	5.35E-11
ENSG00000133687	TMTC1	0.165588695	0.012233704	3.723477451	2.88E-07
ENSG00000141526	SLC16A3	26.0607425	1.980625074	3.722002438	7.08E-16
ENSG00000121653	MAPK8IP1	6.570039427	0.499074259	3.719005856	8.69E-14
ENSG00000179403	VWA1	39.82198831	3.040180445	3.715424975	8.53E-16
ENSG00000133019	CHRM3	1.345924133	0.102988284	3.706861191	3.76E-13
ENSG00000277662	AL354696.1	3.615240206	0.270199931	3.693876223	2.92E-06
ENSG00000060140	STYK1	3.221775409	0.248393492	3.693268164	4.90E-12
ENSG00000116985	BMP8B	2.643003418	0.204228263	3.691184993	2.04E-12
ENSG00000023892	DEF6	1.60902718	0.123120571	3.689797236	4.25E-09
ENSG00000278811	LINC00624	2.906356837	0.225263734	3.688347239	4.88E-13
ENSG00000100979	PLTP	22.11783993	1.726401398	3.682287389	7.67E-15
ENSG00000278356	AC005911.1	5.131546584	0.392658965	3.672965809	4.69E-07
ENSG00000122694	GLIPR2	1.135251199	0.087914478	3.668785715	1.66E-08
ENSG00000120162	MOB3B	1.285343342	0.10105968	3.663425349	2.45E-11
ENSG00000204956	PCDHGA1	0.280708402	0.021479425	3.660031711	3.89E-06
ENSG00000260118	AL157700.1	0.437696761	0.031259163	3.659848339	0.002134
ENSG00000273056	AL354694.1	1.073256441	0.076649181	3.659848339	0.002134
ENSG00000172014	ANKRD20A4	0.138976	0.009925304	3.659848339	0.002134
ENSG00000125999	BPIFB1	0.202122464	0.01443506	3.659848339	0.002134
ENSG00000159231	CBR3	0.536169171	0.038291807	3.659848339	0.002134
ENSG00000102879	CORO1A	0.128676198	0.009189719	3.659848339	0.002134
ENSG00000237989	LINC01679	0.224010637	0.015998257	3.659848339	0.002134
ENSG00000183793	NPIPA5	0.412356422	0.029449422	3.659848339	0.002134
ENSG00000255298	OR8G5	0.374421602	0.026740216	3.659848339	0.002134
ENSG00000240322	RN7SL481P	2.096260071	0.149709438	3.659848339	0.002134
ENSG00000138031	ADCY3	5.966394009	0.472901916	3.659295907	2.64E-14
ENSG00000061455	PRDM6	1.090443849	0.086174994	3.657165017	1.19E-11
ENSG00000198780	FAM169A	1.56543161	0.123979492	3.654517801	8.27E-12
ENSG00000273759	AL117379.1	9.196502456	0.727318082	3.65046379	4.91E-10
ENSG00000215018	COL28A1	0.347212538	0.02705129	3.647030933	5.99E-07
ENSG00000285875	AL035446.2	0.947362446	0.072490886	3.634564336	8.17E-05
ENSG00000284128	AP000356.3	0.729876869	0.055849185	3.634564336	8.17E-05
ENSG00000248019	FAM13A-AS1	0.259421867	0.019850608	3.634564336	8.17E-05
ENSG00000074660	SCARF1	0.235838061	0.018046008	3.634564336	8.17E-05
ENSG00000083290	ULK2	2.254198349	0.181908451	3.631883801	2.66E-13
ENSG00000198719	DLL1	0.699446479	0.055502941	3.620621286	7.68E-07
ENSG00000133135	RNF128	0.657463386	0.052171471	3.620621286	7.68E-07
ENSG00000214182	PTMAP5	79.79510777	6.510299149	3.619113736	7.03E-15
ENSG00000204282	TNRC6C-AS1	1.25299398	0.101688146	3.611786236	1.55E-09
ENSG00000189143	CLDN4	38.98612771	3.198725857	3.611471989	3.85E-15

ENSG00000103184	SEC14L5	0.761260335	0.062199799	3.60029195	1.71E-09
ENSG00000118513	MYB	0.798738818	0.065192968	3.59962625	3.90E-09
ENSG00000259459	LINC02568	3.897477021	0.321170382	3.595721794	5.44E-11
ENSG00000121743	GJA3	0.320748039	0.025770364	3.589863421	6.96E-06
ENSG00000168505	GBX2	3.267026604	0.270705705	3.586361742	1.71E-10
ENSG00000092068	SLC7A8	0.63469805	0.052302095	3.58302803	1.41E-08
ENSG00000224184	MIR3681HG	0.291075121	0.023097586	3.582342136	0.000117
ENSG00000153404	PLEKHG4B	1.890772802	0.158137908	3.580880924	2.59E-13
ENSG00000174292	TNK1	4.297622785	0.359496575	3.57889953	1.23E-12
ENSG00000112319	EYA4	2.366972456	0.199657101	3.568016314	6.35E-13
ENSG00000213853	EMP2	39.45604848	3.342600642	3.565515232	5.52E-15
ENSG00000116667	C1orf21	2.067835346	0.174776559	3.565385833	4.69E-13
ENSG00000008323	PLEKHG6	1.87347913	0.157616627	3.565178851	1.31E-10
ENSG00000061656	SPAG4	3.562955846	0.299753049	3.565178851	1.31E-10
ENSG00000256894	AC022509.3	0.708227979	0.054192642	3.561191766	0.003346
ENSG00000223723	BX842568.2	0.475606757	0.036392782	3.561191766	0.003346
ENSG00000140481	CCDC33	0.082778592	0.006334105	3.561191766	0.003346
ENSG00000048540	LMO3	0.056972761	0.004359478	3.561191766	0.003346
ENSG00000184497	TMEM255B	0.080279444	0.006142874	3.561191766	0.003346
ENSG00000159618	ADGRG5	0.728317836	0.061193605	3.557858182	6.27E-09
ENSG00000157214	STEAP2	0.426724626	0.035954315	3.55101275	2.00E-08
ENSG00000178460	MCMDC2	0.499794501	0.042269237	3.541880151	6.38E-08
ENSG00000179178	TMEM125	6.7776119	0.589265357	3.522668605	4.64E-12
ENSG00000072201	LNX1	1.262686984	0.109464403	3.521191633	3.51E-10
ENSG00000278385	AC121338.2	1.197977373	0.102667711	3.509865225	1.64E-06
ENSG00000050555	LAMC3	0.275121482	0.023578152	3.509865225	1.64E-06
ENSG00000178750	STX19	1.804207718	0.154622183	3.509865225	1.64E-06
ENSG00000160180	TFF3	2.647365887	0.229947544	3.503472126	9.50E-08
ENSG00000160229	ZNF66	1.194266788	0.10413488	3.50161697	3.43E-08
ENSG00000164002	EXO5	3.108232107	0.275064462	3.494468335	6.38E-11
ENSG00000178662	CSRNP3	0.804781463	0.071250493	3.494256968	4.86E-11
ENSG00000112394	SLC16A10	1.253154481	0.111539327	3.488833646	7.38E-12
ENSG00000269313	MAGIX	0.597285668	0.052590314	3.483877738	1.16E-07
ENSG00000130584	ZBTB46	5.913424244	0.529741088	3.482987279	2.71E-13
ENSG00000230612	AC004039.1	0.864204404	0.075063927	3.477764975	1.70E-05
ENSG00000130600	H19	0.533676796	0.046354631	3.477764975	1.70E-05
ENSG00000274827	LINC01297	0.35026318	0.030423508	3.477764975	1.70E-05
ENSG00000167912	AC090152.1	0.59933234	0.051363307	3.471859965	0.000241
ENSG00000167311	ART5	0.61776243	0.052942782	3.471859965	0.000241
ENSG00000224843	LINC00240	0.161184427	0.013813647	3.471859965	0.000241
ENSG00000177839	PCDHB9	0.236610706	0.020277745	3.471859965	0.000241
ENSG00000274386	TMEM269	0.238883208	0.0204725	3.471859965	0.000241
ENSG00000023902	PLEKHO1	2.265898015	0.20455349	3.468810688	6.48E-12

ENSG00000124935	SCGB1D2	7.312453883	0.652795529	3.467721126	4.11E-08
ENSG00000158555	GDPD5	11.34944847	1.034356117	3.459678954	5.02E-14
ENSG00000260249	AC007608.3	0.102978362	0.0084859	3.455290244	0.005282
ENSG00000274677	AC040169.3	0.887599444	0.073142356	3.455290244	0.005282
ENSG00000176029	C11orf16	0.208366268	0.017170357	3.455290244	0.005282
ENSG00000102287	GABRE	0.056513822	0.004657004	3.455290244	0.005282
ENSG00000256356	HSPA8P5	0.26254754	0.021635148	3.455290244	0.005282
ENSG00000263503	MAPK8IP1P2	0.369029117	0.03040973	3.455290244	0.005282
ENSG00000167034	NKX3-1	0.166068743	0.013684843	3.455290244	0.005282
ENSG00000243431	RPL5P30	0.609664265	0.050239194	3.455290244	0.005282
ENSG00000085741	WNT11	0.22624359	0.018643533	3.455290244	0.005282
ENSG00000138336	TET1	1.741060448	0.1590421	3.452248105	5.04E-12
ENSG00000245017	LINC02453	1.045725566	0.093353748	3.451126342	2.75E-06
ENSG00000228526	MIR34AHG	0.172865574	0.015432012	3.438375951	2.31E-05
ENSG00000140470	ADAMTS17	0.709774703	0.065173194	3.436435261	2.88E-09
ENSG00000261659	Z92544.2	0.693680809	0.062975692	3.434647032	7.74E-07
ENSG00000185112	FAM43A	2.513158626	0.233328222	3.421512433	2.10E-09
ENSG00000246575	AC093162.2	2.789653673	0.25433592	3.42083503	3.58E-06
ENSG00000196724	ZNF418	0.965493641	0.089506354	3.418300855	1.20E-08
ENSG00000140403	DNAJA4	2.605647134	0.244495887	3.413932862	5.65E-12
ENSG00000140993	TIGD7	1.254459565	0.117320548	3.408803161	7.80E-09
ENSG00000285813	AP000813.1	1.263538829	0.118735072	3.400504691	1.57E-08
ENSG00000159905	ZNF221	1.56868457	0.148276812	3.390321551	1.65E-08
ENSG00000149212	SESN3	4.031132108	0.385268523	3.390072961	5.05E-13
ENSG00000119888	EPCAM	174.2415144	16.72175378	3.385679648	6.02E-14
ENSG00000134955	SLC37A2	0.373845659	0.035130374	3.385004519	1.23E-06
ENSG00000152503	TRIM36	9.754267664	0.937294227	3.382785117	3.33E-13
ENSG00000131981	LGALS3	7.548227025	0.728311121	3.374032743	9.99E-12
ENSG00000232677	LINC00665	13.57258288	1.317842224	3.368259803	1.91E-13
ENSG00000151364	KCTD14	0.980879126	0.093402445	3.358274911	6.09E-06
ENSG00000066230	SLC9A3	4.471451075	0.436907135	3.357042561	3.02E-12
ENSG00000055118	KCNH2	0.241739889	0.022849986	3.356217012	4.30E-05
ENSG00000197165	SULT1A2	11.46115221	1.121537561	3.355000925	2.76E-12
ENSG00000136002	ARHGEF4	0.563125008	0.054841258	3.352625809	4.25E-09
ENSG00000101825	MXRA5	0.610162306	0.059422096	3.352625809	4.25E-09
ENSG00000071073	MGAT4A	10.13177856	0.994736044	3.35225178	2.37E-13
ENSG00000283108	AC011451.3	1.027877398	0.095749993	3.352210109	0.000507
ENSG00000276966	HIST1H4E	0.646311612	0.060205947	3.352210109	0.000507
ENSG00000181690	PLAG1	1.925768891	0.188584581	3.351948027	1.88E-11
ENSG00000135540	NHSL1	4.264114183	0.419926324	3.346678188	1.21E-12
ENSG00000197594	ENPP1	2.735627462	0.269684438	3.344466145	2.67E-12
ENSG00000170629	DPY19L2P2	1.145730101	0.112308908	3.344091872	3.07E-09
ENSG00000269318	AC007292.2	0.276724839	0.02470371	3.340994755	0.00839

ENSG00000261655	AC100803.2	0.255438313	0.022803424	3.340994755	0.00839
ENSG00000278949	AC127070.4	0.238320061	0.021275248	3.340994755	0.00839
ENSG00000283765	AC131160.1	0.163971684	0.014638039	3.340994755	0.00839
ENSG00000230736	AL021937.1	0.321839159	0.028731144	3.340994755	0.00839
ENSG00000279110	AL022323.4	0.0382184	0.003411823	3.340994755	0.00839
ENSG00000152766	ANKRD22	0.289339532	0.025829845	3.340994755	0.00839
ENSG00000177675	CD163L1	0.088123973	0.007866981	3.340994755	0.00839
ENSG00000267296	CEBPA-DT	0.22812803	0.020365388	3.340994755	0.00839
ENSG00000278705	HIST1H4B	1.14480687	0.102198909	3.340994755	0.00839
ENSG00000227953	LINC01341	0.125859791	0.011235723	3.340994755	0.00839
ENSG00000258616	LINC02303	0.438308924	0.039128603	3.340994755	0.00839
ENSG00000255571	MIR9-3HG	0.069019327	0.006161476	3.340994755	0.00839
ENSG00000277311	RF02247	2.310716171	0.206281668	3.340994755	0.00839
ENSG00000144331	ZNF385B	0.34624479	0.033719866	3.32594723	7.98E-06
ENSG00000169583	CLIC3	3.834178999	0.380315395	3.322592842	3.74E-08
ENSG00000220848	RPS18P9	2.616132569	0.259496359	3.322592842	3.74E-08
ENSG00000143632	ACTA1	0.922354431	0.089825663	3.313313657	5.89E-05
ENSG00000135454	B4GALNT1	0.172300372	0.016779878	3.313313657	5.89E-05
ENSG00000164929	BAALC	0.34498871	0.03359754	3.313313657	5.89E-05
ENSG00000139220	PPFIA2	0.160844497	0.015664221	3.313313657	5.89E-05
ENSG00000218896	TUBB8P2	2.785696717	0.271291648	3.313313657	5.89E-05
ENSG00000135373	EHF	1.219456839	0.122470922	3.30727685	1.30E-08
ENSG00000117983	MUC5B	0.121325645	0.012034391	3.307183123	2.52E-06
ENSG00000140022	STON2	5.300740105	0.538624355	3.301732993	1.58E-12
ENSG00000167971	CASKIN1	1.575202932	0.15962397	3.299902411	4.01E-10
ENSG00000154556	SORBS2	0.922830012	0.093903175	3.297030567	2.65E-11
ENSG00000215068	AC025171.2	0.331510969	0.032284978	3.288445767	0.00074
ENSG00000100206	DMC1	0.260862632	0.025404723	3.288445767	0.00074
ENSG00000273079	GRIN2B	0.029710737	0.00289345	3.288445767	0.00074
ENSG00000222038	POTEJ	0.271735122	0.026463566	3.288445767	0.00074
ENSG00000134755	DSC2	1.735626166	0.179124137	3.277856211	1.23E-11
ENSG00000091622	PITPNM3	1.146155733	0.1181419	3.277191539	1.11E-10
ENSG00000137642	SORL1	21.26978058	2.214104752	3.268404214	3.23E-13
ENSG00000058091	CDK14	6.273009491	0.654335944	3.264049478	2.69E-12
ENSG00000131398	KCNC3	0.279590399	0.028525169	3.259034007	1.38E-05
ENSG00000253313	C1orf210	10.72199231	1.122030762	3.256841897	3.49E-11
ENSG00000166126	AMN	1.295115133	0.134886721	3.25670505	7.70E-09
ENSG00000167183	PRR15L	6.189418315	0.648313814	3.252517773	5.46E-10
ENSG00000145990	GFOD1	1.990341984	0.209447511	3.249794378	1.81E-11
ENSG00000279636	LINC00216	2.300797466	0.238524629	3.248701298	1.17E-06
ENSG00000163406	SLC15A2	0.894921892	0.093707019	3.248134748	1.44E-08
ENSG00000025039	RRAGD	9.19411732	0.970809033	3.246590717	2.71E-12
ENSG00000171606	ZNF274	5.330365313	0.564037898	3.243442382	3.15E-12

ENSG00000117394	SLC2A1	137.3152704	14.54517193	3.243335122	4.37E-13
ENSG00000177706	FAM20C	4.38706612	0.464167175	3.241851048	2.33E-11
ENSG00000213638	ADAT3	6.612449517	0.700361765	3.239120801	6.29E-11
ENSG00000139292	LGR5	6.899254581	0.733122927	3.237058531	4.84E-12
ENSG00000267041	ZNF850	4.13063819	0.439077757	3.236349894	6.67E-12
ENSG00000100336	APOL4	0.295944476	0.030680687	3.223478522	0.000112
ENSG00000100399	CHADL	0.498402837	0.051669629	3.223478522	0.000112
ENSG00000082684	SEMA5B	0.152089817	0.015767214	3.223478522	0.000112
ENSG00000128710	HOXD10	0.325963769	0.033256406	3.221732316	0.001085
ENSG00000163083	INHBB	0.273874677	0.027942024	3.221732316	0.001085
ENSG00000166165	CKB	20.2430078	2.180294438	3.218188713	3.13E-12
ENSG00000171714	ANO5	0.609036648	0.065243718	3.21309773	6.16E-08
ENSG00000132639	SNAP25	1.97911429	0.213348213	3.208440795	6.08E-09
ENSG00000108984	MAP2K6	12.22829703	1.327708331	3.20745546	1.06E-12
ENSG00000171813	PWWP2B	30.76771025	3.360189087	3.1985789	2.30E-12
ENSG00000103196	CRISPLD2	0.671505047	0.073059709	3.192906963	2.70E-08
ENSG00000270127	AC027020.2	0.699630821	0.074948718	3.188865717	2.41E-05
ENSG00000116819	TFAP2E	0.754250014	0.080799859	3.188865717	2.41E-05
ENSG00000127578	WFIKKN1	0.342644123	0.036706127	3.188865717	2.41E-05
ENSG00000254221	PCDHGB1	0.516518247	0.056270424	3.177300465	2.29E-06
ENSG00000269834	ZNF528-AS1	0.592630191	0.064562195	3.177300465	2.29E-06
ENSG00000269949	AC069307.1	2.804392668	0.300423637	3.176372239	0.000155
ENSG00000131379	C3orf20	0.358981536	0.03845629	3.176372239	0.000155
ENSG00000089335	ZNF302	7.498462249	0.836195045	3.167592237	9.79E-12
ENSG00000198570	RD3	1.224374436	0.136408951	3.158726135	3.96E-08
ENSG00000135299	ANKRD6	1.439839641	0.161231079	3.158109918	3.57E-10
ENSG00000267270	PARD6G-AS1	0.701128723	0.078035514	3.152811382	3.99E-07
ENSG00000120217	CD274	0.203434522	0.021793146	3.15178368	0.001598
ENSG00000138735	PDE5A	2.111714852	0.238126071	3.150201192	5.91E-11
ENSG00000109819	PPARGC1A	0.695783495	0.078242807	3.149043262	4.48E-09
ENSG00000196437	ZNF569	5.83963337	0.660380986	3.146974852	2.45E-11
ENSG00000120875	DUSP4	0.43760428	0.049346145	3.133984129	4.82E-07
ENSG00000184916	JAG2	14.08307517	1.609429115	3.133226633	4.85E-12
ENSG00000137877	SPTBN5	1.049885697	0.119726295	3.132302254	3.25E-10
ENSG00000104140	RHOV	5.293640683	0.603842701	3.129871073	1.94E-09
ENSG00000268895	A1BG-AS1	0.433862539	0.04808069	3.127675794	0.000215
ENSG00000101460	MAP1LC3A	0.687728758	0.076214169	3.127675794	0.000215
ENSG00000233087	RAB6D	0.399662953	0.044290688	3.127675794	0.000215
ENSG00000105289	TJP3	18.19257472	2.091139442	3.124761843	6.42E-12
ENSG00000183054	RGPD6	0.207216151	0.023543606	3.120401498	1.36E-06
ENSG00000263002	ZNF234	2.39003154	0.274936642	3.119600118	4.20E-10
ENSG00000161298	ZNF382	1.366020214	0.158201527	3.110849796	2.72E-10
ENSG00000261122	LINC02167	0.987388731	0.112526702	3.107396866	1.12E-05

ENSG00000112297	CRYBG1	0.817664016	0.094928541	3.104162158	3.17E-09
ENSG00000106868	SUSD1	3.676080774	0.428048023	3.103033027	3.01E-10
ENSG00000173705	SUSD5	1.535248191	0.178766462	3.099198146	5.94E-09
ENSG00000057294	PKP2	12.8684712	1.519009818	3.086550775	8.71E-12
ENSG00000128602	SMO	10.53602064	1.246015921	3.083211195	2.04E-11
ENSG00000284735	AL139424.3	0.333441228	0.03760027	3.078270022	0.002365
ENSG00000229422	AL512625.2	0.682064918	0.076912581	3.078270022	0.002365
ENSG00000274641	HIST1H2BO	2.083788883	0.234977018	3.078270022	0.002365
ENSG00000249709	ZNF564	0.2191948	0.024717351	3.078270022	0.002365
ENSG00000105357	MYH14	10.16891888	1.219011116	3.064397512	1.06E-11
ENSG00000280287	AC131212.3	0.98050714	0.11670878	3.061282542	3.12E-07
ENSG00000171219	CDC42BPG	5.120631973	0.615199975	3.059925208	5.48E-11
ENSG00000144648	ACKR2	0.68914982	0.082028811	3.059902903	2.75E-07
ENSG00000134243	SORT1	18.18761923	2.189288061	3.058637381	8.49E-12
ENSG00000152128	TMEM163	0.442174082	0.052631537	3.056117533	1.03E-06
ENSG00000170903	MSANTD4	19.69359344	2.376259755	3.054952805	1.20E-11
ENSG00000154153	RETREG1	0.576287959	0.068594977	3.053421418	2.55E-06
ENSG00000140284	SLC27A2	4.086086191	0.495237966	3.044146941	1.29E-09
ENSG00000117595	IRF6	11.18486688	1.361375915	3.041953143	2.56E-11
ENSG00000163251	FZD5	17.13651983	2.088678768	3.040479115	1.38E-11
ENSG00000274021	AC024909.2	2.129077021	0.257829236	3.038561332	1.30E-07
ENSG00000105538	RASIP1	1.158189163	0.140255624	3.038561332	1.30E-07
ENSG00000236526	AL035448.1	0.821559928	0.097789453	3.037378247	7.63E-05
ENSG00000146013	GFRA3	0.737751951	0.087813873	3.037378247	7.63E-05
ENSG00000168993	CPLX1	3.646290368	0.444298977	3.035016991	4.87E-09
ENSG00000178429	RPS3AP5	1.374186566	0.163568046	3.025055866	0.000419
ENSG00000213160	KLHL23	9.458423869	1.170065268	3.018506353	3.81E-11
ENSG00000175182	FAM131A	1.502871674	0.185572606	3.018096926	8.14E-10
ENSG00000179023	KLHDC7A	4.012052998	0.495917969	3.018044469	2.38E-10
ENSG00000145687	SSBP2	2.084828853	0.257852085	3.017520214	1.58E-10
ENSG00000101004	NINL	2.107355456	0.260770517	3.014777759	1.04E-09
ENSG00000142632	ARHGEF19	9.428225872	1.171371416	3.011786394	8.32E-11
ENSG00000124839	RAB17	9.251681445	1.151131926	3.009977879	5.12E-11
ENSG00000102886	GDPD3	2.67373852	0.331403336	3.005888907	1.31E-07
ENSG00000158747	NBL1	4.787432067	0.597505521	3.003607886	5.08E-10
ENSG00000136111	TBC1D4	5.521106318	0.690217375	3.003150991	5.63E-11
ENSG00000151715	TMEM45B	2.374330205	0.295758982	3.001971516	1.71E-08
ENSG00000233382	NKAPP1	1.232035327	0.152822577	3.001860641	5.76E-07
ENSG00000276292	AC131159.1	3.531164853	0.420311004	3.000808344	0.002365
ENSG00000278231	AL133342.1	2.055022168	0.244607224	3.000808344	0.002365
ENSG00000135374	ELF5	0.167364956	0.019921283	3.000808344	0.002365
ENSG00000198718	TOGARAM1	2.017284755	0.252702071	2.998833694	2.86E-10
ENSG00000226465	AL390198.1	1.406241131	0.172165854	2.99688355	0.000102

ENSG00000150637	CD226	0.107236455	0.01312894	2.99688355	0.000102
ENSG00000228502	EEF1A1P11	1.052909126	0.128907479	2.99688355	0.000102
ENSG00000113389	NPR3	0.148235432	0.018148438	2.99688355	0.000102
ENSG00000282458	WASH5P	0.231004703	0.028281865	2.99688355	0.000102
ENSG00000109452	INPP4B	1.210487869	0.151814446	2.996589663	5.56E-10
ENSG00000277117	FP565260.3	0.647257504	0.08000558	2.99552111	1.15E-05
ENSG00000049540	ELN	0.708985789	0.088951481	2.987545445	2.25E-07
ENSG00000128487	SPECC1	4.237819553	0.536790875	2.984518192	5.23E-11
ENSG00000100167	3-Sep	0.251191721	0.031289754	2.979440356	3.15E-05
ENSG00000170873	MTSS1	1.957594561	0.2491024	2.975292015	1.07E-09
ENSG00000182568	SATB1	0.342784666	0.043397762	2.974504529	2.58E-07
ENSG00000154122	ANKH	4.99372652	0.637862154	2.972516543	5.23E-11
ENSG00000176222	ZNF404	0.438249988	0.054170781	2.970872119	0.000589
ENSG00000085185	BCORL1	6.605403922	0.84532659	2.969615709	6.71E-11
ENSG00000180336	MEIOC	1.504645108	0.192384192	2.967812425	1.54E-09
ENSG00000183682	BMP8A	1.259490537	0.158734475	2.967576791	1.15E-05
ENSG00000259953	AL138756.1	0.656677886	0.083306058	2.966447498	1.16E-06
ENSG00000118407	FILIP1	1.646832834	0.211099385	2.964640496	1.36E-09
ENSG00000154265	ABCA5	1.905401311	0.244941844	2.962030627	2.97E-10
ENSG00000164938	TP53INP1	5.580227568	0.717661046	2.96183667	1.60E-10
ENSG00000064547	LPAR2	8.399156042	1.081525094	2.959624647	3.07E-10
ENSG00000137266	SLC22A23	2.188359188	0.281718906	2.959366598	5.28E-10
ENSG00000125531	FNDC11	0.864526567	0.111136027	2.947371296	1.39E-06
ENSG00000269416	LINC01224	3.638478267	0.47533655	2.936544957	2.77E-09
ENSG00000092969	TGFB2	0.535398903	0.069521447	2.930821383	3.39E-06
ENSG00000005073	HOXA11	1.775021315	0.232838086	2.922431625	7.37E-07
ENSG00000279329	AC020910.5	0.380886146	0.048003348	2.918950405	0.003515
ENSG00000240207	AC080013.1	0.170062883	0.021433144	2.918950405	0.003515
ENSG00000253908	AC104115.2	1.033992231	0.130314766	2.918950405	0.003515
ENSG00000251609	SETP12	0.882425668	0.111212725	2.918950405	0.003515
ENSG00000129204	USP6	0.063030405	0.007943766	2.918950405	0.003515
ENSG00000196345	ZKSCAN7	0.168810043	0.021275248	2.918950405	0.003515
ENSG00000079150	FKBP7	3.296709264	0.436004435	2.918132948	6.76E-09
ENSG00000123989	CHPF	124.2973889	16.4958911	2.918051862	4.00E-11
ENSG00000137440	FGFBP1	4.735069475	0.625827312	2.916164193	6.17E-08
ENSG00000144229	THSD7B	0.20395206	0.026483137	2.912315954	0.000186
ENSG00000257497	AC121761.1	1.156007747	0.151022679	2.910938253	5.36E-05
ENSG00000270510	AP000811.1	2.631648972	0.34380278	2.910938253	5.36E-05
ENSG00000234949	AC104667.2	2.04385357	0.270308707	2.908129409	1.07E-06
ENSG00000165716	FAM69B	9.534623934	1.274689494	2.905625572	5.13E-10
ENSG00000108821	COL1A1	0.510668509	0.068382404	2.895217087	2.81E-07
ENSG00000146674	IGFBP3	65.98280104	8.911494418	2.892741812	5.85E-11
ENSG00000100867	DHRS2	10.38553157	1.409955403	2.88477707	1.30E-10

ENSG00000158578	ALAS2	1.096525418	0.146833109	2.883865171	1.17E-05
ENSG00000105963	ADAP1	1.50236113	0.203388346	2.882939195	3.43E-08
ENSG00000183742	MACC1	1.232708792	0.167330321	2.8815496	4.27E-09
ENSG00000105784	RUNDC3B	0.913779496	0.123550018	2.879760278	6.92E-07
ENSG00000165175	MID1IP1	23.92465123	3.259794634	2.879655863	1.22E-10
ENSG00000127329	PTPRB	1.379247446	0.187769938	2.878931746	1.09E-09
ENSG00000170899	GSTA4	3.938398807	0.535099389	2.878502839	1.91E-08
ENSG00000070759	TESK2	4.352127021	0.592889033	2.877023207	3.35E-09
ENSG00000251595	ABCA11P	0.599074563	0.080220649	2.875427554	7.02E-05
ENSG00000112238	PRDM13	0.510200864	0.068319783	2.875427554	7.02E-05
ENSG00000162591	MEGF6	15.77862312	2.162162447	2.871538595	1.25E-10
ENSG00000064989	CALCRL	1.013351589	0.138427623	2.868586724	1.05E-07
ENSG00000113211	PCDHB6	1.407348311	0.191881111	2.868539143	4.96E-07
ENSG00000130957	FBP2	1.02462408	0.137204972	2.868097516	0.000252
ENSG00000116574	RHOU	1.64389239	0.225249013	2.865256249	4.87E-08
ENSG00000139173	TMEM117	6.316405347	0.871839875	2.859282841	1.05E-09
ENSG00000234869	AL021392.1	1.218565197	0.166969936	2.858474673	2.41E-06
ENSG00000260423	LINC02367	0.523746534	0.071408809	2.857930295	1.46E-05
ENSG00000151320	AKAP6	1.487501108	0.206271222	2.853182732	6.40E-10
ENSG00000125351	UPF3B	27.1652898	3.777478647	2.850248169	1.94E-10
ENSG00000233024	AC126755.2	1.710031648	0.236372921	2.850084488	3.10E-07
ENSG00000183665	TRMT12	4.015418691	0.556898557	2.849450285	1.69E-08
ENSG00000163884	KLF15	4.518932175	0.628695533	2.846356171	7.01E-09
ENSG00000234284	ZNF879	1.409707455	0.195975553	2.842404726	2.37E-07
ENSG00000261801	LOXL1-AS1	1.673001937	0.233306961	2.839890138	6.50E-08
ENSG00000186352	ANKRD37	1.862529782	0.260250709	2.833883953	4.73E-07
ENSG00000279667	AL603839.4	0.665241007	0.08908084	2.83216701	0.005247
ENSG00000184956	MUC6	0.079326912	0.010622478	2.83216701	0.005247
ENSG00000185585	OLFML2A	0.099105724	0.013271012	2.83216701	0.005247
ENSG00000199331	RF00019	6.554580511	0.877708274	2.83216701	0.005247
ENSG00000143554	SLC27A3	2.910467411	0.409169305	2.832042619	3.66E-09
ENSG00000139832	RAB20	13.15773746	1.857758026	2.82645759	1.91E-09
ENSG00000234311	AL451069.3	25.32701556	3.57928221	2.825643439	1.23E-09
ENSG00000171817	ZNF540	0.815515303	0.11440389	2.824578829	3.35E-06
ENSG00000179841	AKAP5	0.845499004	0.119177641	2.823776646	1.30E-07
ENSG00000112419	PHACTR2	24.70866328	3.505593746	2.821672823	1.51E-10
ENSG00000223960	AC009948.1	2.527387924	0.358015472	2.819439398	1.64E-08
ENSG00000105519	CAPS	4.079614426	0.579068944	2.818361133	3.64E-09
ENSG00000138778	CENPE	14.33111764	2.038134524	2.818038812	2.21E-10
ENSG00000174951	FUT1	3.463680008	0.494734021	2.809025734	5.46E-09
ENSG00000042980	ADAM28	0.50494663	0.071804815	2.808601498	6.15E-07
ENSG00000079257	LXN	7.084634271	1.011932278	2.807746987	1.43E-08
ENSG00000106537	TSPAN13	21.86972333	3.139859274	2.803762474	5.42E-10

ENSG00000197168	NEK5	1.567404807	0.225166609	2.799608646	1.39E-08
ENSG00000230490	AL139383.1	0.626101217	0.087484929	2.794923839	0.001665
ENSG00000128652	HOXD3	0.270875244	0.037849314	2.794923839	0.001665
ENSG00000116062	MSH6	7.374126669	1.066855085	2.79309535	4.03E-10
ENSG00000267733	AP005264.5	1.551112251	0.222903731	2.789867408	3.95E-06
ENSG00000064042	LIMCH1	0.707836765	0.103026887	2.779004783	7.37E-08
ENSG00000265018	AGAP12P	1.288384732	0.186513008	2.777949926	3.73E-06
ENSG00000271781	AC026740.1	2.746957572	0.396133823	2.777205203	2.89E-05
ENSG00000109654	TRIM2	45.92187787	6.720405048	2.777013199	2.57E-10
ENSG00000272468	AL021807.1	2.2186965	0.316907058	2.775374535	0.000466
ENSG00000232640	AL354892.2	3.110579461	0.444298977	2.775374535	0.000466
ENSG00000165905	LARGE2	12.15332854	1.781902768	2.773217563	1.08E-09
ENSG00000168556	ING2	12.4242855	1.82065994	2.772621588	4.64E-09
ENSG00000143995	MEIS1	5.839415471	0.859727054	2.767721852	6.55E-10
ENSG00000139318	DUSP6	169.3068523	25.01013809	2.763554896	2.93E-10
ENSG00000168785	TSPAN5	0.899821281	0.132542218	2.761000742	1.58E-07
ENSG00000197016	ZNF470	1.228345545	0.181340164	2.760695682	2.09E-08
ENSG00000182253	SYNM	2.971314826	0.440818569	2.755473078	3.34E-09
ENSG00000166689	PLEKHA7	2.35796664	0.350380312	2.753380323	2.60E-09
ENSG00000196526	AFAP1	14.35594203	2.136462278	2.752599119	4.83E-10
ENSG00000131849	ZNF132	2.736727849	0.406129828	2.752361833	3.38E-08
ENSG00000231466	AL022324.2	2.732125627	0.401720325	2.749260884	2.89E-05
ENSG00000164199	ADGRV1	1.094610294	0.164112918	2.741017851	1.60E-09
ENSG00000166780	C16orf45	0.169951671	0.024275012	2.739827258	0.007865
ENSG00000264049	MIR4737	7.738046437	1.105262271	2.739827258	0.007865
ENSG00000253910	PCDHGB2	0.131428342	0.01877254	2.739827258	0.007865
ENSG00000226245	ZNF32-AS1	1.444197607	0.206281668	2.739827258	0.007865
ENSG00000151690	MFSD6	1.434188079	0.215243001	2.735650567	5.94E-08
ENSG00000121089	NACA3P	1.418641847	0.207236676	2.731159497	0.00237
ENSG00000004139	SARM1	1.19687158	0.180869219	2.727519511	1.82E-08
ENSG00000266921	AC006213.1	0.41656173	0.061551216	2.72667809	0.000637
ENSG00000185652	NTF3	0.41513466	0.061340352	2.72667809	0.000637
ENSG00000110876	SELPLG	2.965527179	0.448708119	2.723088381	1.27E-07
ENSG00000277075	HIST1H2AE	3.53000861	0.527659591	2.722093788	8.24E-05
ENSG00000198478	SH3BGRL2	9.348517997	1.42380014	2.718548855	1.74E-09
ENSG00000095932	SMIM24	1.980485316	0.298819239	2.718349843	6.48E-06
ENSG00000224858	RPL29P11	10.70115202	1.637675194	2.704421929	7.49E-07
ENSG00000164764	SBSPON	0.934285689	0.14298054	2.702096351	2.67E-06
ENSG00000147155	EBP	50.68372234	7.822861912	2.699960518	1.00E-09
ENSG00000175175	PPM1E	0.40239734	0.061581794	2.696304672	1.41E-05
ENSG00000254122	PCDHGB7	0.471201217	0.072111352	2.694285056	2.74E-05
ENSG00000078399	HOXA9	0.675961402	0.103447294	2.691694067	4.60E-05
ENSG00000158296	SLC13A3	0.241534397	0.036963767	2.683445386	0.00028

ENSG00000259248	USP3-AS1	0.204801957	0.031342334	2.683445386	0.00028
ENSG00000198756	COLGALT2	0.444298285	0.068813408	2.683104198	6.52E-06
ENSG00000085117	CD82	6.503852844	1.015837992	2.681581763	5.33E-09
ENSG00000198429	ZNF69	2.15271343	0.33599068	2.677546713	3.93E-07
ENSG00000275895	U2AF1L5	3.055272189	0.479072894	2.676304883	3.90E-09
ENSG00000248243	LINC02014	1.640943368	0.251125509	2.676280363	0.000872
ENSG00000188868	ZNF563	0.807235614	0.125783272	2.66835018	3.41E-05
ENSG00000167703	SLC43A2	6.441562511	1.017151838	2.666822927	2.12E-09
ENSG00000275632	AL035461.2	1.674607759	0.256277416	2.664446047	0.003385
ENSG00000152822	GRM1	0.120667556	0.018466634	2.664446047	0.003385
ENSG00000223760	MED15P9	0.389651184	0.059631157	2.664446047	0.003385
ENSG00000262576	PCDHGA4	0.179556879	0.027478896	2.664446047	0.003385
ENSG00000090339	ICAM1	2.221190987	0.350278073	2.664261074	1.34E-07
ENSG00000249863	AC021106.1	1.895748239	0.296164323	2.662025705	5.82E-05
ENSG00000152192	POU4F1	1.121140311	0.177201714	2.657927759	1.21E-06
ENSG00000151090	THRB	1.440089386	0.228549857	2.657515899	2.07E-08
ENSG00000278000	AC139100.2	2.587920666	0.405708054	2.653591685	0.000138
ENSG00000272602	ZNF595	13.32289734	2.128325402	2.649890255	3.14E-09
ENSG00000088280	ASAP3	3.577062445	0.574346392	2.641052667	1.76E-08
ENSG00000129128	SPCS3	37.80937947	6.107338555	2.634528377	1.70E-09
ENSG00000134317	GRHL1	1.571158171	0.253312436	2.63414761	5.24E-08
ENSG00000152939	MARVELD2	13.12901043	2.127207622	2.629708996	3.32E-09
ENSG00000182010	RTKN2	3.501085108	0.567274587	2.629031981	6.82E-09
ENSG00000010610	CD4	0.84792645	0.136740936	2.626647362	5.55E-06
ENSG00000270659	AC079610.2	1.635082856	0.259496359	2.624058163	0.001197
ENSG00000130997	POLN	0.146121897	0.023190324	2.624058163	0.001197
ENSG00000145569	OTULINL	4.256495352	0.692213226	2.622617444	2.23E-08
ENSG00000219891	ZSCAN12P1	5.976555118	0.972524074	2.620014098	9.53E-08
ENSG00000274333	CU633967.1	0.190888309	0.030673679	2.618080986	0.000178
ENSG00000187867	PALM3	0.659857098	0.106031872	2.618080986	0.000178
ENSG00000198846	TOX	0.404603735	0.065015428	2.618080986	0.000178
ENSG00000204920	ZNF155	2.298562723	0.375833971	2.612099673	2.40E-07
ENSG00000064270	ATP2C2	0.596772624	0.097417023	2.610405191	3.19E-06
ENSG00000128203	ASPHD2	2.439662898	0.4000279	2.60821398	2.16E-07
ENSG00000184060	ADAP2	1.961130489	0.322294478	2.604403508	3.30E-07
ENSG00000166147	FBN1	0.458008304	0.07526962	2.604403508	3.30E-07
ENSG00000198929	NOS1AP	3.247768918	0.535705873	2.602927003	1.34E-08
ENSG00000272674	PCDHB16	0.601590173	0.098459176	2.602555351	2.18E-05
ENSG00000279133	AC018628.1	0.503307341	0.082048142	2.600793408	9.35E-05
ENSG00000180628	PCGF5	8.310807206	1.374259102	2.60032042	4.79E-09
ENSG00000128165	ADM2	14.09135173	2.334465902	2.597549751	5.51E-09
ENSG00000135929	CYP27A1	1.274715394	0.210085051	2.595327146	7.47E-06
ENSG00000136147	PHF11	1.286104558	0.212673374	2.594539553	7.71E-07

ENSG00000099617	EFNA2	0.880620669	0.141506181	2.59449741	0.004849
ENSG00000239335	LLPH-DT	1.341426991	0.215552754	2.59449741	0.004849
ENSG00000185904	LINC00839	17.87995326	2.968884854	2.593968261	7.60E-09
ENSG00000163293	NIPAL1	4.190417927	0.695799823	2.59334301	1.51E-08
ENSG00000137460	FHDC1	1.432354296	0.237632226	2.59199207	1.47E-07
ENSG00000125827	TMX4	49.94056837	8.348434511	2.58507312	3.07E-09
ENSG00000269743	SLC25A53	0.238076345	0.039237214	2.581674116	0.000231
ENSG00000154240	CEP112	0.920994137	0.153981946	2.579618455	4.32E-07
ENSG00000175707	KDF1	11.60577495	1.94730815	2.57798626	2.72E-08
ENSG00000101412	E2F1	65.67477558	11.05218113	2.575330328	4.24E-09
ENSG00000145147	SLIT2	1.342366211	0.225843593	2.572981052	8.25E-08
ENSG00000101977	MCF2	1.120746678	0.188155637	2.572126291	1.36E-06
ENSG00000167555	ZNF528	1.121789944	0.188918217	2.570502077	1.68E-07
ENSG00000268230	AC012313.3	0.2617253	0.043134784	2.569874415	0.001649
ENSG00000273284	AP001033.2	1.083172203	0.178516937	2.569874415	0.001649
ENSG00000154269	ENPP3	0.27964523	0.046088156	2.569874415	0.001649
ENSG00000267053	AC012617.1	0.703195693	0.117180948	2.569174273	0.000119
ENSG00000070404	FSTL3	18.54254923	3.137943013	2.566831304	7.89E-09
ENSG00000196335	STK31	0.729876869	0.123075056	2.56417169	3.69E-06
ENSG00000138028	CGREF1	0.5276202	0.088661791	2.559680769	6.60E-05
ENSG00000167705	RILP	6.636395888	1.128124329	2.558301216	7.82E-08
ENSG00000187239	FNBP1	4.020266853	0.685011412	2.556215688	2.30E-08
ENSG00000205572	SERF1B	4.218262482	0.71890953	2.554373123	1.03E-07
ENSG00000186767	SPIN4	17.6398138	3.011752437	2.554194806	7.67E-09
ENSG00000065361	ERBB3	12.15795281	2.07993265	2.551504135	6.48E-09
ENSG00000168564	CDKN2AIP	11.88261487	2.032775661	2.550889106	1.43E-08
ENSG00000130818	ZNF426	6.097519057	1.044176205	2.5495926	1.18E-08
ENSG00000214796	AC098934.1	2.387336436	0.408588334	2.545895127	6.22E-07
ENSG00000137825	ITPKA	2.376897056	0.406548431	2.544550797	2.92E-06
ENSG00000164061	BSN	1.091958775	0.187605072	2.543581123	4.55E-08
ENSG00000171105	INSR	4.23012218	0.72813422	2.542130363	1.37E-08
ENSG00000047597	XK	2.599056642	0.446857812	2.541881727	9.61E-08
ENSG00000165959	CLMN	1.218886469	0.210971978	2.533048946	5.29E-08
ENSG00000197128	ZNF772	8.485956043	1.475949395	2.527273834	1.40E-08
ENSG00000081059	TCF7	1.893487583	0.329173311	2.526736955	5.49E-08
ENSG00000134548	SPX	1.635359226	0.28476056	2.523214558	1.75E-07
ENSG00000109572	CLCN3	24.85110336	4.342403066	2.521118517	7.83E-09
ENSG00000276790	AC068234.2	1.520926369	0.257259322	2.520983752	0.006966
ENSG00000274943	AC079684.1	1.816758729	0.30729832	2.520983752	0.006966
ENSG00000182224	CYB5D1	0.199478283	0.033741047	2.520983752	0.006966
ENSG00000171757	LRRC34	0.163661836	0.027682821	2.520983752	0.006966
ENSG00000263731	AC145207.5	1.05943723	0.183592223	2.520245081	4.44E-05
ENSG00000250722	SELENOP	1.623520502	0.283310898	2.519230274	2.98E-07

ENSG00000198331	HYLS1	7.147694645	1.253785343	2.514032691	4.77E-08
ENSG00000232653	GOLGA8N	0.137415979	0.02355334	2.513575991	0.002276
ENSG00000136286	MYO1G	0.119701646	0.020517072	2.513575991	0.002276
ENSG00000180096	1-Sep	0.971063356	0.169838736	2.510996061	8.34E-06
ENSG00000013297	CLDN11	0.347092437	0.059972028	2.509042655	0.000884
ENSG00000283646	LINC02009	1.727490679	0.303375954	2.506887505	3.32E-06
ENSG00000144792	ZNF660	0.237380881	0.04123733	2.505982541	0.000392
ENSG00000113356	POLR3G	30.23440926	5.357318155	2.50086692	1.14E-08
ENSG00000168350	DEGS2	0.570737236	0.100230905	2.499769271	4.40E-05
ENSG00000109536	FRG1	24.16065606	4.300616287	2.49372681	2.59E-08
ENSG00000166347	CYB5A	24.05815387	4.292222475	2.491125541	1.03E-08
ENSG00000074657	ZNF532	3.821562855	0.681690921	2.490742397	2.29E-08
ENSG00000197329	PELI1	5.399186728	0.965798268	2.485896257	5.90E-08
ENSG00000256673	AC141557.1	12.53563523	2.238156098	2.483066181	4.21E-06
ENSG00000126460	PRRG2	7.050220087	1.263156881	2.48221111	2.39E-07
ENSG00000138172	CALHM2	1.290670294	0.230440782	2.481288261	1.10E-05
ENSG00000233834	AC005083.1	3.339125921	0.596179205	2.478332037	4.07E-05
ENSG00000120327	PCDHB14	0.571217844	0.101987229	2.477259201	6.37E-05
ENSG00000166833	NAV2	9.01037049	1.626030396	2.474520798	1.53E-08
ENSG00000135525	MAP7	5.032358307	0.912191397	2.467096539	5.49E-08
ENSG00000241547	ACTG1P20	3.214265443	0.573886179	2.466593516	0.000512
ENSG00000185101	ANO9	1.036726183	0.187504619	2.466109949	1.70E-06
ENSG00000113448	PDE4D	0.363841919	0.065876559	2.46409353	2.23E-06
ENSG00000168453	HR	12.80583259	2.32886461	2.46334689	1.83E-08
ENSG00000182378	PLCXD1	12.72259323	2.320943652	2.458721844	2.32E-08
ENSG00000155962	CLIC2	0.956357148	0.173378289	2.455276226	7.64E-05
ENSG00000138615	CILP	0.176093208	0.031440296	2.454991103	0.003149
ENSG00000108771	DHX58	0.260955196	0.046591852	2.454991103	0.003149
ENSG00000261428	AC097461.1	0.873924706	0.158678206	2.451778265	6.54E-05
ENSG00000188039	NWD1	0.276538597	0.050211017	2.451778265	6.54E-05
ENSG00000159915	ZNF233	0.546686964	0.099449049	2.447501777	0.000112
ENSG00000167291	TBC1D16	7.037168946	1.293888597	2.447412269	2.55E-08
ENSG00000146205	ANO7	0.814431086	0.149341408	2.444261579	6.92E-06
ENSG00000164104	HMGB2	160.9982124	29.68077276	2.443923672	1.70E-08
ENSG00000272263	AC034198.2	1.474780615	0.263312482	2.443522074	0.006966
ENSG00000258344	AC078778.2	1.326522246	0.236841915	2.443522074	0.006966
ENSG00000226763	SRRM5	0.278776173	0.049773672	2.443522074	0.006966
ENSG00000105928	GSDME	0.770216948	0.141735544	2.441546662	1.61E-06
ENSG00000237172	B3GNT9	4.741509839	0.874699332	2.440550256	2.28E-07
ENSG00000237276	ANO7L1	0.860906272	0.157458218	2.435275779	0.000315
ENSG00000212994	RPS26P6	4.922998509	0.900407626	2.435275779	0.000315
ENSG00000182552	RWDD4	14.19440925	2.644507205	2.427943301	5.58E-08
ENSG00000269473	AC012313.8	0.270731354	0.049718388	2.426098819	0.00067

ENSG00000243056	EIF4EBP3	2.116484481	0.388681233	2.426098819	0.00067
ENSG00000166922	SCG5	0.665979406	0.122303612	2.426098819	0.00067
ENSG00000140836	ZFHX3	1.200263678	0.223875046	2.425886949	8.62E-08
ENSG00000125637	PSD4	4.827615312	0.905785536	2.418105074	4.05E-08
ENSG00000240087	RPSAP12	5.929016662	1.108996265	2.416666407	5.26E-06
ENSG00000166831	RBPMS2	4.315774781	0.809613819	2.415146126	7.67E-07
ENSG00000143412	ANXA9	10.43492089	1.961144445	2.414438722	1.65E-07
ENSG00000025423	HSD17B6	0.533353025	0.098510392	2.413239927	0.001592
ENSG00000174327	SLC16A13	2.282482696	0.428970982	2.409147201	7.69E-06
ENSG00000160190	SLC37A1	3.372761655	0.637220844	2.407163551	1.21E-07
ENSG00000147050	KDM6A	10.76974778	2.037822473	2.406005767	4.27E-08
ENSG00000179627	ZBTB42	4.419306316	0.836801	2.403483999	2.33E-07
ENSG00000114854	TNNC1	19.60481765	3.7174853	2.401909061	1.28E-07
ENSG00000135842	FAM129A	1.177898516	0.224564157	2.392214785	9.25E-07
ENSG00000168646	AXIN2	1.079417499	0.205571169	2.391229957	4.64E-06
ENSG00000124635	HIST1H2BJ	5.190192831	0.989500591	2.388868428	8.48E-06
ENSG00000003987	MTMR7	0.866247925	0.165148553	2.388868428	8.48E-06
ENSG00000178127	NDUFV2	1.387196816	0.264186629	2.388308022	2.59E-05
ENSG00000161544	CYGB	1.431105638	0.273223532	2.385718965	1.54E-05
ENSG00000236609	ZNF853	2.361739244	0.452621664	2.384417899	9.75E-07
ENSG00000115616	SLC9A2	0.93570361	0.179531238	2.380513836	5.17E-06
ENSG00000095585	BLNK	0.514458354	0.097976738	2.379594158	0.000258
ENSG00000243789	JMJD7	0.954005725	0.181686949	2.379594158	0.000258
ENSG00000164197	RNF180	0.984272672	0.189464815	2.375575927	6.50E-06
ENSG00000184635	ZNF93	4.022008793	0.778279769	2.372662217	1.81E-07
ENSG00000233080	LINC01399	2.02187665	0.388761605	2.36335821	0.000519
ENSG00000138606	SHF	0.332985815	0.064025716	2.36335821	0.000519
ENSG00000120784	ZFP30	3.24304991	0.633248578	2.359776482	1.94E-07
ENSG00000108830	RND2	0.950323828	0.185099057	2.35690131	2.01E-05
ENSG00000239887	C1orf226	12.6146511	2.469689488	2.356689791	9.32E-08
ENSG00000178568	ERBB4	0.247213193	0.04815088	2.354690613	5.57E-05
ENSG00000164056	SPRY1	65.15071921	12.79753146	2.35234869	5.36E-08
ENSG00000165029	ABCA1	0.342382989	0.067046086	2.348720801	2.80E-05
ENSG00000174718	KIAA1551	21.06877001	4.148974289	2.348608864	6.65E-08
ENSG00000145113	MUC4	0.23513501	0.046180009	2.346350913	1.04E-05
ENSG00000120802	TMPO	141.8653253	28.00495097	2.345299072	5.31E-08
ENSG00000102302	FGD1	6.050337782	1.193683252	2.34492426	2.13E-07
ENSG00000178458	H3F3AP6	102.4811055	20.25776322	2.342615427	1.26E-07
ENSG00000276449	AC004076.2	0.955670857	0.18858974	2.33586336	6.53E-05
ENSG00000175697	GPR156	0.337980701	0.066501832	2.334577321	0.000214
ENSG00000166558	SLC38A8	4.341643867	0.861302512	2.334216771	2.27E-06
ENSG00000274104	AC020910.4	1.702370216	0.331578681	2.330161793	0.006073
ENSG00000088340	FER1L4	0.17512376	0.034558493	2.326008764	0.000668

ENSG00000023839	ABCC2	0.287918124	0.057117675	2.324314356	0.000181
ENSG00000129910	CDH15	0.487557118	0.09672239	2.324314356	0.000181
ENSG00000112312	GMNN	22.56346836	4.519701329	2.323688238	1.35E-07
ENSG00000170390	DCLK2	0.780188616	0.155494996	2.322815796	4.65E-05
ENSG00000101986	ABCD1	10.12801145	2.030361308	2.322372874	1.66E-07
ENSG00000269737	AL691432.1	3.07820253	0.61521608	2.321909414	7.77E-06
ENSG00000167861	HID1	6.103265484	1.228168291	2.316810191	1.92E-07
ENSG00000231010	AL121672.1	1.521400832	0.303221825	2.314197767	0.000412
ENSG00000134909	ARHGAP32	2.141174877	0.432258167	2.311871567	2.67E-07
ENSG00000246596	AC139795.1	0.505922498	0.100365744	2.310619999	0.00289
ENSG00000224292	AF196972.1	1.727729204	0.342749785	2.310619999	0.00289
ENSG00000165272	AQP3	0.382443109	0.075869698	2.310619999	0.00289
ENSG00000135363	LMO2	0.362884262	0.071989582	2.310619999	0.00289
ENSG00000205413	SAMD9	0.164581644	0.03264998	2.310619999	0.00289
ENSG00000125775	SDCBP2	5.843347995	1.181085012	2.30925176	7.33E-07
ENSG00000102189	EEA1	7.171453716	1.455352734	2.305032141	1.37E-07
ENSG00000171208	NETO2	8.786059891	1.783105246	2.304914635	1.46E-07
ENSG00000186212	SOWAHB	1.985457753	0.403146129	2.299868793	5.96E-06
ENSG00000196967	ZNF585A	1.021525706	0.208045743	2.297533667	1.52E-06
ENSG00000145506	NKD2	1.520787636	0.31031627	2.290642582	2.34E-05
ENSG00000266066	POLRMTP1	0.95691872	0.195258983	2.288919952	6.24E-05
ENSG00000268521	VN1R83P	3.474295906	0.704138997	2.287666634	0.000861
ENSG00000204172	AGAP9	2.380737873	0.487574852	2.286946182	9.35E-06
ENSG00000075618	FSCN1	30.377072	6.244043285	2.286718063	1.41E-07
ENSG00000205269	TMEM170B	3.802765849	0.781561326	2.286225643	3.05E-07
ENSG00000223773	CD99P1	0.882145135	0.181441467	2.280308909	1.37E-05
ENSG00000004399	PLXND1	9.701879664	2.004181677	2.279490313	1.67E-07
ENSG00000164300	SERINC5	7.942292051	1.641782216	2.278455139	1.83E-07
ENSG00000125967	NECAB3	47.85791032	9.907268587	2.276611033	1.33E-07
ENSG00000151012	SLC7A11	41.62082171	8.623108409	2.275507972	1.27E-07
ENSG00000163141	BNIPL	0.515598894	0.105767419	2.274617646	0.000333
ENSG00000158813	EDA	9.438890934	1.957067484	2.274028706	2.02E-07
ENSG00000163435	ELF3	9.059034589	1.879914175	2.272789485	2.07E-07
ENSG00000138641	HERC3	3.848738696	0.80151701	2.26703259	4.40E-07
ENSG00000100191	SLC5A4	1.914308828	0.396914382	2.266824717	4.53E-05
ENSG00000278206	AL031320.2	0.42207526	0.086124333	2.263448343	0.008459
ENSG00000237766	GGTA2P	0.779995081	0.159157767	2.263448343	0.008459
ENSG00000238099	LINC01625	0.519227495	0.105948218	2.263448343	0.008459
ENSG00000186340	THBS2	0.136851913	0.027924593	2.263448343	0.008459
ENSG00000196405	EVL	4.599114202	0.960925695	2.262810707	2.80E-07
ENSG00000113083	LOX	1.122414307	0.234023893	2.261682123	8.70E-06
ENSG00000113163	COL4A3BP	16.68377078	3.492105942	2.260735554	1.54E-07
ENSG00000267751	AC009005.1	3.236560926	0.672740561	2.260319033	0.000169

ENSG00000105649	RAB3A	4.711780751	0.984887172	2.25935341	4.17E-06
ENSG00000260743	AC007823.1	0.693755887	0.142921845	2.256436251	0.003905
ENSG00000214029	ZNF891	1.987208431	0.417074705	2.255965075	4.35E-07
ENSG00000081665	ZNF506	1.395496989	0.292604196	2.255808522	1.87E-06
ENSG00000127334	DYRK2	33.90721641	7.133621606	2.253351587	1.66E-07
ENSG00000112182	BACH2	0.132367563	0.0274452	2.251696087	0.001999
ENSG00000185739	SRL	0.227174496	0.047102549	2.251696087	0.001999
ENSG00000188338	SLC38A3	0.379101872	0.078967201	2.248277609	0.001111
ENSG00000171722	SPATA46	0.807015143	0.168101853	2.248277609	0.001111
ENSG00000225489	AL354707.1	0.603453146	0.126137716	2.245695664	0.00066
ENSG00000043355	ZIC2	35.03160902	7.410933787	2.245235816	2.30E-07
ENSG00000174652	ZNF266	11.00308964	2.327616075	2.245050912	3.07E-07
ENSG00000226950	DANCR	93.88468455	19.88383755	2.243714987	1.94E-07
ENSG00000242615	AC022415.1	1.404784436	0.295076611	2.242054591	0.000273
ENSG00000101844	ATG4A	9.721862647	2.064578567	2.23892444	5.90E-07
ENSG00000178665	ZNF713	0.653200824	0.138564067	2.234678711	3.89E-05
ENSG00000259207	ITGB3	10.27043396	2.200462982	2.226540254	4.25E-07
ENSG00000170345	FOS	5.484501721	1.175066512	2.225562743	1.15E-06
ENSG00000104154	SLC30A4	0.750512865	0.160799026	2.222457147	1.16E-05
ENSG00000159208	CIART	1.704895988	0.365277701	2.220960976	3.00E-05
ENSG00000180834	MAP6D1	1.735276194	0.371786727	2.220341836	4.42E-05
ENSG00000122547	EEPD1	8.069152447	1.736221772	2.220294364	4.97E-07
ENSG00000119772	DNMT3A	6.876174842	1.48589731	2.214485836	3.67E-07
ENSG00000260103	AC012435.1	0.61721493	0.132239651	2.213558295	0.000337
ENSG00000249550	LINC01234	8.172578126	1.766965341	2.213301745	5.79E-07
ENSG00000277938	AL035252.3	0.675411381	0.144708368	2.212057527	0.000519
ENSG00000132170	PPARG	8.75329178	1.895153124	2.211356082	5.73E-07
ENSG00000101489	CELF4	0.255881511	0.054823174	2.210184965	0.000838
ENSG00000197461	PDGFA	21.16955022	4.62620557	2.198328322	4.27E-07
ENSG00000145331	TRMT10A	8.979243929	1.962667142	2.197625167	6.71E-07
ENSG00000214530	STARD10	10.6547268	2.340713116	2.190328599	7.15E-07
ENSG00000010810	FYN	1.843724517	0.404686165	2.190273192	2.29E-06
ENSG00000106853	PTGR1	14.08036333	3.095322263	2.189556285	5.89E-07
ENSG00000196517	SLC6A9	8.674591541	1.908875137	2.188011333	6.75E-07
ENSG00000089682	RBM41	7.294132938	1.614143064	2.179907263	7.40E-07
ENSG00000151929	BAG3	54.97365091	12.20516139	2.175624541	4.59E-07
ENSG00000163701	IL17RE	2.242974367	0.497793486	2.173742394	4.97E-06
ENSG00000185818	NAT8L	6.547881911	1.45626917	2.172597742	8.83E-07
ENSG00000111424	VDR	5.59692326	1.246488063	2.170463401	9.82E-07
ENSG00000164306	PRIMPOL	5.93785512	1.322582569	2.169527088	2.19E-06
ENSG00000197050	ZNF420	5.286799512	1.179440947	2.167569736	1.67E-06
ENSG00000197013	ZNF429	1.827183343	0.40742058	2.166643419	7.28E-06
ENSG00000153093	ACOXL	0.247208165	0.054522682	2.166118671	0.001859

ENSG00000268912	AC012313.5	1.676730444	0.373470624	2.164702226	5.96E-05
ENSG00000235437	LINC01278	4.480521762	1.003468638	2.162281243	1.24E-06
ENSG00000168116	KIAA1586	9.56939239	2.145289538	2.160903344	1.19E-06
ENSG00000184986	TMEM121	2.523806112	0.564241033	2.160144106	4.20E-05
ENSG00000163795	ZNF513	5.318148278	1.192421431	2.159439513	3.56E-06
ENSG00000160050	CCDC28B	2.906298207	0.651273244	2.159106973	1.06E-05
ENSG00000169760	NLGN1	0.119410531	0.026466174	2.155893359	0.00349
ENSG00000234965	SHISA8	0.862475496	0.19115924	2.155893359	0.00349
ENSG00000056277	ZNF280C	3.128949377	0.705159237	2.152363629	3.45E-06
ENSG00000155760	FZD7	9.950979333	2.25033575	2.148559061	1.13E-06
ENSG00000137563	GGH	28.31531663	6.40698998	2.148119494	7.17E-07
ENSG00000180626	ZNF594	2.830779502	0.640195196	2.147396703	3.28E-06
ENSG00000196787	HIST1H2AG	3.0828377	0.696315231	2.147150222	1.38E-05
ENSG00000213080	AL354714.2	4.685150814	1.060930876	2.142142449	3.68E-05
ENSG00000229368	AC090587.2	1.902942729	0.424697552	2.141552939	0.007168
ENSG00000102103	PQBP1	47.77989535	10.86734753	2.140668886	7.85E-07
ENSG00000269001	AC092070.2	1.897463707	0.430839525	2.139953305	1.14E-05
ENSG00000163596	ICA1L	0.465246574	0.105593324	2.138651551	4.38E-05
ENSG00000138814	PPP3CA	18.78862394	4.284825985	2.136837786	7.91E-07
ENSG00000260910	LINC00565	1.774671654	0.403271369	2.13588457	7.67E-05
ENSG00000204977	TRIM13	6.029930147	1.379457595	2.132056542	1.15E-06
ENSG00000154429	CCSAP	8.65331784	1.979783615	2.131981639	1.04E-06
ENSG00000256235	SMIM3	11.55114339	2.65032776	2.127422623	1.78E-06
ENSG00000168970	JMJD7-PLA2G4B	0.99387933	0.227744197	2.126855169	1.58E-05
ENSG00000126803	HSPA2	1.251321271	0.28669474	2.126791772	1.41E-05
ENSG00000230896	AL604028.1	6.218904879	1.422239661	2.125993387	0.000123
ENSG00000170775	GPR37	1.231017914	0.281529069	2.125993387	0.000123
ENSG00000156265	MAP3K7CL	0.436716964	0.099539964	2.1245281	0.000635
ENSG00000197980	LEKR1	0.246147782	0.055933926	2.123215316	0.00241
ENSG00000198315	ZKSCAN8	8.550413453	1.970405765	2.121654995	1.07E-06
ENSG00000164305	CASP3	34.4632676	7.982936893	2.114365486	9.88E-07
ENSG00000183562	AC131971.1	1.184203058	0.271840822	2.112816623	0.001019
ENSG00000170175	CHRNB1	1.650568741	0.382590786	2.110028642	1.66E-05
ENSG00000181315	ZNF322	5.864334856	1.362625179	2.109265819	1.99E-06
ENSG00000108379	WNT3	1.957186549	0.454447939	2.108109912	1.40E-05
ENSG00000139133	ALG10	4.208058609	0.979097678	2.106792662	3.28E-06
ENSG00000284428	IPO5P1	0.607370528	0.140681585	2.106355045	0.000243
ENSG00000186187	ZNRF1	8.036027871	1.871893926	2.106177968	1.26E-06
ENSG00000285437	POLR2J3	0.296185961	0.068435628	2.106118587	0.00062
ENSG00000169862	CTNND2	0.170478307	0.039134304	2.105495632	0.004621
ENSG00000164379	FOXQ1	0.506709667	0.116318203	2.105495632	0.004621
ENSG00000185187	SIGIRR	9.968268287	2.330345594	2.100621198	1.92E-06

ENSG00000196812	ZSCAN16	5.632225667	1.314579636	2.100235381	1.68E-05
ENSG00000128886	ELL3	0.536826972	0.124342005	2.098086519	0.001728
ENSG00000136928	GABBR2	0.202576216	0.046921511	2.098086519	0.001728
ENSG00000185864	NPIPB4	0.370924495	0.086381941	2.097996494	0.000334
ENSG00000164976	MYORG	8.033151678	1.882038107	2.097730383	1.54E-06
ENSG00000188732	FAM221A	1.921749747	0.450152071	2.096712278	5.78E-06
ENSG00000162458	FBLIM1	3.925138822	0.920700346	2.095120405	3.64E-06
ENSG00000113391	FAM172A	9.319531613	2.18916471	2.093895424	1.65E-06
ENSG00000129354	AP1M2	47.03222371	11.05936211	2.092694214	1.23E-06
ENSG00000187837	HIST1H1C	38.53113216	9.064183575	2.091306065	3.04E-06
ENSG00000205336	ADGRG1	16.6244229	3.913348346	2.091209424	1.16E-06
ENSG00000246273	SBF2-AS1	0.494875063	0.115666982	2.090564684	0.000853
ENSG00000115221	ITGB6	1.096567195	0.257863088	2.087941994	5.27E-05
ENSG00000130714	POMT1	4.504315938	1.062450892	2.087542841	2.73E-06
ENSG00000138794	CASP6	16.30127209	3.84737075	2.08679838	2.45E-06
ENSG00000119699	TGFB3	1.19897203	0.282388437	2.086306967	3.35E-05
ENSG00000196782	MAML3	0.893870167	0.210665006	2.085637014	3.02E-05
ENSG00000108244	KRT23	1.699063814	0.401047957	2.082335089	6.32E-05
ENSG00000167614	TTYH1	0.135974161	0.031666046	2.080487972	0.009733
ENSG00000162086	ZNF75A	3.088692627	0.732820551	2.079013112	3.37E-06
ENSG00000171604	CXXC5	35.32020448	8.390180181	2.078092575	1.36E-06
ENSG00000172465	TCEAL1	15.85864798	3.772173199	2.075373797	3.35E-06
ENSG00000256223	ZNF10	4.411566268	1.0502086	2.074149278	3.66E-06
ENSG00000087301	TXNDC16	7.256684105	1.728561156	2.073569265	2.51E-06
ENSG00000167617	CDC42EP5	4.581129153	1.0905744	2.069145788	0.000115
ENSG00000179222	MAGED1	24.71981703	5.9227801	2.065711799	1.55E-06
ENSG00000143845	ETNK2	0.504564924	0.120115712	2.064155038	0.001028
ENSG00000272462	U91328.1	2.488915469	0.596119058	2.063990967	1.29E-05
ENSG00000057468	MSH4	0.5757334	0.137057936	2.06196798	0.000974
ENSG00000135205	CCDC146	2.259433169	0.542339822	2.060795113	1.40E-05
ENSG00000130717	UCK1	33.93513356	8.159944107	2.060434855	1.85E-06
ENSG00000186111	PIP5K1C	14.66061413	3.529362912	2.058781353	1.83E-06
ENSG00000149177	PTPRJ	30.69189138	7.393825447	2.057938192	1.49E-06
ENSG00000133121	STARD13	5.848369002	1.408853624	2.057567667	2.44E-06
ENSG00000123080	CDKN2C	21.45943583	5.180542144	2.054649251	2.12E-06
ENSG00000270228	AC079880.1	0.551420904	0.131270152	2.053273432	0.006128
ENSG00000200090	RF00019	11.39603202	2.712916482	2.053273432	0.006128
ENSG00000268362	AC092279.1	0.934498899	0.22473498	2.04855177	0.000759
ENSG00000107864	CPEB3	1.370360393	0.33312616	2.042734733	1.33E-05
ENSG00000048740	CELF2	0.319728353	0.077473066	2.042701762	0.000244
ENSG00000089486	CDIP1	5.46304628	1.331986056	2.039644641	4.85E-06
ENSG00000205707	ETFRF1	18.06297263	4.410964573	2.037883316	3.06E-06
ENSG00000111837	MAK	0.524048483	0.127107777	2.037252913	0.00124

ENSG00000213064	SFT2D2	21.6803221	5.301683369	2.036321462	1.92E-06
ENSG00000184992	BRI3BP	34.6452173	8.477251888	2.035439835	1.95E-06
ENSG00000148832	PAOX	1.853460925	0.452152747	2.034175961	0.000132
ENSG00000004799	PDK4	4.12399005	1.01036478	2.032430125	7.21E-06
ENSG00000189164	ZNF527	2.155495595	0.528588762	2.030626656	1.06E-05
ENSG00000136802	LRRC8A	32.09410243	7.891319015	2.028383981	2.18E-06
ENSG00000071575	TRIB2	26.95835533	6.653977589	2.022807703	2.52E-06
ENSG00000276007	AC079414.3	1.840093244	0.451737928	2.018883407	0.000931
ENSG00000225526	MKRN2OS	1.096611064	0.269215058	2.018883407	0.000931
ENSG00000143320	CRABP2	1.32953707	0.325549978	2.018202797	0.002817
ENSG00000084453	SLCO1A2	0.126952324	0.031085501	2.018202797	0.002817
ENSG00000185436	IFNLR1	2.860389378	0.709226795	2.014767733	1.22E-05
ENSG00000148400	NOTCH1	7.195779176	1.785866097	2.01476692	3.13E-06
ENSG00000168491	CCDC110	0.962002703	0.237820601	2.013452686	0.000326
ENSG00000262681	AC005722.2	1.500308469	0.371919114	2.012078009	9.17E-05
ENSG00000130024	PHF10	20.93802945	5.20915261	2.01135835	2.90E-06
ENSG00000144043	TEX261	41.59908372	10.36066626	2.009858935	2.63E-06
ENSG00000121211	MND1	20.18754405	5.036221531	2.006838193	5.50E-06
ENSG00000133805	AMPD3	0.424436347	0.105433527	2.005608197	0.00053
ENSG00000135127	BICDL1	1.699153085	0.424449734	2.003191863	2.58E-05
ENSG00000197558	SSPO	0.051382034	0.012702362	1.999089684	0.008135
ENSG00000231107	LINC01508	1.12509354	0.280295066	1.996571589	0.001501
ENSG00000164180	TMEM161B	9.63877766	2.425174351	1.995067409	3.65E-06
ENSG00000147140	NONO	142.8932922	36.10147478	1.989334871	2.98E-06
ENSG00000127252	HRASLS	5.0042456	1.263629619	1.986189955	6.88E-05
ENSG00000196081	ZNF724	2.075436296	0.525515279	1.98283004	4.74E-05
ENSG00000124782	RREB1	5.274644242	1.338690792	1.982555489	4.08E-06
ENSG00000197712	FAM114A1	8.749981477	2.223912033	1.980199211	5.66E-06
ENSG00000115112	TFCP2L1	1.560489518	0.396429697	1.979850834	1.51E-05
ENSG00000162924	REL	1.989330116	0.506431676	1.977527002	8.09E-06
ENSG00000105750	ZNF85	2.748100553	0.700023116	1.975736531	2.02E-05
ENSG00000111252	SH2B3	37.68191652	9.611229988	1.975529689	3.71E-06
ENSG00000076555	ACACB	3.096818998	0.790028212	1.97486001	5.91E-06
ENSG00000154274	C4orf19	1.900710034	0.484799155	1.971311554	0.000105
ENSG00000156427	FGF18	98.10954526	25.10882068	1.970639549	3.94E-06
ENSG00000197457	STMN3	17.59895139	4.517650172	1.965977906	5.80E-06
ENSG00000137868	STRA6	0.614012475	0.157165708	1.965740312	0.000139
ENSG00000140443	IGF1R	4.112304601	1.056385275	1.964973666	5.74E-06
ENSG00000156531	PHF6	29.08196479	7.482857214	1.962923749	4.20E-06
ENSG00000174607	UGT8	14.93925458	3.858068609	1.957394657	5.84E-06
ENSG00000196696	PDXDC2P-NPIPB14P	0.341719421	0.087856962	1.953398977	0.001807
ENSG00000155957	TMBIM4	4.636094247	1.201040981	1.952016709	1.44E-05

ENSG00000204272	NBDY	40.85525326	10.5942011	1.951483659	6.25E-06
ENSG00000260097	SPDYE6	1.334366849	0.344128081	1.949785151	0.001678
ENSG00000214367	HAUS3	4.286748521	1.114802528	1.946918753	9.75E-06
ENSG00000143801	PSEN2	19.03287189	4.951144886	1.946904886	6.39E-06
ENSG00000163257	DCAF16	23.3915231	6.097302327	1.944102975	5.82E-06
ENSG00000117407	ARTN	0.974679438	0.252613555	1.943972515	0.00105
ENSG00000072163	LIMS2	0.305254854	0.079114744	1.943972515	0.00105
ENSG00000251333	RTN3P1	3.643738322	0.944369662	1.943972515	0.00105
ENSG00000235499	AC073046.1	7.4999527	1.951213009	1.939501995	0.000701
ENSG00000176018	LYSMD3	19.65106307	5.149917373	1.936291963	6.62E-06
ENSG00000125898	FAM110A	6.339967363	1.660938765	1.935758505	1.91E-05
ENSG00000109881	CCDC34	15.72691088	4.124970253	1.934962254	7.83E-06
ENSG00000205795	CYS1	12.22595029	3.207473439	1.934363161	1.02E-05
ENSG00000123191	ATP7B	7.954401287	2.087495399	1.934251743	7.03E-06
ENSG00000146267	FAXC	0.118240428	0.030706995	1.933635201	0.004613
ENSG00000196810	CTBP1-DT	1.982404377	0.52090078	1.93131426	2.10E-05
ENSG00000237522	NONOP2	1.391716881	0.363632185	1.928069486	0.002322
ENSG00000188738	FSIP2	0.094263595	0.024768902	1.922883026	0.002011
ENSG00000140450	ARRDC4	1.498201329	0.395744693	1.921584896	0.000104
ENSG00000233927	RPS28	194.4137607	51.5018836	1.920937517	6.27E-06
ENSG00000134453	RBM17	83.96799024	22.25952412	1.919933436	6.27E-06
ENSG00000138587	MNS1	11.06379946	2.931769969	1.919777801	1.39E-05
ENSG00000107249	GLIS3	2.042278923	0.541275701	1.919404339	1.51E-05
ENSG00000234678	ELF3-AS1	1.335573754	0.353668049	1.918636203	8.45E-05
ENSG00000228106	AL392172.1	9.303028326	2.470555843	1.915250868	4.35E-05
ENSG00000268225	AC010487.1	3.458352153	0.916650962	1.914867077	0.000308
ENSG00000156504	FAM122B	13.27380691	3.537140427	1.912115739	9.81E-06
ENSG00000125843	AP5S1	3.607705585	0.962121747	1.910262709	1.95E-05
ENSG00000250312	ZNF718	2.318111777	0.618551574	1.908660689	3.71E-05
ENSG00000174799	CEP135	3.375722758	0.901479539	1.90865147	1.45E-05
ENSG00000012963	UBR7	43.13154406	11.52401976	1.908521709	7.70E-06
ENSG00000196586	MYO6	8.899325938	2.379553552	1.907288183	9.32E-06
ENSG00000136158	SPRY2	93.61151345	25.06322266	1.905595811	7.51E-06
ENSG00000076513	ANKRD13A	15.77613957	4.226613148	1.904542991	8.59E-06
ENSG00000178409	BEND3	5.859372317	1.570865517	1.903211031	1.25E-05
ENSG00000046653	GPM6B	1.207544304	0.323398767	1.902134422	0.000111
ENSG00000112406	HECA	4.60726648	1.235889545	1.902094199	1.65E-05
ENSG00000163072	NOSTRIN	6.791728182	1.825325476	1.89958545	1.36E-05
ENSG00000131174	COX7B	85.98904103	23.13649459	1.89845778	8.14E-06
ENSG00000100068	LRP5L	2.522996067	0.678084416	1.898361606	4.38E-05
ENSG00000151470	C4orf33	2.823421762	0.759345904	1.898100403	2.17E-05
ENSG00000197062	ZSCAN26	5.727156906	1.541450841	1.896824561	2.82E-05
ENSG00000107719	PALD1	9.522129064	2.564912147	1.896443018	1.29E-05

ENSG00000122778	KIAA1549	1.254202393	0.338596078	1.892334892	2.90E-05
ENSG00000146122	DAAM2	0.116115494	0.031097499	1.891160863	0.004061
ENSG00000182853	VMO1	1.873320333	0.501703733	1.891160863	0.004061
ENSG00000132640	BTBD3	17.27641871	4.671809453	1.8911118	1.01E-05
ENSG00000169129	AFAP1L2	43.01974201	11.63732917	1.890711177	8.84E-06
ENSG00000254726	MEX3A	7.075687857	1.915078046	1.889538186	1.38E-05
ENSG00000189157	FAM47E	0.140632565	0.037663544	1.889416763	0.005912
ENSG00000158806	NPM2	0.648150472	0.173584574	1.889416763	0.005912
ENSG00000254560	BBOX1-AS1	3.789991342	1.023546996	1.888936257	0.000214
ENSG00000088836	SLC4A11	3.615124134	0.978485179	1.888640691	2.94E-05
ENSG00000231769	AL035701.1	1.583210584	0.424007921	1.887179725	0.008985
ENSG00000183914	DNAH2	0.067900448	0.018184775	1.887179725	0.008985
ENSG00000186326	RGS9BP	0.404282177	0.108272928	1.887179725	0.008985
ENSG00000137338	PGBD1	2.345376914	0.635347538	1.885870305	0.000106
ENSG00000126878	AIF1L	42.20338412	11.4801062	1.882686093	9.68E-06
ENSG00000203995	ZYG11A	3.436127821	0.934551758	1.881697944	2.96E-05
ENSG00000241962	AC079447.1	1.572458006	0.42674326	1.879468678	0.000755
ENSG00000067141	NEO1	8.833785081	2.409940162	1.878300806	1.28E-05
ENSG00000197372	ZNF675	3.602724936	0.983007395	1.877519507	2.21E-05
ENSG00000127419	TMEM175	4.25581875	1.161394829	1.876785489	3.37E-05
ENSG00000139372	TDG	43.04676969	11.75910689	1.876584882	1.04E-05
ENSG00000171155	C1GALT1C1	28.27833306	7.723832809	1.876453168	1.44E-05
ENSG00000110195	FOLR1	57.83532353	15.80833555	1.875604357	1.22E-05
ENSG00000114279	FGF12	1.42598887	0.389509151	1.874809697	5.58E-05
ENSG00000198513	ATL1	0.977549536	0.266936048	1.87236469	0.000338
ENSG00000164162	ANAPC10	1.700248328	0.465251378	1.87152575	0.000108
ENSG00000268713	AC005261.3	2.956660054	0.807998591	1.87104009	0.000386
ENSG00000169762	TAPT1	5.167132899	1.41814694	1.869403174	1.69E-05
ENSG00000131389	SLC6A6	25.74255114	7.071857407	1.868453753	1.12E-05
ENSG00000109189	USP46	9.5876452	2.635487163	1.867422633	1.36E-05
ENSG00000119514	GALNT12	9.071052587	2.49714579	1.864824	2.24E-05
ENSG00000244490	RWDD4P1	6.064437101	1.663762055	1.864469437	0.000615
ENSG00000102053	ZC3H12B	0.250851473	0.068611248	1.864368781	0.003196
ENSG00000164117	FBXO8	7.345911984	2.023101953	1.863933794	2.80E-05
ENSG00000170425	ADORA2B	2.358723994	0.649249515	1.862305189	0.000149
ENSG00000139800	ZIC5	8.00760201	2.209690651	1.861530842	1.96E-05
ENSG00000146109	ABT1	14.89200025	4.113532062	1.860178628	1.81E-05
ENSG00000163430	FSTL1	3.892697544	1.077146283	1.857511756	2.11E-05
ENSG00000052802	MSMO1	34.31550676	9.503650375	1.856658305	1.45E-05
ENSG00000151623	NR3C2	1.100449286	0.304879747	1.853536678	0.000133
ENSG00000254531	FLJ20021	5.712441369	1.586541032	1.848287352	0.00033
ENSG00000237686	AL109615.3	4.622606486	1.286744795	1.847630381	6.73E-05
ENSG00000134780	DAGLA	1.785516916	0.497627203	1.845795723	7.33E-05

ENSG00000197847	SLC22A20P	0.325219426	0.089908354	1.843800066	0.007582
ENSG00000110799	VWF	0.124660665	0.034462994	1.843800066	0.007582
ENSG00000120437	ACAT2	21.45161429	5.997571121	1.842974498	1.67E-05
ENSG00000029993	HMGB3	47.75781037	13.36010497	1.842256055	1.48E-05
ENSG00000119547	ONECUT2	7.111814737	1.993577503	1.839241168	1.66E-05
ENSG00000109705	NKX3-2	0.74590237	0.207754582	1.83902909	0.002895
ENSG00000158691	ZSCAN12	1.13303588	0.317700047	1.835797463	0.000177
ENSG00000184602	SNN	22.64640221	6.365770061	1.835165344	1.92E-05
ENSG00000133985	TTC9	1.161374422	0.326049192	1.83392987	0.000177
ENSG00000136830	FAM129B	69.03761663	19.43700046	1.833071572	1.56E-05
ENSG00000162482	AKR7A3	1.588335848	0.445013493	1.832791078	0.001513
ENSG00000079482	OPHN1	2.144146428	0.604171515	1.830801122	4.49E-05
ENSG00000174720	LARP7	16.10832709	4.549766254	1.828197376	2.13E-05
ENSG00000281026	N4BP2L2-IT2	0.487069671	0.13731019	1.822929168	0.002419
ENSG00000185774	KCNIP4	0.893438914	0.25242362	1.822875307	0.000638
ENSG00000168538	TRAPPC11	11.66072329	3.310677741	1.820726293	2.28E-05
ENSG00000101882	NKAP	3.551816248	1.009353396	1.818980511	3.38E-05
ENSG00000251615	AC104825.1	0.488718722	0.137775075	1.818802471	0.004498
ENSG00000232630	PRPS1P2	1.694607395	0.477728089	1.818802471	0.004498
ENSG00000125895	TMEM74B	0.718482864	0.202548063	1.818802471	0.004498
ENSG00000111727	HCFC2	4.392623792	1.249111184	1.818077003	3.47E-05
ENSG00000168298	HIST1H1E	2.937173594	0.831145501	1.816917639	0.003145
ENSG00000167785	ZNF558	3.747619851	1.066569988	1.816912473	3.35E-05
ENSG00000123739	PLA2G12A	10.98986128	3.133761025	1.814422017	2.50E-05
ENSG00000136842	TMOD1	61.02163319	17.40788076	1.814050589	1.93E-05
ENSG00000197020	ZNF100	3.798102829	1.083197144	1.813760239	4.02E-05
ENSG00000110921	MVK	14.75387521	4.213812407	1.812248376	2.28E-05
ENSG00000147894	C9orf72	5.338665455	1.526979524	1.809778312	3.27E-05
ENSG00000151725	CENPU	18.38107028	5.260007897	1.809281655	2.75E-05
ENSG00000101188	NTSR1	0.335309258	0.095083337	1.809001925	0.006485
ENSG00000173991	TCAP	0.669197045	0.189763588	1.809001925	0.006485
ENSG00000164114	MAP9	3.708181585	1.0613363	1.808793584	3.38E-05
ENSG00000082196	C1QTNF3	1.226921792	0.350494244	1.807694582	0.000458
ENSG00000155008	APOOL	11.18660586	3.206155192	1.807147541	2.53E-05
ENSG00000136026	CKAP4	49.57284373	14.21149144	1.806941134	2.11E-05
ENSG00000159167	STC1	351.3845208	100.7790606	1.806398322	1.95E-05
ENSG00000103653	CSK	50.52656475	14.49659092	1.805801216	2.09E-05
ENSG00000130066	SAT1	51.65471255	14.83741593	1.804066731	2.33E-05
ENSG00000170088	TMEM192	6.206002402	1.783356485	1.803321087	2.75E-05
ENSG00000196604	POTEF	4.070333764	1.169860636	1.802236831	6.01E-05
ENSG00000280128	AL662795.2	1.725087417	0.495220723	1.802051847	0.000265
ENSG00000198908	BHLHB9	2.173328084	0.624639433	1.801752809	9.13E-05
ENSG00000102393	GLA	23.78556428	6.847281953	1.800682565	2.99E-05

ENSG00000136866	ZFP37	1.690094562	0.486096225	1.800546044	9.47E-05
ENSG00000001497	LAS1L	21.09832565	6.078942489	1.799629386	2.47E-05
ENSG00000225439	BOLA3-AS1	4.579539587	1.317998138	1.799111178	0.000147
ENSG00000063587	ZNF275	5.03148732	1.450529544	1.798491509	3.35E-05
ENSG00000168297	PXK	7.414302992	2.140336052	1.796545563	3.51E-05
ENSG00000182518	FAM104B	11.65444545	3.367533785	1.795012713	4.03E-05
ENSG00000182952	HMGN4	49.1323432	14.23003485	1.791984517	3.08E-05
ENSG00000175741	RWDD4P2	14.54697753	4.213935105	1.789699198	0.000167
ENSG00000260822	AC004656.1	1.249090429	0.361649137	1.789676336	0.000247
ENSG00000267534	S1PR2	1.949911235	0.565221963	1.788384463	0.000222
ENSG00000158486	DNAH3	0.175968142	0.050867184	1.787793973	0.002064
ENSG00000137822	TUBGCP4	8.779021836	2.552511744	1.786505107	2.86E-05
ENSG00000155545	MIER3	11.0064748	3.200139901	1.786401931	3.22E-05
ENSG00000035115	SH3YL1	7.623552458	2.217107608	1.786117631	3.02E-05
ENSG00000122035	RASL11A	0.952008422	0.275634987	1.780460341	0.005615
ENSG00000112812	PRSS16	1.856253484	0.541557619	1.779833586	0.000127
ENSG00000146950	SHROOM2	1.066189624	0.311500177	1.777550287	0.000167
ENSG00000285763	AL358777.1	1.193181317	0.348602334	1.775150776	0.000656
ENSG00000058668	ATP2B4	10.20887209	2.993110716	1.774472972	3.23E-05
ENSG00000166821	PEX11A	3.175746967	0.931682766	1.771874565	0.000135
ENSG00000281344	HELLPAR	0.059515305	0.017467513	1.771602592	0.000111
ENSG00000170271	FAXDC2	0.908379364	0.266520942	1.770738308	0.000306
ENSG00000280239	AC011498.7	0.907034945	0.265001215	1.769480982	0.005682
ENSG00000283078	AL137077.2	0.857537236	0.250539862	1.76609857	0.008204
ENSG00000205832	C16orf96	0.360690524	0.105380094	1.76609857	0.008204
ENSG00000166823	MESP1	0.582068331	0.170058294	1.76609857	0.008204
ENSG00000187630	DHRS4L2	12.48913314	3.682816473	1.765754176	5.18E-05
ENSG00000174516	PELI3	5.789935103	1.710256825	1.763071152	6.41E-05
ENSG00000206535	LNP1	2.025173025	0.597107007	1.76244625	0.000543
ENSG00000164849	GPR146	0.674385439	0.198317629	1.76156	0.004472
ENSG00000085871	MGST2	21.02404358	6.231949412	1.758564342	4.10E-05
ENSG00000242516	LINC00960	5.884904728	1.744931507	1.75741537	7.73E-05
ENSG00000249464	LINC01091	0.311551118	0.092069669	1.755575639	0.003084
ENSG00000250033	SLC7A11-AS1	0.63753163	0.188904589	1.754683403	0.000853
ENSG00000180747	SMG1P3	1.399315785	0.415698051	1.753812729	0.000151
ENSG00000112033	PPARD	7.753324608	2.309965908	1.750967504	5.67E-05
ENSG00000272009	AL121944.1	3.959262827	1.174542559	1.750894994	0.002232
ENSG00000151726	ACSL1	23.86505136	7.130471846	1.747264582	3.86E-05
ENSG00000149573	MPZL2	8.132030707	2.429809692	1.746854737	5.67E-05
ENSG00000100612	DHRS7	5.248911302	1.571119428	1.743709574	9.83E-05
ENSG00000158423	RIBC1	0.851624623	0.254250943	1.741348119	0.002822
ENSG00000213366	GSTM2	0.165014944	0.049103907	1.741071317	0.007013
ENSG00000181007	ZFP82	3.31789617	0.995928773	1.73997964	7.69E-05

ENSG00000198093	ZNF649	5.466092184	1.640801721	1.739902272	8.03E-05
ENSG00000187325	TAF9B	15.46555737	4.651130551	1.737541401	5.83E-05
ENSG00000101945	SUV39H1	6.364589145	1.913862697	1.737289291	8.52E-05
ENSG00000091513	TF	0.068581793	0.020502976	1.736412271	0.00689
ENSG00000133027	PEMT	51.89169931	15.64106762	1.734518734	4.81E-05
ENSG00000099954	CECR2	0.194188357	0.058247394	1.733063703	0.004472
ENSG00000100241	SBF1	14.47287618	4.371528275	1.731566747	4.54E-05
ENSG00000142910	TINAGL1	71.75766956	21.68226964	1.731126539	4.23E-05
ENSG00000129680	MAP7D3	2.453793981	0.741090317	1.730808544	0.000106
ENSG00000072506	HSD17B10	202.4814264	61.22310403	1.730118514	4.39E-05
ENSG00000083097	DOP1A	3.60656902	1.096239721	1.722126622	7.08E-05
ENSG00000174010	KLHL15	7.788136469	2.369476481	1.720900817	6.42E-05
ENSG00000278635	AC141557.2	3.380980456	1.021565024	1.720215913	0.006069
ENSG00000096654	ZNF184	6.357491707	1.935625069	1.719315742	0.000109
ENSG00000132326	PER2	3.056286423	0.930802158	1.718941283	0.000102
ENSG00000228532	AC005000.1	8.705302241	2.642267616	1.717552158	0.003301
ENSG00000197124	ZNF682	1.774704128	0.540617415	1.717434002	0.000254
ENSG00000144649	FAM198A	0.767313877	0.233405533	1.716269168	0.00141
ENSG00000142599	RERE	6.811068432	2.081915174	1.714300125	5.98E-05
ENSG00000163235	TGFA	25.04769615	7.657443189	1.714157361	5.57E-05
ENSG00000104450	SPAG1	3.794168526	1.160033428	1.713249229	0.000121
ENSG00000109674	NEIL3	9.743819204	2.980174054	1.71303252	8.89E-05
ENSG00000155158	TTC39B	1.964682891	0.60133945	1.711863218	0.000101
ENSG00000270055	AC127502.2	2.488287157	0.761601394	1.709577837	0.000445
ENSG00000164543	STK17A	7.096724918	2.17714341	1.708785812	7.97E-05
ENSG00000008083	JARID2	4.869759099	1.495825023	1.706864104	9.17E-05
ENSG00000081386	ZNF510	6.492588194	1.995856139	1.70588482	8.18E-05
ENSG00000140465	CYP1A1	1.994996776	0.612479398	1.705639153	0.000425
ENSG00000147099	HDAC8	0.836218017	0.257057866	1.705427014	0.000128
ENSG00000138796	HADH	15.31257334	4.718535279	1.702767844	5.80E-05
ENSG00000244701	AC004918.1	2.279206405	0.697607095	1.702567759	0.008356
ENSG00000011347	SYT7	0.294807481	0.09023307	1.702567759	0.008356
ENSG00000088899	LZTS3	4.492226402	1.385623979	1.700728828	0.000112
ENSG00000162407	PLPP3	8.260650027	2.548940543	1.700513403	8.07E-05
ENSG00000087842	PIR	19.62851588	6.058702813	1.700072113	7.93E-05
ENSG00000101843	PSMD10	45.21800914	13.96230766	1.699759606	6.47E-05
ENSG00000182359	KBTBD3	3.310413702	1.022507862	1.698499071	0.000131
ENSG00000198682	PAPSS2	0.992698683	0.306972181	1.693508019	0.001109
ENSG00000214193	SH3D21	1.774527413	0.550284244	1.691555093	0.000359
ENSG00000100902	PSMA6	0.732176666	0.227049343	1.690100067	0.000829
ENSG00000104361	NIPAL2	1.029896804	0.319793691	1.68883854	0.000526
ENSG00000111247	RAD51AP1	44.54610016	13.85941853	1.688837427	7.15E-05
ENSG00000137404	NRM	16.9593761	5.275262784	1.688826007	9.85E-05

ENSG00000124762	CDKN1A	13.3028435	4.138754227	1.688596542	9.42E-05
ENSG00000169246	NPIPB3	0.67324449	0.208774366	1.688285193	0.001942
ENSG00000171169	NAIF1	8.897150659	2.769107793	1.687942218	0.000106
ENSG00000232593	KANTR	1.591105106	0.496015253	1.685037601	0.000174
ENSG00000002586	CD99	22.14322645	6.907664179	1.684993997	7.36E-05
ENSG00000184613	NELL2	1.431008588	0.445958874	1.684730362	0.000295
ENSG00000106078	COBL	0.690163205	0.215252311	1.683725871	0.000256
ENSG00000141527	CARD14	0.189797649	0.058856603	1.682866467	0.007499
ENSG00000070731	ST6GALNAC2	0.512539422	0.158939426	1.682866467	0.007499
ENSG00000197472	ZNF695	1.412873335	0.440957272	1.681237848	0.000672
ENSG00000116990	MYCL	3.111419106	0.972534745	1.681217196	0.00018
ENSG00000118276	B4GALT6	2.535792526	0.793014222	1.68029901	0.000197
ENSG00000164465	DCBLD1	5.999113696	1.877438463	1.680121201	0.0001
ENSG00000147123	NDUFB11	77.17637203	24.18092484	1.678690167	7.87E-05
ENSG00000173894	CBX2	7.531226838	2.361339298	1.677436903	9.97E-05
ENSG00000276672	AL161891.1	1.018497829	0.318633094	1.677403458	0.000829
ENSG00000183617	MRPL54	58.0330338	18.20872758	1.676507443	9.24E-05
ENSG00000113328	CCNG1	77.64589736	24.37681642	1.675885832	7.33E-05
ENSG00000204619	PPP1R11	21.56774645	6.772670935	1.675323599	9.41E-05
ENSG00000099194	SCD	146.5763788	46.0654433	1.67443105	7.12E-05
ENSG00000120662	MTRF1	4.340639118	1.363521289	1.674103946	0.000179
ENSG00000279382	AC018665.1	3.558819595	1.117434763	1.672249081	0.000884
ENSG00000141404	GNAL	0.573962379	0.180218608	1.672249081	0.000884
ENSG00000260257	AL035071.1	2.607914102	0.819221847	1.672061622	0.000639
ENSG00000242951	AC007182.2	6.028746627	1.892967844	1.668749368	0.004523
ENSG00000188833	ENTPD8	1.001907846	0.31567787	1.664823594	0.002956
ENSG00000106484	MEST	23.16765292	7.33913514	1.662835041	9.03E-05
ENSG00000177181	RIMKLA	0.897961474	0.284099538	1.662722025	0.000425
ENSG00000019485	PRDM11	4.241493029	1.345462561	1.660742288	0.000104
ENSG00000119004	CYP20A1	8.291948819	2.631414445	1.659771151	0.000149
ENSG00000187626	ZKSCAN4	4.099372643	1.300486491	1.659182251	0.000317
ENSG00000227354	RBM26-AS1	2.107672857	0.66899782	1.658008389	0.000459
ENSG00000178947	SMIM10L2A	2.47676668	0.787420119	1.65651553	0.000253
ENSG00000112294	ALDH5A1	1.376461907	0.437503816	1.6559508	0.000504
ENSG00000188760	TMEM198	3.112027364	0.989719919	1.655232511	0.000453
ENSG00000064666	CNN2	70.59391105	22.52278894	1.652650345	9.08E-05
ENSG00000153395	LPCAT1	47.83385293	15.27488116	1.651374125	9.10E-05
ENSG00000174669	SLC29A2	10.00085605	3.192663846	1.651235164	0.000159
ENSG00000109775	UFSP2	8.654591743	2.768521027	1.648216846	0.00017
ENSG00000100647	SUSD6	6.214062746	1.988618969	1.64786841	0.000141
ENSG00000164930	FZD6	46.80176071	14.98711168	1.647310122	9.74E-05
ENSG00000175634	RPS6KB2	13.78015552	4.413265545	1.647023813	0.00011
ENSG00000142731	PLK4	13.499628	4.324505089	1.646595512	0.000121

ENSG00000275993	SIK1B	4.129856244	1.322589122	1.646447853	0.000197
ENSG00000163945	UVSSA	1.576259634	0.505156642	1.645592861	0.00017
ENSG00000025708	TYMP	0.465681228	0.148311549	1.644524337	0.00927
ENSG00000175866	BAIAP2	14.84504496	4.768699618	1.642762405	0.000103
ENSG00000197279	ZNF165	4.8984368	1.572126732	1.642559652	0.00039
ENSG00000214717	ZBED1	16.1166176	5.177648176	1.642521552	0.000118
ENSG00000175854	SWI5	35.98261982	11.56403272	1.641934353	0.000123
ENSG00000165891	E2F7	9.838110253	3.167436521	1.639356934	0.000126
ENSG00000102078	SLC25A14	4.068839173	1.309124205	1.639227103	0.000306
ENSG00000184260	HIST2H2AC	13.92848359	4.476312196	1.639179285	0.000829
ENSG00000100605	ITPK1	30.70919535	9.898965493	1.637799063	0.000106
ENSG00000140564	FURIN	26.5401245	8.558769512	1.637125862	0.000111
ENSG00000204839	MROH6	2.644768775	0.853017751	1.635643391	0.000343
ENSG00000183943	PRKX	10.58236617	3.420368743	1.633755641	0.000131
ENSG00000130203	APOE	2.975139351	0.960231279	1.633547562	0.000766
ENSG00000126003	PLAGL2	8.855146357	2.86389825	1.632769119	0.00014
ENSG00000101856	PGRMC1	158.2142185	51.2011739	1.63213357	0.000109
ENSG00000173175	ADCY5	4.252480788	1.377249216	1.630639507	0.000157
ENSG00000211455	STK38L	8.543044766	2.768186129	1.630090486	0.000137
ENSG00000162063	CCNF	15.58427098	5.051726555	1.629633351	0.000126
ENSG00000232040	ZBED9	1.06018221	0.343995692	1.626536081	0.000499
ENSG00000259032	ENSAP2	21.13014271	6.867069846	1.623912572	0.000657
ENSG00000048392	RRM2B	23.06183313	7.511619781	1.622741675	0.000127
ENSG00000078061	ARAF	17.7189224	5.77425279	1.621883429	0.000145
ENSG00000176945	MUC20	0.64905619	0.210699562	1.621837715	0.003928
ENSG00000080546	SESN1	2.415249644	0.786698101	1.621539682	0.000343
ENSG00000180758	GPR157	5.055946201	1.64986935	1.619592153	0.000207
ENSG00000234129	AC073529.1	0.759735468	0.24706918	1.618237726	0.006203
ENSG00000128567	PODXL	38.53938868	12.60712891	1.616590146	0.000127
ENSG00000135821	GLUL	26.8746328	8.792048091	1.616464537	0.000127
ENSG00000250461	AC122718.1	11.40113777	3.730260163	1.614493368	0.000537
ENSG00000213139	CRYGS	1.84547551	0.603500947	1.613078974	0.001837
ENSG00000269293	ZSCAN16-AS1	2.337512502	0.765712211	1.611489046	0.001158
ENSG00000225544	AC245452.2	6.289136721	2.055449478	1.611461029	0.00555
ENSG00000081320	STK17B	9.239791923	3.034096109	1.610887496	0.000163
ENSG00000128791	TWSG1	19.11672595	6.28803269	1.608523624	0.000155
ENSG00000177576	C18orf32	1.921839547	0.631674718	1.608344741	0.000442
ENSG00000151366	NDUFC2	12.9831114	4.270388332	1.608290713	0.000203
ENSG00000084112	SSH1	12.49223148	4.110804693	1.608007615	0.00014
ENSG00000116035	VAX2	1.608761022	0.529114917	1.605512119	0.001376
ENSG00000168778	TCTN2	5.687600524	1.876088543	1.604102318	0.000219
ENSG00000273045	C2orf15	3.365939551	1.109476807	1.603438099	0.000784
ENSG00000186654	PRR5	4.316316812	1.424707713	1.602991289	0.000261

ENSG00000257588	AC025154.2	3.451149046	1.137563455	1.602764874	0.001042
ENSG00000188343	FAM92A	2.46558365	0.814094229	1.602352214	0.000307
ENSG00000089094	KDM2B	6.067064641	2.004367936	1.602216507	0.000167
ENSG00000185619	PCGF3	11.21594658	3.707652526	1.601340239	0.000167
ENSG00000138767	CNOT6L	6.744913158	2.231704703	1.599960399	0.00018
ENSG00000051620	HEBP2	8.95748365	2.965189423	1.599355831	0.000166
ENSG00000215305	VPS16	29.21503573	9.673837594	1.598947055	0.000164
ENSG00000224546	EIF4BP3	12.06131389	3.992386553	1.598912755	0.000276
ENSG00000198040	ZNF84	9.371001824	3.103325682	1.598768103	0.000168
ENSG00000218426	AL590867.2	24.2112567	8.011296995	1.598667555	0.00049
ENSG00000148926	ADM	4.227740603	1.399876883	1.597770474	0.000465
ENSG00000139266	9-Mar	6.771225354	2.246538705	1.595689423	0.000252
ENSG00000106070	GRB10	6.633626945	2.20273862	1.594814457	0.000184
ENSG00000113361	CDH6	0.664975668	0.220579242	1.594650143	0.000653
ENSG00000156709	AIFM1	18.30167353	6.079443969	1.594261442	0.000189
ENSG00000213516	RBMXL1	9.298433312	3.088629017	1.594245691	0.000202
ENSG00000137310	TCF19	34.19131711	11.36158381	1.593893088	0.000165
ENSG00000168939	SPRY3	1.672529962	0.555878663	1.592706474	0.00037
ENSG00000094841	UPRT	6.049645791	2.012359819	1.591601931	0.000329
ENSG00000007866	TEAD3	5.871363403	1.954528963	1.590555824	0.000348
ENSG00000112763	BTN2A1	7.734777127	2.576805014	1.589799099	0.000244
ENSG00000197705	KLHL14	15.21753796	5.075305895	1.588519534	0.00019
ENSG00000145439	CBR4	5.568667704	1.857100167	1.588288495	0.000256
ENSG00000069011	PITX1	26.59939688	8.87357815	1.588195346	0.000182
ENSG00000167081	PBX3	2.103573145	0.701740265	1.586752061	0.000642
ENSG00000018610	CXorf56	23.0953693	7.720771036	1.585082424	0.000206
ENSG00000120549	KIAA1217	9.310573279	3.114820738	1.584123222	0.000187
ENSG00000134291	TMEM106C	120.7148605	40.39630229	1.583828009	0.000168
ENSG00000104369	JPH1	9.649404001	3.230960836	1.582665875	0.000233
ENSG00000157796	WDR19	6.131483927	2.053261652	1.582590286	0.000217
ENSG00000213463	SYNJ2BP	13.15192754	4.412010172	1.580155663	0.000196
ENSG00000145555	MYO10	11.24313819	3.773271829	1.57961516	0.000183
ENSG00000129355	CDKN2D	9.230052995	3.097381693	1.578731476	0.000471
ENSG00000180257	ZNF816	4.229041089	1.419978189	1.577914462	0.000467
ENSG00000168214	RBPJ	10.85166305	3.647616336	1.577293393	0.000198
ENSG00000174013	FBXO45	18.61326471	6.262043166	1.576044042	0.000199
ENSG00000089163	SIRT4	1.641327035	0.549465491	1.575612828	0.009406
ENSG00000228775	WEE2-AS1	0.572403435	0.191622954	1.575612828	0.009406
ENSG00000100092	SH3BP1	0.572286427	0.192155675	1.573514262	0.00474
ENSG00000182621	PLCB1	4.055674924	1.370247186	1.569845785	0.000226
ENSG00000271851	AC087501.4	1.566514371	0.527955946	1.569500968	0.002726
ENSG00000196705	ZNF431	2.531017963	0.855225229	1.569470088	0.000271
ENSG00000169188	APEX2	18.02762128	6.110959331	1.565034563	0.000247

ENSG00000136383	ALPK3	0.374312082	0.126842485	1.562044421	0.002266
ENSG00000145604	SKP2	42.286397	14.40772886	1.55781438	0.000222
ENSG00000066379	ZNRD1	2.719695733	0.926469845	1.556672234	0.000764
ENSG00000185825	BCAP31	97.6807353	33.3221089	1.556107515	0.000217
ENSG00000125386	FAM193A	5.824383853	1.987613836	1.555252537	0.000291
ENSG00000126767	ELK1	22.76188033	7.785463531	1.552127359	0.000261
ENSG00000128011	LRFN1	2.014501175	0.688459728	1.551327354	0.001185
ENSG00000226332	AL354836.1	5.767742102	1.971943699	1.549123106	0.002677
ENSG00000080224	EPHA6	0.624357395	0.21346267	1.549123106	0.002677
ENSG00000008283	CYB561	17.82950624	6.117065727	1.547754153	0.000259
ENSG00000223509	AC135983.2	1.424170317	0.487581441	1.547266987	0.002517
ENSG00000173511	VEGFB	44.38182828	15.23974249	1.546469448	0.000282
ENSG00000188419	CHM	9.947305687	3.427783313	1.541413884	0.000278
ENSG00000113369	ARRDC3	17.69148898	6.096639229	1.541355956	0.000281
ENSG00000260404	AC110079.1	1.587506949	0.546632426	1.540558802	0.001197
ENSG00000132670	PTPRA	52.86528051	18.22977891	1.540517514	0.000253
ENSG00000156869	FRRS1	8.688586653	2.997025599	1.53971531	0.000357
ENSG00000092931	MFSD11	2.81656902	0.971781758	1.538991751	0.000491
ENSG00000198546	ZNF511	5.468257302	1.88700844	1.538542943	0.000566
ENSG00000133958	UNC79	0.280506502	0.096587892	1.538447063	0.003682
ENSG00000005249	PRKAR2B	2.970850714	1.026296682	1.536831054	0.000631
ENSG00000109062	SLC9A3R1	83.31820333	28.81082148	1.536501158	0.000265
ENSG00000083828	ZNF586	4.08756786	1.413861258	1.535050439	0.000667
ENSG00000143178	TBX19	1.065546181	0.368218716	1.533714342	0.002982
ENSG00000102312	PORCN	6.050633189	2.096180736	1.533070603	0.000527
ENSG00000115129	TP53I3	11.72956641	4.065854503	1.532532763	0.000418
ENSG00000215414	PSMA6P1	3.496218552	1.208181429	1.531821608	0.006956
ENSG00000204604	ZNF468	3.727755316	1.293025452	1.531765617	0.000358
ENSG00000197779	ZNF81	2.276596867	0.789968633	1.53080324	0.000511
ENSG00000167103	PIP5KL1	1.275700682	0.44213799	1.52982738	0.002481
ENSG00000145390	USP53	4.496743906	1.562026934	1.529744944	0.000338
ENSG00000141524	TMC6	13.26009632	4.611081775	1.528343429	0.000298
ENSG00000146233	CYP39A1	0.913343186	0.316550525	1.528108327	0.005655
ENSG00000158373	HIST1H2BD	5.181203998	1.798747725	1.52760027	0.002222
ENSG00000177853	ZNF518A	6.518545665	2.268776359	1.526986334	0.000332
ENSG00000180596	HIST1H2BC	3.557274585	1.234474586	1.526489601	0.005142
ENSG00000253293	HOXA10	8.183500742	2.849769836	1.525947321	0.00042
ENSG00000173020	GRK2	33.44089938	11.65251562	1.525463525	0.00029
ENSG00000164109	MAD2L1	30.46674045	10.62228573	1.524614422	0.000297
ENSG00000198856	OSTC	124.3743813	43.3789809	1.524105609	0.000295
ENSG00000187446	CHP1	47.72004263	16.6473484	1.523782421	0.000297
ENSG00000275223	AL121906.2	7.108728137	2.47652127	1.522993021	0.001906
ENSG00000196182	STK40	19.41266827	6.782533864	1.521524918	0.000321

ENSG00000147100	SLC16A2	2.193639529	0.76606638	1.52080731	0.00102
ENSG00000214357	NEURL1B	8.989335764	3.142616439	1.520553331	0.000358
ENSG00000257093	KIAA1147	31.31636624	10.96562612	1.518422556	0.000308
ENSG00000130340	SNX9	19.98708859	6.998762869	1.518296852	0.000336
ENSG00000184675	AMER1	3.672249729	1.288338107	1.515243254	0.000453
ENSG00000170325	PRDM10	4.351337311	1.526783212	1.51501892	0.000478
ENSG00000072501	SMC1A	39.48972206	13.86358804	1.514698536	0.000311
ENSG00000101247	NDUFAF5	5.778284586	2.028202976	1.51464948	0.000413
ENSG00000042286	AIFM2	7.413153971	2.606460266	1.512173761	0.000432
ENSG00000109171	SLAIN2	14.12276501	4.967581284	1.511807603	0.000356
ENSG00000152952	PLOD2	10.54746534	3.711721073	1.511114495	0.000365
ENSG00000128709	HOXD9	9.250896585	3.255499779	1.510439003	0.000654
ENSG00000176024	ZNF613	3.728714672	1.312122641	1.510331416	0.000738
ENSG00000170270	GON7	40.30087235	14.19696936	1.509475875	0.000416
ENSG00000135679	MDM2	12.28650788	4.330067127	1.509082516	0.000341
ENSG00000151135	TMEM263	29.11310139	10.26134486	1.50890214	0.000346
ENSG00000224032	EPB41L4A-AS1	14.6948834	5.180421112	1.508437311	0.000417
ENSG00000109270	LAMTOR3	16.87831624	5.952880867	1.507866784	0.000387
ENSG00000185753	CXorf38	9.597584917	3.385197209	1.507741045	0.0004
ENSG00000135124	P2RX4	2.504361493	0.883302198	1.50731866	0.00062
ENSG00000151131	C12orf45	4.585794631	1.621965017	1.503865598	0.000367
ENSG00000144485	HES6	52.63694975	18.63481955	1.502498889	0.000378
ENSG00000157514	TSC22D3	26.28467926	9.307648733	1.502176525	0.000371
ENSG00000165359	INTS6L	5.518723512	1.954069275	1.502050609	0.00047
ENSG00000102309	PIN4	6.115198104	2.165057507	1.501969312	0.000579
ENSG00000196511	TPK1	2.950027085	1.044578894	1.501791054	0.000569
ENSG00000138617	PARP16	5.308945561	1.879689644	1.501764733	0.000617
ENSG00000071553	ATP6AP1	26.29129611	9.321067779	1.500464175	0.000375
ENSG00000204576	PRR3	4.597278044	1.631171929	1.498705771	0.000633
ENSG00000137185	ZSCAN9	3.642834169	1.29275413	1.498117724	0.000855
ENSG00000108590	MED31	6.537048645	2.320104752	1.497973168	0.000833
ENSG00000204713	TRIM27	14.79181935	5.257676985	1.496738974	0.000391
ENSG00000100749	VRK1	33.51668262	11.92486959	1.495308844	0.000416
ENSG00000160113	NR2F6	49.12444996	17.49709476	1.493775254	0.000397
ENSG00000137193	PIM1	4.519622151	1.608886144	1.493646037	0.000887
ENSG00000165169	DYNLT3	17.84996814	6.364624686	1.492082952	0.000459
ENSG00000050405	LIMA1	17.5754223	6.271230479	1.491168867	0.000412
ENSG00000180178	FAR2P1	3.896525625	1.39139859	1.489762081	0.00057
ENSG00000140577	CRTC3	2.782090304	0.99344824	1.489741582	0.000564
ENSG00000104290	FZD3	2.00031896	0.714287868	1.489699063	0.000604
ENSG00000120696	KBTBD7	5.719681473	2.042423816	1.489689615	0.000587
ENSG00000182141	ZNF708	4.028793749	1.438629816	1.489401807	0.000757
ENSG00000113161	HMGCR	50.27700736	17.96286825	1.489392949	0.000391

ENSG00000126561	STAT5A	1.87359164	0.669035191	1.488904535	0.001075
ENSG00000157216	SSBP3	9.249237905	3.305637091	1.488672268	0.000492
ENSG00000138376	BARD1	8.257912553	2.958630609	1.485124932	0.000513
ENSG00000130338	TULP4	4.078205573	1.46113243	1.485124932	0.000513
ENSG00000151466	SCLT1	2.952999776	1.058076689	1.484722181	0.000663
ENSG00000179611	DGKZP1	6.517687384	2.33813137	1.48278881	0.000777
ENSG00000125650	PSPN	2.553495968	0.915635325	1.482786223	0.001271
ENSG00000091490	SEL1L3	17.38038177	6.240178839	1.482250156	0.000438
ENSG00000258289	CHURC1	11.25825616	4.04226564	1.482074822	0.00049
ENSG00000069509	FUNDC1	34.07948486	12.25466455	1.479837369	0.000536
ENSG00000166908	PIP4K2C	33.50182396	12.04900039	1.479757124	0.000455
ENSG00000181544	FANCB	3.465645512	1.245842526	1.479441071	0.00096
ENSG00000198176	TFDP1	90.31733623	32.51039892	1.478617448	0.000428
ENSG00000082512	TRAF5	0.648410896	0.233192943	1.477564693	0.002355
ENSG00000158470	B4GALT5	57.56339627	20.77001275	1.47515557	0.000445
ENSG00000205208	C4orf46	13.42063539	4.848338143	1.473171483	0.000565
ENSG00000196220	SRGAP3	1.276262447	0.461256013	1.472270377	0.000726
ENSG00000176973	FAM89B	2.808624262	1.013157081	1.472186319	0.003714
ENSG00000114268	PFKFB4	5.980102172	2.162292612	1.471787264	0.000615
ENSG00000108423	TUBD1	6.876404509	2.486840109	1.47129495	0.000737
ENSG00000123136	DDX39A	51.29599346	18.58135844	1.469477192	0.000475
ENSG00000109929	SC5D	19.56393904	7.093257918	1.468107225	0.000503
ENSG00000109670	FBXW7	2.248392438	0.814975271	1.46807137	0.000736
ENSG00000009724	MASP2	0.756875961	0.273780562	1.46731789	0.005702
ENSG00000173801	JUP	53.40285451	19.41063747	1.464574393	0.000488
ENSG00000139263	LRIG3	7.353048097	2.673785666	1.463699712	0.000624
ENSG00000100908	EMC9	25.15547287	9.161218285	1.461476118	0.000653
ENSG00000168904	LRRC28	2.314659089	0.844223121	1.459026404	0.000837
ENSG00000145725	PPIP5K2	6.677929594	2.441624834	1.45599861	0.000554
ENSG00000070961	ATP2B1	14.69731593	5.375535556	1.45553203	0.000547
ENSG00000197046	SIGLEC15	10.33603423	3.782098131	1.454616618	0.000699
ENSG00000149823	VPS51	20.3958914	7.469696859	1.453577859	0.000579
ENSG00000109458	GAB1	1.304892029	0.477756416	1.453115447	0.001218
ENSG00000088832	FKBP1A	286.2716853	104.898911	1.45292388	0.000524
ENSG00000232531	AC027612.3	6.305731824	2.3086997	1.452092241	0.002349
ENSG00000119669	IRF2BPL	15.79142245	5.793254658	1.451046468	0.000632
ENSG00000146426	TIAM2	0.286251354	0.104804396	1.450050505	0.005877
ENSG00000102934	PLLP	3.090781621	1.134478206	1.4499995	0.000843
ENSG00000187605	TET3	7.767306102	2.852641298	1.449531534	0.0006
ENSG00000180776	ZDHHC20	46.17089903	16.99296126	1.446551286	0.000569
ENSG00000177685	CRACR2B	2.829835025	1.041002836	1.446088165	0.001386
ENSG00000133812	SBF2	2.225467139	0.819602698	1.445309026	0.000749
ENSG00000135913	USP37	5.806783308	2.139378488	1.444855877	0.000694

ENSG00000116133	DHCR24	127.176577	46.89097148	1.443991098	0.000565
ENSG00000156500	FAM122C	0.351755899	0.12935693	1.442987997	0.008675
ENSG00000112759	SLC29A1	34.49574757	12.73433642	1.442163773	0.000608
ENSG00000105516	DBP	7.630442185	2.818216114	1.44127392	0.000722
ENSG00000205978	NYNRIN	0.336174588	0.123794932	1.440816343	0.009693
ENSG00000133111	RFXAP	11.92316852	4.406430479	1.440157141	0.000887
ENSG00000279528	AC115618.3	8.84868369	3.265074778	1.439356382	0.005279
ENSG00000170370	EMX2	16.24278098	6.010498902	1.438547665	0.000736
ENSG00000077721	UBE2A	17.83563971	6.600778788	1.438470272	0.000664
ENSG00000279541	AC005261.5	2.621005203	0.968200187	1.437636951	0.005716
ENSG00000054654	SYNE2	4.557589485	1.687855335	1.437543655	0.000635
ENSG00000147889	CDKN2A	53.58074316	19.87404029	1.435329135	0.000629
ENSG00000205559	CHKB-DT	2.895313526	1.072170586	1.433823919	0.006048
ENSG00000204764	RANBP17	2.496075718	0.926696643	1.43356576	0.000937
ENSG00000102221	JADE3	7.036094005	2.614159351	1.432618638	0.000843
ENSG00000139880	CDH24	4.593277938	1.707877919	1.431108985	0.00118
ENSG00000116711	PLA2G4A	11.84526691	4.406248179	1.430857311	0.000886
ENSG00000157625	TAB3	3.264334246	1.214953747	1.430126415	0.000839
ENSG00000187624	C17orf97	1.511060581	0.561603744	1.42927249	0.004683
ENSG00000019505	SYT13	2.811889662	1.048104072	1.42745243	0.001278
ENSG00000131269	ABCB7	12.7208116	4.743683445	1.427446666	0.000788
ENSG00000166086	JAM3	0.551926702	0.205297753	1.426940502	0.008
ENSG00000111832	RWDD1	16.05228858	5.992152559	1.426051231	0.000739
ENSG00000101966	XIAP	18.87174485	7.048021751	1.425417424	0.000695
ENSG00000071889	FAM3A	7.991528931	2.985378868	1.424792562	0.000881
ENSG00000158406	HIST1H4H	1.332961675	0.496678191	1.424256647	0.008869
ENSG00000145337	PYURF	62.17159279	23.25314271	1.423232054	0.000765
ENSG00000115271	GCA	7.70932443	2.883214187	1.423019992	0.000997
ENSG00000234127	TRIM26	21.65167976	8.099701301	1.422957189	0.000758
ENSG00000178184	PARD6G	3.769000175	1.409444891	1.422789691	0.00135
ENSG00000205683	DPF3	0.661143001	0.246915174	1.422628914	0.003954
ENSG00000221890	NPTXR	4.197328301	1.571043181	1.421767008	0.001078
ENSG00000112378	PERP	29.4251391	11.01861464	1.421559813	0.000733
ENSG00000213189	BTF3L4P2	40.09470998	15.00728944	1.421363499	0.00147
ENSG00000173542	MOB1B	6.566217293	2.46169197	1.419743522	0.000826
ENSG00000101849	TBL1X	12.27091438	4.603334694	1.418931668	0.000758
ENSG00000185507	IRF7	3.99092395	1.498917326	1.416120122	0.001845
ENSG00000169239	CA5B	1.412465941	0.530642643	1.4158812	0.00174
ENSG00000055609	KMT2C	6.250990421	2.350097285	1.415817982	0.000769
ENSG00000180817	PPA1	95.67583388	35.97670371	1.415622255	0.000723
ENSG00000165338	HECTD2	1.504999785	0.565836866	1.415115649	0.001308
ENSG00000166441	RPL27A	337.4147555	127.0705112	1.413445391	0.000724
ENSG00000149091	DGKZ	9.300720058	3.510670361	1.410024225	0.000827

ENSG00000087076	HSD17B14	3.979566739	1.502587232	1.407194923	0.00462
ENSG00000156697	UTP14A	27.48208751	10.39398159	1.407185627	0.000834
ENSG00000105204	DYRK1B	5.340997465	2.019388961	1.406993029	0.001422
ENSG00000165113	GKAP1	2.620087418	0.992970762	1.402903349	0.002498
ENSG00000175548	ALG10B	5.803027924	2.201208635	1.402874279	0.000934
ENSG00000183091	NEB	0.135334172	0.051254924	1.402225974	0.005308
ENSG00000231074	HCG18	1.525147937	0.578916651	1.401476502	0.001301
ENSG00000228343	AC115618.2	17.82845899	6.764205096	1.401153712	0.002467
ENSG00000204899	MZT1	62.6509129	23.81265688	1.40007529	0.000867
ENSG00000169860	P2RY1	1.170512849	0.444512994	1.39978446	0.002552
ENSG00000204568	MRPS18B	43.84373832	16.66762433	1.399725191	0.000934
ENSG00000112787	FBRSL1	7.111279528	2.706407566	1.398096097	0.000966
ENSG00000179981	TSHZ1	5.666593552	2.156803002	1.397873023	0.001026
ENSG00000244398	AC116533.1	1048.02076	399.8002201	1.394835353	0.000869
ENSG00000100968	NFATC4	0.445737889	0.169778346	1.393727843	0.00668
ENSG00000119630	PGF	1.317622399	0.503040787	1.392136126	0.002702
ENSG00000174132	FAM174A	17.02698099	6.512149099	1.390650281	0.00134
ENSG00000254198	AC113191.1	27.26941327	10.44102597	1.388430827	0.002075
ENSG00000089123	TASP1	7.752963153	2.973935469	1.386373481	0.001465
ENSG00000163376	KBTBD8	1.0852218	0.415873599	1.385884759	0.005121
ENSG00000153132	CLGN	4.663614427	1.789379675	1.385684838	0.001764
ENSG00000186416	NKRF	5.865751985	2.251923315	1.385245309	0.001358
ENSG00000153130	SCOC	19.94999675	7.666769221	1.384154975	0.000998
ENSG00000163584	RPL22L1	230.5924531	88.64098872	1.383832991	0.00094
ENSG00000120509	PDZD11	38.60579693	14.84606055	1.383061504	0.001145
ENSG00000125834	STK35	14.23252136	5.475630652	1.38252657	0.001039
ENSG00000137166	FOXP4	9.521935781	3.663756336	1.382275399	0.001123
ENSG00000167815	PRDX2	165.884047	63.90454296	1.380705723	0.000975
ENSG00000126945	HNRNPH2	59.84924661	23.0762653	1.379386569	0.001033
ENSG00000162636	FAM102B	3.684980959	1.421127442	1.378816763	0.001298
ENSG00000133983	COX16	3.678375697	1.417618999	1.378337995	0.003457
ENSG00000162783	IER5	7.203699964	2.77899038	1.378321455	0.001377
ENSG00000124486	USP9X	18.2949764	7.06296789	1.377609339	0.00101
ENSG00000155893	PXYLP1	1.72159193	0.665148079	1.376011289	0.00158
ENSG00000107438	PDLIM1	31.29709733	12.09543939	1.375945408	0.001142
ENSG00000183765	CHEK2	8.221308362	3.178151771	1.375400939	0.001321
ENSG00000161888	SPC24	20.17888446	7.804771177	1.374758644	0.001208
ENSG00000091129	NRCAM	0.550815169	0.213079672	1.371990511	0.005323
ENSG00000167513	CDT1	29.94874483	11.60674475	1.371965334	0.001133
ENSG00000138709	LARP1B	8.301657779	3.218910539	1.371227264	0.001176
ENSG00000172795	DCP2	17.55232481	6.810508155	1.370314066	0.001092
ENSG00000125249	RAP2A	13.2708191	5.151375436	1.369617965	0.001209
ENSG00000236814	AC046176.1	6.214246523	2.410321952	1.369214716	0.003323

ENSG00000167536	DHRS13	6.458009543	2.508802691	1.368000102	0.001845
ENSG00000110693	SOX6	0.293664301	0.113982282	1.367780164	0.004606
ENSG00000163939	PBRM1	12.49581483	4.859307211	1.367077455	0.00115
ENSG00000163644	PPM1K	1.407424821	0.54718793	1.366738454	0.001989
ENSG00000168566	SNRNP48	14.41297333	5.606895503	1.366504773	0.001218
ENSG00000198740	ZNF652	8.067769778	3.140023765	1.365778661	0.001251
ENSG00000115507	OTX1	1.547609287	0.602028332	1.365054254	0.00346
ENSG00000204138	PHACTR4	16.68108066	6.498661383	1.36445485	0.001173
ENSG00000180098	TRNAU1AP	10.09984683	3.935333617	1.364051102	0.001382
ENSG00000111707	SUDS3	40.42200995	15.75418199	1.363910267	0.001134
ENSG00000230330	HMGN2P3	13.46930281	5.246959351	1.361353332	0.008226
ENSG00000157978	LDLRAP1	10.67669753	4.171919649	1.360027469	0.001344
ENSG00000260920	AL031985.3	3.975504298	1.553640411	1.358790836	0.002871
ENSG00000165476	REEP3	16.91164204	6.618937241	1.35776512	0.001283
ENSG00000197077	KIAA1671	8.444021377	3.305701689	1.357443023	0.001229
ENSG00000151689	INPP1	2.580331154	1.010701611	1.355476426	0.003047
ENSG00000168528	SERINC2	17.36645248	6.81095685	1.354733923	0.001378
ENSG00000267023	LRRC37A16P	3.800668785	1.49014849	1.35451809	0.002206
ENSG00000103495	MAZ	24.69682321	9.696121452	1.353262885	0.001351
ENSG00000099889	ARVCF	6.69042976	2.627971935	1.352384444	0.001561
ENSG00000170846	AC093323.1	10.54214762	4.141739265	1.352098524	0.001551
ENSG00000218739	CEBPZOS	17.84197834	7.012698545	1.35164679	0.00137
ENSG00000147606	SLC26A7	0.672777065	0.264026066	1.351141749	0.006715
ENSG00000119720	NRDE2	1.860303017	0.731398577	1.350995404	0.001662
ENSG00000126768	TIMM17B	21.70821037	8.54378089	1.349634464	0.001457
ENSG00000155085	AK9	1.013808223	0.398958306	1.348855178	0.002981
ENSG00000167130	DOLPP1	22.92718819	9.036219373	1.347591963	0.001509
ENSG00000077713	SLC25A43	5.68765044	2.241748644	1.347086799	0.00213
ENSG00000198055	GRK6	31.20236185	12.30343444	1.347062643	0.001338
ENSG00000257621	PSMA3-AS1	7.224613635	2.851693017	1.345434592	0.00153
ENSG00000164142	FAM160A1	3.355036192	1.325104584	1.344055157	0.002299
ENSG00000142784	WDTC1	7.255574617	2.868287695	1.343183336	0.001628
ENSG00000102384	CENPI	5.686147893	2.247997562	1.342881269	0.001897
ENSG00000169282	KCNAB1	0.579337102	0.228649922	1.342804073	0.007835
ENSG00000108179	PPIF	113.0738675	44.72964169	1.342479835	0.001335
ENSG00000157869	RAB28	14.61832294	5.784338587	1.341742927	0.00179
ENSG00000151208	DLG5	8.42950579	3.338384911	1.340716884	0.001486
ENSG00000198948	MFAP3L	1.539287855	0.609405276	1.340459461	0.002582
ENSG00000088812	ATRN	16.94237001	6.714723996	1.339712275	0.00141
ENSG00000089289	IGBP1	30.40777969	12.06825307	1.337570601	0.001619
ENSG00000138381	ASNSD1	34.60983421	13.76501754	1.334595817	0.001555
ENSG00000138495	COX17	16.06392789	6.388843374	1.33441547	0.001871
ENSG00000198792	TMEM184B	16.34826251	6.505826624	1.333787711	0.001505

ENSG00000144320	LNPK	9.811931103	3.911007258	1.331421947	0.001589
ENSG00000069493	CLEC2D	0.72675572	0.289541539	1.329924867	0.007169
ENSG00000005022	SLC25A5	368.8262454	147.181145	1.32988409	0.001456
ENSG00000235823	OLMALINC	2.341449321	0.934126724	1.329620424	0.002485
ENSG00000102580	DNAJC3	19.77540739	7.894193928	1.329293986	0.001565
ENSG00000261609	GAN	2.182680414	0.87138645	1.328923335	0.001925
ENSG00000102316	MAGED2	40.83319729	16.30656586	1.328748465	0.001561
ENSG00000171681	ATF7IP	11.52592841	4.609016576	1.326831798	0.00156
ENSG00000054793	ATP9A	7.10407928	2.84176126	1.326220675	0.00176
ENSG00000272141	AL390719.2	5.334312863	2.131577236	1.325709279	0.006832
ENSG00000233901	LINC01503	3.302990461	1.319866206	1.325709279	0.006832
ENSG00000091542	ALKBH5	31.03126422	12.41860907	1.32565736	0.001626
ENSG00000196151	WDSUB1	7.80908424	3.123983687	1.32562001	0.002587
ENSG00000136122	BORA	10.96720674	4.393495508	1.323977312	0.002014
ENSG00000146707	POMZP3	4.984615835	1.996420808	1.323362275	0.00386
ENSG00000115137	DNAJC27	2.243813323	0.899401277	1.322546671	0.003163
ENSG00000196922	ZNF252P	10.88943098	4.374533344	1.32016751	0.001697
ENSG00000184792	OSBP2	3.872949988	1.556687674	1.319061805	0.002265
ENSG00000146414	SHPRH	2.241256246	0.900998373	1.318973772	0.001988
ENSG00000108100	CCNY	29.42370936	11.83186821	1.318789338	0.001652
ENSG00000176542	USF3	4.775306281	1.92242262	1.317047707	0.001855
ENSG00000264112	AC015813.1	3.364692181	1.355524684	1.315587335	0.002579
ENSG00000100592	DAAM1	2.508219272	1.011265451	1.314627747	0.002294
ENSG00000175305	CCNE2	6.253087696	2.524733738	1.312574347	0.002274
ENSG00000185900	POMK	14.87090096	6.009753188	1.311258174	0.002355
ENSG00000042445	RETSAT	56.20430043	22.73363048	1.310358182	0.001742
ENSG00000158526	TSR2	22.2819418	9.016821829	1.309404308	0.002184
ENSG00000086598	TMED2	193.3586276	78.36468218	1.30754506	0.001731
ENSG00000223482	NUTM2A-AS1	2.079818816	0.842861295	1.306842311	0.003117
ENSG00000147400	CETN2	30.68889952	12.44451938	1.306569492	0.002035
ENSG00000181894	ZNF329	3.714046795	1.506301389	1.305822315	0.003023
ENSG00000164118	CEP44	2.829833527	1.148577022	1.3048593	0.002683
ENSG00000070081	NUCB2	9.014545443	3.6604082	1.304666474	0.001981
ENSG00000197182	MIRLET7BHG	1.962340477	0.797108666	1.303267271	0.004033
ENSG00000127720	METTL25	3.209013375	1.304372018	1.302362524	0.003807
ENSG00000163697	APBB2	2.615622149	1.064143375	1.301682	0.002328
ENSG00000084676	NCOA1	6.021209399	2.452560719	1.300102383	0.002181
ENSG00000139726	DENR	81.85321101	33.3845767	1.298375387	0.001896
ENSG00000069974	RAB27A	6.217496152	2.53963832	1.295942523	0.002475
ENSG00000101868	POLA1	8.922934797	3.645481523	1.295765515	0.002211
ENSG00000188706	ZDHHC9	19.89252052	8.136420986	1.294208397	0.002069
ENSG00000110442	COMMD9	12.88046662	5.269445182	1.293885726	0.002124
ENSG00000184182	UBE2F	3.79598833	1.55266138	1.29358694	0.003264

ENSG00000139668	WDFY2	1.640769691	0.671571194	1.292892661	0.00266
ENSG00000242193	CRYZL2P	4.946777313	2.026917143	1.291344551	0.002673
ENSG00000170779	CDCA4	33.05589976	13.55540621	1.290460878	0.002207
ENSG00000170684	ZNF296	3.258498454	1.334680242	1.290288699	0.007315
ENSG00000111581	NUP107	18.14051059	7.440792989	1.290169161	0.00208
ENSG00000172671	ZFAND4	2.749042813	1.127697392	1.289450025	0.00321
ENSG00000079308	TNS1	0.857216421	0.351924305	1.28814682	0.003621
ENSG00000168393	DTYMK	47.88875201	19.68773785	1.286850933	0.002169
ENSG00000023697	DERA	19.02677874	7.822528697	1.286674234	0.002415
ENSG00000117525	F3	3.938732924	1.619257281	1.285967311	0.004458
ENSG00000175567	UCP2	13.07650961	5.379318642	1.285774635	0.002528
ENSG00000106546	AHR	1.292706681	0.531773259	1.284997888	0.004372
ENSG00000259959	AC107068.1	1.495875549	0.615519458	1.283899616	0.006883
ENSG00000185201	IFITM2	20.98750767	8.645113639	1.283786087	0.002727
ENSG00000140455	USP3	11.89410693	4.923712797	1.27686542	0.002414
ENSG00000145386	CCNA2	59.54202722	24.65331342	1.276617248	0.002297
ENSG00000147383	NSDHL	18.33870484	7.602554281	1.274590759	0.002789
ENSG00000223459	TCAF1P1	3.143732972	1.303001759	1.273623979	0.00623
ENSG00000101888	NXT2	15.61499384	6.48656206	1.271732869	0.002743
ENSG00000168769	TET2	3.439424015	1.429246215	1.271275241	0.002692
ENSG00000106588	PSMA2	1.393892473	0.578833474	1.270976381	0.006171
ENSG00000102241	HTATSF1	36.69551384	15.25885758	1.270418156	0.002464
ENSG00000169446	MMGT1	19.89783369	8.274830732	1.270218839	0.002577
ENSG00000009950	MLXIPL	1.744314767	0.725134686	1.269588411	0.005892
ENSG00000135597	REPS1	5.557177736	2.312279576	1.269387712	0.002747
ENSG00000197256	KANK2	14.50291044	6.036399008	1.269040232	0.002498
ENSG00000134690	CDCA8	36.84363155	15.33803207	1.26873821	0.002549
ENSG00000175906	ARL4D	25.14385064	10.46879465	1.268396627	0.002914
ENSG00000070423	RNF126	35.31922397	14.71190707	1.26793754	0.002499
ENSG00000145365	TIFA	8.366922022	3.486257263	1.267266892	0.002979
ENSG00000109685	NSD2	17.22466599	7.182108919	1.266521379	0.002418
ENSG00000170085	SIMC1	2.966344319	1.23713108	1.265578957	0.003954
ENSG00000133302	SLF1	4.105849441	1.713271891	1.265113226	0.003189
ENSG00000172943	PHF8	5.603839153	2.33870554	1.265046035	0.00286
ENSG00000120832	MTERF2	5.73887327	2.39493813	1.264811244	0.003684
ENSG00000215146	BX322639.1	3.913038188	1.633282613	1.264684551	0.00329
ENSG00000166816	LDHD	7.138421643	2.979148007	1.264613842	0.00387
ENSG00000171295	ZNF440	3.634365485	1.518338352	1.263300419	0.003438
ENSG00000188566	NDOR1	6.497667061	2.714829682	1.263025653	0.003853
ENSG00000119446	RBM18	11.85248777	4.956440425	1.262193678	0.002795
ENSG00000145996	CDKAL1	4.303846949	1.799473612	1.262059164	0.003674
ENSG00000175087	PDIK1L	7.758180714	3.247423842	1.260670764	0.003152
ENSG00000110092	CCND1	148.6541202	62.30692939	1.259036751	0.002528

ENSG00000115902	SLC1A4	9.97214389	4.180213724	1.258727256	0.002854
ENSG00000134470	IL15RA	9.049298224	3.793902467	1.258350724	0.003217
ENSG00000130741	EIF2S3	187.7577239	78.73513416	1.258333104	0.002545
ENSG00000182704	TSKU	12.0673064	5.060367455	1.258129238	0.003012
ENSG00000109805	NCAPG	28.18246516	11.82525067	1.257405932	0.002688
ENSG00000176055	MBLAC2	5.094350233	2.137210368	1.257351826	0.003379
ENSG00000203760	CENPW	50.30022186	21.11468017	1.256604874	0.003208
ENSG00000163808	KIF15	17.56016767	7.377233534	1.255595362	0.002824
ENSG00000154839	SKA1	18.34310465	7.708361341	1.255099186	0.003034
ENSG00000138658	ZGRF1	2.598106682	1.091663526	1.255032936	0.003757
ENSG00000243317	STMP1	15.14616643	6.373978016	1.253099457	0.002972
ENSG00000179163	FUCA1	18.18800031	7.653684752	1.253028258	0.003336
ENSG00000158769	F11R	16.50467567	6.953923605	1.251404069	0.002922
ENSG00000276180	HIST1H4I	6.490630296	2.732833566	1.251238144	0.006577
ENSG00000152990	ADGRA3	10.32213567	4.350491529	1.250935625	0.002899
ENSG00000215386	MIR99AHG	1.450142482	0.611141012	1.250522207	0.004358
ENSG00000176593	AC008969.1	0.68952889	0.290371908	1.250493297	0.008841
ENSG00000126822	PLEKHG3	6.234487489	2.630913969	1.249119489	0.003053
ENSG00000175137	SH3BP5L	14.81495957	6.253773636	1.248700476	0.002949
ENSG00000262919	CCNQ	20.53042378	8.668003386	1.24824727	0.003411
ENSG00000139428	MMAB	15.11317973	6.383548553	1.247821061	0.002969
ENSG00000165195	PIGA	3.754807156	1.586290036	1.247188609	0.003949
ENSG00000110536	PTPMT1	2.97378208	1.257941749	1.244482211	0.007137
ENSG00000165821	SALL2	10.79962133	4.57445505	1.243682673	0.0033
ENSG00000139579	NABP2	116.2569958	49.28383401	1.242646824	0.002925
ENSG00000179387	ELMOD2	10.02548162	4.255434863	1.240690313	0.003262
ENSG00000135093	USP30	6.859812072	2.911564311	1.240630314	0.003625
ENSG00000118997	DNAH7	0.282175244	0.119653815	1.240518121	0.009442
ENSG00000170581	STAT2	11.04657112	4.689539705	1.240480148	0.0033
ENSG00000183337	BCOR	8.636833572	3.66739061	1.240161804	0.003273
ENSG00000072310	SREBF1	27.49617955	11.67854801	1.239879189	0.003002
ENSG00000123411	IKZF4	2.074542062	0.881060274	1.239287133	0.005275
ENSG00000185000	DGAT1	11.18729328	4.756962126	1.238076655	0.00356
ENSG00000139323	POC1B	6.449846618	2.743315044	1.237714064	0.00341
ENSG00000165813	CCDC186	7.328582333	3.117458344	1.237560596	0.003355
ENSG00000144749	LRIG1	4.563598718	1.941517662	1.237214793	0.003814
ENSG00000119685	TTLL5	3.522432422	1.499889379	1.236028667	0.003629
ENSG00000105655	ISYNA1	62.3346295	26.56617683	1.234948701	0.003125
ENSG00000176225	RTTN	1.396957056	0.59535902	1.234622563	0.004157
ENSG00000171469	ZNF561	9.562775185	4.076925752	1.234334356	0.003486
ENSG00000102100	SLC35A2	10.9902988	4.685485663	1.234261248	0.003699
ENSG00000198171	DDRGK1	40.05199319	17.07937896	1.234032016	0.003386
ENSG00000118707	TGIF2	14.94072803	6.376142372	1.232865729	0.003551

ENSG00000151247	EIF4E	8.237083597	3.516261446	1.232557547	0.003279
ENSG00000182628	SKA2	49.70981991	21.24399753	1.230965533	0.003259
ENSG00000141905	NFIC	7.908581895	3.379727604	1.230933705	0.00347
ENSG00000185010	F8	0.600955873	0.256697313	1.230160283	0.008737
ENSG00000126106	TMEM53	2.161118224	0.923585735	1.229683831	0.008186
ENSG00000164040	PGRMC2	23.32253218	9.976389698	1.229594609	0.003369
ENSG00000114302	PRKAR2A	25.19453879	10.77980059	1.229274972	0.00329
ENSG00000237765	FAM200B	4.406686524	1.886425941	1.228103398	0.00462
ENSG00000040608	RTN4R	9.886345043	4.23634218	1.226858966	0.004121
ENSG00000241343	RPL36A	2.424483773	1.038901922	1.226090057	0.006721
ENSG00000178150	ZNF114	2.3035748	0.987091898	1.225663188	0.008439
ENSG00000107263	RAPGEF1	11.45787853	4.916144071	1.22516799	0.003606
ENSG00000213066	FGFR1OP	1.257529175	0.539415743	1.225162209	0.004867
ENSG00000168743	NPNT	3.019318168	1.295254633	1.224980242	0.004951
ENSG00000135916	ITM2C	75.10498675	32.24796339	1.224204546	0.003393
ENSG00000176244	ACBD7	15.73230589	6.753314795	1.224178014	0.004647
ENSG00000179818	PCBP1-AS1	1.524969812	0.654825221	1.223808335	0.004338
ENSG00000103226	NOMO3	2.833168706	1.217271264	1.222879731	0.004712
ENSG00000161981	SNRNP25	33.19621921	14.27738792	1.22174173	0.003588
ENSG00000111684	LPCAT3	7.746889918	3.334734165	1.220319599	0.004204
ENSG00000171848	RRM2	22.38029685	9.637147805	1.220021105	0.003593
ENSG00000172270	BSG	415.2133414	178.8638626	1.219541233	0.003392
ENSG00000125319	C17orf53	4.324550811	1.862026704	1.219437566	0.005949
ENSG00000145241	CENPC	3.701648546	1.59552751	1.21836744	0.004308
ENSG00000185236	RAB11B	33.14076844	14.29882722	1.217141192	0.003779
ENSG00000165240	ATP7A	1.729580929	0.746052033	1.217085288	0.005097
ENSG00000144026	ZNF514	1.889023754	0.814711159	1.2170359	0.006107
ENSG00000120685	PROSER1	8.277117997	3.573706524	1.216094197	0.004007
ENSG00000177595	PIDD1	4.221975175	1.822863546	1.21585292	0.004765
ENSG00000119314	PTBP3	33.58963956	14.522615	1.214234862	0.003613
ENSG00000153140	CETN3	12.99573259	5.620610935	1.21354168	0.004299
ENSG00000154447	SH3RF1	3.402869212	1.472471117	1.212533427	0.005303
ENSG00000116704	SLC35D1	9.364769149	4.053525163	1.212445585	0.004107
ENSG00000106799	TGFBR1	14.2622462	6.174223719	1.212323458	0.003858
ENSG00000099821	POLRMT	36.66420504	15.8819976	1.211482296	0.003737
ENSG00000139433	GLTP	44.26529947	19.17707933	1.211264309	0.003833
ENSG00000100697	DICER1	9.815876183	4.25275146	1.211192607	0.003832
ENSG00000092847	AGO1	6.608155687	2.865793996	1.209758332	0.003951
ENSG00000122335	SERAC1	1.537485923	0.66695303	1.208869756	0.005889
ENSG00000138439	FAM117B	7.636699487	3.320218201	1.206005591	0.004452
ENSG00000109133	TMEM33	16.59247511	7.219683142	1.205004303	0.003981
ENSG00000228589	SPCS2P4	17.97817485	7.822043338	1.204356664	0.006768
ENSG00000144579	CTDSP1	26.75789284	11.65716165	1.203224733	0.004052

ENSG00000113838	TBCCD1	17.04551868	7.427654599	1.202796214	0.004407
ENSG00000164096	C4orf3	32.06913883	13.97781096	1.202503771	0.004135
ENSG00000145388	METTL14	7.885770727	3.437797329	1.202137097	0.004496
ENSG00000089220	PEBP1	455.0858137	198.5420494	1.20123847	0.003907
ENSG00000138030	KHK	3.086114991	1.345819353	1.200793294	0.008002
ENSG00000175105	ZNF654	4.624355072	2.018496216	1.200206337	0.004947
ENSG00000187801	ZFP69B	3.87032046	1.688466461	1.200202312	0.008255
ENSG00000185630	PBX1	1.649865555	0.720456855	1.199633535	0.004972
ENSG00000132646	PCNA	196.0308604	85.63115241	1.199395083	0.004029
ENSG00000103657	HERC1	4.092048482	1.787584944	1.199223799	0.004424
ENSG00000130699	TAF4	3.817267932	1.670401982	1.196633443	0.004908
ENSG00000182809	CRIP2	45.62103896	19.97081191	1.196317693	0.004155
ENSG00000259781	HMGB1P6	284.2186852	124.4497942	1.195939766	0.004196
ENSG00000180182	MED14	15.79503886	6.916588439	1.195830029	0.004234
ENSG00000135482	ZC3H10	2.305506321	1.010131887	1.194582774	0.006145
ENSG00000111206	FOXM1	37.30752431	16.35558039	1.194185748	0.004251
ENSG00000151729	SLC25A4	15.86363418	6.958429157	1.193316346	0.004531
ENSG00000167232	ZNF91	4.62987778	2.032143269	1.192321113	0.004879
ENSG00000123737	EXOSC9	15.64626091	6.869823218	1.191894341	0.004609
ENSG00000189308	LIN54	6.686412998	2.936587788	1.191427825	0.004964
ENSG00000148730	EIF4EBP2	36.03325766	15.84628088	1.189703609	0.004339
ENSG00000165912	PACSIN3	16.53501304	7.270754185	1.189685069	0.005001
ENSG00000196233	LCOR	4.541477892	1.99730917	1.189576755	0.004496
ENSG00000196547	MAN2A2	7.682807833	3.37893992	1.18949018	0.004654
ENSG00000138663	COPS4	22.48633763	9.891570417	1.189209878	0.004649
ENSG00000197601	FAR1	24.1042055	10.60446177	1.189115304	0.004414
ENSG00000184983	NDUFA6	74.0098238	32.56074549	1.189044916	0.00455
ENSG00000149679	CABLES2	6.98816125	3.074877598	1.188564382	0.005684
ENSG00000168268	NT5DC2	27.61788958	12.16069556	1.187857931	0.004526
ENSG00000177485	ZBTB33	10.97919994	4.836068512	1.187252854	0.004888
ENSG00000245958	AC093752.1	2.277707913	1.003297516	1.186608093	0.007499
ENSG00000197943	PLCG2	5.537755773	2.442352808	1.185453237	0.004856
ENSG00000148468	FAM171A1	6.159524094	2.716346213	1.185301337	0.005937
ENSG00000168301	KCTD6	4.930356315	2.173958661	1.185268657	0.007092
ENSG00000138764	CCNG2	7.617692937	3.361278466	1.184726872	0.005036
ENSG00000167173	C15orf39	3.681030762	1.625089252	1.183685133	0.00615
ENSG00000167771	RCOR2	3.296441696	1.455046696	1.183304672	0.009384
ENSG00000142065	ZFP14	2.710147567	1.196813674	1.183262808	0.006225
ENSG00000229944	EIF4EP2	25.87631125	11.43033848	1.182736559	0.006962
ENSG00000164073	MFSD8	3.226977028	1.426046948	1.182382367	0.005836
ENSG00000156671	SAMD8	5.550163302	2.455026967	1.181106371	0.005455
ENSG00000102081	FMR1	6.952529311	3.076554968	1.180620084	0.005151
ENSG00000129534	MIS18BP1	8.423946873	3.731453847	1.179128506	0.005345

ENSG00000170348	TMED10	105.8329531	46.88698541	1.179067715	0.004623
ENSG00000153443	UBALD1	17.78104288	7.879002125	1.178659199	0.005141
ENSG00000174197	MGA	6.294686622	2.789611589	1.178538716	0.00489
ENSG00000117620	SLC35A3	2.797457801	1.241920151	1.175908759	0.005406
ENSG00000233426	EIF3FP3	21.97006665	9.76874761	1.173441064	0.006428
ENSG00000247556	OIP5-AS1	22.75729355	10.1267159	1.172685689	0.0049
ENSG00000152061	RABGAP1L	4.22248597	1.880101258	1.171695508	0.005403
ENSG00000114423	CBLB	1.835712606	0.817258075	1.171600591	0.006744
ENSG00000106144	CASP2	12.88992583	5.75446119	1.167954258	0.005303
ENSG00000116209	TMEM59	19.54311332	8.738325043	1.16573444	0.005233
ENSG00000188486	H2AFX	116.424518	52.05723663	1.1657297	0.005223
ENSG00000253797	UTP14C	2.879409792	1.287273719	1.165403436	0.007623
ENSG00000253276	CCDC71L	8.491372319	3.797249713	1.165330587	0.006207
ENSG00000130119	GNL3L	17.72129198	7.935988019	1.163348644	0.005942
ENSG00000197961	ZNF121	12.59729628	5.642462208	1.163168686	0.005537
ENSG00000095066	HOOK2	7.254516073	3.250028351	1.162760615	0.006151
ENSG00000169914	OTUD3	8.533691879	3.830035114	1.160202892	0.005887
ENSG00000090612	ZNF268	3.480391571	1.562501313	1.159753337	0.006175
ENSG00000092330	TINF2	15.91606587	7.146914384	1.159425834	0.00629
ENSG00000102225	CDK16	47.22960054	21.22886256	1.158190076	0.005433
ENSG00000245910	SNHG6	72.91510894	32.77364102	1.158133329	0.005741
ENSG00000078140	UBE2K	36.97355138	16.62508324	1.157639588	0.005529
ENSG00000090615	GOLGA3	17.98727196	8.100559278	1.155389556	0.005624
ENSG00000169738	DCXR	62.65620692	28.21818256	1.155309137	0.005752
ENSG00000166710	B2M	35.02856672	15.78012344	1.154935559	0.005619
ENSG00000187678	SPRY4	24.67664777	11.12089138	1.154372028	0.005701
ENSG00000060762	MPC1	16.51417232	7.441217558	1.154277291	0.007357
ENSG00000203499	IQANK1	2.331665056	1.051241523	1.153003182	0.00983
ENSG00000188153	COL4A5	1.739739796	0.784722239	1.152713172	0.007775
ENSG00000006607	FARP2	5.293848129	2.39142468	1.150878885	0.006185
ENSG00000167460	TPM4	96.94108398	43.84006407	1.149403907	0.005704
ENSG00000112972	HMGCS1	37.56424933	17.00275113	1.148095409	0.005941
ENSG00000112200	ZNF451	5.327543155	2.415943351	1.145332819	0.006305
ENSG00000177600	RPLP2	314.6839854	142.8756425	1.143685403	0.00596
ENSG00000214113	LYRM4	5.345422484	2.427059033	1.143465268	0.006791
ENSG00000167182	SP2	6.252902434	2.839176599	1.143197208	0.008016
ENSG00000100154	TTC28	4.596564809	2.088591693	1.142420833	0.006767
ENSG00000135119	RNFT2	3.310886649	1.504294834	1.142249659	0.008011
ENSG00000027697	IFNGR1	13.46519255	6.119584571	1.142073264	0.007005
ENSG00000182287	AP1S2	12.64999868	5.750615184	1.141767748	0.006618
ENSG00000101997	CCDC22	12.83789882	5.835573928	1.14172884	0.007444
ENSG00000090975	PITPNM2	1.806248062	0.820893406	1.141718612	0.009219
ENSG00000185049	NELFA	11.96887385	5.445488468	1.140578977	0.006663

ENSG00000066629	EML1	1.861288126	0.846666772	1.140428375	0.009246
ENSG00000165355	FBXO33	9.445919442	4.298560894	1.140136924	0.007259
ENSG00000044446	PHKA2	3.942984167	1.794689351	1.139847563	0.007319
ENSG00000109323	MANBA	3.625936671	1.650551972	1.139742109	0.007253
ENSG00000120675	DNAJC15	3.481254913	1.586357841	1.138112254	0.007906
ENSG00000141873	SLC39A3	9.81297163	4.474345451	1.137361313	0.007157
ENSG00000115825	PRKD3	7.281045451	3.320965706	1.136974584	0.006875
ENSG00000000003	TSPAN6	15.0188059	6.850188895	1.13697433	0.006866
ENSG00000064490	RFXANK	31.91920969	14.57528741	1.135340947	0.006833
ENSG00000155959	VBP1	52.94618315	24.1782968	1.135283828	0.006672
ENSG00000081760	AACS	6.890678906	3.14849348	1.134457489	0.006695
ENSG00000079337	RAPGEF3	18.83770321	8.609433045	1.13414899	0.006502
ENSG00000111077	TNS2	3.800085476	1.736762671	1.133885859	0.008064
ENSG00000072786	STK10	7.406647112	3.385694681	1.133787476	0.007036
ENSG00000188283	ZNF383	2.219091896	1.0157724	1.131452895	0.009379
ENSG00000159259	CHAF1B	10.76885689	4.932864554	1.130756453	0.007329
ENSG00000213722	DDAH2	14.20837037	6.508113477	1.130729924	0.007782
ENSG00000088808	PPP1R13B	8.393698181	3.846976689	1.130023478	0.007127
ENSG00000168256	NKIRAS2	16.91387362	7.752721806	1.12989098	0.006988
ENSG00000166123	GPT2	23.68762884	10.8631939	1.129180987	0.006838
ENSG00000204271	SPIN3	2.131345509	0.977718591	1.128556714	0.008138
ENSG00000112039	FANCE	7.466122517	3.425409779	1.128180266	0.009124
ENSG00000138621	PPCDC	6.476137039	2.971579903	1.128083358	0.008893
ENSG00000250317	SMIM20	27.99521515	12.85690166	1.126989971	0.007837
ENSG00000145354	CISD2	9.005711916	4.13664241	1.126775952	0.007549
ENSG00000013375	PGM3	12.24225408	5.626666455	1.125971305	0.007206
ENSG00000186184	POLR1D	17.4261358	8.010242877	1.125844624	0.006929
ENSG00000198668	CALM1	67.63765631	31.10470538	1.125233115	0.00681
ENSG00000124333	VAMP7	26.16486793	12.03418014	1.124918336	0.007456
ENSG00000120686	UFM1	33.07935388	15.22068729	1.124391116	0.007084
ENSG00000177732	SOX12	9.052010175	4.166934247	1.123589865	0.008088
ENSG00000137337	MDC1	6.534158409	3.008632453	1.12327642	0.007777
ENSG00000132912	DCTN4	27.71371021	12.7655467	1.122849927	0.0071
ENSG00000144746	ARL6IP5	70.84638677	32.64051597	1.12253606	0.007114
ENSG00000103978	TMEM87A	13.63113946	6.286422915	1.121033488	0.007628
ENSG00000100129	EIF3L	83.48336216	38.52170901	1.120348447	0.00709
ENSG00000087269	NOP14	29.986789	13.84139498	1.119808561	0.007437
ENSG00000130021	PUDP	8.072189351	3.730260163	1.117884482	0.00928
ENSG00000146574	CCZ1B	7.013741598	3.242817747	1.117315881	0.00819
ENSG00000153561	RMND5A	22.69212824	10.49999157	1.116301809	0.007475
ENSG00000204519	ZNF551	5.111153034	2.366960579	1.114793065	0.00931
ENSG00000174456	C12orf76	3.330037341	1.54246152	1.114474579	0.009532
ENSG00000111962	UST	4.89007905	2.266114655	1.113787759	0.00984

ENSG00000179889	PDXDC1	22.54460505	10.45107109	1.113645659	0.007527
ENSG00000188917	TRMT2B	5.214164756	2.417539756	1.113025329	0.009779
ENSG00000163001	CFAP36	21.72128139	10.08926287	1.110726829	0.008213
ENSG00000114315	HES1	56.47723146	26.24569081	1.110068298	0.007914
ENSG00000147027	TMEM47	18.51175865	8.612539499	1.108368399	0.008283
ENSG00000131652	THOC6	18.30921633	8.519432889	1.108033567	0.009226
ENSG00000102158	MAGT1	27.22671905	12.67766741	1.107222878	0.008017
ENSG00000123131	PRDX4	87.43342217	40.76641464	1.105306885	0.008044
ENSG00000102786	INTS6	3.362951523	1.568788703	1.104529497	0.008385
ENSG00000169692	AGPAT2	74.39111673	34.70511235	1.104472156	0.008167
ENSG00000146433	TMEM181	19.69885536	9.193464937	1.103903871	0.008302
ENSG00000148019	CEP78	5.32688087	2.488399256	1.102513191	0.008643
ENSG00000204392	LSM2	76.54846272	35.7855599	1.101444622	0.008657
ENSG00000137812	KNL1	9.686071317	4.528717802	1.101276116	0.008489
ENSG00000135837	CEP350	10.79333299	5.048801883	1.100626689	0.008333
ENSG00000162378	ZYG11B	10.21444783	4.783178194	1.09902055	0.008723
ENSG00000100075	SLC25A1	81.05898988	37.97177044	1.098549574	0.008422
ENSG00000175115	PACS1	4.142941671	1.941700101	1.097655035	0.009699
ENSG00000242125	SNHG3	24.51113281	11.5083271	1.095227684	0.008843
ENSG00000110344	UBE4A	10.34469493	4.860229483	1.094221832	0.009193
ENSG00000143772	ITPKB	7.399754355	3.480020994	1.09276895	0.009508
ENSG00000111554	MDM1	7.407681834	3.485905054	1.091864255	0.009668
ENSG00000071994	PDCD2	18.56987759	8.743877282	1.09110302	0.009001
ENSG00000165480	SKA3	21.0846533	9.927779018	1.091070429	0.009535
ENSG00000121741	ZMYM2	13.41196984	6.324294805	1.089053509	0.008967
ENSG00000053900	ANAPC4	6.507021755	3.070222136	1.088031567	0.009957
ENSG00000122026	RPL21	382.9249178	180.7641946	1.087499365	0.008819
ENSG00000102317	RBM3	60.65666076	28.65031767	1.086633224	0.009056
ENSG00000086758	HUWE1	19.14485296	9.055646537	1.084597741	0.009103
ENSG00000215193	PEX26	3.933606664	1.862431714	1.083111687	0.009817
ENSG00000174032	SLC25A30	10.99066831	5.205014182	1.082745506	0.009955
ENSG00000187109	NAP1L1	56.80943305	26.91203771	1.08242698	0.009124
ENSG00000138160	KIF11	34.97596989	16.56972354	1.082320298	0.009413
ENSG00000144357	UBR3	10.08614329	4.790806156	1.07850595	0.0099
ENSG00000090889	KIF4A	28.5944117	13.58792319	1.077898477	0.009811
ENSG00000253719	ATXN7L3B	27.79468831	13.22172172	1.076415247	0.00973
ENSG00000164024	METAP1	38.76090271	18.46193371	1.074551485	0.009948
ENSG00000130513	GDF15	38.54192272	81.54738366	-1.076602238	0.009781
ENSG00000187735	TCEA1	46.98142187	99.48261669	-1.077777404	0.009493
ENSG00000163162	RNF149	8.752824983	18.54022607	-1.078184498	0.009989
ENSG00000151576	QTRT2	5.758858994	12.19953473	-1.078325964	0.009922
ENSG00000241837	ATP5PO	13.10179301	27.8025146	-1.080800303	0.009792
ENSG00000160410	SHKBP1	12.37851514	26.29378505	-1.082242347	0.009632

ENSG00000143164	DCAF6	8.912374131	18.94588844	-1.083373037	0.009484
ENSG00000184220	CMSS1	7.134242702	15.17972721	-1.084676771	0.009444
ENSG00000087157	PGS1	5.411489393	11.52210873	-1.085597055	0.009876
ENSG00000105738	SIPA1L3	5.057919584	10.7717353	-1.085999	0.00935
ENSG00000101333	PLCB4	3.79096988	8.07682418	-1.086506794	0.009982
ENSG00000125124	BBS2	4.440675228	9.492767138	-1.091381144	0.009226
ENSG00000108262	GIT1	13.53299869	28.97885872	-1.093908052	0.008703
ENSG00000105429	MEGF8	2.193625932	4.703827888	-1.095794807	0.009388
ENSG00000166965	RCCD1	3.540284719	7.603154545	-1.09794009	0.009614
ENSG00000175792	RUVBL1	14.77109593	31.7322248	-1.098573936	0.008347
ENSG00000072134	EPN2	3.836588293	8.246253183	-1.09923087	0.008927
ENSG00000146859	TMEM140	8.328185387	17.92073106	-1.100782845	0.009334
ENSG00000106211	HSPB1	54.00910159	116.3457644	-1.102540186	0.008155
ENSG00000140688	C16orf58	6.561351772	14.15649146	-1.104667042	0.008785
ENSG00000126456	IRF3	10.84835767	23.40802161	-1.104868255	0.008335
ENSG00000033170	FUT8	2.145402248	4.629976645	-1.104910138	0.009639
ENSG00000172062	SMN1	4.411960736	9.524991079	-1.105436025	0.009531
ENSG00000143374	TARS2	8.773202318	18.93951076	-1.105550225	0.008423
ENSG00000168827	GFM1	10.83960486	23.41242119	-1.10636247	0.007915
ENSG00000155366	RHOC	22.79112098	49.23054377	-1.106479456	0.007924
ENSG00000068001	HYAL2	5.672524338	12.26184529	-1.107394623	0.008633
ENSG00000270231	NBPF8	2.59493137	5.61008733	-1.107468396	0.009455
ENSG00000089876	DHX32	5.66241544	12.24523093	-1.107986933	0.008776
ENSG00000163029	SMC6	4.92118959	10.64399968	-1.10831813	0.008053
ENSG00000180921	FAM83H	16.56193759	35.83266537	-1.108802392	0.007792
ENSG00000177000	MTHFR	1.538435781	3.329766377	-1.109173259	0.008932
ENSG00000120306	CYSTM1	14.90952819	32.28382056	-1.109901637	0.008226
ENSG00000159314	ARHGAP27	6.196077774	13.43408868	-1.11184964	0.007738
ENSG00000163946	FAM208A	5.682549085	12.32079416	-1.11185327	0.007793
ENSG00000134815	DHX34	4.863720831	10.54636757	-1.111905986	0.008188
ENSG00000269825	AC022150.4	3.359855416	7.292868185	-1.113092968	0.009875
ENSG00000172292	CERS6	7.680767154	16.68494216	-1.114592127	0.007647
ENSG00000145730	PAM	4.085961441	8.885828868	-1.116157851	0.007849
ENSG00000142546	NOSIP	20.49926539	44.59452263	-1.116689222	0.007392
ENSG00000173818	ENDOV	1.382670368	3.017946593	-1.121133866	0.009342
ENSG00000179456	ZBTB18	2.20373857	4.813238921	-1.122009364	0.009613
ENSG00000181038	METTL23	8.033984263	17.58718271	-1.125505801	0.008212
ENSG00000153113	CAST	3.282950742	7.188422301	-1.126021948	0.007196
ENSG00000181523	SGSH	2.807275181	6.154679215	-1.127732273	0.007723
ENSG00000139190	VAMP1	1.949209574	4.27625355	-1.128296275	0.009861
ENSG00000166128	RAB8B	3.712476587	8.14344488	-1.128481627	0.007776
ENSG00000153767	GTF2E1	8.605075023	18.87489505	-1.128506398	0.007296
ENSG00000180917	CMTR2	5.858340036	12.85862301	-1.12952026	0.006981

ENSG00000267321	LINC02001	5.417661501	11.90508563	-1.130760036	0.009598
ENSG00000103018	CYB5B	22.67540179	49.81461725	-1.130857079	0.006586
ENSG00000082996	RNF13	10.03540537	22.05522735	-1.131348854	0.007043
ENSG00000171843	MLLT3	3.161280873	6.94932463	-1.131642001	0.00727
ENSG00000128805	ARHGAP22	1.330629126	2.930093786	-1.13381213	0.008845
ENSG00000137038	DMAC1	22.52235796	49.58261809	-1.13385695	0.006615
ENSG00000070214	SLC44A1	3.347092876	7.369807461	-1.134057427	0.006802
ENSG00000006282	SPATA20	11.38393206	25.08647139	-1.135283891	0.006581
ENSG00000158417	EIF5B	10.22505829	22.5661102	-1.137439096	0.006402
ENSG00000106351	AGFG2	1.334876576	2.949888103	-1.138816082	0.009082
ENSG00000104946	TBC1D17	3.240684063	7.160212765	-1.138870886	0.007513
ENSG00000110400	NECTIN1	1.540977537	3.406205619	-1.13940469	0.008043
ENSG00000070778	PTPN21	1.768367508	3.909812978	-1.139799562	0.007811
ENSG00000198517	MAFK	3.158747623	6.998630778	-1.142860728	0.007436
ENSG00000154079	SDHAF4	5.89058785	13.05591057	-1.14316062	0.00834
ENSG00000158079	PTPDC1	1.396328305	3.096512028	-1.143972501	0.008252
ENSG00000131188	PRR7	6.744152888	14.95472246	-1.143978552	0.007697
ENSG00000143924	EML4	6.410069148	14.21578625	-1.144445578	0.006208
ENSG00000105321	CCDC9	4.053569085	8.999192009	-1.145772172	0.007126
ENSG00000196776	CD47	3.485084404	7.738985949	-1.146245179	0.006524
ENSG00000151693	ASAP2	3.621751209	8.043421667	-1.146402642	0.006606
ENSG00000167716	WDR81	2.730074313	6.069863499	-1.147969243	0.006595
ENSG00000172037	LAMB2	11.40654548	25.36468236	-1.148347683	0.005929
ENSG00000242372	EIF6	38.4805316	85.57413505	-1.148421083	0.005984
ENSG00000196369	SRGAP2B	0.826461889	1.838817043	-1.14848605	0.009167
ENSG00000136444	RSAD1	14.91057771	33.18314329	-1.149489181	0.00594
ENSG00000105520	PLPPR2	2.425675418	5.402209953	-1.150067794	0.008411
ENSG00000206195	DUXAP8	3.187600662	7.103976073	-1.151356823	0.006676
ENSG00000108852	MPP2	1.606081277	3.580171187	-1.151491012	0.007598
ENSG00000127399	LRRC61	2.123648137	4.746363937	-1.154983005	0.008745
ENSG00000135749	PCNX2	1.076773107	2.410695819	-1.157889725	0.00651
ENSG00000131759	RARA	1.66046602	3.718433086	-1.158003035	0.007964
ENSG00000133739	LRRCC1	1.814965285	4.065357572	-1.158416112	0.007568
ENSG00000115170	ACVR1	2.167816618	4.857752333	-1.158992532	0.00756
ENSG00000130054	FAM155B	2.040854311	4.573356592	-1.158998654	0.007813
ENSG00000080503	SMARCA2	2.784198628	6.239634962	-1.15955953	0.005583
ENSG00000154743	TSEN2	4.829622419	10.82940537	-1.16025767	0.00591
ENSG00000079819	EPB41L2	7.907653617	17.73608582	-1.160756433	0.005415
ENSG00000150093	ITGB1	84.85692853	190.3717268	-1.161149502	0.005227
ENSG00000182700	IGIP	1.450044561	3.26208986	-1.164283018	0.0093
ENSG00000108774	RAB5C	14.98283095	33.68988317	-1.164344062	0.005476
ENSG00000114631	PODXL2	35.36280142	79.53337864	-1.164715191	0.005274
ENSG00000161010	MRNIP	1.839425445	4.141221029	-1.165968034	0.006224

ENSG00000186866	POFUT2	3.278967477	7.39179496	-1.167901539	0.00588
ENSG00000044574	HSPA5	175.5245547	395.7206595	-1.168246292	0.004957
ENSG00000106976	DNM1	1.799392033	4.058800014	-1.16876394	0.00587
ENSG00000143919	CAMKMT	1.968344962	4.441542545	-1.168970791	0.007409
ENSG00000103356	EARS2	9.852032107	22.22531972	-1.169077455	0.005187
ENSG00000168237	GLYCTK	1.678074856	3.788448755	-1.169388565	0.009007
ENSG00000196455	PIK3R4	4.640236998	10.47470376	-1.169908802	0.005605
ENSG00000065600	TMEM206	5.291294211	11.95116983	-1.170621866	0.005992
ENSG00000153250	RBMS1	2.532343935	5.726179222	-1.172397217	0.005373
ENSG00000106100	NOD1	1.256014282	2.841998799	-1.172990617	0.006868
ENSG00000196116	TDRD7	2.843732065	6.434698782	-1.173177594	0.006081
ENSG00000103260	METRN	5.172251082	11.72425215	-1.175847607	0.005549
ENSG00000023330	ALAS1	15.58715947	35.4080531	-1.179052618	0.004941
ENSG00000107372	ZFAND5	15.24964558	34.68459684	-1.180922029	0.004633
ENSG00000143653	SCCPDH	17.31183078	39.3801017	-1.181051212	0.004836
ENSG00000165060	FXN	0.839536956	1.910564231	-1.181236699	0.006715
ENSG00000122707	RECK	0.711977077	1.621691267	-1.182028582	0.009354
ENSG00000237036	ZEB1-AS1	1.63607873	3.727339051	-1.182652434	0.007404
ENSG00000117153	KLHL12	12.39917373	28.24159806	-1.182921937	0.004765
ENSG00000183020	AP2A2	5.185245809	11.81081336	-1.182979964	0.004722
ENSG00000109089	CDR2L	14.39329158	32.80012698	-1.183662316	0.004694
ENSG00000105254	TBCB	19.82484705	45.27454669	-1.18676735	0.004523
ENSG00000123213	NLN	5.102954664	11.65689265	-1.187143478	0.00455
ENSG00000278053	DDX52	8.299310144	18.95844355	-1.187165177	0.004474
ENSG00000133265	HSPBP1	18.98634886	43.39472638	-1.18789378	0.00462
ENSG00000112679	DUSP22	1.749021681	3.999237769	-1.188180875	0.005851
ENSG00000008277	ADAM22	1.140630798	2.608949891	-1.188766483	0.005341
ENSG00000234171	RNASEH1-AS1	7.58176077	17.34662854	-1.189020456	0.006048
ENSG00000134686	PHC2	14.3876336	32.9402592	-1.190404567	0.004387
ENSG00000183853	KIRREL1	18.83317826	43.159565	-1.191817391	0.004242
ENSG00000243335	KCTD7	2.214932684	5.076901341	-1.191867305	0.005112
ENSG00000100364	KIAA0930	4.053843511	9.29307253	-1.192200583	0.00448
ENSG00000162302	RPS6KA4	14.21385835	32.59347887	-1.19264729	0.004379
ENSG00000134775	FHOD3	0.66101278	1.517393965	-1.193457077	0.007465
ENSG00000090006	LTBP4	2.62318629	6.039819367	-1.198421749	0.004615
ENSG00000159840	ZYX	9.3003229	21.41739264	-1.198737593	0.004409
ENSG00000138036	DYNC2LI1	3.112645098	7.172955879	-1.199564073	0.004961
ENSG00000186792	HYAL3	6.669204949	15.3694069	-1.19959334	0.004965
ENSG00000157557	ETS2	5.901797071	13.59941638	-1.199599562	0.00447
ENSG00000197774	EME2	0.870908222	2.008477031	-1.20022209	0.006547
ENSG00000058453	CROCC	1.234109807	2.845947558	-1.20047987	0.005161
ENSG00000177380	PPFIA3	2.07923263	4.794809685	-1.200537712	0.004912
ENSG00000105948	TTC26	3.109459428	7.172486635	-1.200969781	0.004817

ENSG00000172123	SLFN12	1.831047842	4.224833983	-1.200993779	0.006413
ENSG00000104856	RELB	2.684732421	6.194567125	-1.201128606	0.005891
ENSG00000084731	KIF3C	0.672169519	1.551993296	-1.201646632	0.008254
ENSG00000169604	ANTXR1	3.389803214	7.826026576	-1.202362956	0.004306
ENSG00000160094	ZNF362	3.123559663	7.216390421	-1.203106314	0.00527
ENSG00000157350	ST3GAL2	2.294254532	5.310567425	-1.206086402	0.004409
ENSG00000012061	ERCC1	5.732760013	13.26998576	-1.206170162	0.004151
ENSG00000135211	TMEM60	8.536812521	19.77803629	-1.207067782	0.005369
ENSG00000116337	AMPD2	3.245828653	7.518472485	-1.207096613	0.004356
ENSG00000005020	SKAP2	7.6682358	17.76739134	-1.20760409	0.003987
ENSG00000137124	ALDH1B1	18.76703129	43.48620248	-1.207725813	0.003897
ENSG00000099364	FBXL19	6.893636577	15.98682927	-1.208842165	0.004114
ENSG00000164967	RPP25L	15.99479709	37.09764228	-1.208915072	0.00441
ENSG00000198431	TXNRD1	54.97888915	127.6029246	-1.210142822	0.003654
ENSG00000104852	SNRNP70	29.51922642	68.53039018	-1.210500092	0.003699
ENSG00000091317	CMTM6	18.37878086	42.68371113	-1.21103311	0.003746
ENSG00000184110	EIF3C	4.805745769	11.16834123	-1.211893951	0.003972
ENSG00000146540	C7orf50	13.97340512	32.47379076	-1.211971443	0.003746
ENSG00000170832	USP32	4.978692005	11.59050276	-1.214432839	0.003839
ENSG00000221944	TIGD1	1.274432353	2.969417927	-1.214444146	0.008123
ENSG00000144560	VGLL4	6.912269839	16.10259901	-1.2154166	0.003718
ENSG00000109762	SNX25	2.818024706	6.566956802	-1.215639473	0.004557
ENSG00000158106	RHPN1	3.936165424	9.173650354	-1.215878262	0.00428
ENSG00000263528	IKBKE	1.058138558	2.467816536	-1.21604508	0.007553
ENSG00000169641	LUZP1	4.036635489	9.40869099	-1.216170071	0.003766
ENSG00000176531	PHLDB3	1.128840422	2.634859804	-1.21746489	0.006363
ENSG00000136231	IGF2BP3	4.521724844	10.55570271	-1.218405269	0.003724
ENSG00000198898	CAPZA2	18.17729104	42.45722345	-1.219286894	0.003455
ENSG00000154917	RAB6B	2.191909573	5.121109873	-1.219373991	0.004416
ENSG00000171603	CLSTN1	24.73451634	57.8699027	-1.221698973	0.0034
ENSG00000144366	GULP1	0.90345035	2.115028552	-1.222192151	0.004419
ENSG00000172456	FGGY	0.384423401	0.900851395	-1.223085729	0.006321
ENSG00000165752	STK32C	3.774017568	8.843969905	-1.223703258	0.004312
ENSG00000105855	ITGB8	2.249731316	5.274936383	-1.224713471	0.003588
ENSG00000197694	SPTAN1	14.22702462	33.38210647	-1.225864802	0.003265
ENSG00000157388	CACNA1D	0.178604996	0.41962709	-1.226566878	0.007444
ENSG00000022567	SLC45A4	1.072790964	2.522085752	-1.228208441	0.004625
ENSG00000117262	GPR89A	2.315983234	5.453416154	-1.23063152	0.004102
ENSG00000227473	TSSK5P	3.915946322	9.229000889	-1.23099937	0.007262
ENSG00000167535	CACNB3	2.412608858	5.682219312	-1.231044709	0.003847
ENSG00000106049	HIBADH	28.18674186	66.40280597	-1.231602309	0.003243
ENSG00000159685	CHCHD6	7.501116025	17.67519582	-1.231776498	0.003672
ENSG00000104365	IKBKB	2.401334507	5.662588687	-1.232902782	0.00349

ENSG00000148225	WDR31	1.211295248	2.858378962	-1.233341403	0.005236
ENSG00000089057	SLC23A2	2.279711884	5.390635446	-1.236785413	0.003679
ENSG00000227671	AL390728.4	4.720208326	11.17005682	-1.237717568	0.004114
ENSG00000117305	HMGCL	5.021934697	11.90676892	-1.240751872	0.00327
ENSG00000068885	IFT80	1.851954473	4.3920889	-1.24096383	0.003807
ENSG00000090013	BLVRB	35.45207279	84.0666802	-1.241032207	0.003035
ENSG00000151746	BICD1	1.305389432	3.095988386	-1.241095575	0.003534
ENSG00000125826	RBCK1	23.0318414	54.6190181	-1.24116824	0.002972
ENSG00000187098	MITF	2.461390568	5.840836273	-1.241890291	0.003471
ENSG00000073008	PVR	19.31938857	45.84435679	-1.242071712	0.002991
ENSG00000062716	VMP1	14.51857116	34.45258794	-1.242111207	0.002942
ENSG00000078142	PIK3C3	1.847599876	4.385596039	-1.242403638	0.003209
ENSG00000104980	TIMM44	9.506019076	22.5738739	-1.243087158	0.003036
ENSG00000274265	AC245297.3	1.695277491	4.035743528	-1.244763784	0.009542
ENSG00000226137	BAIAP2-DT	2.214900281	5.270663009	-1.245760088	0.003868
ENSG00000105865	DUS4L	1.551877377	3.695282545	-1.246579818	0.004042
ENSG00000161647	MPP3	1.702680358	4.056689076	-1.247349218	0.004325
ENSG00000196924	FLNA	58.47199779	139.2794919	-1.247600848	0.002743
ENSG00000146232	NFKBIE	2.07227342	4.94284764	-1.248728601	0.005177
ENSG00000253982	AC100810.1	3.729714969	8.905220178	-1.250114416	0.0051
ENSG00000119401	TRIM32	6.610502527	15.78551364	-1.251031829	0.003106
ENSG00000176912	TYMSOS	2.954094367	7.063033588	-1.251450396	0.007889
ENSG00000198712	MT-CO2	5670.664425	13550.33365	-1.252180593	0.002636
ENSG00000197860	SGTB	1.214017098	2.904774616	-1.253433209	0.004161
ENSG00000184428	TOP1MT	5.300219418	12.68323204	-1.254109121	0.002867
ENSG00000104983	CCDC61	1.501563725	3.597654315	-1.254870865	0.005878
ENSG00000225138	SLC9A3-AS1	0.942163813	2.257925957	-1.255276065	0.005843
ENSG00000164741	DLC1	0.578085414	1.385508543	-1.255892566	0.004042
ENSG00000170502	NUDT9	11.3518891	27.21777767	-1.256934725	0.002822
ENSG00000173531	MST1	0.682177894	1.637675194	-1.257567517	0.006131
ENSG00000114841	DNAH1	0.626540784	1.503720793	-1.257978507	0.003765
ENSG00000147065	MSN	24.682178	59.26184544	-1.259046174	0.002557
ENSG00000015532	XYLT2	4.742098853	11.38710997	-1.259072463	0.002893
ENSG00000104635	SLC39A14	12.67046584	30.4611046	-1.260890203	0.002561
ENSG00000100300	TSPO	71.79630714	172.7148912	-1.261810567	0.002528
ENSG00000152492	CCDC50	8.514205626	20.48927078	-1.262309947	0.002546
ENSG00000168734	PKIG	6.428530886	15.47699289	-1.262724408	0.002991
ENSG00000215252	GOLGA8B	1.841392745	4.435459282	-1.26340773	0.003132
ENSG00000143079	CTTNBP2NL	2.720230587	6.553649947	-1.263787255	0.002869
ENSG00000173992	CCS	3.580644932	8.63056151	-1.264165338	0.003548
ENSG00000141198	TOM1L1	9.098028041	21.92931501	-1.264609389	0.002522
ENSG00000221978	CCNL2	16.37257662	39.46996447	-1.264876392	0.002463
ENSG00000163703	CRELD1	5.109814254	12.32229932	-1.265136404	0.002872

ENSG00000167964	RAB26	1.32678883	3.202506314	-1.265329495	0.005865
ENSG00000213625	LEPROT	10.95501697	26.4202425	-1.265415835	0.002526
ENSG00000112981	NME5	1.100536538	2.657342524	-1.26549456	0.007425
ENSG00000095539	SEMA4G	1.11364232	2.692774898	-1.268472816	0.004102
ENSG00000177646	ACAD9	11.00887771	26.61786753	-1.269075075	0.002486
ENSG00000242110	AMACR	0.699591052	1.693189607	-1.269258013	0.005697
ENSG00000137841	PLCB2	0.301310053	0.730826481	-1.271665909	0.008154
ENSG00000048471	SNX29	1.483091322	3.593569802	-1.271981227	0.002823
ENSG00000197249	SERPINA1	1.389438693	3.369561812	-1.272832252	0.003848
ENSG00000132182	NUP210	16.76116468	40.66539789	-1.274089115	0.002281
ENSG00000198691	ABCA4	0.274324178	0.666461488	-1.274133318	0.007851
ENSG00000145882	PCYOX1L	2.382231992	5.782548627	-1.274518887	0.002881
ENSG00000081087	OSTM1	5.68909893	13.8114196	-1.274881882	0.002478
ENSG00000103047	TANGO6	2.217885455	5.393304942	-1.277126463	0.002766
ENSG00000186814	ZSCAN30	1.833891745	4.45953482	-1.277126463	0.002766
ENSG00000077150	NFKB2	5.964025086	14.50508836	-1.277472961	0.002489
ENSG00000120889	TNFRSF10B	21.83245246	53.09437382	-1.277487263	0.002234
ENSG00000215158	AC138409.2	0.324312584	0.79021795	-1.278164016	0.007961
ENSG00000179406	LINC00174	0.334901886	0.81609708	-1.279242369	0.00501
ENSG00000271122	AC018647.2	3.133908807	7.641704392	-1.28102639	0.002802
ENSG00000160766	GBAP1	0.902913187	2.20387362	-1.28143828	0.005277
ENSG00000251474	RPL32P3	1.994642965	4.869877833	-1.282687979	0.003123
ENSG00000072954	TMEM38A	1.835500666	4.483760303	-1.283147583	0.003964
ENSG00000155903	RASA2	2.809867621	6.864226445	-1.283765729	0.002567
ENSG00000148450	MSRB2	5.579538746	13.63399183	-1.284032758	0.002813
ENSG00000198853	RUSC2	1.463446797	3.576638045	-1.284125364	0.003084
ENSG00000285796	AL162458.1	0.27406714	0.671079833	-1.285437912	0.007315
ENSG00000214293	APTR	1.981439117	4.851744832	-1.286650616	0.003531
ENSG00000169220	RGS14	3.587584131	8.794650772	-1.288719254	0.002563
ENSG00000147592	LACTB2	5.950802154	14.60422933	-1.290272928	0.002692
ENSG00000236859	NIFK-AS1	1.113841886	2.737376333	-1.291073559	0.006153
ENSG00000161970	RPL26	10.50871663	25.81049094	-1.291639848	0.002224
ENSG00000131650	KREMEN2	1.90224508	4.674954971	-1.291766822	0.003839
ENSG00000041353	RAB27B	0.376698762	0.927545853	-1.293805237	0.006075
ENSG00000075651	PLD1	0.458180928	1.127867759	-1.294269539	0.003323
ENSG00000109861	CTSC	7.599017706	18.71890995	-1.296001497	0.001952
ENSG00000113758	DBN1	18.43979943	45.44519336	-1.296698931	0.001935
ENSG00000110455	ACCS	0.494884329	1.221650599	-1.297511223	0.005873
ENSG00000120756	PLS1	13.08656542	32.28288622	-1.298059475	0.001941
ENSG00000223705	NSUN5P1	1.854435185	4.576569707	-1.298213528	0.002789
ENSG00000100003	SEC14L2	0.502682114	1.24155025	-1.298333199	0.005761
ENSG00000145358	DDIT4L	1.325857656	3.274256416	-1.29852142	0.004381
ENSG00000224699	LAMTOR5-AS1	0.202031003	0.49944906	-1.299079228	0.006933

ENSG00000259291	ZNF710-AS1	0.97912152	2.421712663	-1.300284973	0.005797
ENSG00000198585	NUDT16	6.693756943	16.54496953	-1.300849363	0.001945
ENSG00000198873	GRK5	0.268620755	0.665052098	-1.30088265	0.007973
ENSG00000171174	RBKS	1.072318402	2.659566242	-1.30469621	0.004216
ENSG00000133059	DSTYK	2.264902715	5.614848971	-1.304995928	0.002115
ENSG00000181619	GPR135	0.39459839	0.980345649	-1.305990295	0.007547
ENSG00000112837	TBX18	0.902909138	2.241063137	-1.306367511	0.002779
ENSG00000107614	TRDMT1	0.511658581	1.270448359	-1.306667537	0.003359
ENSG00000272645	GTF2IP20	0.91736454	2.278653494	-1.306838636	0.00416
ENSG00000164050	PLXNB1	5.551072417	13.78812695	-1.307945273	0.001822
ENSG00000118960	HS1BP3	2.749103505	6.833800383	-1.308983583	0.001988
ENSG00000122783	CYREN	4.162053919	10.34742342	-1.309137492	0.002018
ENSG00000121964	GTDC1	0.731732226	1.820615609	-1.310033657	0.002418
ENSG00000050820	BCAR1	5.381390586	13.38796794	-1.310235114	0.0018
ENSG00000143702	CEP170	4.369920072	10.88489847	-1.311994991	0.00178
ENSG00000161048	NAPEPLD	3.57008348	8.909998486	-1.31473804	0.001866
ENSG00000163472	TMEM79	1.226178862	3.064967594	-1.315907733	0.003916
ENSG00000187118	CMC1	1.594773683	3.986310491	-1.316868135	0.002005
ENSG00000141101	NOB1	31.82714371	79.5765363	-1.31745939	0.001668
ENSG00000198793	MTOR	4.527130691	11.32467464	-1.318158849	0.001679
ENSG00000116266	STXBP3	5.720247457	14.31356826	-1.318538719	0.001763
ENSG00000221990	EXOC3-AS1	1.33170709	3.337719256	-1.318924784	0.006064
ENSG00000168994	PXDC1	0.844491729	2.116930182	-1.319031199	0.006226
ENSG00000143862	ARL8A	9.224534952	23.09550444	-1.319251066	0.001933
ENSG00000227232	WASH7P	1.79389796	4.506132189	-1.322309029	0.005698
ENSG00000114735	HEMK1	0.860446862	2.15923013	-1.322522644	0.001917
ENSG00000180340	FZD2	5.737585568	14.41236881	-1.323849228	0.002002
ENSG00000169258	GPRIN1	1.835741722	4.613439784	-1.324318187	0.002447
ENSG00000270959	LPP-AS2	0.791169124	1.993308727	-1.32647784	0.005729
ENSG00000123384	LRP1	0.475222027	1.196461391	-1.327070722	0.002138
ENSG00000154957	ZNF18	2.413900542	6.078392059	-1.327122908	0.002422
ENSG00000113851	CRBN	2.444562811	6.158535619	-1.328216509	0.001782
ENSG00000100644	HIF1A	35.59591495	89.73480826	-1.329373548	0.001466
ENSG00000152056	AP1S3	4.477012581	11.29526957	-1.330309103	0.001745
ENSG00000168502	MTCL1	1.121757067	2.831050982	-1.33069055	0.001862
ENSG00000155254	MARVELD1	5.070149397	12.79703339	-1.330878641	0.001802
ENSG00000124275	MTRR	6.73917396	17.02211055	-1.332102194	0.001528
ENSG00000204406	MBD5	0.494410635	1.249366489	-1.332466904	0.001974
ENSG00000114988	LMAN2L	9.455327174	23.89349367	-1.332678407	0.001632
ENSG00000075151	EIF4G3	5.538880092	13.99897134	-1.333009377	0.001495
ENSG00000167216	KATNAL2	0.934778012	2.367775425	-1.335229181	0.002935
ENSG00000104723	TUSC3	10.14444469	25.69351451	-1.336068153	0.00146
ENSG00000162836	ACP6	2.536474686	6.426277626	-1.336446666	0.001539

ENSG00000130590	SAMD10	2.452957566	6.222676147	-1.337674484	0.002446
ENSG00000198938	MT-CO3	5553.361023	14083.36886	-1.338001377	0.001342
ENSG00000166503	HDGFL3	1.419157621	3.602421924	-1.339171357	0.001582
ENSG00000182263	FIGN	1.34913797	3.42634639	-1.339725241	0.001741
ENSG00000251136	AF117829.1	0.223421739	0.568439592	-1.339866423	0.00822
ENSG00000103152	MPG	16.20098151	41.15188876	-1.340145527	0.001524
ENSG00000175265	GOLGA8A	1.888083331	4.801259304	-1.341563824	0.001765
ENSG00000213699	SLC35F6	10.90148357	27.76185421	-1.343911636	0.001394
ENSG00000198468	FLVCR1-DT	0.958877506	2.445044477	-1.344296078	0.004269
ENSG00000102781	KATNAL1	0.824882433	2.104225721	-1.345766202	0.002215
ENSG00000105854	PON2	15.16802077	38.68288525	-1.346024639	0.001339
ENSG00000258056	AC009779.2	2.316587707	5.924987037	-1.348840319	0.003341
ENSG00000142627	EPHA2	20.01361335	51.16707906	-1.349620475	0.001276
ENSG00000234072	AC074117.1	0.404138299	1.036426329	-1.350712647	0.008986
ENSG00000221926	TRIM16	2.259674199	5.785871615	-1.351491289	0.001638
ENSG00000125247	TMTC4	2.764821758	7.079881535	-1.351708886	0.001489
ENSG00000197959	DNM3	0.224990069	0.576874906	-1.351984231	0.004297
ENSG00000259877	AC009113.1	1.077561767	2.766775037	-1.354079031	0.004183
ENSG00000110841	PPFIBP1	6.684242337	17.15090019	-1.354830768	0.001227
ENSG00000238072	AC009244.1	6.478364459	16.65604538	-1.354954215	0.0075
ENSG00000111647	UHRF1BP1L	3.252646182	8.349107089	-1.355230805	0.001398
ENSG00000137094	DNAJB5	0.907916771	2.332758181	-1.3559615	0.002363
ENSG00000119185	ITGB1BP1	4.486398352	11.53598875	-1.357796698	0.001317
ENSG00000276256	AC011043.1	5.368335815	13.80621119	-1.357810977	0.00162
ENSG00000214106	PAXIP1-AS2	0.315341016	0.811935351	-1.357903686	0.004553
ENSG00000104059	FAM189A1	2.343602378	6.030306962	-1.358581392	0.001542
ENSG00000196372	ASB13	10.56115788	27.17388323	-1.358746097	0.001279
ENSG00000006125	AP2B1	38.88850241	100.1580016	-1.360289067	0.001134
ENSG00000101187	SLCO4A1	12.31592896	31.73529654	-1.360910687	0.0012
ENSG00000177640	CASC2	0.274904281	0.709463356	-1.361519096	0.003908
ENSG00000106348	IMPDH1	16.21439801	41.80499102	-1.361762731	0.001178
ENSG00000135519	KCNH3	2.185552378	5.64180362	-1.363100111	0.00169
ENSG00000124496	TRERF1	1.145695235	2.95834911	-1.363475039	0.001683
ENSG00000184557	SOCS3	1.558930496	4.027698611	-1.36372614	0.002649
ENSG00000227372	TP73-AS1	1.554947709	4.023651412	-1.36676648	0.001374
ENSG00000107560	RAB11FIP2	2.492631849	6.450707639	-1.366920821	0.001385
ENSG00000130529	TRPM4	2.492953907	6.452830422	-1.367229519	0.001338
ENSG00000267221	C17orf113	0.423878038	1.099361244	-1.367388878	0.007242
ENSG00000038382	TRIO	1.960334576	5.074859084	-1.367571991	0.001183
ENSG00000102038	SMARCA1	8.073604169	20.92981178	-1.369582215	0.001171
ENSG00000017260	ATP2C1	11.14539856	28.89957095	-1.369992763	0.001076
ENSG00000167772	ANGPTL4	0.993288314	2.579019517	-1.370047056	0.003863
ENSG00000160691	SHC1	20.31531243	52.70130803	-1.370660178	0.001073

ENSG00000165512	ZNF22	4.373467406	11.35309287	-1.371251132	0.001455
ENSG00000171224	FAM241B	3.717015635	9.656092284	-1.371785719	0.0021
ENSG00000101871	MID1	1.658830697	4.310166128	-1.37271009	0.00131
ENSG00000182197	EXT1	4.316575093	11.21632101	-1.372950673	0.001133
ENSG00000144199	FAHD2B	2.825462486	7.34841634	-1.373754083	0.001688
ENSG00000239382	ALKBH6	0.292001752	0.761171186	-1.373879051	0.009559
ENSG00000165424	ZCCHC24	1.79517775	4.670395025	-1.374333803	0.001562
ENSG00000183741	CBX6	9.902579426	25.78757188	-1.376182566	0.001031
ENSG00000160172	FAM86C2P	2.21087041	5.763153268	-1.377050762	0.001646
ENSG00000023909	GCLM	7.26234218	18.92934973	-1.377439495	0.00107
ENSG00000142102	PGGHG	1.382256356	3.608282074	-1.379071247	0.001608
ENSG00000245694	CRNDE	3.245055533	8.468698749	-1.379087707	0.001181
ENSG00000090857	PDPR	5.576758281	14.56902766	-1.380766244	0.00101
ENSG00000188157	AGRN	15.30734908	39.99471354	-1.380998815	0.000967
ENSG00000160049	DFFA	11.97025069	31.28416316	-1.381361722	0.000988
ENSG00000102362	SYTL4	0.545663533	1.42822562	-1.38240029	0.002332
ENSG00000132676	DAP3	21.9036637	57.32957817	-1.383502772	0.000961
ENSG00000278619	MRM1	3.082717692	8.075199708	-1.383899888	0.001731
ENSG00000239213	NCK1-DT	1.432811118	3.758974552	-1.384590365	0.004126
ENSG00000104728	ARHGEF10	0.154875651	0.406484984	-1.384664526	0.006244
ENSG00000235609	AF127577.4	0.627239858	1.645869554	-1.385912524	0.002407
ENSG00000197548	ATG7	3.060702665	8.027259729	-1.386310008	0.001052
ENSG00000114779	ABHD14B	9.041837494	23.71978149	-1.386677742	0.001045
ENSG00000102908	NFAT5	5.49801858	14.42430947	-1.386908332	0.000938
ENSG00000118162	KPTN	1.514820955	3.983159158	-1.388714566	0.002551
ENSG00000075420	FNDC3B	5.094865059	13.39520402	-1.389972595	0.000928
ENSG00000176485	PLA2G16	5.980844364	15.7303012	-1.390310006	0.001082
ENSG00000158163	DZIP1L	0.414127361	1.091350644	-1.391108339	0.00391
ENSG00000142669	SH3BGRL3	34.3922803	90.52877745	-1.391656356	0.00092
ENSG00000223768	LINC00205	2.91220296	7.667131403	-1.391801995	0.001052
ENSG00000166886	NAB2	6.038761156	15.91247193	-1.393011406	0.001074
ENSG00000147082	CCNB3	0.61812797	1.630609287	-1.393435516	0.002447
ENSG00000167202	TBC1D2B	1.741952111	4.594146083	-1.394251337	0.001091
ENSG00000270441	AC135506.1	0.743265383	1.965695739	-1.394931085	0.007107
ENSG00000104756	KCTD9	9.123585758	24.07179522	-1.394993345	0.000925
ENSG00000178921	PFAS	6.857695674	18.10308919	-1.395774463	0.000911
ENSG00000171621	SPSB1	3.831049261	10.11543955	-1.395820539	0.00116
ENSG00000055483	USP36	3.855030216	10.1789449	-1.396081482	0.000933
ENSG00000101384	JAG1	7.114042649	18.80455521	-1.397710722	0.000872
ENSG00000166548	TK2	2.92014379	7.723808323	-1.398485294	0.001006
ENSG00000106701	FSD1L	0.778426945	2.062085703	-1.400285183	0.001355
ENSG00000198624	CCDC69	0.209220162	0.55584089	-1.400472487	0.009889
ENSG00000260261	AC124944.3	0.335197782	0.889549735	-1.400878095	0.004295

ENSG00000184922	FMNL1	0.584643318	1.553946816	-1.404658525	0.00171
ENSG00000251381	LINC00958	15.03985039	39.95927473	-1.405071034	0.000846
ENSG00000167397	VKORC1	1.258712802	3.35059425	-1.406154726	0.002881
ENSG00000144504	ANKMY1	0.648062232	1.723908679	-1.406270707	0.001284
ENSG00000258839	MC1R	0.292783431	0.780097526	-1.406292719	0.005571
ENSG00000250420	AACSP1	0.531590054	1.417057411	-1.407259343	0.004183
ENSG00000177570	SAMD12	0.782560139	2.08417189	-1.408240805	0.001074
ENSG00000142733	MAP3K6	2.726055463	7.26010643	-1.408259513	0.001002
ENSG00000076928	ARHGEF1	5.491719154	14.64286933	-1.410209247	0.000804
ENSG00000154814	OXNAD1	2.913669659	7.777546539	-1.411647379	0.000925
ENSG00000171004	HS6ST2	5.688902832	15.19547803	-1.412692023	0.000848
ENSG00000255284	AP006621.3	1.262638739	3.381538958	-1.413050876	0.006373
ENSG00000175793	SFN	28.71166957	76.77553645	-1.41432423	0.000808
ENSG00000107331	ABCA2	2.689162673	7.2039313	-1.416913252	0.000804
ENSG00000113070	HBEGF	1.121478301	3.010905818	-1.41880025	0.001934
ENSG00000216895	AC009403.1	1.090413043	2.93131227	-1.419915816	0.003123
ENSG00000239697	TNFSF12	0.911763428	2.453752827	-1.420858217	0.004867
ENSG00000113522	RAD50	0.436200591	1.172792879	-1.421183952	0.00175
ENSG00000279069	AC015813.5	0.965978056	2.601819506	-1.421570451	0.005571
ENSG00000250571	GLI4	1.763272211	4.744275929	-1.422470873	0.001401
ENSG00000145014	TMEM44	2.455273143	6.605038606	-1.422690854	0.000962
ENSG00000077348	EXOSC5	22.10023504	59.44350067	-1.422752055	0.000754
ENSG00000143387	CTSK	1.638645128	4.412263214	-1.422881106	0.002021
ENSG00000205220	PSMB10	0.518653733	1.40111229	-1.42410065	0.009486
ENSG00000206503	HLA-A	1.495641595	4.030332752	-1.424373276	0.001721
ENSG00000105552	BCAT2	15.38159795	41.44005251	-1.425193765	0.000691
ENSG00000186472	PCLO	0.279507453	0.755413154	-1.429057161	0.00118
ENSG00000173451	THAP2	0.793840339	2.146599843	-1.429631413	0.001371
ENSG00000176155	CCDC57	2.035848371	5.502863424	-1.429801582	0.000738
ENSG00000251209	LINC00923	0.189047706	0.512627298	-1.430945908	0.005716
ENSG00000104881	PPP1R13L	2.731997874	7.392320022	-1.431157778	0.000832
ENSG00000173821	RNF213	2.694042713	7.291829528	-1.431887543	0.00065
ENSG00000132589	FLOT2	29.34751533	79.4491742	-1.432186895	0.000641
ENSG00000174151	CYB561D1	1.637637174	4.444844272	-1.435433867	0.000946
ENSG00000073711	PPP2R3A	1.519567879	4.129607908	-1.437374595	0.000851
ENSG00000210077	MT-TV	21.1955185	57.73793992	-1.437850024	0.005033
ENSG00000107186	MPDZ	1.419660041	3.859642912	-1.438058154	0.000756
ENSG00000142192	APP	111.4036107	302.9164971	-1.438560248	0.000587
ENSG00000135503	ACVR1B	7.63006737	20.75309591	-1.438888803	0.000637
ENSG00000126368	NR1D1	1.552633993	4.230857848	-1.440533879	0.001454
ENSG00000158966	CACHD1	0.663678334	1.809108719	-1.441001654	0.001513
ENSG00000082212	ME2	3.107604785	8.468698749	-1.441682922	0.000614
ENSG00000112367	FIG4	3.071091036	8.374392527	-1.442230514	0.00082

ENSG00000160207	HSF2BP	1.116906703	3.049898247	-1.442389068	0.002739
ENSG00000111674	ENO2	6.237071487	17.01228672	-1.442895191	0.00066
ENSG00000108786	HSD17B1	0.509578668	1.392419488	-1.443954006	0.002045
ENSG00000172830	SSH3	8.951565941	24.44083671	-1.44437372	0.000639
ENSG00000242294	STAG3L5P	1.102754407	3.017200446	-1.445949845	0.001712
ENSG00000137449	CPEB2	1.542865327	4.222465315	-1.447471429	0.000788
ENSG00000174282	ZBTB4	5.866060668	16.06094386	-1.448398641	0.000606
ENSG00000113552	GNPDA1	23.98892073	65.70380992	-1.448999943	0.000557
ENSG00000253683	AC027309.2	12.40505569	34.03862399	-1.449617749	0.002282
ENSG00000128283	CDC42EP1	10.05254334	27.55724259	-1.450123291	0.000627
ENSG00000245573	BDNF-AS	0.314007681	0.864987864	-1.453106796	0.00643
ENSG00000049883	PTCD2	1.679695792	4.622258817	-1.455598151	0.000621
ENSG00000114021	NIT2	4.068488405	11.20527632	-1.456925244	0.000564
ENSG00000141441	GAREM1	0.999361336	2.756291601	-1.458438849	0.000861
ENSG00000235919	ASH1L-AS1	0.829487856	2.295544716	-1.459057376	0.007402
ENSG00000185875	THNSL1	4.213844295	11.62801267	-1.459528449	0.000628
ENSG00000111897	SERINC1	23.39026188	64.58332483	-1.460629242	0.00051
ENSG00000164171	ITGA2	3.042783772	8.420673259	-1.463780725	0.000564
ENSG00000265660	MIR4664	22.95257154	63.67711716	-1.464520234	0.003838
ENSG00000135686	KLHL36	4.749201807	13.15350567	-1.465028897	0.000506
ENSG00000116096	SPR	17.19414832	47.63643047	-1.465395655	0.000552
ENSG00000152518	ZFP36L2	11.58822701	32.11899336	-1.466093961	0.000506
ENSG00000165915	SLC39A13	3.956126882	10.97594875	-1.467354186	0.000586
ENSG00000133943	DGLUCY	2.613039142	7.255697389	-1.468564889	0.000565
ENSG00000085831	TTC39A	0.772151568	2.146832062	-1.470008615	0.000778
ENSG00000088854	C20orf194	1.364962037	3.799703012	-1.472042026	0.00064
ENSG00000271601	LIX1L	7.232299402	20.13495901	-1.472446146	0.000515
ENSG00000080947	CROCCP3	0.202395402	0.56504161	-1.473017212	0.004269
ENSG00000127415	IDUA	0.97151173	2.708168879	-1.473176636	0.001206
ENSG00000001617	SEMA3F	7.273058151	20.26676782	-1.473783183	0.000492
ENSG00000280187	AC022107.1	1.161457261	3.240769011	-1.474457482	0.001271
ENSG00000206573	THUMPD3-AS1	1.997710793	5.571314462	-1.474715664	0.000601
ENSG00000159131	GART	18.45330899	51.45073585	-1.474720408	0.00044
ENSG00000170293	CMTM8	4.235492879	11.81990444	-1.475057864	0.00097
ENSG00000148175	STOM	3.854505308	10.76386516	-1.476617226	0.000596
ENSG00000273702	AC091271.1	0.755158749	2.11873641	-1.478120839	0.007788
ENSG00000112305	SMAP1	0.522192607	1.46305806	-1.479480639	0.002094
ENSG00000260630	SNAI3-AS1	0.431376502	1.20861318	-1.479480639	0.002094
ENSG00000145911	N4BP3	1.896839541	5.308258377	-1.479651227	0.000599
ENSG00000168615	ADAM9	29.95641458	83.82158452	-1.479875796	0.000417
ENSG00000164889	SLC4A2	13.64771178	38.20494125	-1.48049502	0.000423
ENSG00000163702	IL17RC	3.626314449	10.16536977	-1.482226336	0.000524
ENSG00000138246	DNAJC13	7.699380371	21.58937581	-1.482882773	0.000421

ENSG00000167700	MFSD3	6.555479384	18.3914308	-1.48328535	0.000581
ENSG00000136717	BIN1	6.223493315	17.58482003	-1.493860732	0.000398
ENSG00000179588	ZFPM1	0.629175363	1.780268189	-1.494698054	0.001027
ENSG00000259456	ADNP-AS1	0.901519973	2.559272345	-1.494749617	0.007535
ENSG00000147437	GNRH1	0.474295695	1.34645032	-1.494749617	0.007535
ENSG00000173905	GOLIM4	15.69989176	44.40671234	-1.495397384	0.000378
ENSG00000177788	AL162595.1	1.447115178	4.108130673	-1.497740974	0.00301
ENSG00000175155	YPEL2	0.573724315	1.626904744	-1.498132549	0.000811
ENSG00000275964	AL355001.2	1.999807467	5.675257276	-1.498158405	0.001619
ENSG00000243701	DUBR	0.596083463	1.692186494	-1.49874099	0.001568
ENSG00000136059	VILL	0.187118294	0.532236008	-1.49941356	0.00465
ENSG00000126254	RBM42	18.12723317	51.43903634	-1.500022138	0.00038
ENSG00000007384	RHBDF1	2.984675054	8.480762423	-1.501719951	0.000451
ENSG00000059122	FLYWCH1	5.975731407	16.98223204	-1.502181868	0.000366
ENSG00000161513	FDXR	2.925794274	8.320791495	-1.502857272	0.000518
ENSG00000280385	AP000648.3	1.131090524	3.217939098	-1.502878032	0.000755
ENSG00000273230	AC102953.2	0.289984952	0.82839882	-1.504059541	0.006717
ENSG00000135740	SLC9A5	0.185061971	0.52866577	-1.504773323	0.005655
ENSG00000144730	IL17RD	0.909044426	2.589060396	-1.504869162	0.000543
ENSG00000162496	DHRS3	4.445553556	12.66519162	-1.505503498	0.000452
ENSG00000143067	ZNF697	0.613394834	1.74932256	-1.505721787	0.001102
ENSG00000121858	TNFSF10	0.397039987	1.134222497	-1.506138047	0.003455
ENSG00000069869	NEDD4	1.62157965	4.622545592	-1.506389821	0.000435
ENSG00000248508	SRP14-AS1	0.544663295	1.555937394	-1.506698714	0.002905
ENSG00000173621	LRFN4	6.309189347	17.99705434	-1.50739457	0.000413
ENSG00000255624	AC073585.1	1.076440183	3.08205102	-1.510869165	0.001508
ENSG00000149489	ROM1	0.519177945	1.489113877	-1.511700224	0.003562
ENSG00000284968	AC093827.4	0.417705327	1.19858413	-1.511903746	0.004484
ENSG00000171522	PTGER4	0.200435134	0.57634921	-1.512896917	0.007267
ENSG00000162849	KIF26B	0.710784936	2.03567624	-1.512972418	0.000472
ENSG00000089505	CMTM1	0.912719744	2.627732757	-1.51723565	0.003323
ENSG00000272758	AC083798.2	0.419155334	1.207721727	-1.518000243	0.004137
ENSG00000168517	HEXIM2	0.984151932	2.833387013	-1.518494081	0.001677
ENSG00000159842	ABR	4.300948939	12.3813657	-1.520791745	0.000309
ENSG00000256092	AC137767.1	0.669757893	1.933224769	-1.521373319	0.002856
ENSG00000251003	ZFPM2-AS1	2.162126811	6.243675054	-1.524507116	0.000568
ENSG00000112667	DNPH1	24.93755701	71.97910011	-1.524538116	0.000323
ENSG00000167925	GHDC	3.784499646	10.92625595	-1.524733897	0.00037
ENSG00000146729	NIPSNAP2	6.898925068	19.92200526	-1.525232175	0.000306
ENSG00000188002	AC026412.1	1.478141689	4.27139184	-1.525343673	0.000632
ENSG00000272636	DOC2B	1.328476645	3.839020851	-1.525815685	0.000456
ENSG00000275074	NUDT18	3.33141407	9.635797324	-1.526843992	0.000559
ENSG00000041982	TNC	2.03810314	5.895018863	-1.527453639	0.000345

ENSG00000164761	TNFRSF11B	3.000511675	8.692944449	-1.529479888	0.000441
ENSG00000258704	SRP54-AS1	0.573151186	1.666556468	-1.529540108	0.005794
ENSG00000175274	TP53I11	1.598599273	4.631718542	-1.529749113	0.000394
ENSG00000137502	RAB30	0.25427252	0.737728937	-1.530467928	0.001067
ENSG00000105404	RABAC1	1.295996972	3.761983236	-1.530910947	0.001218
ENSG00000178718	RPP25	0.945620237	2.74539318	-1.531358592	0.001081
ENSG00000266094	RASSF5	0.311474117	0.904759202	-1.531523712	0.001344
ENSG00000175920	DOK7	1.212825041	3.520195897	-1.531686387	0.000624
ENSG00000104142	VPS18	4.520135202	13.12270333	-1.532791942	0.000336
ENSG00000261762	AC027228.2	0.484975055	1.413134769	-1.533491259	0.004513
ENSG00000115840	SLC25A12	4.672468667	13.57875319	-1.534326216	0.000307
ENSG00000169515	CCDC8	3.459143494	10.05626225	-1.534608644	0.000376
ENSG00000106004	HOXA5	7.441579983	21.63419778	-1.534674669	0.000364
ENSG00000186834	HEXIM1	17.89810141	52.03712928	-1.53509878	0.000269
ENSG00000066468	FGFR2	0.159907373	0.466651707	-1.536350511	0.003682
ENSG00000262049	AC139530.1	0.464987312	1.3585166	-1.536674558	0.005225
ENSG00000275202	AL161421.1	0.83494997	2.439407191	-1.536674558	0.005225
ENSG00000179583	CIITA	0.044287706	0.129391883	-1.536674558	0.005225
ENSG00000258472	AC005726.1	0.428502136	1.251920751	-1.538515539	0.002797
ENSG00000148672	GLUD1	39.41060275	114.9161818	-1.539332505	0.00025
ENSG00000106948	AKNA	1.616077239	4.71657459	-1.540388328	0.00032
ENSG00000115239	ASB3	0.12603962	0.369532043	-1.540845679	0.006203
ENSG00000231185	SPRY4-AS1	0.438389599	1.285302232	-1.540845679	0.006203
ENSG00000168890	TMEM150A	1.539606137	4.499460546	-1.541561597	0.000577
ENSG00000114054	PCCB	11.01223045	32.17572256	-1.542247298	0.000249
ENSG00000143515	ATP8B2	0.126923583	0.37243485	-1.543161389	0.00474
ENSG00000177426	TGIF1	4.172446494	12.22247735	-1.54586519	0.000261
ENSG00000124201	ZNFX1	3.145093349	9.23059667	-1.548589775	0.000262
ENSG00000109107	ALDOC	7.477997335	21.95349834	-1.54893611	0.000276
ENSG00000246334	PRR7-AS1	1.645870194	4.842101376	-1.550396126	0.000951
ENSG00000259663	AC010478.1	0.146062118	0.431858979	-1.554515766	0.003962
ENSG00000082438	COBLL1	0.968134207	2.858606636	-1.5571673	0.000276
ENSG00000117115	PADI2	0.209485883	0.620878864	-1.558806507	0.002726
ENSG00000160213	CSTB	18.86982109	55.78611184	-1.559199083	0.000215
ENSG00000164778	EN2	0.221542891	0.659250692	-1.561857679	0.005928
ENSG00000128266	GNAZ	1.08816278	3.229006454	-1.563407167	0.000569
ENSG00000103599	IQCH	0.364476357	1.083181905	-1.564834152	0.000888
ENSG00000141295	SCRN2	3.789939011	11.25329882	-1.565097804	0.000291
ENSG00000174953	DHX36	3.659490812	10.8665636	-1.565490701	0.000218
ENSG00000136205	TNS3	2.706118876	8.036644258	-1.565649653	0.000223
ENSG00000111012	CYP27B1	0.546612001	1.626566932	-1.565945766	0.001252
ENSG00000139182	CLSTN3	0.859683281	2.555298472	-1.566189274	0.000395
ENSG00000163517	HDAC11	1.986015554	5.907952283	-1.567732672	0.000297

ENSG00000143499	SMYD2	6.615246244	19.68515284	-1.568524719	0.000217
ENSG00000149948	HMGA2	2.881006863	8.575989931	-1.569056916	0.000204
ENSG00000143224	PPOX	1.913768203	5.703484391	-1.570285174	0.000312
ENSG00000186010	NDUFA13	0.385759331	1.157096949	-1.575238278	0.003479
ENSG00000153558	FBXL2	1.248547524	3.738047763	-1.576936719	0.000275
ENSG00000162627	SNX7	11.62391591	34.80132414	-1.577273742	0.00021
ENSG00000198919	DZIP3	3.607980688	10.8119761	-1.578603983	0.000205
ENSG00000229043	AC091729.3	2.817700226	8.451778272	-1.579262184	0.000364
ENSG00000049246	PER3	0.696510168	2.089203618	-1.579262184	0.000364
ENSG00000067601	PMS2P4	1.222027333	3.67395435	-1.579507809	0.002289
ENSG00000251141	MRPS30-DT	0.680360121	2.044810185	-1.579600835	0.001927
ENSG00000124406	ATP8A1	1.563363146	4.688556305	-1.579638122	0.00022
ENSG00000196388	INCA1	1.103157425	3.314719269	-1.579671238	0.001532
ENSG00000179913	B3GNT3	7.681549618	23.07216977	-1.581834272	0.000217
ENSG00000171055	FEZ2	1.846638292	5.549064571	-1.582472302	0.000219
ENSG00000013563	DNASE1L1	1.618806809	4.866499216	-1.582621339	0.000331
ENSG00000110888	CAPRIN2	2.380057848	7.168927063	-1.585943808	0.0002
ENSG00000127955	GNAI1	0.609363964	1.836312489	-1.586526658	0.000216
ENSG00000273136	NBPF26	1.094512802	3.302319177	-1.587847344	0.000316
ENSG00000155636	RBM45	1.056864453	3.190682397	-1.588490627	0.000381
ENSG00000087074	PPP1R15A	10.9023145	32.89575694	-1.588556086	0.00018
ENSG00000073792	IGF2BP2	11.92231129	36.02793195	-1.590802871	0.000164
ENSG00000260285	AL133367.1	0.42165973	1.278002005	-1.592149121	0.001402
ENSG00000215440	NPEPL1	0.601544246	1.821925262	-1.593032554	0.000382
ENSG00000250067	YJEFN3	0.423644313	1.285861902	-1.593108638	0.002174
ENSG00000130940	CASZ1	0.154815537	0.469902215	-1.594290432	0.001348
ENSG00000116584	ARHGEF2	9.820838837	29.79797971	-1.596681362	0.000151
ENSG00000110047	EHD1	7.826313126	23.7589363	-1.59741482	0.000155
ENSG00000154803	FLCN	1.682349029	5.109381487	-1.597798166	0.000191
ENSG00000262655	SPON1	117.6577392	357.9119556	-1.600442896	0.00014
ENSG00000155850	SLC26A2	14.75066226	44.89315659	-1.601117783	0.000143
ENSG00000166313	APBB1	0.262951921	0.802390058	-1.601168936	0.001837
ENSG00000169562	GJB1	0.556240369	1.697351135	-1.601168936	0.001837
ENSG00000174804	FZD4	2.161999484	6.584416965	-1.601805686	0.000184
ENSG00000124120	TTPAL	2.710952725	8.267578318	-1.603834474	0.000175
ENSG00000175550	DRAP1	25.83625453	78.80577257	-1.604231066	0.000148
ENSG00000100418	DESI1	6.370348285	19.44656948	-1.605349636	0.000155
ENSG00000232442	MHENCR	3.964661995	12.12683828	-1.606731622	0.000475
ENSG00000124831	LRRFIP1	6.407734445	19.64146062	-1.61139037	0.000134
ENSG00000162576	MXRA8	0.438389599	1.347095608	-1.611715923	0.00145
ENSG00000160209	PDXK	11.31981612	34.72599144	-1.612569589	0.000129
ENSG00000114450	GNB4	0.938464992	2.882401457	-1.613546139	0.000255
ENSG00000205464	ATP6AP1L	0.398837474	1.226047607	-1.613768967	0.000576

ENSG00000261824	LINC00662	1.071152545	3.290578832	-1.614005886	0.000213
ENSG00000143198	MGST3	5.19769028	15.96361307	-1.614167703	0.000136
ENSG00000274964	AC026356.1	0.523472854	1.616354441	-1.614640289	0.004758
ENSG00000224536	AC096677.1	0.468570358	1.44682914	-1.614640289	0.004758
ENSG00000187634	SAMD11	0.170511921	0.526498554	-1.614640289	0.004758
ENSG00000246985	SOCS2-AS1	0.273751643	0.844587207	-1.615811167	0.002684
ENSG00000103811	CTSH	9.134361944	28.11750074	-1.617438097	0.000129
ENSG00000111885	MAN1A1	1.500076014	4.624510805	-1.619010506	0.000221
ENSG00000167393	PPP2R3B	0.579387836	1.787545636	-1.619816967	0.000284
ENSG00000139410	SDSL	5.845437606	18.03856855	-1.62073959	0.000169
ENSG00000229980	TOB1-AS1	0.369438606	1.145635974	-1.621583133	0.003863
ENSG00000163754	GYG1	7.271691943	22.47718143	-1.623335342	0.000137
ENSG00000250988	SNHG21	0.793645788	2.465585065	-1.624527078	0.003515
ENSG00000250479	CHCHD10	19.81736451	61.35180833	-1.625588652	0.000133
ENSG00000108819	PPP1R9B	8.827638271	27.33904029	-1.62616758	0.000125
ENSG00000117280	RAB29	8.234381718	25.51797756	-1.627060189	0.000127
ENSG00000164898	FMC1	0.57578377	1.791699764	-1.62718719	0.003215
ENSG00000166974	MAPRE2	1.724156949	5.34549903	-1.627382684	0.000177
ENSG00000175309	PHYKPL	2.441877687	7.570625602	-1.627608101	0.000139
ENSG00000146856	AGBL3	0.228742032	0.710622984	-1.62765982	0.001116
ENSG00000185686	PRAME	5.811221629	18.02382659	-1.628195039	0.000136
ENSG00000111816	FRK	0.587459852	1.823682747	-1.629058207	0.000199
ENSG00000120049	KCNIP2	0.159908996	0.500735542	-1.632566688	0.007946
ENSG00000227533	SLC2A1-AS1	0.161477663	0.505647632	-1.632566688	0.007946
ENSG00000253716	MINCR	2.26028061	7.041031995	-1.63306841	0.000385
ENSG00000271303	SRXN1	1.672657036	5.209644291	-1.633318069	0.000275
ENSG00000154710	RABGEF1	0.498795397	1.554443736	-1.633410232	0.000507
ENSG00000167880	EVPL	3.282745133	10.22220428	-1.63396079	0.000126
ENSG00000168487	BMP1	2.533637959	7.902898925	-1.636335764	0.000129
ENSG00000105643	ARRDC2	1.757205714	5.483168703	-1.636522029	0.000185
ENSG00000143248	RGS5	0.226662026	0.7087549	-1.63677603	0.001251
ENSG00000248124	RRN3P1	0.843186328	2.633124821	-1.637103879	0.000267
ENSG00000127528	KLF2	1.014612035	3.172015084	-1.637337546	0.00064
ENSG00000144724	PTPRG	1.299636345	4.057413338	-1.637617589	0.000128
ENSG00000230615	AL139220.2	0.078357515	0.246227949	-1.63888865	0.006036
ENSG00000234602	MCIDAS	1.039140813	3.253942869	-1.639810427	0.000601
ENSG00000168010	ATG16L2	0.378236644	1.184401974	-1.640619676	0.000359
ENSG00000230337	AL109811.2	1.247326888	3.9251245	-1.64145378	0.005337
ENSG00000237437	ASS1P12	0.544879553	1.714642814	-1.64145378	0.005337
ENSG00000096092	TMEM14A	17.27019638	54.10381374	-1.642580123	0.000128
ENSG00000109944	JHY	0.38392615	1.205160976	-1.642826733	0.00074
ENSG00000147459	DOCK5	1.115325941	3.500274198	-1.645148224	0.000123
ENSG00000120738	EGR1	0.798956993	2.510614871	-1.645183774	0.000489

ENSG00000210140	MT-TC	1051.600511	3302.297588	-1.646250149	9.72E-05
ENSG00000134108	ARL8B	10.87870656	34.1857064	-1.647193089	0.000101
ENSG00000141503	MINK1	6.617677886	20.80394296	-1.647800648	9.79E-05
ENSG00000106733	NMRK1	3.070392759	9.659105748	-1.648462918	0.000137
ENSG00000152527	PLEKHH2	0.492843141	1.55203097	-1.649306591	0.000231
ENSG00000101104	PABPC1L	3.320031802	10.45430858	-1.64995132	0.000119
ENSG00000198720	ANKRD13B	2.336028725	7.360946522	-1.650704672	0.00015
ENSG00000172878	METAP1D	1.490491336	4.697820428	-1.650913047	0.000166
ENSG00000116871	MAP7D1	4.502776709	14.19069945	-1.651278425	0.000106
ENSG00000105722	ERF	8.788260205	27.71373863	-1.652211678	0.000102
ENSG00000163191	S100A11	130.4566352	412.2156908	-1.655212291	8.81E-05
ENSG00000213742	ZNF337-AS1	0.354247416	1.121607992	-1.655360066	0.000647
ENSG00000141510	TP53	9.513389369	30.10178296	-1.657162459	8.94E-05
ENSG00000003249	DBNDD1	5.18938493	16.42529084	-1.657512735	0.000102
ENSG00000148082	SHC3	0.235671794	0.748381332	-1.660165676	0.000487
ENSG00000105355	PLIN3	15.71596406	49.83935229	-1.660377268	8.84E-05
ENSG00000176046	NUPR1	9.124457788	28.95323539	-1.66125411	8.63E-05
ENSG00000099282	TSPAN15	3.221554027	10.23495419	-1.66249455	0.000137
ENSG00000254615	AC027031.2	2.00240356	6.37219918	-1.66362839	0.00037
ENSG00000262879	AC068152.1	1.784742968	5.682295948	-1.665309152	0.000167
ENSG00000169087	HSPBAP1	1.222651209	3.904622711	-1.669810295	0.000154
ENSG00000260793	AC003102.1	0.269985517	0.867674829	-1.669924072	0.006493
ENSG00000249476	AC008467.1	0.099289135	0.319093723	-1.669924072	0.006493
ENSG00000222019	URAHP	0.267855454	0.860829268	-1.671222277	0.00502
ENSG00000167100	SAMD14	0.095291792	0.306247129	-1.671750313	0.004472
ENSG00000163898	LIPH	0.172332039	0.553837754	-1.672216535	0.00339
ENSG00000197948	FCHSD1	1.520596841	4.872126398	-1.674778232	0.000122
ENSG00000138161	CUZD1	0.313632136	1.007945587	-1.674803919	0.001716
ENSG00000167588	GPD1	0.28268784	0.908497339	-1.674985262	0.001436
ENSG00000128591	FLNC	0.130579534	0.419654268	-1.675310538	0.001299
ENSG00000266472	MRPS21	19.96331103	64.02135725	-1.676482073	7.98E-05
ENSG00000232859	LYRM9	0.357273371	1.148199039	-1.676935479	0.000539
ENSG00000269713	NBPF9	1.274904312	4.092317252	-1.677508295	0.000106
ENSG00000137767	SQOR	2.273301725	7.305898144	-1.67895331	0.00013
ENSG00000173614	NMNAT1	1.622471949	5.214272558	-1.679000128	0.000128
ENSG00000118200	CAMSAP2	4.061952009	13.15208097	-1.690319035	7.05E-05
ENSG00000076242	MLH1	5.595843207	18.12323454	-1.69058433	7.88E-05
ENSG00000251562	MALAT1	5.125568374	16.5992141	-1.69065541	6.62E-05
ENSG00000126790	L3HYPDH	2.444639671	7.920415415	-1.690770343	0.000107
ENSG00000174353	STAG3L3	0.355474471	1.154701829	-1.691004072	0.001064
ENSG00000096433	ITPR3	16.60410718	53.86541029	-1.693229259	6.01E-05
ENSG00000197415	VEPH1	2.148565289	6.973321258	-1.693533635	8.42E-05
ENSG00000003137	CYP26B1	0.716560073	2.329646028	-1.694898229	0.000202

ENSG00000041880	PARP3	5.608498001	18.22323818	-1.695208507	7.80E-05
ENSG00000169946	ZFPM2	0.118235359	0.385740086	-1.69557002	0.001934
ENSG00000138623	SEMA7A	0.494827706	1.613828344	-1.698071458	0.000467
ENSG00000103269	RHBDL1	0.931522788	3.041231216	-1.699369713	0.000493
ENSG00000280254	AC233723.2	0.33729946	1.105262271	-1.700165297	0.002863
ENSG00000167106	FAM102A	4.212795445	13.73714169	-1.700465494	6.72E-05
ENSG00000064225	ST3GAL6	1.01672614	3.317368771	-1.700708718	0.00012
ENSG00000169814	BTD	1.860311164	6.07061517	-1.70124585	8.53E-05
ENSG00000180530	NRIP1	3.958725181	12.91740321	-1.701491096	6.33E-05
ENSG00000163344	PMVK	21.0559498	68.75335764	-1.70244526	6.53E-05
ENSG00000167766	ZNF83	6.922602081	22.60459597	-1.702575861	5.80E-05
ENSG00000245498	AP000866.1	0.129126856	0.424207583	-1.702847101	0.004177
ENSG00000234665	AL512625.3	0.137938295	0.453858582	-1.70447029	0.004688
ENSG00000235092	ID2-AS1	0.649094381	2.129507744	-1.705432727	0.000919
ENSG00000265458	AC132938.3	1.191251886	3.926589646	-1.706338483	0.005308
ENSG00000172508	CARNS1	0.095804384	0.31578922	-1.706338483	0.005308
ENSG00000261238	AC009166.1	1.029620963	3.400894276	-1.708511682	0.009726
ENSG00000251867	AC009812.1	0.29989558	0.990571479	-1.708511682	0.009726
ENSG00000108187	PBLD	0.484750009	1.592500859	-1.709271053	0.000294
ENSG00000127561	SYNGR3	0.525372626	1.727699444	-1.709878067	0.000445
ENSG00000111846	GCNT2	0.783016604	2.575655827	-1.712506972	9.68E-05
ENSG00000179528	LBX2	0.419778492	1.386551287	-1.713859057	0.00149
ENSG00000149782	PLCB3	4.385620436	14.44611631	-1.715023317	6.07E-05
ENSG00000281376	ABALON	1.954232852	6.445136846	-1.715596844	0.000168
ENSG00000103381	CPPED1	4.253300046	14.02817147	-1.716919156	5.58E-05
ENSG00000113594	LIFR	0.496607262	1.641264165	-1.719153806	0.000105
ENSG00000197892	KIF13B	1.346219526	4.455364731	-1.721710439	6.24E-05
ENSG00000158186	MRAS	1.608395198	5.32375229	-1.721734937	7.43E-05
ENSG00000162946	DISC1	0.349669044	1.158444346	-1.722368363	0.000124
ENSG00000164638	SLC29A4	0.453017277	1.502118187	-1.722744158	0.000255
ENSG00000100889	PCK2	16.64834503	55.22459162	-1.725297302	4.59E-05
ENSG00000184470	TXNRD2	2.021152222	6.709389473	-1.726125733	5.86E-05
ENSG00000072778	ACADVL	19.01603042	63.21123286	-1.728349171	4.38E-05
ENSG00000134716	CYP2J2	0.363351746	1.212646448	-1.729169009	0.001044
ENSG00000169410	PTPN9	3.596685578	11.96863826	-1.729787047	4.90E-05
ENSG00000106367	AP1S1	32.62406207	108.5684148	-1.729944293	4.45E-05
ENSG00000263142	LRRC37A17P	0.749404024	2.496642396	-1.730187489	0.000148
ENSG00000108828	VAT1	30.1246723	100.2663246	-1.730211211	4.27E-05
ENSG00000111275	ALDH2	3.91631128	13.04209479	-1.730908485	4.67E-05
ENSG00000008311	AASS	0.97387132	3.246813246	-1.731448423	0.000115
ENSG00000203727	SAMD5	0.94546194	3.151833746	-1.731633207	9.11E-05
ENSG00000179743	FLJ37453	1.013799857	3.384837771	-1.732588359	0.000244
ENSG00000125746	EML2	3.023144845	10.08041122	-1.732656728	5.00E-05

ENSG00000124107	SLPI	106.9875803	356.8281752	-1.733143936	4.28E-05
ENSG00000186594	MIR22HG	0.825633488	2.758343751	-1.733466098	0.000245
ENSG00000143669	LYST	0.553901152	1.848584113	-1.733662435	6.26E-05
ENSG00000170442	KRT86	0.261813601	0.878808159	-1.733793532	0.003476
ENSG00000144821	MYH15	0.081463707	0.273442518	-1.733793532	0.003476
ENSG00000220412	AL356234.1	0.328834351	1.107126119	-1.737545223	0.00387
ENSG00000160188	RSPH1	0.195781898	0.659162439	-1.737545223	0.00387
ENSG00000253981	ALG1L13P	0.635300564	2.134523438	-1.737929965	0.00146
ENSG00000010379	SLC6A13	0.295442735	0.991688302	-1.738110355	0.000794
ENSG00000197576	HOXA4	0.90889933	3.049119355	-1.738215038	0.000523
ENSG00000087077	TRIP6	26.55956024	88.91917803	-1.738633183	4.01E-05
ENSG00000142794	NBPF3	1.061198324	3.555027742	-1.738855721	7.50E-05
ENSG00000152556	PFKM	9.792572841	32.79855971	-1.739239601	4.00E-05
ENSG00000166750	SLFN5	2.16555469	7.264759172	-1.741386229	4.64E-05
ENSG00000107902	LHPP	1.531996103	5.143793788	-1.741987181	8.34E-05
ENSG00000205213	LGR4	10.58476876	35.53514082	-1.742618419	3.89E-05
ENSG00000180861	LINC01559	0.211411337	0.71340164	-1.743587818	0.001652
ENSG00000139687	RB1	2.836644053	9.546505862	-1.745932296	4.76E-05
ENSG00000118257	NRP2	0.099231425	0.33501473	-1.746841608	0.000651
ENSG00000196843	ARID5A	0.621214275	2.096897999	-1.748070898	0.000252
ENSG00000248527	MTATP6P1	167.6326747	565.1590641	-1.748748769	3.56E-05
ENSG00000167550	RHEBL1	1.036538313	3.504709549	-1.748881566	0.00067
ENSG00000217555	CKLF	0.647836446	2.197672654	-1.750464081	0.001897
ENSG00000259479	SORD2P	0.257141235	0.872306992	-1.750464081	0.001897
ENSG00000261270	AC012181.2	0.764791944	2.606837385	-1.75274534	0.00927
ENSG00000142871	CYR61	13.12284421	44.4896202	-1.756661336	3.76E-05
ENSG00000004660	CAMKK1	0.3996184	1.356640954	-1.756941128	0.000168
ENSG00000234719	NPIPB2	0.338094857	1.152415792	-1.758652476	0.001269
ENSG00000185100	ADSSL1	0.571610495	1.945548677	-1.760854801	0.000125
ENSG00000101096	NFATC2	0.368131536	1.253324197	-1.761111859	0.000129
ENSG00000224086	AC245452.1	0.04857233	0.165561584	-1.761622816	0.00029
ENSG00000106714	CNTNAP3	0.618816149	2.106278609	-1.761625506	6.97E-05
ENSG00000125503	PPP1R12C	4.272651579	14.53671667	-1.761774709	3.53E-05
ENSG00000138772	ANXA3	10.02426683	34.11189306	-1.7621143	3.28E-05
ENSG00000159216	RUNX1	0.662707483	2.256263692	-1.762402934	5.06E-05
ENSG00000159714	ZDHHC1	0.708227979	2.415185394	-1.763103207	0.00019
ENSG00000104714	ERICH1	1.074065668	3.663525096	-1.765067632	4.96E-05
ENSG00000129667	RHBDF2	1.635450196	5.585692862	-1.767006218	4.55E-05
ENSG00000167702	KIFC2	1.390655883	4.751111663	-1.767054251	6.48E-05
ENSG00000256525	POLG2	2.590007278	8.85221125	-1.767898434	5.19E-05
ENSG00000108602	ALDH3A1	15.26160797	52.14366734	-1.767936213	3.06E-05
ENSG00000231925	TAPBP	3.933915063	13.44841421	-1.768564998	3.68E-05
ENSG00000280832	GSEC	0.392825887	1.346620913	-1.769069558	0.00046

ENSG00000210144	MT-TY	54.44770856	186.5130082	-1.770246036	0.000107
ENSG00000198113	TOR4A	4.885714469	16.72681751	-1.770693133	3.58E-05
ENSG00000138678	GPAT3	2.132648438	7.305630631	-1.771061933	5.50E-05
ENSG00000160781	PAQR6	1.096461193	3.762378025	-1.772176363	0.000158
ENSG00000186868	MAPT	0.324873478	1.11607784	-1.774794864	7.53E-05
ENSG00000172831	CES2	6.279939539	21.56471569	-1.775129019	3.11E-05
ENSG00000188191	PRKAR1B	8.625052962	29.64273541	-1.77637417	3.01E-05
ENSG00000265683	SYPL1P2	0.74719513	2.586040058	-1.776520783	0.003553
ENSG00000173548	SNX33	1.513209381	5.204104214	-1.777026508	3.96E-05
ENSG00000134250	NOTCH2	3.823765292	13.15434399	-1.777830712	2.76E-05
ENSG00000181458	TMEM45A	0.417506585	1.438681522	-1.778121959	0.000167
ENSG00000175471	MCTP1	1.883784487	6.483598855	-1.778361871	3.25E-05
ENSG00000116675	DNAJC6	0.522768797	1.802369018	-1.779576039	9.79E-05
ENSG00000137941	TTLL7	0.471636976	1.626128778	-1.78002684	6.93E-05
ENSG00000176401	EID2B	0.69455709	2.400167397	-1.780160218	0.000556
ENSG00000178700	DHFR2	1.228720059	4.246683818	-1.783659163	5.70E-05
ENSG00000135736	CCDC102A	2.848272399	9.847193367	-1.784317682	4.87E-05
ENSG00000188177	ZC3H6	0.623082211	2.154151764	-1.784317682	4.87E-05
ENSG00000104324	CPQ	0.785668007	2.719194565	-1.784598406	0.000141
ENSG00000085063	CD59	5.893854369	20.39362546	-1.786187504	2.55E-05
ENSG00000127920	GNG11	6.797829469	23.56108023	-1.788436431	3.05E-05
ENSG00000107796	ACTA2	0.280816201	0.977689156	-1.788590683	0.001225
ENSG00000083720	OXCT1	12.0155588	41.69993264	-1.790474587	2.49E-05
ENSG00000169871	TRIM56	1.009762079	3.50556983	-1.790586285	3.65E-05
ENSG00000104518	GSDMD	4.1083037	14.26190164	-1.790731446	2.99E-05
ENSG00000253854	AC010834.3	0.667499213	2.335881764	-1.793763436	0.002412
ENSG00000285184	AC244033.2	0.191735014	0.670967567	-1.793763436	0.002412
ENSG00000188707	ZBED6CL	1.65261244	5.753732349	-1.79405055	6.34E-05
ENSG00000157827	FMNL2	5.755995518	20.02976327	-1.794303461	2.54E-05
ENSG00000145819	ARHGAP26	0.496808017	1.729685853	-1.794543063	4.02E-05
ENSG00000188372	ZP3	1.061559684	3.70828514	-1.798087892	0.000121
ENSG00000224934	AL391684.1	0.157498075	0.551589644	-1.798608759	0.000638
ENSG00000172380	GNG12	24.95274272	87.11942068	-1.799191978	2.16E-05
ENSG00000124228	DDX27	5.314110835	18.56311985	-1.799790762	2.49E-05
ENSG00000273311	DGCR11	0.547325236	1.920730165	-1.80205405	0.000523
ENSG00000139192	TAPBPL	0.720890775	2.527403373	-1.802832552	0.000146
ENSG00000225377	NRSN2-AS1	1.193870022	4.194944001	-1.803143214	0.000658
ENSG00000277559	AC018553.1	0.286594312	1.013157081	-1.803988285	0.008765
ENSG00000224183	SDHDP6	0.870530224	3.077464635	-1.803988285	0.008765
ENSG00000196876	SCN8A	0.058754233	0.206806523	-1.804643415	0.001005
ENSG00000148057	IDNK	1.911903204	6.712000647	-1.805575223	7.94E-05
ENSG00000136048	DRAM1	0.459839889	1.617041009	-1.806707041	0.000178
ENSG00000247903	AC024896.1	1.688466625	5.935635631	-1.807840688	5.90E-05

ENSG00000005448	WDR54	3.681794962	12.94374191	-1.808688589	3.26E-05
ENSG00000103034	NDRG4	0.272881041	0.960502358	-1.808848981	0.000117
ENSG00000214176	PLEKHM1P1	0.991719873	3.488447337	-1.809361445	3.49E-05
ENSG00000087086	FTL	1264.414414	4446.589075	-1.809667759	1.87E-05
ENSG00000145700	ANKRD31	0.086595068	0.306841104	-1.810371812	0.00291
ENSG00000139194	RBP5	0.396793908	1.406000178	-1.810371812	0.00291
ENSG00000197927	C2orf27A	0.553665365	1.95380735	-1.810733799	0.000349
ENSG00000139289	PHLDA1	2.563365736	9.031399498	-1.812084205	2.41E-05
ENSG00000087253	LPCAT2	1.881721468	6.631127894	-1.812246635	2.70E-05
ENSG00000132334	PTPRE	0.200837948	0.70919766	-1.813133414	0.000136
ENSG00000108797	CNTNAP1	0.158985172	0.562778705	-1.813336246	0.000803
ENSG00000135362	PRR5L	0.751582322	2.652228508	-1.813525649	5.13E-05
ENSG00000118762	PKD2	2.447238113	8.646496094	-1.815990107	2.62E-05
ENSG00000116141	MARK1	0.146561485	0.520597505	-1.817101476	0.00111
ENSG00000204410	MSH5	0.127353796	0.452370337	-1.817101476	0.00111
ENSG00000155367	PPM1J	0.276243411	0.981237538	-1.817101476	0.00111
ENSG00000113924	HGD	5.435050249	19.23778825	-1.818676816	2.38E-05
ENSG00000163082	SGPP2	3.857889339	13.65972966	-1.819055056	2.64E-05
ENSG00000258534	AL132712.1	0.861555686	3.076503228	-1.820633971	0.005308
ENSG00000162620	LRRIQ3	0.14788777	0.526384469	-1.822753815	0.000404
ENSG00000234327	AC012146.1	0.62490704	2.231461713	-1.827266501	0.000411
ENSG00000197872	FAM49A	0.253105704	0.903807529	-1.827670229	0.000353
ENSG00000153786	ZDHHC7	14.58838387	51.96468833	-1.828057508	1.69E-05
ENSG00000118873	RAB3GAP2	2.468185461	8.797701754	-1.828899944	1.92E-05
ENSG00000178209	PLEC	5.157088473	18.41529177	-1.831654759	1.58E-05
ENSG00000145002	FAM86B2	1.254175316	4.489419992	-1.831969952	0.000229
ENSG00000238018	AC093110.1	0.777560977	2.788641093	-1.832165515	0.000703
ENSG00000249637	AC008438.1	0.837231254	3.016822427	-1.832647221	0.006069
ENSG00000188522	FAM83G	2.318466478	8.292203522	-1.833721116	2.04E-05
ENSG00000137819	PAQR5	1.899948998	6.796457303	-1.833791862	2.41E-05
ENSG00000139625	MAP3K12	0.718179244	2.572012277	-1.83494661	3.57E-05
ENSG00000170017	ALCAM	9.452510382	33.85137054	-1.835805323	1.54E-05
ENSG00000134504	KCTD1	2.121733651	7.599953605	-1.83589285	1.93E-05
ENSG00000263412	AC004477.1	0.313426497	1.129379824	-1.838688366	0.000728
ENSG00000271576	AL359504.2	0.574191079	2.069001265	-1.838688366	0.000728
ENSG00000272899	ATP6V1FNB	0.257009436	0.929219087	-1.844052322	0.000524
ENSG00000143570	SLC39A1	0.43166788	1.55904616	-1.844278584	0.000258
ENSG00000170917	NUDT6	0.583259213	2.105381248	-1.845854894	5.11E-05
ENSG00000266338	NBPF15	1.992408413	7.187745931	-1.845937771	2.26E-05
ENSG00000131187	F12	2.671009312	9.650987292	-1.847974813	2.64E-05
ENSG00000166349	RAG1	0.122977014	0.446772043	-1.850752198	0.000615
ENSG00000070159	PTPN3	7.366279563	26.75934504	-1.856401307	1.24E-05
ENSG00000005238	FAM214B	1.095327778	3.981117104	-1.856451434	2.47E-05

ENSG00000103021	CCDC113	1.12800272	4.100957521	-1.856790862	2.72E-05
ENSG00000181722	ZBTB20	0.111438357	0.405890507	-1.858701576	4.91E-05
ENSG00000248932	AC097103.2	0.630723785	2.301500139	-1.858727809	0.000291
ENSG00000020577	SAMD4A	0.288412314	1.051951757	-1.860701025	4.84E-05
ENSG00000123146	ADGRE5	3.454947155	12.61717875	-1.863793352	1.46E-05
ENSG00000149289	ZC3H12C	0.649202908	2.37195265	-1.863882586	2.60E-05
ENSG00000236397	DDX11L2	0.269005048	0.992601417	-1.864091578	0.008204
ENSG00000133863	TEX15	0.031698336	0.116963653	-1.864091578	0.008204
ENSG00000227036	LINC00511	0.37085439	1.355622893	-1.86492281	1.87E-05
ENSG00000183255	PTTG1IP	62.76677972	229.4726965	-1.865667042	1.08E-05
ENSG00000108679	LGALS3BP	7.041967706	25.76627249	-1.866652601	1.31E-05
ENSG00000140400	MAN2C1	0.932425285	3.412808577	-1.866680018	2.01E-05
ENSG00000086300	SNX10	1.460946687	5.356814766	-1.868815692	2.99E-05
ENSG00000187653	TMSB4XP8	110.808825	406.1838845	-1.869125485	1.49E-05
ENSG00000113645	WWC1	5.109138084	18.7253433	-1.869173611	1.13E-05
ENSG00000268996	MAN1B1-DT	0.669638634	2.462928185	-1.869860098	0.000296
ENSG00000242247	ARFGAP3	2.718958641	9.982666063	-1.871198961	1.89E-05
ENSG00000204237	OXLD1	5.797731293	21.29045988	-1.871480233	1.89E-05
ENSG00000169231	THBS3	0.659817847	2.430357692	-1.874617537	4.79E-05
ENSG00000236307	EEF1E1P1	1.263281069	4.684512763	-1.875780372	0.001955
ENSG00000260924	LINC01311	0.636179873	2.35432337	-1.877100588	0.000552
ENSG00000184903	IMMP2L	1.457774447	5.380498389	-1.878276377	2.83E-05
ENSG00000156253	RWDD2B	3.766229141	13.89700458	-1.878581257	1.47E-05
ENSG00000173715	C11orf80	1.265216836	4.672485241	-1.879059353	2.83E-05
ENSG00000177483	RBM44	0.103617418	0.384804487	-1.879288443	0.00117
ENSG00000266028	SRGAP2	1.487482075	5.491514101	-1.879531709	1.19E-05
ENSG00000108784	NAGLU	3.811322739	14.08160794	-1.880396414	1.52E-05
ENSG00000134897	BIVM	2.379651197	8.795244436	-1.880980391	1.43E-05
ENSG00000231991	ANXA2P2	26.21832204	96.94287884	-1.881790598	1.12E-05
ENSG00000156463	SH3RF2	1.034236129	3.831356816	-1.883974432	1.88E-05
ENSG00000205155	PSENEN	1.222988803	4.536966833	-1.884247315	7.42E-05
ENSG00000271327	AC010201.2	0.223601074	0.838372853	-1.885227483	0.009731
ENSG00000267141	AC012615.4	0.550714343	2.064855708	-1.885227483	0.009731
ENSG00000152778	IFIT5	0.083258681	0.312171212	-1.885227483	0.009731
ENSG00000124140	SLC12A5	0.037522012	0.140685533	-1.885227483	0.009731
ENSG00000267317	AC027307.2	0.963221148	3.586946107	-1.885805244	0.000568
ENSG00000189171	S100A13	15.33013153	56.94370073	-1.888442042	1.01E-05
ENSG00000282386	AL358472.4	0.588527475	2.206632773	-1.888576233	0.005615
ENSG00000152104	PTPN14	2.873919358	10.6824341	-1.88948659	9.10E-06
ENSG00000186577	SMIM29	2.174568097	8.093108188	-1.890236026	2.54E-05
ENSG00000254978	ALG1L9P	0.913343186	3.424501134	-1.890816271	0.003553
ENSG00000176907	TCIM	0.315531991	1.183059858	-1.892420008	0.001496
ENSG00000213185	FAM24B	0.639029833	2.395986987	-1.894563154	0.000727

ENSG00000130589	HELZ2	0.557554912	2.088648903	-1.89998062	1.77E-05
ENSG00000068697	LAPTM4A	110.4544495	413.9783689	-1.901520404	7.49E-06
ENSG00000092871	RFFL	1.565972602	5.874107992	-1.902326268	1.11E-05
ENSG00000244026	FAM86DP	2.29324454	8.616839252	-1.904525836	1.38E-05
ENSG00000168026	TTC21A	0.146959381	0.553926871	-1.904673977	0.000271
ENSG00000147003	CLTRN	0.353585198	1.336877709	-1.90616992	0.000755
ENSG00000139187	KLRG1	0.273422888	1.033790346	-1.90616992	0.000755
ENSG00000163040	CCDC74A	0.841955784	3.171387906	-1.906539883	5.09E-05
ENSG00000147257	GPC3	0.955624473	3.599542821	-1.906539883	5.09E-05
ENSG00000233122	CTAGE7P	0.188686354	0.716650967	-1.9083551	0.003981
ENSG00000237424	FOXD2-AS1	0.183196476	0.695799823	-1.9083551	0.003981
ENSG00000258101	AC010173.1	0.258822475	0.985836134	-1.909720117	0.006485
ENSG00000183250	LINC01547	1.274330388	4.820125332	-1.913086449	3.06E-05
ENSG00000132359	RAP1GAP2	3.446051118	13.04253514	-1.915430979	8.05E-06
ENSG00000171552	BCL2L1	22.6705614	85.83875681	-1.916175993	6.72E-06
ENSG00000169136	ATF5	4.823574377	18.32045174	-1.920321785	9.26E-06
ENSG00000100271	TTLL1	1.93517525	7.370942896	-1.923135881	2.79E-05
ENSG00000189283	FHIT	0.314985012	1.201584079	-1.923684016	9.90E-05
ENSG00000267213	AC007773.1	0.515869762	1.980261568	-1.9246673	0.00291
ENSG00000225177	FLJ46906	0.411004434	1.577716594	-1.9246673	0.00291
ENSG00000159899	NPR2	0.400015958	1.528779327	-1.925989314	0.000123
ENSG00000149243	KLHL35	1.400861174	5.34564559	-1.926681281	1.24E-05
ENSG00000247796	AC008966.1	0.667206634	2.550685096	-1.927432866	5.38E-05
ENSG00000278864	AC055811.4	2.525232486	9.64678455	-1.927780277	1.88E-05
ENSG00000074964	ARHGEF10L	2.207674448	8.43495953	-1.928964264	7.68E-06
ENSG00000262583	TMEM231P1	0.503439166	1.941533001	-1.929080261	0.004498
ENSG00000186522	10-Sep	14.22182129	54.43958478	-1.93189515	5.81E-06
ENSG00000218336	TENM3	0.191788349	0.735903783	-1.932947711	4.76E-05
ENSG00000219507	FTH1P8	4.146027215	15.93706482	-1.935332831	5.02E-05
ENSG00000285877	AC007448.4	0.133127681	0.516977514	-1.935639665	0.007582
ENSG00000164949	GEM	0.126240033	0.490230565	-1.935639665	0.007582
ENSG00000141682	PMAIP1	4.113109247	15.82231157	-1.938476551	9.70E-06
ENSG00000232187	FTH1P7	7.418431475	28.62569212	-1.942162246	1.91E-05
ENSG00000115255	REEP6	0.699552259	2.705162127	-1.943296692	8.39E-05
ENSG00000259065	AC005520.2	0.668081335	2.602507091	-1.944769511	0.003227
ENSG00000172828	CES3	0.719708878	2.784656219	-1.945835367	2.11E-05
ENSG00000135828	RNASEL	0.163730746	0.635819038	-1.946997525	0.000243
ENSG00000105971	CAV2	1.122161975	4.343173254	-1.947170135	9.89E-06
ENSG00000163755	HPS3	2.457967692	9.523611786	-1.949110923	6.66E-06
ENSG00000204248	COL11A2	0.185988653	0.72391399	-1.951450789	0.000156
ENSG00000204856	FAM216A	3.673112635	14.25981037	-1.95175224	7.85E-06
ENSG00000103254	FAM173A	4.063379767	15.78231757	-1.952261581	9.42E-06
ENSG00000196074	SYCP2	0.109859676	0.429216449	-1.953382047	0.000544

ENSG00000283378	CNTNAP3C	1.063981385	4.144733515	-1.95411229	5.39E-05
ENSG00000171658	NMRAL2P	0.572403435	2.231342837	-1.954417299	0.000101
ENSG00000088387	DOCK9	4.044299162	15.72570531	-1.954509799	4.58E-06
ENSG00000061938	TNK2	0.820754588	3.192841908	-1.954675874	7.68E-06
ENSG00000170876	TMEM43	12.21276892	47.50755749	-1.955077387	4.70E-06
ENSG00000181634	TNFSF15	0.121823472	0.476625966	-1.957507918	0.000244
ENSG00000263934	SNORD3A	0.717346794	2.817707248	-1.957742233	0.002384
ENSG00000242622	AC092910.3	0.552314527	2.162666786	-1.959928601	0.000154
ENSG00000275464	FP565260.1	0.626918048	2.452773806	-1.962305	1.25E-05
ENSG00000117266	CDK18	0.764629095	2.994692832	-1.963911422	1.18E-05
ENSG00000031081	ARHGAP31	0.249359041	0.977881501	-1.964406585	3.32E-05
ENSG00000007255	TRAPPC6A	12.58489996	49.32099612	-1.965458233	6.39E-06
ENSG00000275832	ARHGAP23	5.086388325	19.95159438	-1.967093584	4.17E-06
ENSG00000139211	AMIGO2	2.355448817	9.244557796	-1.967423877	7.19E-06
ENSG00000241889	AC079944.2	0.398498849	1.585614674	-1.968164901	0.008985
ENSG00000117148	ACTL8	0.156919611	0.624378311	-1.968164901	0.008985
ENSG00000224287	MSL3P1	0.128401297	0.510904816	-1.968164901	0.008985
ENSG00000019144	PHLDB1	1.142646062	4.486203688	-1.968326189	4.73E-06
ENSG00000258701	LINC00638	0.165946985	0.657758345	-1.968478114	0.003605
ENSG00000180447	GAS1	0.192150994	0.760038859	-1.96864816	0.001083
ENSG00000258512	LINC00239	0.850095242	3.362488035	-1.96864816	0.001083
ENSG00000232284	GNG12-AS1	0.19294869	0.762200345	-1.968754938	0.000558
ENSG00000197122	SRC	3.876201369	15.22555169	-1.968991032	4.58E-06
ENSG00000135678	CPM	0.593370037	2.33469236	-1.970375619	1.21E-05
ENSG00000105991	HOXA1	0.674755211	2.662162826	-1.972224671	6.56E-05
ENSG00000123836	PFKFB2	1.905485398	7.519783066	-1.97567688	4.66E-06
ENSG00000197442	MAP3K5	1.040105506	4.110812822	-1.977084628	1.00E-05
ENSG00000152763	WDR78	0.286714012	1.135103033	-1.977564239	4.16E-05
ENSG00000165983	PTER	2.390424238	9.449122672	-1.97775005	6.03E-06
ENSG00000090776	EFNB1	3.454801903	13.65585887	-1.977781129	5.67E-06
ENSG00000114738	MAPKAPK3	22.82694097	90.21121901	-1.977947517	3.47E-06
ENSG00000002834	LASP1	24.03802591	95.02891916	-1.978457052	3.36E-06
ENSG00000204444	APOM	1.283769873	5.096524127	-1.981845179	3.33E-05
ENSG00000213062	AL021068.1	0.36694139	1.474087441	-1.984349605	0.005912
ENSG00000159958	TNFRSF13C	0.084757507	0.340490279	-1.984349605	0.005912
ENSG00000237149	ZNF503-AS2	0.312018001	1.243420054	-1.984498784	0.000168
ENSG00000163393	SLC22A15	0.971203787	3.865598401	-1.987156998	9.41E-06
ENSG00000114770	ABCC5	2.24248464	8.92445426	-1.987882062	3.77E-06
ENSG00000106133	NSUN5P2	0.308200141	1.233766172	-1.989257289	0.000329
ENSG00000183628	DGCR6	0.74645237	2.977402639	-1.989893443	1.28E-05
ENSG00000270392	PFN1P2	0.361583133	1.452562097	-1.992953314	0.000471
ENSG00000186952	TMEM232	0.056803394	0.229362436	-1.998321531	0.000891
ENSG00000172748	ZNF596	0.361658362	1.452864311	-1.998960372	2.79E-05

ENSG00000115266	APC2	0.109351299	0.440176616	-2.000327991	7.96E-05
ENSG00000100490	CDKL1	0.251433026	1.011883876	-2.000469785	6.57E-05
ENSG00000115306	SPTBN1	13.6597596	54.89191528	-2.002081551	2.58E-06
ENSG00000165698	SPACA9	0.799296614	3.218885497	-2.002615303	2.43E-05
ENSG00000102024	PLS3	24.8735057	99.99900166	-2.00268962	2.65E-06
ENSG00000159423	ALDH4A1	2.401332491	9.65883575	-2.002797995	5.07E-06
ENSG00000063438	AHRR	0.14149801	0.571344875	-2.003655828	0.000134
ENSG00000140950	TLDC1	1.090856847	4.394565595	-2.005045395	4.96E-06
ENSG00000073849	ST6GAL1	0.273973013	1.105503832	-2.005712002	2.14E-05
ENSG00000157064	NMNAT2	2.181124715	8.796437644	-2.006916133	3.63E-06
ENSG00000187193	MT1X	1.932547638	7.801356281	-2.006958762	1.21E-05
ENSG00000239521	CASTOR3	0.342925324	1.386793479	-2.00773537	5.01E-05
ENSG00000176842	IRX5	1.365921589	5.522620671	-2.009416896	1.07E-05
ENSG00000273151	AC073957.3	0.228775034	0.92732126	-2.010802774	6.01E-05
ENSG00000178531	CTXN1	6.461329513	26.14110105	-2.011149279	4.65E-06
ENSG00000119698	PPP4R4	0.126011613	0.512967019	-2.011445166	0.00048
ENSG00000029534	ANK1	1.164899572	4.713871415	-2.011684625	3.73E-06
ENSG00000080493	SLC4A4	0.186736061	0.758302731	-2.013961012	3.27E-05
ENSG00000151914	DST	0.200336471	0.812990737	-2.015566464	4.48E-06
ENSG00000115310	RTN4	13.95573679	56.65458949	-2.016714415	2.29E-06
ENSG00000118515	SGK1	0.225822092	0.918378703	-2.016751108	2.14E-05
ENSG00000114541	FRMD4B	1.501148486	6.096528469	-2.016973768	3.27E-06
ENSG00000169855	ROBO1	0.941373373	3.823902556	-2.017077581	3.97E-06
ENSG00000159166	LAD1	14.76051247	60.04840399	-2.01971208	2.32E-06
ENSG00000150867	PIP4K2A	4.196612684	17.08529537	-2.020626397	2.77E-06
ENSG00000174306	ZHX3	3.847382524	15.66574452	-2.020976049	2.32E-06
ENSG00000218537	MIF-AS1	0.198169482	0.818837597	-2.022365357	0.006892
ENSG00000021826	CPS1	0.860916016	3.518734506	-2.025922482	3.93E-06
ENSG00000141968	VAV1	0.388250414	1.590884474	-2.026703387	4.25E-05
ENSG00000187595	ZNF385C	0.104503821	0.430580242	-2.027396859	0.000604
ENSG00000186056	MATN1-AS1	0.2492174	1.023411337	-2.027507176	0.00013
ENSG00000167105	TMEM92	0.471619083	1.934298079	-2.027548404	5.72E-05
ENSG00000104870	FCGRT	2.102790477	8.624382952	-2.03088946	3.74E-06
ENSG00000117226	GBP3	0.136140635	0.562533997	-2.032268854	0.000489
ENSG00000082458	DLG3	1.097374153	4.50826955	-2.033278235	3.70E-06
ENSG00000253741	LNCOC1	0.110271246	0.456766551	-2.0364835	0.000403
ENSG00000253210	AC040970.1	0.466008745	1.941399342	-2.038537629	0.003217
ENSG00000133030	MPRIP	11.91666824	49.35952252	-2.045763314	1.62E-06
ENSG00000128872	TMOD2	0.064638889	0.269286571	-2.046289899	0.000243
ENSG00000245275	SAP30L-AS1	0.233083143	0.976072132	-2.04880284	0.001727
ENSG00000130956	HABP4	1.49380926	6.213421515	-2.050860861	4.01E-06
ENSG00000222041	CYTOR	0.318828681	1.328245014	-2.05306421	4.13E-06
ENSG00000163923	RPL39L	9.145494883	38.09088303	-2.053146405	2.74E-06

ENSG00000225663	MCRIP1	2.455988652	10.24770344	-2.055722126	2.89E-06
ENSG00000065717	TLE2	0.331137797	1.385683567	-2.056123161	5.25E-05
ENSG00000008294	SPAG9	4.656023227	19.42741196	-2.056260506	1.55E-06
ENSG00000270177	AC104109.2	0.41255029	1.736228285	-2.058682469	0.000407
ENSG00000105639	JAK3	0.085864716	0.36136382	-2.058682469	0.000407
ENSG00000277363	SRCIN1	0.050179817	0.211183025	-2.058682469	0.000407
ENSG00000176809	LRRC37A3	0.306240707	1.282963448	-2.059659187	1.39E-05
ENSG00000144061	NPHP1	0.341018726	1.430836849	-2.06290431	6.03E-06
ENSG00000213398	LCAT	0.254692271	1.074314927	-2.063204488	0.000285
ENSG00000125968	ID1	49.17330823	206.2806642	-2.06400668	1.41E-06
ENSG00000137845	ADAM10	7.463902882	31.31591245	-2.064287795	1.35E-06
ENSG00000085662	AKR1B1	21.80606857	91.55648388	-2.065301226	1.36E-06
ENSG00000182718	ANXA2	45.45067502	191.4962705	-2.070370954	1.22E-06
ENSG00000270021	AC026691.1	0.124299804	0.532630108	-2.071288621	0.008135
ENSG00000230074	AL162231.2	0.377011586	1.615511169	-2.071288621	0.008135
ENSG00000276840	PMS2P10	0.409661282	1.755416547	-2.071288621	0.008135
ENSG00000229298	TUBB8P1	0.344859291	1.477737176	-2.071288621	0.008135
ENSG00000150403	TMCO3	4.55717177	19.21799067	-2.071564862	1.33E-06
ENSG00000258561	AL359232.1	0.162770259	0.697477693	-2.074603077	0.005291
ENSG00000224950	AL390066.1	0.127062622	0.544468903	-2.074603077	0.005291
ENSG00000228436	AL139260.1	0.342503695	1.467643343	-2.077097053	0.003603
ENSG00000261572	AC097639.1	0.198873113	0.852180006	-2.079041657	0.002551
ENSG00000226419	SLC16A1-AS1	0.037094995	0.158953678	-2.079041657	0.002551
ENSG00000140280	LYSMD2	1.652076645	7.01447186	-2.079763864	5.74E-06
ENSG00000269843	AC008537.2	0.866918066	3.714781905	-2.080600404	0.001866
ENSG00000203999	LINC01270	0.138637859	0.594069303	-2.080600404	0.001866
ENSG00000197140	ADAM32	0.04576264	0.196094919	-2.081877773	0.001404
ENSG00000273783	AL136040.1	0.406761025	1.74298882	-2.081877773	0.001404
ENSG00000250072	SH3TC2-DT	0.106570823	0.456660646	-2.081877773	0.001404
ENSG00000272631	AC067750.1	0.082996312	0.355642833	-2.083846538	0.000411
ENSG00000144815	NXPE3	0.866195215	3.685764877	-2.083848129	2.45E-06
ENSG00000174500	GCSAM	0.165683786	0.709962283	-2.085293066	0.000284
ENSG00000231764	DLX6-AS1	0.256244573	1.092636777	-2.086268248	4.50E-06
ENSG00000163686	ABHD6	1.257405595	5.363093464	-2.086740372	3.72E-06
ENSG00000026103	FAS	7.928722963	33.83809948	-2.088754657	1.21E-06
ENSG00000175938	ORAI3	0.837922395	3.590534187	-2.092065512	1.09E-05
ENSG00000119242	CCDC92	1.051877454	4.501897344	-2.092320969	1.99E-06
ENSG00000171084	FAM86JP	1.609046734	6.894835776	-2.093283592	4.29E-06
ENSG00000173559	NABP1	0.998783047	4.292798311	-2.098685648	1.41E-06
ENSG00000073670	ADAM11	0.297841787	1.285649415	-2.10112706	3.18E-05
ENSG00000270194	AC097359.2	0.542213886	2.342147445	-2.101739859	3.76E-05
ENSG00000133466	C1QTNF6	0.590834457	2.550551678	-2.104012062	3.79E-06
ENSG00000228801	AC064807.1	0.447338159	1.938646158	-2.10457178	8.44E-05

ENSG00000238278	ALG1L6P	2.811422149	12.16517563	-2.106027172	9.95E-06
ENSG00000203865	ATP1A1-AS1	0.288996201	1.256572586	-2.107425326	0.000176
ENSG00000225190	PLEKHM1	1.905304318	8.266124006	-2.112292578	1.10E-06
ENSG00000204531	POU5F1	0.432860332	1.885739254	-2.113833472	3.61E-05
ENSG00000171298	GAA	5.194816512	22.61596833	-2.117416552	9.19E-07
ENSG00000134030	CTIF	0.93372427	4.067365156	-2.117677384	1.69E-06
ENSG00000204556	AL450124.1	1.065351436	4.691885305	-2.11843951	0.002025
ENSG00000224358	AL451074.2	0.17491584	0.770342099	-2.11843951	0.002025
ENSG00000175084	DES	0.152625429	0.672173503	-2.11843951	0.002025
ENSG00000134107	BHLHE40	3.964009402	17.27760845	-2.118920477	1.09E-06
ENSG00000274512	TBC1D3L	0.427520181	1.871767555	-2.119558755	6.76E-05
ENSG00000185761	ADAMTSL5	0.25359446	1.111360269	-2.1206697	7.41E-05
ENSG00000138674	SEC31A	2.714112384	11.84546256	-2.120983784	9.12E-07
ENSG00000229847	EMX2OS	1.131768215	4.940988303	-2.12106414	1.33E-06
ENSG00000109667	SLC2A9	0.208485013	0.913671222	-2.122832774	2.77E-05
ENSG00000141068	KSR1	0.236754484	1.036557759	-2.123701442	5.52E-06
ENSG00000108924	HLF	0.278485282	1.220442586	-2.123916255	1.08E-05
ENSG00000157483	MYO1E	3.202969979	14.0296453	-2.126233717	8.16E-07
ENSG00000166359	WDR88	0.408548677	1.799278123	-2.126325789	0.000132
ENSG00000228925	AC016722.2	0.136628177	0.609851798	-2.129893043	0.006128
ENSG00000224738	AC099850.1	0.187378703	0.836381203	-2.129893043	0.006128
ENSG00000198569	SLC34A3	0.108863528	0.485921862	-2.129893043	0.006128
ENSG00000158457	TSPAN33	2.537516464	11.16636424	-2.132252855	1.47E-06
ENSG00000166510	CCDC68	0.274267437	1.209814107	-2.132350737	2.41E-05
ENSG00000075643	MOCOS	0.399160491	1.760307455	-2.13383469	6.02E-06
ENSG00000197956	S100A6	79.61182688	350.7750721	-2.134856869	6.32E-07
ENSG00000169330	KIAA1024	0.135188223	0.599035897	-2.136589976	6.51E-05
ENSG00000255471	AP001528.2	0.171533049	0.771777965	-2.136645554	0.009733
ENSG00000180815	MAP3K15	0.03748919	0.168674962	-2.136645554	0.009733
ENSG00000217801	AL390719.1	0.904613628	4.000021032	-2.13701832	8.78E-06
ENSG00000120915	EPHX2	2.092542135	9.246842095	-2.138504637	1.17E-06
ENSG00000115423	DNAH6	0.037104145	0.165617589	-2.141692347	0.000339
ENSG00000173156	RHOD	21.96626463	97.4172923	-2.144114691	6.74E-07
ENSG00000103355	PRSS33	0.356126001	1.589599501	-2.145659738	0.000114
ENSG00000182957	SPATA13	1.020694063	4.544707799	-2.149486499	9.75E-07
ENSG00000243926	TIPARP-AS1	0.333217311	1.499243319	-2.150782703	0.001209
ENSG00000144455	SUMF1	2.458321254	10.95898066	-2.15145847	7.38E-07
ENSG00000262001	DLGAP1-AS2	1.423156317	6.366498898	-2.155512264	1.79E-06
ENSG00000238098	ABCA17P	0.057205515	0.25874637	-2.156790005	0.001609
ENSG00000198108	CHSY3	0.732147309	3.285053897	-2.159060797	3.90E-06
ENSG00000241935	HOGA1	0.634639199	2.848954725	-2.159574314	4.23E-06
ENSG00000226007	BX005266.2	0.257254314	1.161400262	-2.159751263	0.000197
ENSG00000196935	SRGAP1	0.710514215	3.187404465	-2.160570591	6.31E-07

ENSG00000246089	AC016065.1	0.783299179	3.524294878	-2.162514694	5.32E-06
ENSG00000230454	U73166.1	0.127346136	0.579789008	-2.164240447	0.002205
ENSG00000178623	GPR35	0.853937292	3.843875869	-2.16441203	1.71E-06
ENSG00000244274	DBNDD2	0.196530702	0.890727825	-2.164583991	0.000233
ENSG00000158023	WDR66	0.098532245	0.44443683	-2.164950119	1.34E-05
ENSG00000013588	GPRC5A	1.292180409	5.823448805	-2.166954763	7.50E-07
ENSG00000188643	S100A16	55.09980367	248.2366428	-2.166975531	4.35E-07
ENSG00000147852	VLDLR	3.163327303	14.28834635	-2.170562372	5.00E-07
ENSG00000269190	FBXO17	3.108635349	14.0606742	-2.172362815	6.05E-07
ENSG00000283959	AP002851.1	0.137323078	0.630466506	-2.173725198	0.003128
ENSG00000158815	FGF17	0.088986357	0.4085469	-2.173725198	0.003128
ENSG00000176472	ZNF575	0.09078155	0.416788845	-2.173725198	0.003128
ENSG00000118557	PMFBP1	0.080766115	0.369683137	-2.176782655	0.000759
ENSG00000114529	C3orf52	0.63847558	2.899454836	-2.176945431	1.89E-06
ENSG00000169379	ARL13B	3.289321714	14.96773626	-2.180993174	5.73E-07
ENSG00000213204	AL049697.1	0.118372382	0.545273157	-2.184633733	0.000974
ENSG00000155749	ALS2CR12	0.158578561	0.730479789	-2.184633733	0.000974
ENSG00000121769	FABP3	3.217398255	14.70289678	-2.185738772	2.04E-06
ENSG00000282034	AC106886.5	0.021443098	0.099541667	-2.186209504	0.004621
ENSG00000197142	ACSL5	0.059186191	0.27475004	-2.186209504	0.004621
ENSG00000273143	AL355512.1	0.399224052	1.853250272	-2.186209504	0.004621
ENSG00000146966	DENND2A	0.048700992	0.226076374	-2.186209504	0.004621
ENSG00000170190	SLC16A5	4.842729483	22.14245722	-2.187960048	5.07E-07
ENSG00000274012	RN7SL2	178.8417292	819.1651319	-2.190800695	3.47E-07
ENSG00000272240	AC004908.1	0.695136889	3.226916371	-2.194147388	0.00128
ENSG00000138639	ARHGAP24	0.285333124	1.313232713	-2.195153042	3.72E-06
ENSG00000104327	CALB1	0.152202538	0.702363359	-2.196078693	2.39E-05
ENSG00000036672	USP2	0.174950102	0.808853239	-2.196733156	5.68E-05
ENSG00000185033	SEMA4B	16.47918933	75.85323778	-2.197955843	3.03E-07
ENSG00000144681	STAC	1.501842862	6.924231787	-2.199441332	8.09E-07
ENSG00000161642	ZNF385A	0.722931674	3.337168891	-2.199489796	3.49E-06
ENSG00000243224	AC006252.1	0.56928407	2.642691124	-2.200547104	0.00012
ENSG00000107679	PLEKHA1	2.832616649	13.06353097	-2.200691068	3.03E-07
ENSG00000072657	TRHDE	1.877722766	8.66419256	-2.201252904	3.75E-07
ENSG00000225329	LHFPL3-AS2	0.721348834	3.336030563	-2.201255935	5.77E-06
ENSG00000107338	SHB	2.94284885	13.59138989	-2.202107137	6.05E-07
ENSG00000143036	SLC44A3	1.339930802	6.194130156	-2.202602431	1.46E-06
ENSG00000150281	CTF1	0.853789258	3.954434452	-2.202680789	1.17E-05
ENSG00000279822	AC016397.2	0.105253025	0.496115206	-2.203384336	0.007168
ENSG00000276075	AC027682.6	0.236610706	1.115275972	-2.203384336	0.007168
ENSG00000109927	TECTA	0.024922731	0.117474494	-2.203384336	0.007168
ENSG00000124243	BCAS4	0.451853747	2.095596505	-2.207097834	1.54E-06
ENSG00000050165	DKK3	4.493236848	20.81985095	-2.207307575	3.52E-07

ENSG00000182648	LINC01006	0.274072079	1.276042579	-2.208888966	2.13E-05
ENSG00000174827	PDZK1	1.280521878	5.956383164	-2.211227503	1.62E-06
ENSG00000161011	SQSTM1	32.94685071	153.54207	-2.215840381	2.40E-07
ENSG00000260563	AC132872.1	0.454569757	2.13514473	-2.216003261	0.00016
ENSG00000215271	HOMEZ	1.238311154	5.77536485	-2.216090286	5.92E-07
ENSG00000162989	KCNJ3	0.082465859	0.388706802	-2.217708666	0.000786
ENSG00000163297	ANTXR2	0.182154743	0.853149526	-2.219679515	4.51E-06
ENSG00000139174	PRICKLE1	1.869629117	8.744196614	-2.220620393	3.55E-07
ENSG00000257176	AC009318.1	0.103980859	0.49330138	-2.220844108	0.00241
ENSG00000243176	AC092944.1	0.18944181	0.898741438	-2.220844108	0.00241
ENSG00000183722	LHFPL6	0.064088115	0.304043992	-2.220844108	0.00241
ENSG00000229320	KRT8P12	1.020650971	4.789019887	-2.22275977	3.32E-06
ENSG00000242574	HLA-DMB	0.34110572	1.605895031	-2.226680657	7.55E-06
ENSG00000196196	HRCT1	0.747739645	3.533930681	-2.227517677	6.90E-05
ENSG00000125753	VASP	12.62498484	59.39411184	-2.22934665	2.28E-07
ENSG00000164442	CITED2	7.862587776	37.13485029	-2.234833222	2.67E-07
ENSG00000249395	CASC9	1.07796818	5.105366252	-2.235287575	6.96E-06
ENSG00000225434	LINC01504	0.849988827	4.024674513	-2.235868066	2.92E-06
ENSG00000250510	GPR162	0.29167386	1.385686932	-2.237249895	2.50E-05
ENSG00000271966	AC021321.1	0.225258495	1.085897837	-2.24040996	0.00349
ENSG00000162148	PPP1R32	0.104463627	0.503585122	-2.24040996	0.00349
ENSG00000039560	RAI14	6.338211992	30.1410068	-2.244896825	1.89E-07
ENSG00000146250	PRSS35	0.137001478	0.660439504	-2.246418594	0.001356
ENSG00000117318	ID3	9.208146077	43.9660046	-2.250477427	2.42E-07
ENSG00000257702	LBX2-AS1	0.461163804	2.212380788	-2.251308498	2.22E-05
ENSG00000011028	MRC2	0.25582929	1.22674445	-2.253524027	3.37E-06
ENSG00000172602	RND1	0.147131869	0.70927482	-2.254200812	9.69E-05
ENSG00000012171	SEMA3B	3.535246181	16.94138267	-2.255810381	2.10E-07
ENSG00000157429	ZNF19	0.081532587	0.393042046	-2.256519054	4.01E-05
ENSG00000095397	WHRN	0.557943586	2.681587241	-2.258349562	1.02E-06
ENSG00000137393	RNF144B	2.7937712	13.41596177	-2.25868201	2.41E-07
ENSG00000104419	NDRG1	2.875594494	13.80762084	-2.258757734	1.85E-07
ENSG00000076067	RBMS2	4.625433071	22.21893383	-2.259421182	1.63E-07
ENSG00000237489	C10orf143	0.09225519	0.448026537	-2.265523965	7.26E-05
ENSG00000274419	TBC1D3D	0.296210662	1.438512426	-2.265523965	7.26E-05
ENSG00000262877	AC110285.2	0.133499843	0.657860578	-2.267171819	0.005286
ENSG00000138356	AOX1	0.037603897	0.185304501	-2.267171819	0.005286
ENSG00000100504	PYGL	12.87179996	62.25600518	-2.269342389	1.39E-07
ENSG00000107104	KANK1	1.466771785	7.097142615	-2.269683805	1.88E-07
ENSG00000230487	PSMG3-AS1	0.170955424	0.831164184	-2.271287719	1.23E-05
ENSG00000071282	LMCD1	0.663624355	3.226082612	-2.275768803	3.57E-07
ENSG00000089723	OTUB2	1.513395912	7.369281602	-2.278217794	3.40E-07
ENSG00000274712	AC005332.4	0.4376531	2.143271675	-2.280371589	2.18E-05

ENSG00000104901	DKKL1	0.286005823	1.40937961	-2.281667116	0.000513
ENSG00000230510	PPP5D1	0.073269246	0.361056218	-2.281667116	0.000513
ENSG00000248383	PCDHAC1	0.114348778	0.560909061	-2.282002579	2.80E-05
ENSG00000223396	RPS10P7	0.332289867	1.63152532	-2.28499508	1.48E-05
ENSG00000240859	AC093627.4	0.121117249	0.596841626	-2.286548473	6.15E-05
ENSG00000257557	PPP1R12A-AS1	0.193810068	0.955057333	-2.286548473	6.15E-05
ENSG00000162976	PQLC3	1.744886955	8.559102441	-2.288210649	5.65E-07
ENSG00000184481	FOXO4	1.151615266	5.66194406	-2.291522464	5.46E-07
ENSG00000119125	GDA	10.87457897	53.43937485	-2.292314595	1.03E-07
ENSG00000075826	SEC31B	0.284254767	1.404134211	-2.297122677	1.35E-06
ENSG00000272031	ANKRD34A	0.159096087	0.791298049	-2.299203677	6.83E-05
ENSG00000172927	MYEOV	7.434698552	36.71528408	-2.299255851	1.16E-07
ENSG00000109099	PMP22	0.62250206	3.084237454	-2.301116703	1.61E-06
ENSG00000275395	FCGBP	0.206331984	1.02228872	-2.301888191	9.46E-07
ENSG00000120896	SORBS3	1.679093888	8.310948673	-2.302167373	1.74E-07
ENSG00000176927	EFCAB5	0.102533583	0.510433983	-2.30236217	3.74E-05
ENSG00000171798	KNDC1	0.088960885	0.442866203	-2.30236217	3.74E-05
ENSG00000143333	RGS16	0.568158169	2.821902621	-2.30325315	4.85E-06
ENSG00000235052	AL021154.1	0.366539042	1.865130082	-2.306002759	0.008459
ENSG00000227755	AP000344.1	0.210241262	1.069810462	-2.306002759	0.008459
ENSG00000174483	BBS1	0.027199642	0.138405096	-2.306002759	0.008459
ENSG00000122735	DNAI1	0.04623563	0.235269521	-2.306002759	0.008459
ENSG00000249307	LINC01088	0.041837748	0.212890943	-2.306002759	0.008459
ENSG00000213904	LIPE-AS1	0.03972001	0.202114857	-2.306002759	0.008459
ENSG00000002079	MYH16	0.026500999	0.134850058	-2.306002759	0.008459
ENSG00000285219	AL591485.1	0.015542292	0.0776993	-2.308909683	2.59E-05
ENSG00000125257	ABCC4	3.56640153	17.7549152	-2.310918718	9.92E-08
ENSG00000261068	AL512274.1	0.206144306	1.037921427	-2.312613547	0.000416
ENSG00000111145	ELK3	4.962471777	24.79884661	-2.316315642	1.01E-07
ENSG00000166016	ABTB2	1.487236031	7.453019301	-2.319793157	1.83E-07
ENSG00000188064	WNT7B	1.058906691	5.30847556	-2.320001977	2.64E-07
ENSG00000138119	MYOF	7.002872663	35.0846091	-2.320157702	7.68E-08
ENSG00000245937	LINC01184	1.279938003	6.426388796	-2.322624959	1.55E-07
ENSG00000246465	AC138904.1	0.134737447	0.68561009	-2.324166941	0.000838
ENSG00000135437	RDH5	0.112062892	0.570230853	-2.324166941	0.000838
ENSG00000114626	ABTB1	1.148437336	5.77283391	-2.324168889	1.77E-07
ENSG00000242950	ERVW-1	0.034082749	0.17525529	-2.328258009	0.003905
ENSG00000226608	FTLP3	0.395695556	2.034687362	-2.328258009	0.003905
ENSG00000196611	MMP1	0.106054444	0.545337526	-2.328258009	0.003905
ENSG00000170214	ADRA1B	0.248815486	1.266095002	-2.333379344	3.46E-05
ENSG00000272092	AC087623.2	0.342503695	1.753018437	-2.334595129	0.000519
ENSG00000006468	ETV1	1.147187916	5.809523316	-2.335195535	1.12E-07
ENSG00000277639	AC007906.2	1.496100355	7.58467422	-2.335221386	5.74E-07

ENSG00000242265	PEG10	5.853336427	29.65916528	-2.336430547	6.65E-08
ENSG00000246090	AP002026.1	0.077369443	0.395287503	-2.337063289	6.37E-05
ENSG00000037042	TUBG2	1.190855198	6.055617148	-2.339828348	3.95E-07
ENSG00000184005	ST6GALNAC3	0.196311362	1.00202097	-2.342748672	3.12E-06
ENSG00000175600	SUGCT	0.438769171	2.240506279	-2.342757716	4.11E-06
ENSG00000153292	ADGRF1	0.047173318	0.241845752	-2.342845101	4.83E-05
ENSG00000170927	PKHD1	0.045486626	0.233381695	-2.34676572	1.61E-05
ENSG00000148498	PARD3	7.198414824	36.74933412	-2.347276656	5.71E-08
ENSG00000272853	AC069544.1	0.460100058	2.374820284	-2.349695162	0.000187
ENSG00000230650	AC140479.2	0.146849827	0.757969797	-2.349695162	0.000187
ENSG00000125864	BFSP1	0.745946756	3.822379055	-2.350276013	6.49E-07
ENSG00000189060	H1F0	60.61029547	310.1144785	-2.350565134	5.07E-08
ENSG00000210082	MT-RNR2	4421.216962	22693.12265	-2.355181866	4.61E-08
ENSG00000057252	SOAT1	4.861547804	24.96638365	-2.355758446	5.67E-08
ENSG00000049769	PPP1R3F	0.220292336	1.137045514	-2.356444898	9.81E-06
ENSG00000145431	PDGFC	4.699275617	24.14631485	-2.35649753	5.98E-08
ENSG00000204104	TRAF3IP1	1.257998193	6.476449095	-2.358437975	1.45E-07
ENSG00000246898	LINC00920	0.155698	0.813116794	-2.361524325	0.00066
ENSG00000008517	IL32	0.247405287	1.28333039	-2.36251223	1.40E-05
ENSG00000196159	FAT4	0.393986548	2.034068502	-2.362625373	1.29E-07
ENSG00000178075	GRAMD1C	0.566836163	2.933032819	-2.365417124	1.83E-07
ENSG00000236404	VLDLR-AS1	0.23024209	1.195875691	-2.367743567	2.61E-06
ENSG00000221949	LINC01465	0.344622274	1.799754388	-2.370795297	2.53E-05
ENSG00000253948	AC104986.2	1.099617125	5.772086779	-2.372583451	0.000273
ENSG00000077616	NAALAD2	0.057603323	0.302370137	-2.372583451	0.000273
ENSG00000175482	POLD4	0.438548516	2.290274525	-2.375464059	2.60E-06
ENSG00000158286	RNF207	0.387078587	2.018928963	-2.375871793	4.62E-07
ENSG00000111254	AKAP3	0.205054011	1.073924409	-2.37592963	1.46E-05
ENSG00000174137	FAM53A	0.108431224	0.570230853	-2.376601404	0.000154
ENSG00000233639	PANTR1	6.238112317	32.56464086	-2.37837452	1.39E-07
ENSG00000071246	VASH1	0.526977386	2.753806334	-2.379223554	2.42E-07
ENSG00000250917	AL035458.2	0.349669044	1.872933973	-2.379547179	0.006073
ENSG00000214146	LINC02026	0.071581072	0.383410038	-2.379547179	0.006073
ENSG00000164620	RELL2	0.193153085	1.017343681	-2.379948273	5.88E-05
ENSG00000088367	EPB41L1	2.863733084	14.99661756	-2.383884188	4.26E-08
ENSG00000013016	EHD3	0.216318123	1.1393547	-2.386171031	5.26E-06
ENSG00000272341	AL137003.2	0.082319643	0.440929097	-2.386862431	0.00289
ENSG00000110169	HPX	0.057539866	0.30820106	-2.386862431	0.00289
ENSG00000227110	LMCD1-AS1	0.032351696	0.173285545	-2.386862431	0.00289
ENSG00000156345	CDK20	0.539920265	2.837264409	-2.38709102	2.50E-07
ENSG00000227398	KIF9-AS1	0.40096288	2.120262208	-2.394939298	6.40E-07
ENSG00000235618	FAM21EP	0.114750159	0.614636826	-2.395290159	0.000861
ENSG00000146072	TNFRSF21	2.836063974	14.97914763	-2.395818453	5.91E-08

ENSG00000160963	COL26A1	4.567801176	24.14031482	-2.396878501	4.79E-08
ENSG00000181652	ATG9B	0.058843539	0.315183929	-2.400003689	0.000333
ENSG00000107821	KAZALD1	0.948355096	5.030121095	-2.40069684	1.92E-07
ENSG00000131378	RFTN1	0.262471424	1.396504433	-2.402005825	2.33E-06
ENSG00000186765	FSCN2	0.227657235	1.219401539	-2.403015019	0.000127
ENSG00000285867	BX470102.2	0.447325354	2.381916754	-2.403733543	1.72E-06
ENSG00000197813	AC011450.1	0.951213513	5.094989492	-2.405927557	2.89E-05
ENSG00000139344	AMDHD1	0.217794606	1.166574288	-2.405927557	2.89E-05
ENSG00000099860	GADD45B	0.859943795	4.585184148	-2.406677765	6.30E-07
ENSG00000144959	NCEH1	7.821735791	41.6628475	-2.408475845	3.00E-08
ENSG00000090932	DLL3	1.228983846	6.56571384	-2.410966236	1.80E-07
ENSG00000166949	SMAD3	1.742397815	9.313165723	-2.413242594	3.79E-08
ENSG00000144802	NFKBIZ	0.913304498	4.891937253	-2.415508385	8.95E-08
ENSG00000136826	KLF4	0.628095051	3.388735934	-2.424382491	3.33E-07
ENSG00000164574	GALNT10	3.666577758	19.75019224	-2.42463917	2.45E-08
ENSG00000143344	RGL1	0.133902481	0.725660413	-2.424666734	1.14E-05
ENSG00000179715	PCED1B	0.255373267	1.384955263	-2.425160387	1.59E-05
ENSG00000148180	GSN	1.300817717	7.032579789	-2.429636111	3.27E-08
ENSG00000182013	PNMA8A	0.768779938	4.166264598	-2.431325407	2.16E-07
ENSG00000128564	VGF	0.139078793	0.761502814	-2.431628513	0.000267
ENSG00000283491	AC017104.5	0.523135534	2.872125196	-2.433456611	0.000412
ENSG00000242114	MTFP1	0.197101183	1.082127359	-2.433456611	0.000412
ENSG00000284707	AC079781.5	0.068102015	0.375197297	-2.435794187	0.000668
ENSG00000205542	TMSB4X	118.2736571	643.3287628	-2.438839006	1.73E-08
ENSG00000059378	PARP12	0.458742419	2.499897503	-2.439355771	1.95E-07
ENSG00000135631	RAB11FIP5	1.574798796	8.587314698	-2.441911386	3.17E-08
ENSG00000280149	AC004877.2	0.348793412	1.942973574	-2.443178893	0.002142
ENSG00000106113	CRHR2	0.05244158	0.292128808	-2.443178893	0.002142
ENSG00000050344	NFE2L3	2.753571714	15.04394873	-2.444707364	3.07E-08
ENSG00000165695	AK8	0.214205899	1.180133571	-2.446500446	2.06E-05
ENSG00000139985	ADAM21	0.062882544	0.353658977	-2.449523664	0.004369
ENSG00000258458	AL160314.2	0.066510865	0.374065086	-2.449523664	0.004369
ENSG00000123329	ARHGAP9	0.032600313	0.183348071	-2.449523664	0.004369
ENSG00000248099	INSL3	0.181085377	1.018445895	-2.449523664	0.004369
ENSG00000091536	MYO15A	0.008356255	0.046996578	-2.449523664	0.004369
ENSG00000165046	LETM2	0.205031652	1.127497675	-2.44958587	1.47E-06
ENSG00000101670	LIPG	0.85406504	4.687291523	-2.451211958	2.82E-08
ENSG00000215769	ARHGAP27P1-BPTFP1-KPNA2P3	0.165051155	0.913533101	-2.451358777	3.34E-05
ENSG00000176014	TUBB6	7.358380325	40.38140271	-2.451464024	1.85E-08
ENSG00000176681	LRRC37A	0.128452047	0.708669161	-2.451734927	5.44E-06
ENSG00000102904	TSNAXIP1	0.119325015	0.662382646	-2.454434289	8.61E-05

ENSG00000141337	ARSG	0.399648307	2.201799228	-2.454907902	1.83E-07
ENSG00000132535	DLG4	0.433366602	2.395002745	-2.460386771	6.19E-08
ENSG00000068650	ATP11A	0.934110055	5.163095113	-2.461518341	2.34E-08
ENSG00000232860	SMG7-AS1	0.185273947	1.032079076	-2.463135048	1.42E-05
ENSG00000105997	HOXA3	1.203296771	6.663530232	-2.463508053	5.27E-08
ENSG00000236333	TRHDE-AS1	1.037379372	5.745135913	-2.464141124	2.92E-08
ENSG00000006062	MAP3K14	1.863873841	10.32399805	-2.464477723	2.44E-08
ENSG00000170485	NPAS2	1.512050576	8.374943241	-2.464678704	1.84E-08
ENSG00000267519	AC020916.1	0.144776091	0.803663589	-2.465902011	1.54E-07
ENSG00000178764	ZHX2	1.816373496	10.08024502	-2.467096923	2.86E-08
ENSG00000137267	TUBB2A	1.137183663	6.324378552	-2.467658685	2.81E-07
ENSG00000133104	SPART	0.937988286	5.209975911	-2.468033181	3.95E-08
ENSG00000205309	NT5M	1.85381653	10.31765993	-2.470235028	7.97E-08
ENSG00000163485	ADORA1	1.964145502	10.92643134	-2.47051697	2.76E-08
ENSG00000145623	OSMR	2.0560725	11.43724597	-2.470787336	1.89E-08
ENSG00000229155	LINC02038	0.764368002	4.276151895	-2.47096052	6.32E-06
ENSG00000188158	NHS	0.406035263	2.260981242	-2.471204396	6.74E-08
ENSG00000119943	PYROXD2	1.296678069	7.269521746	-2.481209586	4.35E-08
ENSG00000173068	BNC2	0.076355323	0.429431504	-2.481619735	1.27E-06
ENSG00000109339	MAPK10	0.018616601	0.104964409	-2.482654988	4.79E-06
ENSG00000134215	VAV3	1.218335244	6.852063642	-2.486348852	2.24E-08
ENSG00000227097	RPS28P7	326.7224293	1840.616943	-2.489401852	9.84E-09
ENSG00000284693	AL928921.2	0.207095257	1.177527798	-2.491106568	1.83E-05
ENSG00000121236	TRIM6	0.137626263	0.782532408	-2.491106568	1.83E-05
ENSG00000167767	KRT80	1.508118568	8.520428732	-2.492644011	2.85E-08
ENSG00000171033	PKIA	0.961382525	5.446746102	-2.496076262	5.22E-08
ENSG00000115525	ST3GAL5	0.092185086	0.523946335	-2.498005201	5.39E-07
ENSG00000185950	IRS2	0.287538123	1.633650433	-2.498982186	1.39E-07
ENSG00000122786	CALD1	1.710813437	9.72300668	-2.501782616	1.26E-08
ENSG00000255495	AC145124.1	0.337722232	1.940502715	-2.504083813	5.88E-05
ENSG00000002587	HS3ST1	0.138159095	0.789357037	-2.504119291	1.12E-06
ENSG00000171729	TMEM51	3.059291931	17.44562744	-2.506103381	2.12E-08
ENSG00000117519	CNN3	9.864782244	56.27724424	-2.50740656	9.60E-09
ENSG00000153956	CACNA2D1	0.593622386	3.403189627	-2.513513412	2.81E-08
ENSG00000275180	AC048341.2	1.531849519	8.843223687	-2.516227196	4.19E-06
ENSG00000131969	ABHD12B	0.037610667	0.221599614	-2.516262446	0.003149
ENSG00000271870	AC024060.1	0.338343731	1.993499359	-2.516262446	0.003149
ENSG00000277782	AC068870.2	0.429670445	2.531590445	-2.516262446	0.003149
ENSG00000110427	KIAA1549L	0.012845205	0.075683114	-2.516262446	0.003149
ENSG00000135477	KRT87P	0.094005514	0.5538744	-2.516262446	0.003149
ENSG00000214837	LINC01347	0.043515179	0.256388618	-2.516262446	0.003149
ENSG00000135862	LAMC1	57.23006122	329.051015	-2.518895932	6.36E-09
ENSG00000126882	FAM78A	0.129178517	0.748901113	-2.521831847	4.80E-06

ENSG00000279989	AC011815.3	0.795072002	4.637194453	-2.522544847	0.000139
ENSG00000171502	COL24A1	0.096078587	0.556731973	-2.523162646	1.85E-06
ENSG00000282936	AC004706.3	0.165393359	0.961831289	-2.524375669	1.06E-05
ENSG00000169247	SH3TC2	0.468415867	2.709696404	-2.52680721	1.64E-08
ENSG00000272449	AL139246.5	0.436686618	2.54369145	-2.528105591	5.55E-06
ENSG00000207721	MIR186	2.915264006	17.1765468	-2.528746813	0.00067
ENSG00000124067	SLC12A4	0.946693751	5.486152692	-2.529449247	1.51E-08
ENSG00000261438	AL157394.1	0.273107521	1.609131835	-2.535046998	0.000205
ENSG00000150687	PRSS23	0.623663444	3.63068051	-2.53596291	1.45E-08
ENSG00000174939	ASPHD1	4.319516752	25.1695755	-2.537537185	1.09E-08
ENSG00000105875	WDR91	2.604663376	15.17582633	-2.537639315	8.43E-09
ENSG00000117308	GALE	7.426423752	43.2777695	-2.5381116	6.52E-09
ENSG00000234494	SP2-AS1	0.577831204	3.37801513	-2.538499898	3.79E-07
ENSG00000174600	CMKLR1	0.073436645	0.432683956	-2.538845912	6.54E-05
ENSG00000186564	FOXD2	0.089380643	0.526624964	-2.538845912	6.54E-05
ENSG00000150938	CRIM1	2.792737231	16.3163528	-2.541685878	7.01E-09
ENSG00000167107	ACSF2	0.937055132	5.490181079	-2.544904225	1.95E-08
ENSG00000054598	FOXC1	2.181868927	12.80412276	-2.547662165	1.08E-08
ENSG00000088992	TESC	12.84371072	75.39541414	-2.548596946	6.09E-09
ENSG00000164078	MST1R	1.20137149	7.057095389	-2.54881292	1.49E-08
ENSG00000087903	RFX2	0.192983168	1.137044334	-2.55051162	1.51E-07
ENSG00000237523	LINC00857	0.885366529	5.216522274	-2.55083049	1.20E-07
ENSG00000231856	AL162377.1	0.118468269	0.7070724	-2.550899918	0.000315
ENSG00000147234	FRMPD3	0.031939087	0.190626966	-2.550899918	0.000315
ENSG00000239445	ST3GAL6-AS1	0.346972901	2.070891763	-2.550899918	0.000315
ENSG00000130475	FCHO1	1.005375264	5.919675244	-2.552099715	1.50E-08
ENSG00000173334	TRIB1	2.241752071	13.22989322	-2.555849709	9.50E-09
ENSG00000051128	HOMER3	1.343190995	7.966318315	-2.562285855	1.81E-08
ENSG00000049239	H6PD	0.919001975	5.465125286	-2.566802748	8.52E-09
ENSG00000168071	CCDC88B	0.16551673	0.988646554	-2.56923534	3.18E-07
ENSG00000185015	CA13	0.28723718	1.716675444	-2.570675271	2.08E-07
ENSG00000275342	PRAG1	0.349348181	2.090284447	-2.572630548	1.24E-07
ENSG00000146648	EGFR	1.962290955	11.71899319	-2.573417304	4.47E-09
ENSG00000221968	FADS3	3.099159646	18.50940488	-2.573420098	4.90E-09
ENSG00000165655	ZNF503	0.524346857	3.14291415	-2.575266679	1.08E-07
ENSG00000081052	COL4A4	0.352497352	2.114653241	-2.578356639	2.31E-08
ENSG00000261118	AC092123.1	0.159944309	0.985217038	-2.580049929	0.002276
ENSG00000230102	LINC02028	0.022752764	0.140151348	-2.580049929	0.002276
ENSG00000171951	SCG2	0.061471792	0.378650903	-2.580049929	0.002276
ENSG00000189120	SP6	0.041016393	0.25265075	-2.580049929	0.002276
ENSG00000233384	AC096537.1	0.549808563	3.337601199	-2.580121093	7.33E-05
ENSG00000232104	RFX3-AS1	0.24076124	1.461535992	-2.580121093	7.33E-05
ENSG00000172086	KRCC1	4.9818364	30.00380394	-2.585096467	6.80E-09

ENSG00000177406	AC021054.1	0.177558573	1.077865543	-2.586939888	4.09E-06
ENSG00000123352	SPATS2	3.842550954	23.23232207	-2.591193179	3.44E-09
ENSG00000181019	NQO1	113.9019509	690.265499	-2.594784468	2.47E-09
ENSG00000173295	FAM86B3P	0.56629153	3.437660701	-2.594900335	3.52E-08
ENSG00000222020	AC062017.1	0.340827494	2.117668086	-2.60002925	0.000884
ENSG00000280213	UCKL1-AS1	0.058003124	0.360391598	-2.60002925	0.000884
ENSG00000162545	CAMK2N1	3.946847548	24.03343145	-2.601083364	4.94E-09
ENSG00000180769	WDFY3-AS2	0.357025454	2.17934271	-2.602304733	4.83E-08
ENSG00000163995	ABLIM2	0.103617418	0.636407421	-2.60378988	3.51E-06
ENSG00000078114	NEBL	0.058145859	0.357344389	-2.606839276	1.50E-06
ENSG00000230513	THAP7-AS1	0.266865064	1.640060603	-2.606839276	1.50E-06
ENSG00000103723	AP3B2	0.041289971	0.255564596	-2.608069855	5.93E-05
ENSG00000158104	HPD	0.169171865	1.047090572	-2.608069855	5.93E-05
ENSG00000256043	CTSO	0.579960556	3.561047279	-2.60992427	7.76E-08
ENSG00000116791	CRYZ	11.01662925	67.47889964	-2.610052042	2.28E-09
ENSG00000141756	FKBP10	12.0183288	73.6658726	-2.611076445	2.22E-09
ENSG00000155754	C2CD6	0.036788365	0.229891309	-2.613334761	0.000392
ENSG00000165801	ARHGEF40	0.637828959	3.930233812	-2.617569983	8.26E-09
ENSG00000104998	IL27RA	0.648930025	4.004803281	-2.617654262	5.44E-08
ENSG00000177606	JUN	5.984526422	36.86002839	-2.617883771	2.65E-09
ENSG00000243232	PCDHAC2	0.291003456	1.799593697	-2.620465705	6.07E-08
ENSG00000183889	PKD1P1	0.058204376	0.362775713	-2.621210263	2.31E-05
ENSG00000273117	AC144652.1	0.180109702	1.130103449	-2.622832204	0.000193
ENSG00000070087	PFN2	14.42043057	89.16418039	-2.623671189	1.88E-09
ENSG00000166963	MAP1A	0.117938049	0.732786078	-2.62561274	2.26E-07
ENSG00000235162	C12orf75	28.70519269	177.955434	-2.627491028	1.73E-09
ENSG00000233184	AC093157.1	0.529674728	3.294187496	-2.627856906	1.28E-07
ENSG00000181513	ACBD4	1.029143782	6.389651149	-2.628057867	1.15E-08
ENSG00000260498	AC126696.3	0.156304679	1.004658598	-2.62858614	0.004849
ENSG00000204936	CD177	0.050122492	0.322165612	-2.62858614	0.004849
ENSG00000123364	HOXC13	0.05173601	0.332536606	-2.62858614	0.004849
ENSG00000257108	NHLRC4	0.059551711	0.382772539	-2.62858614	0.004849
ENSG00000264745	TTC39C-AS1	0.153434948	0.986213213	-2.62858614	0.004849
ENSG00000261341	AC010325.1	0.40178318	2.528685976	-2.62995188	0.000103
ENSG00000140297	GCNT3	0.516268117	3.214648913	-2.63196055	1.36E-08
ENSG00000064393	HIPK2	6.098798423	38.02046237	-2.63555269	1.54E-09
ENSG00000143878	RHOB	12.4898333	77.90141967	-2.636118041	1.92E-09
ENSG00000197405	C5AR1	0.212396395	1.334854196	-2.636910715	2.60E-06
ENSG00000167191	GPRC5B	1.10934825	6.92898483	-2.63749182	4.16E-09
ENSG00000261575	AC005829.1	0.247984871	1.593939061	-2.641136119	0.001649
ENSG00000140416	TPM1	3.560006173	22.30855496	-2.642945891	1.50E-09
ENSG00000271888	AL136162.1	0.193810068	1.245726956	-2.648739189	0.000661
ENSG00000226435	ANKRD18DP	0.060576183	0.389357396	-2.648739189	0.000661

ENSG00000242808	SOX2-OT	0.00660556	0.04245767	-2.648739189	0.000661
ENSG00000204525	HLA-C	0.100005068	0.642789147	-2.65383879	0.000301
ENSG00000175573	C11orf68	11.38956068	71.96161174	-2.654632095	1.71E-09
ENSG00000106688	SLC1A1	1.222835853	7.736697668	-2.655591541	5.51E-09
ENSG00000110881	ASIC1	0.741882677	4.706166347	-2.659196277	6.03E-09
ENSG00000272269	AL138724.1	0.307245961	1.974843616	-2.660248413	8.25E-05
ENSG00000136114	THSD1	0.101503119	0.652417972	-2.662393693	3.91E-05
ENSG00000170915	PAQR8	1.041124539	6.629934417	-2.665050392	4.59E-09
ENSG00000108785	HSD17B1P1	0.481298386	3.093577015	-2.665521952	7.80E-06
ENSG00000171877	FRMD5	0.10645177	0.684225752	-2.670509599	1.20E-06
ENSG00000067057	PFKP	14.49875649	92.70113036	-2.672007252	9.88E-10
ENSG00000100994	PYGB	15.43838849	98.80103473	-2.673352043	9.72E-10
ENSG00000125037	EMC3	2.828101937	18.11491212	-2.674171439	1.72E-09
ENSG00000089091	DZANK1	0.498723464	3.198815031	-2.674681598	8.36E-09
ENSG00000151883	PARP8	0.486167237	3.120420495	-2.676316675	4.31E-09
ENSG00000106804	C5	0.754734913	4.844256425	-2.676345481	4.29E-09
ENSG00000153714	LURAP1L	1.195549951	7.705471745	-2.681076955	1.47E-08
ENSG00000078018	MAP2	0.079529364	0.515052792	-2.683473183	3.82E-07
ENSG00000245248	USP2-AS1	0.157945845	1.02460737	-2.684300954	8.54E-07
ENSG00000276529	AP001505.1	1.414857249	9.195133405	-2.685208484	1.66E-06
ENSG00000110811	P3H3	1.444009274	9.339838531	-2.687771393	2.60E-09
ENSG00000186862	PDZD7	0.063225073	0.413156369	-2.687912263	2.01E-05
ENSG00000250303	AP002884.1	0.0666169	0.437105017	-2.689921784	6.60E-05
ENSG00000077147	TM9SF3	5.018690502	32.59658264	-2.694606902	8.61E-10
ENSG00000261079	AC009053.2	0.088341334	0.586747053	-2.6958581	0.000495
ENSG00000226944	AL031847.1	0.552717603	3.671049685	-2.6958581	0.000495
ENSG00000285103	AL451123.1	0.021310409	0.141539859	-2.6958581	0.000495
ENSG00000267787	AC027097.2	0.266049686	1.743582279	-2.69849113	9.17E-07
ENSG00000103253	HAGHL	0.916798239	5.977447343	-2.698938668	3.30E-09
ENSG00000172461	FUT9	0.048732941	0.319759892	-2.699647206	1.14E-06
ENSG00000283674	AC068587.4	0.012007313	0.080393538	-2.699740541	0.001197
ENSG00000156299	TIAM1	0.17229244	1.126849424	-2.700664926	5.12E-08
ENSG00000121281	ADCY7	0.87153895	5.69523846	-2.702686725	1.84E-09
ENSG00000237596	AL138828.1	0.019036652	0.129157072	-2.706081974	0.003385
ENSG00000162006	MSLNL	0.05320728	0.360992919	-2.706081974	0.003385
ENSG00000148942	SLC5A12	0.016616696	0.11273851	-2.706081974	0.003385
ENSG00000279227	AC009303.4	0.278569672	1.850209041	-2.711325209	1.66E-05
ENSG00000251396	LINC01301	0.09436642	0.62676458	-2.711325209	1.66E-05
ENSG00000163710	PCOLCE2	0.625955028	4.124583878	-2.712585646	1.33E-08
ENSG00000073578	SDHA	19.54440563	128.6185517	-2.713673943	5.50E-10
ENSG00000183935	HTR7P1	0.350501401	2.313759195	-2.713929257	4.72E-08
ENSG00000119915	ELOVL3	0.27430274	1.828398855	-2.714746011	2.59E-05
ENSG00000105245	NUMBL	1.801158877	11.89224621	-2.717837831	1.15E-09

ENSG00000043143	JADE2	3.270239287	21.63150563	-2.720904657	6.35E-10
ENSG00000054277	OPN3	0.146181609	0.975730054	-2.722058234	2.41E-06
ENSG00000169435	RASSF6	0.081907548	0.546714849	-2.722058234	2.41E-06
ENSG00000133460	SLC2A11	0.441825914	2.930499098	-2.72335908	3.60E-09
ENSG00000206712	RNU6-26P	2.343109388	15.8971835	-2.731587137	0.000178
ENSG00000186523	FAM86B1	0.31595804	2.11545945	-2.733672226	6.00E-08
ENSG00000134013	LOXL2	1.252025947	8.376908052	-2.73667094	1.23E-09
ENSG00000115419	GLS	12.13976411	81.25415382	-2.73808879	4.06E-10
ENSG00000140961	OSGIN1	1.304285746	8.745627296	-2.739279385	2.14E-09
ENSG00000116701	NCF2	0.128438886	0.871413235	-2.740226578	2.11E-05
ENSG00000137936	BCAR3	2.750804507	18.45028185	-2.740810023	5.86E-10
ENSG00000106025	TSPAN12	7.369524273	49.441453	-2.741254172	5.33E-10
ENSG00000243024	AC012158.1	0.097720886	0.669981245	-2.741486638	0.000372
ENSG00000262587	AC133552.2	0.185548183	1.272131352	-2.741486638	0.000372
ENSG00000169994	MYO7B	0.023741733	0.162774989	-2.741486638	0.000372
ENSG00000165757	JCAD	0.764127003	5.141996038	-2.744976744	1.09E-09
ENSG00000174428	GTF2IRD2B	0.508320893	3.425104889	-2.745484781	5.42E-09
ENSG00000272711	AC019069.1	1.347917766	9.13396037	-2.752267149	1.37E-08
ENSG00000172915	NBEA	0.276261663	1.873090717	-2.754749602	3.14E-09
ENSG00000137834	SMAD6	1.146745408	7.768071848	-2.754779489	7.29E-10
ENSG00000246777	AC044802.1	0.057754597	0.40215659	-2.756057003	0.000872
ENSG00000234690	AC106869.1	0.047055688	0.327657987	-2.756057003	0.000872
ENSG00000185519	FAM131C	0.098260907	0.684209977	-2.756057003	0.000872
ENSG00000163637	PRICKLE2	0.051002788	0.34907161	-2.761523284	3.51E-07
ENSG00000155970	MICU3	0.517101757	3.527792721	-2.762905194	6.30E-09
ENSG00000091844	RGS17	0.174568917	1.195723349	-2.76665135	3.56E-08
ENSG00000157470	FAM81A	0.329609149	2.263033416	-2.771264221	1.05E-08
ENSG00000185189	NRBP2	0.831635697	5.718116108	-2.775337331	1.77E-09
ENSG00000140876	NUDT7	0.204080346	1.421051491	-2.775446791	2.74E-05
ENSG00000224468	LAMC1-AS1	1.74469523	12.05520404	-2.776120716	2.07E-07
ENSG00000142046	TMEM91	0.149447249	1.040630518	-2.77936712	9.41E-06
ENSG00000204396	VWA7	0.114858303	0.799780904	-2.77936712	9.41E-06
ENSG00000263105	AC009171.2	0.394202366	2.815290689	-2.779626394	0.00237
ENSG00000283312	AC017104.4	0.078741427	0.562350778	-2.779626394	0.00237
ENSG00000254554	AC080023.1	0.156110028	1.114897185	-2.779626394	0.00237
ENSG00000231530	AL157932.1	0.345334304	2.466287711	-2.779626394	0.00237
ENSG00000146049	KAAG1	0.090706478	0.647802054	-2.779626394	0.00237
ENSG00000142347	MYO1F	0.014663277	0.104721305	-2.779626394	0.00237
ENSG00000223875	NBEAP3	0.083962728	0.599639946	-2.779626394	0.00237
ENSG00000182118	FAM89A	0.558535683	3.870495508	-2.783273521	3.23E-08
ENSG00000177822	AC098864.1	0.052692876	0.370942823	-2.788381042	5.82E-05
ENSG00000145777	TSLP	0.068308771	0.480874267	-2.788381042	5.82E-05
ENSG00000138166	DUSP5	4.346326044	30.14449628	-2.788883819	4.27E-10

ENSG00000161677	JOSD2	2.870334794	19.92757213	-2.789387029	1.19E-09
ENSG00000267750	RUNDC3A-AS1	0.177876248	1.242729649	-2.793096598	9.57E-08
ENSG00000240891	PLCXD2	0.433033978	3.017794605	-2.794672443	1.50E-09
ENSG00000105996	HOXA2	0.54399285	3.80614015	-2.797137637	2.77E-08
ENSG00000227992	AC108463.1	1.445029997	10.19102777	-2.800338899	1.64E-06
ENSG00000129474	AJUBA	3.717989597	26.11369282	-2.80735486	2.36E-10
ENSG00000256967	AC018653.3	0.141765736	1.025109663	-2.810257458	0.000637
ENSG00000277232	GTSE1-DT	0.11010659	0.796182011	-2.810257458	0.000637
ENSG00000027869	SH2D2A	0.066537342	0.481132282	-2.810257458	0.000637
ENSG00000132141	CCT6B	0.241032826	1.70417977	-2.810844639	6.00E-08
ENSG00000148814	LRRC27	0.215041169	1.5177002	-2.811516637	4.51E-09
ENSG00000133315	MACROD1	10.48136726	73.99093352	-2.814659751	2.14E-10
ENSG00000178343	SHISA3	8.501251017	60.02482564	-2.814856899	2.41E-10
ENSG00000135047	CTSL	6.693442873	47.29831146	-2.815989778	2.49E-10
ENSG00000260877	AP005233.2	2.695835533	19.13262471	-2.816030129	6.50E-08
ENSG00000156103	MMP16	0.110370155	0.783308449	-2.817687157	2.21E-08
ENSG00000172965	MIR4435-2HG	0.689297187	4.881757917	-2.818905886	3.55E-10
ENSG00000162746	FCRLB	0.112846511	0.81167698	-2.819327473	4.60E-05
ENSG00000115290	GRB14	2.04830641	14.55785102	-2.823634362	4.96E-10
ENSG00000223891	OSER1-DT	1.051326529	7.494648965	-2.826187107	3.18E-09
ENSG00000285872	AC007240.2	0.11166609	0.81343995	-2.828630031	0.000211
ENSG00000018408	WWTR1	2.150612169	15.33960307	-2.829515345	1.92E-10
ENSG00000167377	ZNF23	0.063094023	0.454697464	-2.830295997	1.82E-06
ENSG00000160460	SPTBN4	0.06150772	0.441856083	-2.830794476	2.18E-07
ENSG00000120913	PDLIM2	0.394647864	2.822085748	-2.832199296	6.07E-10
ENSG00000119681	LTBP2	0.239010388	1.713515174	-2.834180497	3.34E-09
ENSG00000148516	ZEB1	0.618328057	4.42973785	-2.834969363	5.45E-10
ENSG00000163362	INAVA	1.24283347	8.906314863	-2.835644214	3.95E-10
ENSG00000134109	EDEM1	2.467060499	17.69196489	-2.837270921	1.80E-10
ENSG00000196275	GTF2IRD2	0.03321138	0.243116704	-2.840876807	8.24E-05
ENSG00000135424	ITGA7	0.327831875	2.374067978	-2.848571876	3.02E-09
ENSG00000183098	GPC6	0.019381007	0.145334812	-2.849602879	0.001665
ENSG00000223685	LINC00571	0.105964795	0.794611632	-2.849602879	0.001665
ENSG00000236714	LINC01844	0.056037708	0.420217059	-2.849602879	0.001665
ENSG00000172901	LVRN	0.018890348	0.141655449	-2.849602879	0.001665
ENSG00000153208	MERTK	1.137986744	8.238319255	-2.850056579	4.43E-10
ENSG00000103044	HAS3	2.765155562	20.04142046	-2.852536874	1.53E-10
ENSG00000118263	KLF7	0.054137919	0.396304902	-2.854029503	8.41E-07
ENSG00000181264	TMEM136	0.439551519	3.205953229	-2.858679746	3.18E-09
ENSG00000106327	TFR2	0.032999369	0.247456548	-2.862495178	0.000466
ENSG00000107281	NPDC1	1.847292489	13.49272036	-2.86281301	4.02E-10
ENSG00000226711	FAM66C	0.044824556	0.336131574	-2.87030415	0.00016
ENSG00000148841	ITPRIP	0.26406522	1.943281569	-2.87148028	2.85E-09

ENSG00000153086	ACMSD	0.390097565	2.877055236	-2.871957161	2.48E-08
ENSG00000015520	NPC1L1	0.103429334	0.767075586	-2.873846303	4.93E-07
ENSG00000138079	SLC3A1	0.038329415	0.287425642	-2.875541232	6.40E-05
ENSG00000254614	AP003068.2	0.326631106	2.424356689	-2.875864854	3.52E-07
ENSG00000104953	TLE6	0.217394185	1.617944167	-2.882770537	8.29E-08
ENSG00000171236	LRG1	0.13337201	1.00013359	-2.886091	3.73E-06
ENSG00000145632	PLK2	3.794693829	28.15532776	-2.88642369	9.05E-11
ENSG00000143479	DYRK3	0.481843297	3.582288589	-2.886470913	1.93E-09
ENSG00000135046	ANXA1	28.9685585	215.1201206	-2.887974792	5.59E-11
ENSG00000152076	CCDC74B	0.260751643	1.9453552	-2.888985851	1.62E-08
ENSG00000228262	LINC01320	0.089417425	0.670525778	-2.889766579	4.23E-07
ENSG00000132793	LPIN3	2.067318711	15.4142712	-2.893163991	1.22E-10
ENSG00000185483	ROR1	1.070304919	8.026031101	-2.901170328	1.46E-10
ENSG00000139508	SLC46A3	0.652897668	4.931530386	-2.909815464	9.58E-10
ENSG00000140682	TGFB1I1	0.409888745	3.098539258	-2.91068331	1.24E-09
ENSG00000213654	GPSM3	0.112689997	0.869186834	-2.910808178	0.000121
ENSG00000171847	FAM90A1	0.065855714	0.511477753	-2.912907359	0.000342
ENSG00000166415	WDR72	0.175487194	1.331614104	-2.914700747	4.93E-09
ENSG00000006534	ALDH3B1	1.412608055	10.69423816	-2.915122562	9.89E-11
ENSG00000283828	AL137002.2	0.056012669	0.44003069	-2.916341662	0.001174
ENSG00000240889	NDUFB2-AS1	0.076065748	0.597565949	-2.916341662	0.001174
ENSG00000084764	MAPRE3	0.195689439	1.492104065	-2.91667928	9.06E-08
ENSG00000143786	CNIH3	0.163582253	1.247703681	-2.920178708	1.66E-08
ENSG00000250770	AC005865.2	0.013972731	0.112263305	-2.922978807	0.007865
ENSG00000269961	AC010359.1	0.164834125	1.324352721	-2.922978807	0.007865
ENSG00000266983	AC011444.1	0.130783883	1.050777511	-2.922978807	0.007865
ENSG00000267284	AC022031.2	0.067888628	0.545448277	-2.922978807	0.007865
ENSG00000260963	AC026462.3	0.073501233	0.590542506	-2.922978807	0.007865
ENSG00000229512	AC068580.1	0.331630562	2.664471545	-2.922978807	0.007865
ENSG00000270878	AL136038.4	0.066273514	0.532471712	-2.922978807	0.007865
ENSG00000237153	AL162725.2	0.020210617	0.162381337	-2.922978807	0.007865
ENSG00000243649	CFB	0.016724215	0.134369988	-2.922978807	0.007865
ENSG00000225914	HCG23	0.009479458	0.076162299	-2.922978807	0.007865
ENSG00000089127	OAS1	0.016893249	0.135728084	-2.922978807	0.007865
ENSG00000269959	SPACA6P-AS	0.02109311	0.169471688	-2.922978807	0.007865
ENSG00000214063	TSPAN4	2.539605379	19.35826846	-2.925261216	5.87E-11
ENSG00000137962	ARHGAP29	4.056343358	30.99739507	-2.929231004	3.37E-11
ENSG00000141012	GALNS	0.5420869	4.150258511	-2.930564338	1.84E-10
ENSG00000273032	DGCR9	0.059559795	0.464858322	-2.936873623	1.46E-05
ENSG00000160867	FGFR4	4.473613179	34.38111811	-2.937233918	4.22E-11
ENSG00000159433	STARD9	0.106955901	0.825824041	-2.941207915	8.58E-10
ENSG00000151413	NUBPL	0.511995642	3.963217122	-2.946169796	2.20E-10
ENSG00000163462	TRIM46	0.08296251	0.651746316	-2.951314241	3.58E-06

ENSG00000006327	TNFRSF12A	23.80956691	184.8416578	-2.951965706	2.58E-11
ENSG00000234975	FTH1P2	4.766786858	37.15773716	-2.954569117	9.65E-10
ENSG00000116678	LEPR	0.657836032	5.137719803	-2.959864826	6.66E-11
ENSG00000153246	PLA2R1	0.092511905	0.725793929	-2.962524654	3.33E-09
ENSG00000214353	VAC14-AS1	0.060271617	0.47963782	-2.964822385	1.17E-05
ENSG00000253669	GASAL1	0.236326339	1.86710795	-2.967279097	6.90E-08
ENSG00000133069	TMCC2	0.141974886	1.120679019	-2.967583997	3.37E-08
ENSG00000233237	LINC00472	0.958771597	7.566696771	-2.973791523	1.97E-10
ENSG00000227014	AC007285.1	0.088403633	0.710275207	-2.974800821	3.04E-05
ENSG00000123453	SARDH	0.089773429	0.716909375	-2.978165043	3.84E-07
ENSG00000272692	AC010997.3	0.066326112	0.544736405	-2.980129145	0.00083
ENSG00000261502	AC040174.1	0.032772903	0.269163871	-2.980129145	0.00083
ENSG00000198049	AVPR1B	0.055150177	0.45294844	-2.980129145	0.00083
ENSG00000021488	SLC7A9	0.12871914	1.034188417	-2.981451414	5.08E-06
ENSG00000188937	NYX	0.279688425	2.232163712	-2.983880654	2.07E-08
ENSG00000232533	AC093673.1	0.967255805	7.736835895	-2.984409822	8.71E-08
ENSG00000197043	ANXA6	5.297289501	42.1319379	-2.986794535	1.98E-11
ENSG00000062282	DGAT2	0.449305922	3.578784703	-2.987229199	1.46E-10
ENSG00000105737	GRIK5	0.041668778	0.339249827	-2.988556526	7.02E-05
ENSG00000100505	TRIM9	0.16349813	1.306112334	-2.988732287	2.25E-09
ENSG00000106258	CYP3A5	0.129826079	1.039770698	-2.989324646	1.61E-08
ENSG00000003096	KLHL13	0.281242473	2.250015152	-2.99203075	6.14E-10
ENSG00000226690	AC013470.2	0.078270847	0.634852947	-2.99223998	9.35E-06
ENSG00000162909	CAPN2	5.565373389	44.46782384	-2.993515407	1.45E-11
ENSG00000161714	PLCD3	4.910100445	39.2640322	-2.994621313	1.67E-11
ENSG00000102265	TIMP1	7.34838232	58.79335422	-2.994996605	2.82E-11
ENSG00000116183	PAPPA2	0.064991095	0.524215884	-2.997737289	3.87E-08
ENSG00000166589	CDH16	1.600099318	12.88041693	-3.003224997	4.90E-11
ENSG00000103316	CRYM	0.198873113	1.609673344	-3.00326211	2.87E-08
ENSG00000112186	CAP2	1.585480397	12.77539747	-3.004638268	4.91E-11
ENSG00000165072	MAMDC2	0.086736795	0.708497779	-3.005250596	4.15E-06
ENSG00000118965	WDR35	2.67158127	21.5475365	-3.006874715	1.70E-11
ENSG00000259605	AC074212.1	0.064285309	0.533714146	-3.00873621	0.000186
ENSG00000137726	FXYD6	0.032536851	0.2701298	-3.00873621	0.000186
ENSG00000144935	TRPC1	0.192801996	1.567835857	-3.011628265	1.05E-08
ENSG00000100097	LGALS1	58.66692495	474.7579159	-3.011910104	1.10E-11
ENSG00000134871	COL4A2	1.806306222	14.65190044	-3.015220783	1.26E-11
ENSG00000260473	AC068987.3	0.038798004	0.332502299	-3.015367104	0.005247
ENSG00000227482	AL157702.2	0.068332708	0.585617295	-3.015367104	0.005247
ENSG00000270761	AL355353.1	0.253245156	2.170333186	-3.015367104	0.005247
ENSG00000235527	HIPK1-AS1	0.027071883	0.232008407	-3.015367104	0.005247
ENSG00000152583	SPARCL1	0.024035347	0.205985031	-3.015367104	0.005247
ENSG00000135838	NPL	0.277169824	2.258400726	-3.016964052	1.93E-09

ENSG00000260018	AC040169.1	1.12283361	9.193193954	-3.01725115	8.48E-08
ENSG00000110628	SLC22A18	0.550222264	4.479680377	-3.018421903	1.75E-10
ENSG00000183426	NPIPA1	0.068905512	0.565921014	-3.019808577	1.70E-07
ENSG00000135549	PKIB	0.267855454	2.191201774	-3.020235183	9.56E-09
ENSG00000111181	SLC6A12	0.49066249	4.001551812	-3.020568231	2.19E-10
ENSG00000144063	MALL	0.105552133	0.876322564	-3.028663542	3.39E-06
ENSG00000130052	STARD8	0.428530172	3.519518585	-3.030487586	2.32E-10
ENSG00000198363	ASPH	9.425540397	77.44620752	-3.033954264	7.63E-12
ENSG00000243083	LINC00870	0.100580117	0.838036517	-3.036052396	1.68E-06
ENSG00000179431	FJX1	1.108593592	9.153601674	-3.038537459	1.57E-10
ENSG00000250091	DNAH10OS	0.111372183	0.924642199	-3.038760908	3.26E-08
ENSG00000152661	GJA1	1.714023764	14.15159935	-3.039781008	3.01E-11
ENSG00000161653	NAGS	0.340561789	2.823471194	-3.039828565	5.90E-09
ENSG00000113578	FGF1	0.023764237	0.203661598	-3.041215335	0.000589
ENSG00000186231	KLHL32	0.030396788	0.260503135	-3.041215335	0.000589
ENSG00000243444	PALM2	0.025360379	0.217340669	-3.041215335	0.000589
ENSG00000081237	PTPRC	0.01367325	0.117180948	-3.041215335	0.000589
ENSG00000124813	RUNX2	0.015302289	0.131141959	-3.041215335	0.000589
ENSG00000166793	YPEL4	0.028106805	0.240877786	-3.041215335	0.000589
ENSG00000188176	SMTNL2	0.169171865	1.413572272	-3.041955064	9.01E-07
ENSG00000236924	AL162411.1	1.112160286	9.276008163	-3.043938612	6.44E-08
ENSG00000159733	ZFYVE28	0.030392576	0.255815847	-3.045559838	6.02E-06
ENSG00000272405	AL365181.3	2.567820996	21.28401702	-3.045770453	1.82E-11
ENSG00000152784	PRDM8	0.100183077	0.839064623	-3.046779003	1.97E-07
ENSG00000229743	LINC01159	2.2195793	18.42087293	-3.047506809	2.06E-11
ENSG00000263874	LINC00672	0.26031023	2.170259705	-3.047905441	5.38E-09
ENSG00000119632	IFI27L2	2.01133337	16.72557084	-3.049228898	7.31E-11
ENSG00000169884	WNT10B	0.381457139	3.183987755	-3.049877498	4.49E-09
ENSG00000107984	DKK1	1.325920922	11.03859399	-3.050595078	1.13E-10
ENSG00000264278	ZNF236-DT	0.129067029	1.106115755	-3.054364748	0.000138
ENSG00000131067	GGT7	1.028434897	8.617580121	-3.060031624	7.26E-11
ENSG00000273402	AC004908.3	0.334283606	2.864839806	-3.062328321	4.11E-05
ENSG00000139567	ACVRL1	0.232141393	1.9539458	-3.062913668	2.05E-09
ENSG00000101353	MROH8	0.064650001	0.554056177	-3.067668609	1.47E-05
ENSG00000172183	ISG20	0.113501156	0.960555924	-3.068426255	8.16E-09
ENSG00000204682	CASC10	0.351784905	2.979497463	-3.07262702	1.10E-09
ENSG00000171408	PDE7B	0.182714719	1.550824609	-3.074514279	2.33E-09
ENSG00000230426	ERVMER61-1	0.037080365	0.317781974	-3.076625285	1.16E-06
ENSG00000065357	DGKA	0.286341806	2.427681798	-3.076868617	8.11E-11
ENSG00000182890	GLUD2	0.402266674	3.429505132	-3.080392345	3.17E-09
ENSG00000023445	BIRC3	0.655850384	5.59092465	-3.08569948	2.21E-11
ENSG00000132692	BCAN	0.262694986	2.251319052	-3.089460346	9.97E-10
ENSG00000165092	ALDH1A1	0.572001607	4.902103914	-3.090544023	4.36E-10

ENSG00000196593	ANKRD20A19P	1.524361938	13.07992512	-3.094908533	2.30E-11
ENSG00000257354	AC048341.1	0.192271716	1.652934792	-3.095148754	2.65E-10
ENSG00000049130	KITLG	5.023681912	43.09667858	-3.095982276	4.32E-12
ENSG00000231721	LINC-PINT	0.032391822	0.282557913	-3.096979108	3.90E-06
ENSG00000135048	CEMIP2	1.072312347	9.213064375	-3.097733127	7.69E-12
ENSG00000166035	LIPC	0.040302325	0.354029321	-3.097846196	3.15E-05
ENSG00000266313	AC026254.2	0.184076876	1.676152144	-3.102193375	0.003515
ENSG00000250794	ALG1L12P	0.192559681	1.753394178	-3.102193375	0.003515
ENSG00000283709	FAM238C	0.015071398	0.137235901	-3.102193375	0.003515
ENSG00000258777	HIF1A-AS1	0.128176229	1.167136616	-3.102193375	0.003515
ENSG00000233532	LINC00460	0.019747377	0.179814053	-3.102193375	0.003515
ENSG00000236204	LINC01376	0.027463326	0.250073307	-3.102193375	0.003515
ENSG00000184368	MAP7D2	0.019097555	0.173896955	-3.102193375	0.003515
ENSG00000282164	PEG13	0.01479131	0.1346855	-3.102193375	0.003515
ENSG00000263400	TMEM220-AS1	0.038511936	0.350678836	-3.102193375	0.003515
ENSG00000183242	WT1-AS	0.015028035	0.136841049	-3.102193375	0.003515
ENSG00000236753	MKLN1-AS	0.370754903	3.221532603	-3.108566833	1.37E-09
ENSG00000178401	DNAJC22	0.400760831	3.478363558	-3.109681494	1.21E-10
ENSG00000166033	HTRA1	19.50843335	169.1531401	-3.111448409	2.89E-12
ENSG00000137331	IER3	5.731632626	49.7478059	-3.112172621	8.44E-12
ENSG00000135480	KRT7	0.316479104	2.755999691	-3.112532801	7.58E-10
ENSG00000180801	ARSJ	0.170519717	1.496163423	-3.120871839	3.70E-09
ENSG00000170537	TMC7	0.051879772	0.460493273	-3.122017442	3.15E-06
ENSG00000236144	TMEM147-AS1	0.791163093	6.918523275	-3.122069289	2.20E-11
ENSG00000285517	LINC00941	0.050812381	0.447299886	-3.126280566	2.20E-09
ENSG00000215039	CD27-AS1	0.526758834	4.632477881	-3.1281816	1.41E-10
ENSG00000134070	IRAK2	0.179954568	1.593633972	-3.131100659	1.63E-08
ENSG00000128253	RFPL2	0.076868011	0.691703871	-3.132510621	2.42E-05
ENSG00000143353	LYPLAL1	0.676231165	5.976465373	-3.135712389	9.12E-11
ENSG00000160932	LY6E	3.133908807	27.65479239	-3.136104028	5.36E-12
ENSG00000063180	CA11	2.215132329	19.56633813	-3.136852095	1.20E-11
ENSG00000125246	CLYBL	0.537712318	4.756894654	-3.138782435	1.76E-11
ENSG00000244560	AC004890.2	0.194894826	1.73290071	-3.139631778	3.66E-09
ENSG00000066923	STAG3	0.038947175	0.349426677	-3.140346877	1.26E-06
ENSG00000253958	CLDN23	0.443091456	3.941796355	-3.141457313	1.85E-09
ENSG00000251417	AC145285.2	0.085146104	0.775316427	-3.141508141	7.63E-05
ENSG00000149599	DUSP15	0.044089107	0.401462977	-3.141508141	7.63E-05
ENSG00000253320	AZIN1-AS1	0.062496935	0.562384422	-3.148710784	2.75E-07
ENSG00000167996	FTH1	236.2627605	2102.543684	-3.149100292	1.52E-12
ENSG00000131370	SH3BP5	0.261221861	2.335560824	-3.14952209	9.70E-10
ENSG00000067082	KLF6	4.968786293	44.26729328	-3.15049244	2.02E-12
ENSG00000169252	ADRB2	0.072818096	0.663061057	-3.154918225	5.70E-06
ENSG00000184451	CCR10	0.047864205	0.4443838	-3.156136218	0.0003

ENSG00000232938	RPL23AP87	0.055565759	0.515887044	-3.156136218	0.0003
ENSG00000225193	RPS12P26	0.316556445	2.938992856	-3.156136218	0.0003
ENSG00000116852	KIF21B	0.085978294	0.773683589	-3.158541575	1.01E-09
ENSG00000177337	DLGAP1-AS1	0.218356275	1.97765812	-3.159520613	6.41E-08
ENSG00000184985	SORCS2	0.231516666	2.083324024	-3.160423659	2.71E-10
ENSG00000153982	GDPD1	0.225106805	2.034687362	-3.161265691	8.66E-09
ENSG00000196139	AKR1C3	3.817117523	34.29337146	-3.162417715	2.13E-12
ENSG00000106003	LFNG	0.146701407	1.335821662	-3.168486329	3.74E-08
ENSG00000090530	P3H2	0.954244014	8.649145599	-3.174277201	6.12E-12
ENSG00000111799	COL12A1	0.227658894	2.072997716	-3.179781126	2.21E-11
ENSG00000223745	CCDC18-AS1	0.384153747	3.50734867	-3.182306323	6.47E-11
ENSG00000213420	GPC2	0.065494437	0.608067487	-3.182866987	4.53E-06
ENSG00000159403	C1R	0.028401326	0.266220776	-3.18318226	5.70E-05
ENSG00000240288	GHRLOS	0.097629558	0.915133919	-3.18318226	5.70E-05
ENSG00000138472	GUCA1C	0.141645596	1.327719719	-3.18318226	5.70E-05
ENSG00000177508	IRX3	2.540716175	23.17348842	-3.183675001	3.26E-12
ENSG00000254208	AC011773.1	0.178570302	1.721658537	-3.184089459	0.002365
ENSG00000272438	AL645608.6	0.238093737	2.295544716	-3.184089459	0.002365
ENSG00000228522	AL845321.1	0.09572841	0.922950968	-3.184089459	0.002365
ENSG00000279900	AP001767.4	0.184891375	1.782602202	-3.184089459	0.002365
ENSG00000227071	FOCAD-AS1	0.197567143	1.9048137	-3.184089459	0.002365
ENSG00000237264	FTH1P11	0.151671328	1.462316144	-3.184089459	0.002365
ENSG00000155093	PTPRN2	0.017291724	0.166715538	-3.184089459	0.002365
ENSG00000099953	MMP11	0.532812935	4.877589024	-3.187400164	2.17E-11
ENSG00000275216	AL161431.1	0.197139929	1.812699651	-3.189397592	8.21E-10
ENSG00000273796	BX322562.1	0.819322564	7.580207819	-3.190177978	4.69E-08
ENSG00000272734	ADIRF-AS1	0.203547777	1.876811481	-3.194374245	4.04E-10
ENSG00000128604	IRF5	0.582608571	5.381806206	-3.200216072	2.17E-11
ENSG00000075426	FOSL2	2.306169212	21.30266779	-3.202486656	1.28E-12
ENSG00000147509	RGS20	0.484355162	4.487531024	-3.202747504	1.29E-10
ENSG00000100379	KCTD17	0.644929786	5.976985416	-3.20379081	5.21E-11
ENSG00000233429	HOTAIRM1	2.15072173	19.9279674	-3.205189827	9.71E-12
ENSG00000272079	AC004233.3	0.395134286	3.703804418	-3.210257705	2.42E-08
ENSG00000272121	AC006058.3	0.234530126	2.219312876	-3.210284583	3.60E-06
ENSG00000219755	AL137784.1	0.16826356	1.622287642	-3.210336674	0.000215
ENSG00000234928	LINC01659	0.115749171	1.11597811	-3.210336674	0.000215
ENSG00000271605	MILR1	0.069028828	0.665530998	-3.210336674	0.000215
ENSG00000137709	POU2F3	0.018864763	0.18188176	-3.210336674	0.000215
ENSG00000196754	S100A2	1.333294536	12.45483164	-3.215760814	2.92E-11
ENSG00000120129	DUSP1	1.386639525	12.94782725	-3.21600975	1.42E-11
ENSG00000174791	RIN1	0.809884399	7.579201122	-3.220133232	4.02E-12
ENSG00000253549	CA3-AS1	0.132968817	1.28199872	-3.223686288	4.27E-05
ENSG00000108242	CYP2C18	0.069123988	0.666448466	-3.223686288	4.27E-05

ENSG00000171488	LRRC8C	0.071281902	0.67198136	-3.223964308	1.27E-09
ENSG00000116729	WLS	2.702169449	25.35141233	-3.224743708	1.12E-12
ENSG00000256940	AP001453.2	0.349961899	3.37410467	-3.23177021	1.12E-05
ENSG00000100218	RSPH14	0.085766525	0.826904962	-3.23177021	1.12E-05
ENSG00000277196	AC007325.2	3.266388666	30.84095347	-3.233636936	1.58E-12
ENSG00000107819	SFXN3	1.96701107	18.58766649	-3.234948315	1.32E-12
ENSG00000256546	AC156455.1	0.547706618	5.207288355	-3.237578875	4.65E-10
ENSG00000182195	LDOC1	1.694413644	16.07712418	-3.238623837	1.60E-11
ENSG00000095383	TBC1D2	2.724397767	25.84124808	-3.240415776	1.08E-12
ENSG00000120694	HSPH1	2.779056201	26.36702607	-3.241212552	6.33E-13
ENSG00000072756	TRNT1	1.635675492	15.55108822	-3.243729089	1.17E-12
ENSG00000151632	AKR1C2	0.355703419	3.39481965	-3.247004646	1.49E-11
ENSG00000271270	TMCC1-AS1	0.294452247	2.819799483	-3.249888044	1.03E-10
ENSG00000106538	RARRES2	1.935921456	18.57168569	-3.255583082	3.88E-12
ENSG00000105329	TGFB1	1.85827823	17.83336236	-3.25682571	1.54E-12
ENSG00000262903	AC027796.4	0.09561888	0.973111347	-3.261585294	0.001085
ENSG00000270019	AC110769.2	0.05398637	0.549418164	-3.261585294	0.001085
ENSG00000100079	LGALS2	0.10093104	1.027173088	-3.261585294	0.001085
ENSG00000229404	LINC00858	0.029027753	0.295414838	-3.261585294	0.001085
ENSG00000251284	AC111000.5	0.106414561	1.06397913	-3.262574393	0.000155
ENSG00000185275	CD24P4	0.515869762	5.157890596	-3.262574393	0.000155
ENSG00000173212	MAB21L3	0.038500108	0.384940852	-3.262574393	0.000155
ENSG00000169184	MN1	0.027158099	0.26765938	-3.263395034	8.67E-06
ENSG00000082497	SERTAD4	0.053502498	0.524024109	-3.263755082	9.00E-07
ENSG00000125966	MMP24	0.104132297	1.014116945	-3.264086875	2.18E-08
ENSG00000144893	MED12L	0.072848872	0.709455931	-3.271607949	4.82E-10
ENSG00000196154	S100A4	79.41273959	771.0611525	-3.274760251	2.74E-13
ENSG00000137203	TFAP2A	0.146456136	1.42826495	-3.275418279	1.33E-10
ENSG00000165376	CLDN2	0.159299372	1.562114813	-3.276339249	6.84E-09
ENSG00000169031	COL4A3	0.059536482	0.588730232	-3.288399879	6.00E-09
ENSG00000141448	GATA6	0.11651203	1.157373496	-3.292621868	1.61E-08
ENSG00000245532	NEAT1	0.545099437	5.363631427	-3.293498783	4.26E-13
ENSG00000156011	PSD3	1.126144404	11.09000241	-3.294831074	3.52E-13
ENSG00000145246	ATP10D	0.991519171	9.81810095	-3.302289185	6.06E-13
ENSG00000196739	COL27A1	0.185208604	1.836862454	-3.302766459	5.22E-12
ENSG00000164707	SLC13A4	0.072987687	0.731624327	-3.308908833	3.25E-09
ENSG00000238273	AC108058.1	0.048047663	0.497558657	-3.312986575	0.000112
ENSG00000112232	KHDRBS2	0.053754868	0.55665975	-3.312986575	0.000112
ENSG00000233328	PFN1P1	0.592701429	6.031910051	-3.31502371	1.46E-06
ENSG00000014914	MTMR11	0.357218891	3.591254414	-3.320759192	2.25E-11
ENSG00000162511	LAPTM5	0.102102506	1.039095073	-3.321877444	2.29E-07
ENSG00000127824	TUBA4A	2.704427311	27.16075677	-3.322936845	3.09E-13
ENSG00000165171	METTL27	0.682675846	6.886634148	-3.323001141	1.67E-10

ENSG00000132563	REEP2	0.470786138	4.743473338	-3.323719066	2.68E-11
ENSG00000126458	RRAS	6.267817614	63.17002642	-3.32768464	4.47E-13
ENSG00000271447	MMP28	0.102343651	1.049380396	-3.32972247	4.91E-07
ENSG00000260442	ATP2A1-AS1	0.645058417	6.603136344	-3.332571292	8.34E-08
ENSG00000170099	SERPINA6	0.248185723	2.537865986	-3.334393245	1.03E-08
ENSG00000154310	TNIK	0.568709643	5.761485955	-3.334674063	7.55E-13
ENSG00000262769	AC025627.1	0.114480687	1.226386903	-3.335129713	0.00074
ENSG00000258824	AL122035.1	0.151947094	1.627749889	-3.335129713	0.00074
ENSG00000244242	IFITM10	0.017468834	0.187136797	-3.335129713	0.00074
ENSG00000229051	LINC01788	0.040062752	0.429176625	-3.335129713	0.00074
ENSG00000236671	PRKG1-AS1	0.025698309	0.275295953	-3.335129713	0.00074
ENSG00000163347	CLDN1	49.59954679	503.1726854	-3.338059235	1.07E-13
ENSG00000272183	AC005041.3	0.257537447	2.689925665	-3.338792019	1.82E-05
ENSG00000091136	LAMB1	5.343815017	54.29315763	-3.340110245	1.15E-13
ENSG00000172594	SMPDL3A	0.2846178	2.915605645	-3.341686067	1.02E-09
ENSG00000124102	PI3	0.65176613	6.749378875	-3.349226754	7.06E-08
ENSG00000188042	ARL4C	2.051266833	21.03744032	-3.352962493	2.62E-13
ENSG00000165996	HACD1	0.357510987	3.679001727	-3.353614914	2.82E-11
ENSG00000073910	FRY	0.011084262	0.116366543	-3.354311369	4.11E-06
ENSG00000227218	AL157935.1	0.165815281	1.723024933	-3.356028297	3.44E-08
ENSG00000130270	ATP8B3	0.327658938	3.376060952	-3.357464383	3.42E-12
ENSG00000273002	AL355388.2	0.154976173	1.618694257	-3.359293397	1.59E-07
ENSG00000229848	AC139149.1	0.130988874	1.403232663	-3.361696515	8.10E-05
ENSG00000203952	CCDC160	0.070031482	0.750219921	-3.361696515	8.10E-05
ENSG00000231210	LINC01510	0.074131492	0.794141726	-3.361696515	8.10E-05
ENSG00000143867	OSR1	0.023612046	0.25294663	-3.361696515	8.10E-05
ENSG00000154229	PRKCA	1.614914749	16.69385081	-3.364839792	1.24E-13
ENSG00000132688	NES	0.541301269	5.605457518	-3.365437519	1.24E-12
ENSG00000077063	CTTNBP2	0.190068859	1.972504308	-3.365631146	2.69E-11
ENSG00000239552	HOXB-AS2	0.346151663	3.655213509	-3.372082414	3.30E-07
ENSG00000238271	IFNWP19	0.516781193	5.456988366	-3.372082414	3.30E-07
ENSG00000129991	TNNI3	0.143804403	1.518512987	-3.372082414	3.30E-07
ENSG00000197385	ZNF860	0.56345689	5.871472939	-3.372968946	5.18E-12
ENSG00000237357	BX088651.4	0.064808764	0.694270988	-3.375206431	1.38E-05
ENSG00000124215	CDH26	0.029078254	0.31150398	-3.375206431	1.38E-05
ENSG00000129295	LRRC6	0.225623384	2.36867492	-3.376356813	9.45E-10
ENSG00000198216	CACNA1E	0.011434911	0.122497734	-3.383386697	3.22E-06
ENSG00000226328	NUP50-DT	0.443151046	4.668182405	-3.385432544	7.21E-11
ENSG00000283154	IQCJ-SCHIP1	0.236932699	2.497556816	-3.386689083	6.00E-11
ENSG00000113946	CLDN16	0.244904958	2.598584808	-3.394871406	1.24E-10
ENSG00000130045	NXNL2	0.28436856	3.028409772	-3.39828642	3.88E-10
ENSG00000170955	CAVIN3	2.099269057	22.32801658	-3.404332659	5.54E-13
ENSG00000226822	AL390036.1	0.022519779	0.253307885	-3.405106199	0.000507

ENSG00000255443	CD44-AS1	0.142369509	1.601406407	-3.405106199	0.000507
ENSG00000229719	MIR194-2HG	0.022914972	0.257753102	-3.405106199	0.000507
ENSG00000226824	AC006001.2	0.133642167	1.479378231	-3.408815425	5.89E-05
ENSG00000232022	FAAHP1	0.050628575	0.560442965	-3.408815425	5.89E-05
ENSG00000231982	AC112907.2	0.506490312	5.561478789	-3.410724306	1.05E-05
ENSG00000199094	MIR30C2	2.321413931	25.49011112	-3.410724306	1.05E-05
ENSG00000154654	NCAM2	0.024931653	0.272424728	-3.411887614	2.52E-06
ENSG00000235374	SSR4P1	0.079259201	0.866054332	-3.411887614	2.52E-06
ENSG00000189056	RELN	0.019831728	0.215990384	-3.412670792	6.13E-07
ENSG00000204934	ATP6V0E2-AS1	0.101575085	1.10173591	-3.413658487	9.30E-08
ENSG00000136193	SCRN1	5.563560678	59.65948342	-3.417904437	4.47E-14
ENSG00000183840	GPR39	0.246740188	2.669663607	-3.422616699	1.20E-10
ENSG00000169302	STK32A	0.079209119	0.859850188	-3.424572983	5.42E-10
ENSG00000197747	S100A10	17.76490972	191.8170852	-3.427814514	4.19E-14
ENSG00000099377	HSD3B7	0.351701189	3.821460913	-3.429121635	8.16E-11
ENSG00000279312	AL136164.4	1.761447573	19.05839713	-3.429160247	3.44E-13
ENSG00000242960	FTH1P23	0.683761921	7.487649492	-3.429736406	3.11E-08
ENSG00000147454	SLC25A37	2.379826826	25.73591055	-3.429933273	4.71E-14
ENSG00000065534	MYLK	0.258249053	2.794792295	-3.430116295	1.42E-13
ENSG00000170558	CDH2	0.700644932	7.587123096	-3.430206157	3.87E-13
ENSG00000134369	NAV1	0.033498335	0.364835493	-3.431169982	1.80E-10
ENSG00000198959	TGM2	4.846197126	52.72868498	-3.438924686	2.79E-14
ENSG00000136327	NKX2-8	0.165683786	1.845901937	-3.439836376	1.56E-06
ENSG00000260025	AC009414.2	0.456671593	5.1367517	-3.44538873	7.98E-06
ENSG00000074276	CDHR2	0.031253142	0.351542843	-3.44538873	7.98E-06
ENSG00000196155	PLEKHG4	0.615833608	6.743789116	-3.446824393	1.72E-13
ENSG00000165730	STOX1	0.171251847	1.891883997	-3.450537594	2.86E-10
ENSG00000111863	ADTRP	0.03020635	0.345161422	-3.454443963	4.30E-05
ENSG00000251271	ALG1L7P	0.163224417	1.865130082	-3.454443963	4.30E-05
ENSG00000157782	CABP1	0.021399172	0.244523712	-3.454443963	4.30E-05
ENSG00000188859	FAM78B	0.023461792	0.268092814	-3.454443963	4.30E-05
ENSG00000172476	RAB40A	0.031198694	0.356500722	-3.454443963	4.30E-05
ENSG00000215277	RNF212B	0.060882153	0.69568718	-3.454443963	4.30E-05
ENSG00000180611	MB21D2	0.260538737	2.884084758	-3.455948162	5.88E-11
ENSG00000181804	SLC9A9	0.173807845	1.927650958	-3.456797583	1.76E-10
ENSG00000090971	NAT14	2.279557647	25.24780804	-3.463340787	1.21E-13
ENSG00000104894	CD37	0.321349297	3.571582911	-3.464508903	7.24E-12
ENSG00000234184	LINC01781	0.117871511	1.388982628	-3.471844981	0.000349
ENSG00000173080	RXFP4	0.067395888	0.794184422	-3.471844981	0.000349
ENSG00000174448	STARD6	0.044882332	0.528887585	-3.471844981	0.000349
ENSG00000176533	GNG7	0.219875207	2.463394785	-3.474288903	2.40E-11
ENSG00000064763	FAR2	0.895788632	10.00986464	-3.476102738	1.03E-13
ENSG00000153237	CCDC148	0.04410074	0.507866555	-3.47923976	6.09E-06

ENSG00000248668	OXCT1-AS1	0.08094034	0.932113436	-3.47923976	6.09E-06
ENSG00000088538	DOCK3	0.147955337	1.67190644	-3.488241025	5.99E-12
ENSG00000105464	GRIN2D	0.172294221	1.951190472	-3.488794703	3.92E-11
ENSG00000241388	HNF1A-AS1	0.187860087	2.127470151	-3.488794703	3.92E-11
ENSG00000162039	MEIOB	0.290302741	3.304268664	-3.493575248	1.71E-10
ENSG00000171608	PIK3CD	0.112184565	1.279552286	-3.497184495	1.08E-10
ENSG00000233818	AP000695.2	0.106414561	1.253975403	-3.498673527	3.15E-05
ENSG00000186976	EFCAB6	0.014649568	0.172628611	-3.498673527	3.15E-05
ENSG00000237505	PKN2-AS1	0.009032739	0.106440627	-3.498673527	3.15E-05
ENSG00000143469	SYT14	0.009001605	0.106073749	-3.498673527	3.15E-05
ENSG00000182871	COL18A1	6.886634406	78.14249631	-3.499558578	1.10E-14
ENSG00000248445	SEMA6A-AS1	0.19201515	2.199389874	-3.500251866	5.54E-10
ENSG00000228696	ARL17B	0.138657575	1.59148336	-3.504136021	3.11E-10
ENSG00000082014	SMARCD3	1.028960696	11.7376174	-3.50599644	6.00E-14
ENSG00000181778	TMEM252	3.993950715	45.73388846	-3.511391002	6.04E-14
ENSG00000215478	CES5AP1	0.107142181	1.262549594	-3.512314693	4.66E-06
ENSG00000261019	AC010132.4	0.03054492	0.392658965	-3.518999954	0.00839
ENSG00000227359	AC017074.1	0.074483869	0.957499935	-3.518999954	0.00839
ENSG00000250101	AC106795.3	0.076390221	0.982006332	-3.518999954	0.00839
ENSG00000267681	AC135721.1	0.139284836	1.790524878	-3.518999954	0.00839
ENSG00000135298	ADGRB3	0.006438436	0.082766944	-3.518999954	0.00839
ENSG00000231683	AL033397.1	0.010462056	0.134491103	-3.518999954	0.00839
ENSG00000258623	AL121820.1	0.090640891	1.165200572	-3.518999954	0.00839
ENSG00000258938	AL162311.3	0.014574625	0.187358725	-3.518999954	0.00839
ENSG00000238005	AL391832.2	0.025081303	0.322423447	-3.518999954	0.00839
ENSG00000254941	AP000866.4	0.093271095	1.199012195	-3.518999954	0.00839
ENSG00000250659	AP001363.1	0.158278223	2.034687362	-3.518999954	0.00839
ENSG00000255031	AP002807.1	0.034561994	0.444298977	-3.518999954	0.00839
ENSG00000254413	CHKB-CPT1B	0.00832048	0.106960865	-3.518999954	0.00839
ENSG00000188626	GOLGA8M	0.018976136	0.243940719	-3.518999954	0.00839
ENSG00000135077	HAVCR2	0.010984609	0.141208587	-3.518999954	0.00839
ENSG00000170523	KRT83	0.022285574	0.286483981	-3.518999954	0.00839
ENSG00000273769	LCA10	0.030018284	0.385888982	-3.518999954	0.00839
ENSG00000230836	LINC01293	0.033005885	0.424294995	-3.518999954	0.00839
ENSG00000177453	NIM1K	0.011546132	0.148427042	-3.518999954	0.00839
ENSG00000229873	OGFR-AS1	0.06255307	0.804127939	-3.518999954	0.00839
ENSG00000244586	WNT5A-AS1	0.083570902	1.074314927	-3.518999954	0.00839
ENSG00000091986	CCDC80	0.022377642	0.263695233	-3.530082307	7.14E-08
ENSG00000264107	AC138207.2	0.169859556	2.09258497	-3.535632464	0.000241
ENSG00000227959	AL451042.2	0.092445688	1.13888474	-3.535632464	0.000241
ENSG00000236816	ANKRD20A7P	0.034619263	0.426492049	-3.535632464	0.000241
ENSG00000250486	FAM218A	0.038423403	0.473357152	-3.535632464	0.000241
ENSG00000255200	PGAM1P8	0.035501657	0.437362704	-3.535632464	0.000241

ENSG00000140675	SLC5A2	0.01870432	0.230427888	-3.535632464	0.000241
ENSG00000056558	TRAF1	0.181422332	2.123975085	-3.538719089	4.90E-12
ENSG00000271780	AL118558.3	0.116178269	1.410515428	-3.541587357	2.31E-05
ENSG00000186047	DLEU7	0.034287843	0.416287239	-3.541587357	2.31E-05
ENSG00000260057	LINC01571	0.131263196	1.593660887	-3.541587357	2.31E-05
ENSG00000280374	AC019080.5	0.220794984	2.660951768	-3.54464832	3.58E-06
ENSG00000237515	SHISA9	0.028803363	0.343823093	-3.54866899	5.94E-08
ENSG00000134531	EMP1	2.665568433	31.31933667	-3.549640941	8.01E-15
ENSG00000188549	CCDC9B	0.650985052	7.659202626	-3.550485621	3.49E-14
ENSG00000125848	FLRT3	0.740705486	8.728377538	-3.552333877	5.87E-14
ENSG00000120075	HOXB5	1.187435641	14.03645177	-3.555764876	1.91E-13
ENSG00000237238	BMS1P10	0.706896723	8.414120669	-3.562383532	4.19E-12
ENSG00000164236	ANKRD33B	2.575552163	30.54214853	-3.56300824	5.91E-15
ENSG00000112218	GPR63	0.161469316	1.925295568	-3.563796751	9.38E-12
ENSG00000167757	KLK11	0.107383105	1.294147439	-3.565446556	1.67E-08
ENSG00000275160	AL354718.1	0.496601282	6.003882232	-3.567019258	4.94E-08
ENSG00000134138	MEIS2	0.027088179	0.327494595	-3.567019258	4.94E-08
ENSG00000186907	RTN4RL2	0.122537979	1.481477434	-3.567019258	4.94E-08
ENSG00000227210	AC079145.1	0.463424593	5.626418287	-3.569102509	1.42E-07
ENSG00000260027	HOXB7	3.473005397	41.39416972	-3.569271682	2.43E-14
ENSG00000188488	SERPINA5	0.15496591	1.861671562	-3.569886276	1.43E-10
ENSG00000169439	SDC2	2.256930211	26.93089943	-3.571575651	9.55E-15
ENSG00000118194	TNNT2	0.023025458	0.283662159	-3.576273144	2.75E-06
ENSG00000007174	DNAH9	0.088591062	1.065035411	-3.578306627	9.86E-13
ENSG00000133519	ZDHHC8P1	0.204517348	2.492165248	-3.591911351	4.75E-11
ENSG00000196975	ANXA4	5.158884398	62.44066551	-3.592672733	2.79E-15
ENSG00000224383	PRR29	0.121058766	1.480576463	-3.593656667	3.00E-10
ENSG00000255236	AP002992.1	0.107694461	1.384426452	-3.596718654	0.000167
ENSG00000185168	LINC00482	0.038038644	0.488991774	-3.596718654	0.000167
ENSG00000205863	C1QTNF9B	0.095139915	1.182268249	-3.597031433	3.90E-07
ENSG00000131016	AKAP12	2.012547165	24.47351102	-3.599163809	4.15E-15
ENSG00000144645	OSBPL10	2.962245131	36.20370736	-3.606616215	2.76E-15
ENSG00000105339	DENND3	0.287684426	3.530397553	-3.611022489	1.79E-14
ENSG00000184588	PDE4B	0.014502123	0.1864267	-3.623765504	1.26E-05
ENSG00000128242	GAL3ST1	0.263150005	3.278969159	-3.625747243	1.16E-11
ENSG00000188910	GJB3	0.385119362	4.800737001	-3.627561093	5.12E-12
ENSG00000103544	VPS35L	0.723020495	9.001205235	-3.632439923	5.76E-15
ENSG00000112246	SIM1	0.310890242	3.875866609	-3.63300509	4.19E-14
ENSG00000125845	BMP2	0.011603846	0.161599718	-3.633369841	0.005282
ENSG00000259462	CPEB1-AS1	0.008333756	0.116059151	-3.633369841	0.005282
ENSG00000130487	KLHDC7B	0.008140551	0.113368515	-3.633369841	0.005282
ENSG00000205312	KRT17P4	0.014097655	0.196329482	-3.633369841	0.005282
ENSG00000122367	LDB3	0.006259055	0.087166055	-3.633369841	0.005282

ENSG00000225950	NTF4	0.028444827	0.396133823	-3.633369841	0.005282
ENSG00000229474	PATL2	0.008758216	0.12197036	-3.633369841	0.005282
ENSG00000259863	SH3RF3-AS1	0.02605078	0.362793382	-3.633369841	0.005282
ENSG00000181585	TMIE	0.194250546	2.450873413	-3.633844818	3.57E-09
ENSG00000167992	VWCE	0.034292532	0.440835013	-3.637516107	1.64E-06
ENSG00000174567	GOLT1A	0.294263738	3.743394882	-3.643347203	7.37E-09
ENSG00000116717	GADD45A	3.55448977	44.65494622	-3.64557528	4.60E-15
ENSG00000160323	ADAMTS13	0.095166584	1.207694601	-3.647975215	8.71E-11
ENSG00000187498	COL4A1	0.89025862	11.2014109	-3.648209462	2.54E-15
ENSG00000232295	AL589935.1	0.016167712	0.216497978	-3.655323076	0.000117
ENSG00000187134	AKR1C1	0.829511515	10.51537571	-3.658552312	3.86E-15
ENSG00000132437	DDC	0.679546553	8.635742598	-3.659759805	6.33E-14
ENSG00000151474	FRMD4A	0.510692045	6.491661276	-3.662547254	3.55E-15
ENSG00000257642	KCCAT198	0.380559661	4.973680218	-3.669640181	2.00E-07
ENSG00000171903	CYP4F11	0.757471543	9.704049037	-3.672416882	1.60E-14
ENSG00000250564	AC109454.3	0.437216974	5.687392177	-3.67247383	1.41E-08
ENSG00000154217	PITPNC1	1.298614213	16.61943254	-3.672563179	2.18E-15
ENSG00000226337	AL353616.2	0.240284363	3.114627808	-3.674622441	3.66E-10
ENSG00000227403	LINC01806	0.246106988	3.182569462	-3.676066139	4.01E-11
ENSG00000171889	MIR31HG	0.953493507	12.31736913	-3.676705963	1.10E-11
ENSG00000115363	EVA1A	1.020271575	13.22991688	-3.689659715	1.97E-14
ENSG00000111052	LIN7A	0.498104711	6.488841079	-3.696762368	9.10E-15
ENSG00000135899	SP110	0.164341904	2.15828017	-3.704197818	6.24E-13
ENSG00000100557	CCDC198	0.305226083	4.010915238	-3.705703033	3.76E-13
ENSG00000095303	PTGS1	1.399823424	18.33502423	-3.705842246	1.61E-15
ENSG00000118523	CTGF	0.857503906	11.27211579	-3.708335306	3.89E-14
ENSG00000164418	GRIK2	0.011017917	0.153439838	-3.711639537	8.17E-05
ENSG00000136999	NOV	5.533875634	72.94730986	-3.715449706	8.22E-16
ENSG00000260063	AL512408.1	0.104463627	1.410038342	-3.716092172	1.29E-07
ENSG00000163536	SERPINI1	0.552991904	7.349445583	-3.722378683	2.22E-13
ENSG00000273270	AC090114.2	0.130320374	1.738743325	-3.725548439	1.36E-12
ENSG00000196668	LINC00173	0.182115766	2.4495074	-3.726001508	1.29E-09
ENSG00000171227	TMEM37	1.69548295	22.61592288	-3.731162996	4.36E-15
ENSG00000179761	PIPOX	0.030883556	0.430096516	-3.738871235	5.19E-06
ENSG00000271275	AC007326.1	0.091234609	1.368305038	-3.739335228	0.003346
ENSG00000236234	AC091132.2	0.054337387	0.8149333	-3.739335228	0.003346
ENSG00000253390	AC104561.1	0.066326112	0.994736044	-3.739335228	0.003346
ENSG00000276740	AL445649.1	0.060470985	0.906922876	-3.739335228	0.003346
ENSG00000256050	AL583722.1	0.031970506	0.479482561	-3.739335228	0.003346
ENSG00000285955	BX842242.1	0.014188608	0.212795826	-3.739335228	0.003346
ENSG00000184530	C6orf58	0.027310752	0.409597194	-3.739335228	0.003346
ENSG00000136542	GALNT5	0.006128696	0.091916062	-3.739335228	0.003346
ENSG00000257671	KRT7-AS	0.024681306	0.37016167	-3.739335228	0.003346

ENSG00000242242	NECTIN3-AS1	0.025096367	0.376386611	-3.739335228	0.003346
ENSG00000105499	PLA2G4C	0.006318683	0.094765418	-3.739335228	0.003346
ENSG00000244219	TMEM225B	0.029635071	0.44445653	-3.739335228	0.003346
ENSG00000119283	TRIM67	0.004480533	0.067197481	-3.739335228	0.003346
ENSG00000138311	ZNF365	0.007784175	0.116744357	-3.739335228	0.003346
ENSG00000120885	CLU	1.856850442	24.95936471	-3.743508652	6.24E-16
ENSG00000161638	ITGA5	0.965085638	13.05024897	-3.751734645	9.74E-16
ENSG00000244509	APOBEC3C	1.06761975	14.48685849	-3.755324694	5.00E-15
ENSG00000151789	ZNF385D	0.187131084	2.540334284	-3.755515752	9.89E-15
ENSG00000272068	AL365181.2	0.674377231	9.197140515	-3.761686742	1.40E-14
ENSG00000204176	SYT15	0.011675175	0.168846646	-3.765839993	5.75E-05
ENSG00000143507	DUSP10	0.086037304	1.198189263	-3.766713593	2.01E-08
ENSG00000167315	ACAA2	1.845414229	25.24103486	-3.76842726	5.96E-16
ENSG00000166432	ZMAT1	0.133464309	1.834847161	-3.769403347	4.74E-13
ENSG00000137251	TINAG	0.639870012	8.80722214	-3.773057534	9.55E-14
ENSG00000168497	CAVIN2	1.087016991	14.97187108	-3.777197053	2.65E-15
ENSG00000188641	DPYD	0.164071325	2.291198922	-3.792991798	1.56E-13
ENSG00000092421	SEMA6A	1.02976193	14.41032595	-3.80152307	2.88E-16
ENSG00000105137	SYDE1	0.790393142	11.14279984	-3.810536029	2.03E-15
ENSG00000166888	STAT6	1.557364018	22.0196777	-3.816422296	2.30E-16
ENSG00000237651	C2orf74	0.055162311	0.827305224	-3.818077713	4.06E-05
ENSG00000284308	C2orf81	0.027903473	0.418486616	-3.818077713	4.06E-05
ENSG00000240875	LINC00886	0.021865751	0.327934959	-3.818077713	4.06E-05
ENSG00000028277	POU2F2	0.00921196	0.138157784	-3.818077713	4.06E-05
ENSG00000255650	FAM222A-AS1	0.234749723	3.394955879	-3.821078683	1.15E-08
ENSG00000175505	CLCF1	1.184802654	16.86266468	-3.823469146	4.70E-15
ENSG00000168702	LRP1B	0.012260989	0.178632177	-3.826071898	4.46E-08
ENSG00000253552	HOXA-AS2	0.316496503	4.517845864	-3.826494936	1.35E-14
ENSG00000196730	DAPK1	0.638017436	9.12148494	-3.831771231	4.17E-16
ENSG00000074527	NTN4	0.972920961	13.92534951	-3.83290587	9.05E-16
ENSG00000109846	CRYAB	0.055089586	0.811460682	-3.833451939	2.27E-07
ENSG00000173706	HEG1	0.218926291	3.13905621	-3.833929963	4.95E-15
ENSG00000278834	AC073508.3	0.029447111	0.473183107	-3.838047221	0.002134
ENSG00000232528	AL109809.1	0.02671704	0.42931383	-3.838047221	0.002134
ENSG00000279170	AL137784.3	0.018262872	0.293464523	-3.838047221	0.002134
ENSG00000205090	TMEM240	0.030279312	0.486555673	-3.838047221	0.002134
ENSG00000259498	TPM1-AS	0.012458393	0.200192853	-3.838047221	0.002134
ENSG00000170703	TTLL6	0.008198048	0.131733731	-3.838047221	0.002134
ENSG00000153162	BMP6	0.315645491	4.572923251	-3.843954521	3.64E-13
ENSG00000157399	ARSE	0.537200027	7.776775879	-3.846407536	2.12E-14
ENSG00000213963	AC019080.1	0.052443042	0.767795845	-3.848211691	2.79E-10
ENSG00000183696	UPP1	4.345613843	62.96372715	-3.851967978	8.86E-17
ENSG00000047648	ARHGAP6	0.068891675	1.01050548	-3.85676951	6.16E-12

ENSG00000272398	CD24	22.34314061	324.9915393	-3.857836624	5.13E-17
ENSG00000266714	MYO15B	0.091685026	1.339982179	-3.858646713	6.44E-14
ENSG00000162878	PKDCC	0.18098157	2.657275775	-3.861309821	1.06E-12
ENSG00000003436	TFPI	0.26040105	3.806049981	-3.862556301	1.01E-15
ENSG00000168676	KCTD19	0.039617792	0.588110908	-3.862742223	1.87E-09
ENSG00000254027	AC009902.2	0.119216693	1.851826729	-3.868489894	2.88E-05
ENSG00000142512	SIGLEC10	0.022789992	0.354003419	-3.868489894	2.88E-05
ENSG00000158089	GALNT14	1.399862922	20.5731975	-3.871717881	2.04E-16
ENSG00000131094	C1QL1	0.806668934	11.92531048	-3.874423759	9.14E-14
ENSG00000263961	RHEX	0.210747344	3.140191499	-3.877014942	1.58E-11
ENSG00000229953	AL590666.2	0.992266641	14.76356265	-3.880999144	5.48E-13
ENSG00000167994	RAB3IL1	1.056451849	15.6507123	-3.882089747	6.91E-16
ENSG00000117834	SLC5A9	0.036855965	0.562623584	-3.884871209	1.42E-07
ENSG00000177469	CAVIN1	7.924826868	117.4856547	-3.885165285	4.49E-17
ENSG00000154760	SLFN13	0.174684594	2.600665892	-3.887053692	9.33E-15
ENSG00000266074	BAHCC1	0.040318478	0.604682565	-3.888809198	4.13E-12
ENSG00000157510	AFAP1L1	0.628419192	9.434294927	-3.901112956	6.06E-16
ENSG00000135253	KCP	0.182137817	2.742479684	-3.903001715	1.03E-14
ENSG00000251664	PCDHA12	0.068952889	1.050546317	-3.905641847	1.44E-10
ENSG00000101331	CCM2L	0.129216701	1.9725535	-3.906075971	3.52E-10
ENSG00000269113	TRABD2B	0.201976873	3.054634424	-3.909076341	1.22E-14
ENSG00000224081	SLC44A3-AS1	0.016314476	0.253417382	-3.909909543	1.12E-07
ENSG00000258655	ARHGAP5-AS1	0.067833524	1.065788618	-3.912393908	1.26E-06
ENSG00000173917	HOXB2	1.609884661	24.3552774	-3.912717434	3.17E-16
ENSG00000165863	C10orf82	0.03466234	0.556986171	-3.917199834	2.06E-05
ENSG00000125657	TNFSF9	1.305011075	19.86783611	-3.920402442	1.43E-15
ENSG00000099204	ABLIM1	1.681574072	25.70568763	-3.929215737	3.14E-17
ENSG00000234286	AC006026.3	0.292205949	5.008461198	-3.930435518	0.00137
ENSG00000250602	AC093535.1	0.01451892	0.248856828	-3.930435518	0.00137
ENSG00000272482	AC254633.1	0.121117249	2.075970874	-3.930435518	0.00137
ENSG00000277128	AL589743.4	0.00568974	0.097523142	-3.930435518	0.00137
ENSG00000154645	CHODL	0.013509683	0.231558342	-3.930435518	0.00137
ENSG00000280890	ELDR	0.014207906	0.243525995	-3.930435518	0.00137
ENSG00000231381	RNF2P1	0.041371733	0.709118764	-3.930435518	0.00137
ENSG00000182557	SPNS3	0.01556835	0.266844244	-3.930435518	0.00137
ENSG00000168477	TNXB	0.002626529	0.045019168	-3.930435518	0.00137
ENSG00000183801	OLFML1	0.048644297	0.768633351	-3.934520733	8.90E-08
ENSG00000154330	PGM5	0.196941931	3.038057294	-3.937784129	7.35E-15
ENSG00000125148	MT2A	34.1296099	525.1654955	-3.938955279	1.55E-17
ENSG00000214575	CPEB1	0.086470196	1.340441631	-3.939598643	3.53E-13
ENSG00000166801	FAM111A	0.225679626	3.496247166	-3.944443453	4.20E-15
ENSG00000117114	ADGRL2	3.766833903	58.23708723	-3.945707798	1.82E-17
ENSG00000178038	ALS2CL	0.562860218	8.718355652	-3.947223523	9.86E-17

ENSG00000168679	SLC16A4	0.51456665	8.014111194	-3.952095389	3.64E-15
ENSG00000078487	ZCWPW1	0.20149705	3.156892869	-3.954268593	4.51E-13
ENSG00000138152	BTBD16	0.39944529	6.251209052	-3.955952023	4.56E-14
ENSG00000254109	RBPMS-AS1	0.142734247	2.293584387	-3.958719109	7.08E-08
ENSG00000188015	S100A3	0.175017595	2.812342741	-3.958719109	7.08E-08
ENSG00000145850	TIMD4	0.10748669	1.727194417	-3.958719109	7.08E-08
ENSG00000144218	AFF3	0.062332069	0.980522182	-3.961802009	1.30E-13
ENSG00000250548	LINC01303	0.016233664	0.269552599	-3.964318744	1.47E-05
ENSG00000011590	ZBTB32	0.030170001	0.500959127	-3.964318744	1.47E-05
ENSG00000146038	DCDC2	1.154201802	18.12565967	-3.967246337	5.39E-17
ENSG00000128298	BAIAP2L2	1.421406293	22.33984325	-3.967284872	2.16E-16
ENSG00000124920	MYRF	0.622086767	9.774119889	-3.968125989	4.67E-17
ENSG00000136883	KIF12	0.756982804	11.91324194	-3.968438127	5.83E-16
ENSG00000174099	MSRB3	0.648930025	10.2099815	-3.969701139	7.55E-17
ENSG00000235034	C19orf81	0.282334127	4.536802901	-3.972866752	1.92E-09
ENSG00000204362	AL590644.1	0.06255307	1.005159924	-3.976938063	5.27E-10
ENSG00000144824	PHLDB2	0.614442001	9.731587811	-3.979806045	3.11E-17
ENSG00000148143	ZNF462	0.098848036	1.584729173	-3.9923167	8.07E-15
ENSG00000135447	PPP1R1A	0.325301098	5.238476854	-3.999121294	5.29E-15
ENSG00000167972	ABCA3	1.263629726	20.32810152	-4.002533076	1.56E-17
ENSG00000101076	HNF4A	0.062869643	1.022492322	-4.003235896	3.34E-12
ENSG00000163638	ADAMTS9	0.794236564	12.79185845	-4.004096278	1.80E-17
ENSG00000185345	PRKN	0.044092984	0.724271407	-4.004533538	1.36E-09
ENSG00000246228	CASC8	0.071367123	1.1850186	-4.005931236	4.50E-08
ENSG00000267498	AC007786.1	0.102289965	1.753267935	-4.009947283	1.06E-05
ENSG00000170477	KRT4	0.02523276	0.432493932	-4.009947283	1.06E-05
ENSG00000116962	NID1	0.477425853	7.748050612	-4.013322404	1.76E-16
ENSG00000154734	ADAMTS1	1.08856335	17.66948662	-4.015256569	1.79E-17
ENSG00000095637	SORBS1	0.270912885	4.406414526	-4.016688209	1.08E-16
ENSG00000144583	4-Mar	0.009396324	0.171120547	-4.017261789	0.000885
ENSG00000242828	AC068756.1	0.103685982	1.888270653	-4.017261789	0.000885
ENSG00000274414	AL121772.1	0.017895268	0.325898532	-4.017261789	0.000885
ENSG00000272825	AL844908.1	0.064088115	1.167136616	-4.017261789	0.000885
ENSG00000225506	CYP4A22-AS1	0.045369653	0.826246551	-4.017261789	0.000885
ENSG00000230583	GTF2IRD1P1	0.012797994	0.233069854	-4.017261789	0.000885
ENSG00000207425	RF00019	0.417854508	7.609730733	-4.017261789	0.000885
ENSG00000261713	SSTR5-AS1	0.010389222	0.189202654	-4.017261789	0.000885
ENSG00000167094	TTC16	0.006559725	0.119462021	-4.017261789	0.000885
ENSG00000162366	PDZK1IP1	1.481959658	24.28041386	-4.026234117	3.14E-16
ENSG00000095587	TLL2	0.210183227	3.448808808	-4.027563771	6.53E-16
ENSG00000141837	CACNA1A	0.003872699	0.065341567	-4.028970282	3.60E-08
ENSG00000089820	ARHGAP4	0.644005155	10.62121388	-4.037143259	5.36E-17
ENSG00000126217	MCF2L	0.037908004	0.628478255	-4.038425864	2.54E-14

ENSG00000198914	POU3F3	2.809335136	46.45780935	-4.042214525	9.96E-18
ENSG00000160255	ITGB2	0.04303342	0.724428927	-4.043976839	2.46E-10
ENSG00000119042	SATB2	0.076264739	1.270426258	-4.044873512	3.03E-14
ENSG00000196639	HRH1	0.158221144	2.655439438	-4.056067207	1.88E-14
ENSG00000131018	SYNE1	0.032656222	0.549044024	-4.062079211	1.08E-15
ENSG00000108511	HOXB6	3.498167537	58.75589511	-4.06470228	6.87E-18
ENSG00000165164	CFAP47	0.021905872	0.375470486	-4.065855191	6.96E-10
ENSG00000106571	GLI3	0.022896137	0.392443809	-4.065855191	6.96E-10
ENSG00000139549	DHH	0.026665891	0.466580084	-4.067268694	3.28E-07
ENSG00000279673	AC092919.2	0.228613408	3.918473898	-4.07541558	1.90E-11
ENSG00000006210	CX3CL1	0.125712538	2.147253218	-4.080031884	3.66E-14
ENSG00000111331	OAS3	0.035450025	0.613044817	-4.082754038	1.58E-10
ENSG00000157110	RBPMS	0.725198171	12.38992694	-4.088961052	7.59E-18
ENSG00000140859	KIFC3	0.50490753	8.635552278	-4.090129824	1.21E-17
ENSG00000059377	TBXAS1	0.262843964	4.50519163	-4.09026459	5.02E-16
ENSG00000159173	TNNI1	0.105518815	1.814560367	-4.09029286	2.18E-14
ENSG00000183044	ABAT	0.107208156	1.842160499	-4.091359056	4.27E-15
ENSG00000132932	ATP8A2	0.020060226	0.352431685	-4.095723853	2.82E-09
ENSG00000240476	LINC00973	0.130308058	2.32656559	-4.096344022	2.53E-07
ENSG00000046889	PREX2	0.298021958	5.122997869	-4.096903059	2.17E-17
ENSG00000237594	AP000251.1	0.101667763	1.851515994	-4.097090676	5.52E-06
ENSG00000164308	ERAP2	0.01125989	0.205058764	-4.097090676	5.52E-06
ENSG00000236908	AC005865.1	0.013909937	0.268221104	-4.099157873	0.000575
ENSG00000223886	AC073073.1	0.090249354	1.740250962	-4.099157873	0.000575
ENSG00000253666	AP000424.1	0.056314624	1.085897837	-4.099157873	0.000575
ENSG00000230306	BANF1P2	0.158278223	3.052031043	-4.099157873	0.000575
ENSG00000283567	C19orf85	0.03234168	0.623634826	-4.099157873	0.000575
ENSG00000231527	CR769775.1	0.021538892	0.415327936	-4.099157873	0.000575
ENSG00000224316	GTF2IP5	0.057162039	1.102238297	-4.099157873	0.000575
ENSG00000214922	HLA-F-AS1	0.009998911	0.192805981	-4.099157873	0.000575
ENSG00000229950	TFAP2A-AS1	0.024695893	0.476203425	-4.099157873	0.000575
ENSG00000162078	ZG16B	0.015679344	0.302340036	-4.099157873	0.000575
ENSG00000182985	CADM1	0.223776127	3.855877529	-4.099404591	6.89E-17
ENSG00000275410	HNF1B	1.009242689	17.39620154	-4.101652763	6.70E-18
ENSG00000105559	PLEKHA4	0.358068002	6.207090919	-4.105748296	8.48E-16
ENSG00000204540	PSORS1C1	0.050596463	0.890461689	-4.108038311	1.02E-10
ENSG00000150782	IL18	0.731404389	12.70673097	-4.110413289	1.27E-16
ENSG00000116106	EPHA4	0.094036625	1.641432987	-4.110746992	3.40E-14
ENSG00000214944	ARHGEF28	0.650180222	11.31044882	-4.115393683	2.75E-18
ENSG00000137501	SYTL2	0.404566898	7.085439984	-4.124240434	7.84E-18
ENSG00000235961	PNMA6A	0.056748009	1.033462752	-4.124844939	1.95E-07
ENSG00000197635	DPP4	2.711174186	47.55132201	-4.127398606	1.70E-18
ENSG00000159708	LRRC36	0.052010768	0.93604736	-4.130438701	1.61E-09

ENSG00000205885	C1RL-AS1	0.047997728	0.859416684	-4.132887081	7.62E-11
ENSG00000118785	SPP1	42.07350212	740.7612027	-4.133392639	7.25E-19
ENSG00000198910	L1CAM	2.247333046	39.65877147	-4.136348008	1.31E-18
ENSG00000225298	LINC00113	0.136777253	2.564172289	-4.138764795	4.01E-06
ENSG00000213889	PPM1N	0.065010425	1.183933214	-4.138950008	1.21E-08
ENSG00000173557	C2orf70	0.100005068	1.803380661	-4.139014341	3.07E-10
ENSG00000280071	GATD3B	0.134531393	2.406774426	-4.139066789	1.20E-12
ENSG00000091656	ZFHX4	0.061265084	1.090043513	-4.141000941	3.43E-15
ENSG00000110328	GALNT18	0.472347251	8.404655654	-4.144432399	1.19E-16
ENSG00000188766	SPRED3	0.092634867	1.664113333	-4.149040722	1.34E-13
ENSG00000226330	AL606489.1	0.257934881	4.789469839	-4.152793701	1.17E-07
ENSG00000123342	MMP19	0.026831411	0.498219679	-4.152793701	1.17E-07
ENSG00000136531	SCN2A	0.005290414	0.09823517	-4.152793701	1.17E-07
ENSG00000042832	TG	0.011260901	0.209098306	-4.152793701	1.17E-07
ENSG00000075891	PAX2	0.066931685	1.21175198	-4.156229582	9.69E-13
ENSG00000231789	PIK3CD-AS2	0.311499633	5.672855712	-4.157315096	5.73E-11
ENSG00000135697	BCO1	0.242441182	4.3846471	-4.161889823	1.71E-14
ENSG00000189410	SH2D5	0.935283659	16.84056861	-4.164060787	6.07E-18
ENSG00000152953	STK32B	0.251152221	4.540211469	-4.166082771	3.95E-16
ENSG00000132837	DMGDH	0.047718444	0.877541218	-4.167269893	2.23E-10
ENSG00000163762	TM4SF18	0.055937685	1.028692896	-4.167269893	2.23E-10
ENSG00000175592	FOSL1	2.767981635	50.07681529	-4.171272945	3.17E-18
ENSG00000276115	AC026356.2	0.023265841	0.473551958	-4.176653707	0.000248
ENSG00000240057	AC078785.1	0.020871854	0.424824834	-4.176653707	0.000248
ENSG00000276952	AL121772.3	0.061996218	1.261868423	-4.176653707	0.000248
ENSG00000161609	CCDC155	0.007429845	0.151226763	-4.176653707	0.000248
ENSG00000232352	SEMA3B-AS1	0.123625594	2.516270169	-4.176653707	0.000248
ENSG00000245571	FAM111A-DT	0.031595804	0.609252322	-4.179268823	2.93E-06
ENSG00000132031	MATN3	0.455646352	8.312330858	-4.179543167	2.45E-16
ENSG00000183778	B3GALT5	0.131318198	2.394619137	-4.180195083	4.94E-17
ENSG00000135709	KIAA0513	0.288436403	5.257906597	-4.18072428	1.84E-17
ENSG00000106927	AMBP	0.348041481	6.382199163	-4.181849889	1.30E-14
ENSG00000196569	LAMA2	0.064545975	1.192761311	-4.192314958	1.65E-14
ENSG00000006747	SCIN	0.039545725	0.73558946	-4.196829286	2.57E-13
ENSG00000108846	ABCC3	0.647600599	11.9334181	-4.19802082	1.48E-18
ENSG00000198842	DUSP27	0.077668124	1.456049506	-4.202185109	1.08E-11
ENSG00000120068	HOXB8	2.220679733	41.09651811	-4.203770848	2.33E-18
ENSG00000092929	UNC13D	0.123976441	2.303984219	-4.203797601	1.36E-15
ENSG00000163453	IGFBP7	1.515534483	28.08711953	-4.204938823	8.71E-18
ENSG00000107105	ELAVL2	0.024388395	0.470274044	-4.207117539	7.11E-08
ENSG00000160469	BRSK1	0.217575896	4.06725581	-4.211124926	2.94E-15
ENSG00000285906	AC083855.2	0.713205485	13.34803938	-4.211281563	8.16E-15
ENSG00000099139	PCSK5	0.631468441	11.78533199	-4.216663406	7.95E-19

ENSG00000197408	CYP2B6	1.542676668	28.81960362	-4.217718539	1.16E-18
ENSG00000226124	FTCDNL1	0.030290287	0.600302832	-4.218666676	2.15E-06
ENSG00000105048	TNNT1	4.330597154	81.4437272	-4.227825363	4.99E-19
ENSG00000153029	MR1	0.151979603	2.868325851	-4.228525766	1.18E-16
ENSG00000241749	RPSAP52	0.39013443	7.412860983	-4.234177928	2.88E-15
ENSG00000132109	TRIM21	0.324925745	6.195828701	-4.236847477	1.37E-14
ENSG00000185551	NR2F2	0.388160249	7.380821317	-4.241562968	7.67E-18
ENSG00000128394	APOBEC3F	0.103110304	1.988244776	-4.242792516	6.57E-12
ENSG00000107738	VSIR	0.13852431	2.653664016	-4.244876439	5.16E-15
ENSG00000253522	MIR3142HG	0.017579071	0.376635439	-4.250198127	0.000164
ENSG00000227212	PFN1P6	0.10472544	2.243765512	-4.250198127	0.000164
ENSG00000250327	RPSAP70	0.04836279	1.036183379	-4.250198127	0.000164
ENSG00000260075	NSFP1	0.056812306	1.156355292	-4.257017171	1.58E-06
ENSG00000138496	PARP9	0.014025101	0.280458137	-4.259469857	4.35E-08
ENSG00000276851	AC002401.4	0.28669263	5.681768482	-4.260732917	3.48E-09
ENSG00000126895	AVPR2	0.241212638	4.690984362	-4.263438456	2.72E-14
ENSG00000122861	PLAU	1.048177409	20.34825307	-4.272008455	2.10E-18
ENSG00000074416	MGLL	1.608082163	31.21216252	-4.273751072	1.41E-19
ENSG00000058085	LAMC2	11.16322298	217.1863166	-4.277449708	7.76E-20
ENSG00000104833	TUBB4A	0.337562787	6.599525482	-4.278282134	1.50E-16
ENSG00000130208	APOC1	1.357772567	26.58151272	-4.282142191	1.66E-17
ENSG00000228065	LINC01515	0.037438433	0.744195258	-4.289028185	1.20E-12
ENSG00000157851	DPYSL5	0.141665605	2.794928649	-4.290347742	3.08E-16
ENSG00000257135	AC007249.2	0.047673076	0.995870939	-4.294374554	1.16E-06
ENSG00000285417	BX571818.1	0.684347459	13.54106002	-4.296720879	4.26E-17
ENSG00000122870	BICC1	1.138620138	22.4667397	-4.29688816	2.39E-19
ENSG00000124942	AHNAK	7.315942265	144.2791425	-4.297066453	5.52E-20
ENSG00000103187	COTL1	3.479031171	68.71357675	-4.298966754	8.65E-20
ENSG00000248874	C5orf17	0.168489721	3.384308616	-4.301637796	2.82E-12
ENSG00000170689	HOXB9	1.214222707	24.12456515	-4.30548514	1.21E-18
ENSG00000183780	SLC35F3	0.065391942	1.330984847	-4.307736788	2.32E-10
ENSG00000261087	AP003469.4	0.04437393	0.919030469	-4.309988758	2.69E-08
ENSG00000160678	S100A1	1.544544714	30.77279968	-4.310266914	4.33E-19
ENSG00000114251	WNT5A	0.039542809	0.804852673	-4.317625633	8.49E-12
ENSG00000267121	AC008105.3	0.011235668	0.25276299	-4.320174613	0.00011
ENSG00000249328	AC036214.1	0.01702055	0.382902469	-4.320174613	0.00011
ENSG00000184227	ACOT1	0.024393141	0.548759814	-4.320174613	0.00011
ENSG00000284630	AP000553.5	0.088716456	1.995807986	-4.320174613	0.00011
ENSG00000154975	CA10	0.009389989	0.211241699	-4.320174613	0.00011
ENSG00000172318	B3GALT1	0.065623009	1.335687974	-4.320731331	2.22E-12
ENSG00000125872	LRRN4	0.682971706	13.76815193	-4.324792092	6.09E-18
ENSG00000198915	RASGEF1A	0.378429078	7.643562897	-4.328379161	2.67E-18
ENSG00000006611	USH1C	0.25562062	5.17550778	-4.329179701	4.90E-17

ENSG00000235385	LINC02154	0.053674311	1.149983865	-4.330788966	8.61E-07
ENSG00000173572	NLRP13	0.023343827	0.500146614	-4.330788966	8.61E-07
ENSG00000067066	SP100	0.16557874	3.366635183	-4.337624561	3.18E-18
ENSG00000172159	FRMD3	0.24481146	4.982882216	-4.338028911	1.38E-17
ENSG00000117016	RIMS3	0.127060113	2.592363248	-4.338102581	2.50E-16
ENSG00000124225	PMEPA1	0.171038506	3.497243378	-4.341589976	1.87E-16
ENSG00000156510	HKDC1	1.550152904	31.58327812	-4.34300124	1.47E-19
ENSG00000143850	PLEKHA6	0.374757406	7.644072093	-4.343364553	6.93E-19
ENSG00000170439	METTL7B	1.304061212	26.66170013	-4.345365308	3.45E-18
ENSG00000180525	PRR26	0.02711287	0.566376692	-4.350931683	2.29E-11
ENSG00000089847	ANKRD24	0.028018854	0.600310084	-4.358798324	1.68E-08
ENSG00000013364	MVP	2.245432136	46.51982484	-4.367686735	4.03E-20
ENSG00000156113	KCNMA1	0.164121959	3.403323435	-4.368281717	1.12E-19
ENSG00000057019	DCBLD2	6.756685891	140.10611	-4.369406122	1.82E-20
ENSG00000251493	FOXD1	0.14337364	3.020614787	-4.372870478	3.73E-13
ENSG00000101049	SGK2	0.145088371	3.039471244	-4.374444397	5.68E-16
ENSG00000164749	HNF4G	0.252138592	5.281042479	-4.377876147	2.83E-17
ENSG00000275591	XKR5	0.058139168	1.236746959	-4.381227713	3.90E-12
ENSG00000244503	AC108751.5	0.031276535	0.737117278	-4.386913395	7.35E-05
ENSG00000121753	ADGRB2	0.0053875	0.126971207	-4.386913395	7.35E-05
ENSG00000280303	ERICD	0.020861433	0.491656856	-4.386913395	7.35E-05
ENSG00000144481	TRPM8	0.00528729	0.124609475	-4.386913395	7.35E-05
ENSG00000158125	XDH	0.006921559	0.163125507	-4.386913395	7.35E-05
ENSG00000197321	SVIL	0.639165595	13.50056119	-4.394962228	6.78E-20
ENSG00000241635	UGT1A1	0.046771268	1.012105511	-4.395983729	8.52E-11
ENSG00000005884	ITGA3	6.910244407	146.4460105	-4.400821955	1.11E-20
ENSG00000023171	GRAMD1B	0.067472974	1.439599832	-4.403282279	5.65E-17
ENSG00000175147	TMEM51-AS1	0.015922311	0.352510296	-4.406010452	1.05E-08
ENSG00000141665	FBXO15	0.033455125	0.731119046	-4.410180678	7.24E-11
ENSG00000167165	UGT1A6	0.532401496	11.38089414	-4.410711654	4.33E-19
ENSG00000164794	KCNV1	0.041911184	0.910784255	-4.412007273	2.67E-12
ENSG00000173193	PARP14	0.091573641	1.974780893	-4.41836064	5.98E-17
ENSG00000150995	ITPR1	0.056651255	1.224799787	-4.425706769	1.31E-18
ENSG00000151967	SCHIP1	0.016397168	0.368878572	-4.429049498	8.38E-09
ENSG00000212993	POU5F1B	0.016835395	0.387754683	-4.434822295	3.56E-07
ENSG00000250799	PRODH2	0.137703792	3.032287647	-4.436603805	1.64E-13
ENSG00000272384	AC016405.3	0.048587733	1.197153262	-4.450700878	4.96E-05
ENSG00000251127	AC091173.1	0.015194709	0.374382475	-4.450700878	4.96E-05
ENSG00000280157	AL359510.2	0.018579569	0.457782039	-4.450700878	4.96E-05
ENSG00000273001	AL731533.2	0.072293167	1.781231497	-4.450700878	4.96E-05
ENSG00000158008	EXTL1	0.00920993	0.226923475	-4.450700878	4.96E-05
ENSG00000230020	NHS-AS1	0.114167898	2.812983074	-4.450700878	4.96E-05
ENSG00000133321	RARRES3	0.032167399	0.792572598	-4.450700878	4.96E-05

ENSG00000274718	AL136964.1	0.041925201	0.958140403	-4.451726398	6.68E-09
ENSG00000186897	C1QL4	0.060529383	1.383312316	-4.451726398	6.68E-09
ENSG00000168077	SCARA3	1.73112634	38.23354724	-4.459434982	1.98E-20
ENSG00000162004	CCDC78	0.080608538	1.813408365	-4.46501204	3.61E-13
ENSG00000204528	PSORS1C3	0.634180534	14.26682976	-4.467444828	1.10E-13
ENSG00000139178	C1RL	0.108338823	2.428314554	-4.468160479	1.58E-15
ENSG00000173267	SNCG	0.435614162	9.763888672	-4.468160479	1.58E-15
ENSG00000273356	LINC02019	0.304262991	6.844846319	-4.475260063	4.86E-16
ENSG00000212978	AC016747.1	0.336003946	7.544508172	-4.47722156	1.51E-17
ENSG00000276547	PCDHGB5	0.043068904	0.987353958	-4.47914251	3.25E-11
ENSG00000204442	FAM155A	0.186739636	4.195720123	-4.480587069	1.61E-18
ENSG00000213988	ZNF90	0.033315089	0.776239109	-4.493871385	2.34E-10
ENSG00000104267	CA2	1.574581282	35.67555457	-4.495115535	5.56E-20
ENSG00000122176	FMOD	0.45459032	10.31881435	-4.495206757	1.44E-18
ENSG00000124143	ARHGAP40	0.039235165	0.92468421	-4.496038087	4.26E-09
ENSG00000099250	NRP1	0.010879739	0.25641087	-4.496038087	4.26E-09
ENSG00000233817	AL162727.1	0.027463326	0.661958754	-4.500230855	2.01E-07
ENSG00000282221	AC119427.1	0.45092213	10.46619758	-4.502785849	3.70E-12
ENSG00000035862	TIMP2	2.358915914	53.69508523	-4.503477434	4.75E-21
ENSG00000122863	CHST3	0.53355884	12.1958635	-4.508044377	3.63E-20
ENSG00000128342	LIF	0.536925671	12.33833596	-4.514236654	1.97E-19
ENSG00000184232	OAF	2.999711201	69.31518697	-4.524937816	4.69E-21
ENSG00000184489	PTP4A3	0.348575189	8.121776039	-4.530290719	8.70E-18
ENSG00000226833	AC092164.1	0.058116065	1.431921843	-4.531855678	1.52E-07
ENSG00000128536	CDHR3	0.047168563	1.111656479	-4.532079078	1.37E-13
ENSG00000022556	NLRP2	0.403756138	9.458895089	-4.542639222	7.11E-20
ENSG00000198053	SIRPA	1.896340504	44.37449186	-4.54310886	3.51E-21
ENSG00000120093	HOXB3	0.622074173	14.68489757	-4.554940144	9.59E-21
ENSG00000178445	GLDC	1.467796346	34.77291855	-4.561115579	2.05E-21
ENSG00000182795	C1orf116	0.440486395	10.45449141	-4.561252651	6.26E-20
ENSG00000187800	PEAR1	0.083117533	1.991270887	-4.561710912	1.69E-15
ENSG00000188000	OR7D2	0.02415344	0.608053969	-4.562802109	1.15E-07
ENSG00000233101	HOXB-AS3	0.698972517	16.70719764	-4.56896874	9.75E-19
ENSG00000262823	AC127521.1	0.039457461	1.056730924	-4.57039149	2.30E-05
ENSG00000131400	NAPSA	0.011668654	0.312504342	-4.57039149	2.30E-05
ENSG00000249740	OSMR-AS1	0.01971942	0.528116116	-4.57039149	2.30E-05
ENSG00000134121	CHL1	0.021137569	0.517034627	-4.578381628	1.46E-12
ENSG00000180543	TSPYL5	0.846688047	20.4429221	-4.587109877	1.00E-20
ENSG00000115641	FHL2	0.769366887	18.6726938	-4.595094807	4.43E-21
ENSG00000251169	LINC01843	0.18873284	4.731061671	-4.607901072	6.09E-12
ENSG00000261786	AC006058.1	0.189671476	4.708652853	-4.621434518	2.92E-18
ENSG00000119673	ACOT2	0.349230679	8.698224702	-4.625003972	6.83E-18
ENSG00000102524	TNFSF13B	0.01365984	0.380464587	-4.626707951	1.58E-05

ENSG00000249835	VCAN-AS1	0.047645896	1.327070891	-4.626707951	1.58E-05
ENSG00000026508	CD44	2.20121277	54.79052663	-4.632659891	4.51E-22
ENSG00000276170	AC244153.1	0.703120566	17.61113212	-4.633527787	4.31E-18
ENSG00000139117	CPNE8	0.597165577	14.92968552	-4.636938233	7.21E-21
ENSG00000153822	KCNJ16	0.243858309	6.107271372	-4.637032406	1.67E-19
ENSG00000144837	PLA1A	0.246689764	6.220488338	-4.638014261	1.40E-16
ENSG00000131080	EDA2R	0.070051049	1.788523733	-4.640167511	6.75E-13
ENSG00000265735	RN7SL5P	0.390518231	10.1797754	-4.64121556	9.42E-10
ENSG00000135596	MICAL1	0.201347127	5.081527876	-4.647698587	2.13E-19
ENSG00000144619	CNTN4	0.151197355	3.828424905	-4.652279685	2.27E-19
ENSG00000185567	AHNAK2	1.484784809	37.60903502	-4.657928441	2.64E-22
ENSG00000167653	PSCA	0.071104	1.878883168	-4.660812132	7.65E-10
ENSG00000105976	MET	9.97888091	253.5644214	-4.662667135	1.70E-22
ENSG00000176165	FOXG1	0.116980545	2.999246134	-4.663824239	3.03E-17
ENSG00000234745	HLA-B	0.150652232	3.887980111	-4.668994145	3.85E-16
ENSG00000102854	MSLN	0.473350809	12.22243057	-4.679761788	2.99E-19
ENSG00000261308	FIGNL2	0.02520739	0.675092328	-4.680146082	6.22E-10
ENSG00000254290	AC124067.4	0.035128584	0.959612955	-4.680348258	3.88E-08
ENSG00000279148	AC126474.1	0.064633335	1.765598778	-4.680348258	3.88E-08
ENSG00000165887	ANKRD2	0.057595384	1.573341985	-4.680348258	3.88E-08
ENSG00000184995	IFNE	0.028329119	0.819392741	-4.680908407	1.09E-05
ENSG00000173269	MMRN2	0.007833793	0.226584982	-4.680908407	1.09E-05
ENSG00000164342	TLR3	0.006214374	0.179744838	-4.680908407	1.09E-05
ENSG00000008513	ST3GAL1	0.3823567	9.891173357	-4.686385214	2.67E-21
ENSG00000069424	KCNAB2	0.070124524	1.826395473	-4.687233342	1.31E-17
ENSG00000169903	TM4SF4	0.859140109	22.29789159	-4.689249833	2.23E-20
ENSG00000123700	KCNJ2	0.284713489	7.408348856	-4.692308123	6.25E-20
ENSG00000067798	NAV3	0.012600211	0.337452861	-4.694523795	2.41E-11
ENSG00000171992	SYNPO	0.0162938	0.436372801	-4.694523795	2.41E-11
ENSG00000163840	DTX3L	0.078329918	2.059652851	-4.69594901	2.46E-16
ENSG00000113249	HAVCR1	1.398676176	36.48059172	-4.697953219	2.94E-21
ENSG00000089327	FXYD5	0.879546742	22.95736459	-4.698656238	5.59E-21
ENSG00000142227	EMP3	3.534342266	92.2651992	-4.700446394	5.91E-22
ENSG00000169908	TM4SF1	15.57271297	407.993646	-4.706766518	8.45E-23
ENSG00000274922	AL139384.1	0.027427273	0.763925941	-4.708297021	2.30E-08
ENSG00000254300	LINC01111	0.050253098	1.39968872	-4.708297021	2.30E-08
ENSG00000163449	TMEM169	0.115174892	3.059882648	-4.709217895	4.85E-16
ENSG00000198353	HOXC4	0.107338773	2.874695906	-4.713239824	4.63E-14
ENSG00000100360	IFT27	0.146084732	3.862069831	-4.713555659	2.15E-19
ENSG00000196581	AJAP1	0.111158394	2.937297967	-4.713825017	8.82E-20
ENSG00000104783	KCNN4	0.159392858	4.255648919	-4.720444767	4.38E-17
ENSG00000026025	VIM	9.761820117	259.466425	-4.727548231	6.10E-23
ENSG00000241158	ADAMTS9-AS1	0.00601316	0.180366587	-4.733146126	7.53E-06

ENSG00000248636	AC002070.1	0.206246055	5.656149665	-4.737408368	1.26E-12
ENSG00000105255	FSD1	0.188041244	5.07919706	-4.738173407	1.83E-17
ENSG00000182963	GJC1	0.545237406	14.67982291	-4.744663346	4.62E-22
ENSG00000227906	SNAP25-AS1	0.038405745	1.061478444	-4.748625071	1.10E-12
ENSG00000128335	APOL2	0.239870555	6.526884603	-4.752570011	9.78E-19
ENSG00000244128	LINC01322	0.359444738	9.843073593	-4.759542112	4.62E-18
ENSG00000105472	CLEC11A	0.36978275	10.14712023	-4.759958588	2.35E-17
ENSG00000171234	UGT2B7	0.832379497	22.72833151	-4.762568552	6.46E-21
ENSG00000225953	SATB2-AS1	0.018695951	0.540762547	-4.762620859	1.37E-08
ENSG00000161249	DMKN	1.257380571	34.44427225	-4.770413072	1.04E-22
ENSG00000147573	TRIM55	0.135623014	3.777478647	-4.777356953	1.89E-16
ENSG00000267649	AC010327.4	0.026496798	0.823164576	-4.783558308	5.25E-06
ENSG00000253616	AC107959.3	0.04742957	1.473473935	-4.783558308	5.25E-06
ENSG00000186466	AQP7P1	0.039986077	1.242230179	-4.783558308	5.25E-06
ENSG00000198520	ARMH1	0.012700745	0.394568552	-4.783558308	5.25E-06
ENSG00000002746	HECW1	0.002992155	0.092956	-4.783558308	5.25E-06
ENSG00000278709	NKILA	0.016083699	0.499665334	-4.783558308	5.25E-06
ENSG00000203706	SERTAD4-AS1	0.08323795	2.452163056	-4.789034474	1.06E-08
ENSG00000108622	ICAM2	0.193272205	5.493593136	-4.812561234	3.40E-18
ENSG00000160801	PTH1R	0.106054444	3.067523586	-4.824197358	1.08E-14
ENSG00000072840	EVC	0.379974021	10.83382778	-4.826179708	6.83E-22
ENSG00000184304	PRKD1	0.10472544	3.020453574	-4.831765901	8.43E-18
ENSG00000054392	HHAT	0.100672672	2.911860968	-4.838449883	1.40E-18
ENSG00000157168	NRG1	0.32460449	9.349359689	-4.841514483	1.89E-22
ENSG00000176697	BDNF	0.037790459	1.110403025	-4.846903024	7.98E-15
ENSG00000119946	CNNM1	0.16289354	4.732480382	-4.848864432	5.34E-20
ENSG00000162105	SHANK2	0.033383849	0.977516911	-4.85540389	1.82E-18
ENSG00000105974	CAV1	4.483593197	130.275818	-4.855802687	1.40E-23
ENSG00000102452	NALCN	0.013715137	0.416287239	-4.860445146	7.09E-11
ENSG00000134954	ETS1	0.549424027	16.06688641	-4.863557509	1.17E-22
ENSG00000136546	SCN7A	0.022989355	0.689572741	-4.866574694	2.52E-13
ENSG00000075223	SEMA3C	2.126604785	62.29558278	-4.867385327	1.40E-23
ENSG00000162572	SCNN1D	0.031144435	0.956429438	-4.877295139	5.87E-11
ENSG00000283033	AC022596.2	0.01357552	0.450830663	-4.879387158	2.59E-06
ENSG00000108932	SLC16A6	0.061736692	1.862833689	-4.88098811	2.74E-14
ENSG00000206190	ATP10A	0.044722209	1.346779624	-4.887962663	3.26E-16
ENSG00000184500	PROS1	0.821279065	24.73318715	-4.906193211	3.77E-23
ENSG00000272702	AC010913.1	0.028917267	0.929338172	-4.914301643	3.10E-09
ENSG00000167600	CYP2S1	0.247165158	7.538396322	-4.915598858	2.70E-19
ENSG00000089250	NOS1	0.143395507	4.369465119	-4.920831172	5.36E-22
ENSG00000284879	AC133644.3	0.010856184	0.372153781	-4.925015696	1.83E-06
ENSG00000141433	ADCYAP1	0.010981722	0.376457267	-4.925015696	1.83E-06
ENSG00000127325	BEST3	0.006088511	0.208716291	-4.925015696	1.83E-06

ENSG00000173482	PTPRM	0.09707731	2.979686603	-4.927514896	2.64E-20
ENSG00000261175	LINC02188	0.042930258	1.341360675	-4.931284426	1.27E-14
ENSG00000178726	THBD	0.071184754	2.222359912	-4.934312461	2.47E-15
ENSG00000024422	EHD2	0.564524316	17.36667643	-4.935189806	2.33E-22
ENSG00000230499	AC108463.2	0.202841994	6.627549611	-4.938100825	2.44E-09
ENSG00000100292	HMOX1	0.469109011	14.51776928	-4.940537476	7.92E-21
ENSG00000204876	AC021218.1	0.18124604	5.648343465	-4.940960614	7.28E-18
ENSG00000261780	LINC02582	1.285706177	39.90312586	-4.948739592	7.80E-23
ENSG00000166401	SERPINB8	0.099692623	3.119992652	-4.950538436	7.65E-19
ENSG00000198467	TPM2	2.032666632	63.23066612	-4.953481615	8.99E-24
ENSG00000142623	PADI1	0.102269102	3.213668964	-4.954279065	2.74E-18
ENSG00000238266	LINC00707	0.038245607	1.206369096	-4.954786434	1.29E-16
ENSG00000038427	VCAN	2.422998291	75.80242789	-4.962612219	1.76E-24
ENSG00000227051	C14orf132	0.075240683	2.383528295	-4.968063212	5.93E-19
ENSG00000116299	KIAA1324	0.004493059	0.158836884	-4.96924526	1.30E-06
ENSG00000226476	LINC01748	0.023603154	0.758554129	-4.971898744	7.43E-15
ENSG00000101265	RASSF2	0.15962492	5.067816551	-4.975875578	1.72E-20
ENSG00000086289	EPDR1	0.948342982	30.29012932	-4.989965421	5.00E-23
ENSG00000106366	SERPINE1	0.130988874	4.237762643	-4.993300625	7.83E-18
ENSG00000182132	KCNIP1	1.359180583	43.5608895	-4.99601315	8.64E-24
ENSG00000164867	NOS3	0.119326837	3.849944304	-4.996715918	7.80E-20
ENSG00000214820	MPRIPP1	0.038370478	1.397563036	-5.01215909	9.27E-07
ENSG00000139973	SYT16	0.002742907	0.099904565	-5.01215909	9.27E-07
ENSG00000280435	AC006058.4	0.150127368	4.985593224	-5.019173222	3.97E-15
ENSG00000135100	HNF1A	0.058173857	1.940803617	-5.030016568	6.15E-16
ENSG00000135919	SERPINE2	0.272585327	8.961686772	-5.03132379	3.67E-23
ENSG00000065675	PRKCQ	0.063407361	2.146456228	-5.04093061	2.39E-14
ENSG00000182742	HOXB4	0.645077494	21.36286337	-5.041821326	3.12E-23
ENSG00000173210	ABLIM3	0.063617987	2.131282208	-5.045259261	1.53E-18
ENSG00000280623	PCAT14	0.017017084	0.601581358	-5.051541406	7.62E-10
ENSG00000039139	DNAH5	0.081161422	2.711599133	-5.052014977	5.26E-22
ENSG00000235142	LINC02532	0.006190437	0.232105077	-5.053833208	6.64E-07
ENSG00000225706	PTPRD-AS1	0.016754391	0.628191367	-5.053833208	6.64E-07
ENSG00000164690	SHH	0.058058387	1.972491678	-5.056233702	4.30E-16
ENSG00000259120	SMIM6	0.269335318	9.150472445	-5.056233702	4.30E-16
ENSG00000234155	LINC02535	0.099766297	3.420023644	-5.064947627	2.15E-15
ENSG00000146678	IGFBP1	0.202228437	6.905381791	-5.066694057	8.67E-17
ENSG00000153707	PTPRD	0.108533638	3.692506523	-5.077610352	6.16E-22
ENSG00000147642	SYBU	0.101132213	3.460474833	-5.081504862	2.12E-20
ENSG00000119771	KLHL29	0.064163369	2.211672632	-5.092091148	1.81E-20
ENSG00000268279	AC090004.1	0.031299963	1.207095424	-5.094337237	4.78E-07
ENSG00000135318	NT5E	6.895106368	236.9789104	-5.09824341	2.05E-25
ENSG00000138685	FGF2	0.135687073	4.691043039	-5.098800789	2.52E-21

ENSG00000198758	EPS8L3	0.048070694	1.712250567	-5.105517734	1.42E-13
ENSG00000125798	FOXA2	0.064211219	2.287166816	-5.105517734	1.42E-13
ENSG00000166473	PKD1L2	0.008968278	0.321846775	-5.116314711	1.24E-13
ENSG00000179674	ARL14	0.193750158	6.883973161	-5.116577812	1.07E-15
ENSG00000177494	ZBED2	0.614757822	21.50024465	-5.118323508	1.32E-22
ENSG00000139910	NOVA1	0.041594118	1.474874912	-5.125532711	1.10E-18
ENSG00000204267	TAP2	0.065391942	2.325720891	-5.129883149	1.04E-18
ENSG00000273055	AC005046.1	0.066856721	2.64997682	-5.13373509	3.45E-07
ENSG00000261838	AC092718.6	0.08424486	3.339184501	-5.13373509	3.45E-07
ENSG00000175294	CATSPER1	0.031101936	1.166140133	-5.136279562	3.11E-10
ENSG00000167779	IGFBP6	2.825019331	100.9664475	-5.152926587	1.17E-24
ENSG00000080293	SCTR	0.036589712	1.371899535	-5.16490938	2.08E-12
ENSG00000084636	COL16A1	0.004508086	0.183514795	-5.172085584	2.51E-07
ENSG00000257718	CPNE8-AS1	0.07944002	3.233837708	-5.172085584	2.51E-07
ENSG00000151025	GPR158	0.041858703	1.54142982	-5.175578707	1.74E-17
ENSG00000259674	AC092868.1	0.156304679	5.916322853	-5.178568664	1.77E-12
ENSG00000254166	CASC19	0.016933945	0.624845452	-5.180966789	4.57E-18
ENSG00000237517	DGCR5	0.088341334	3.264963441	-5.188260222	8.09E-20
ENSG00000072832	CRMP1	0.086111181	3.190227651	-5.191739776	7.68E-20
ENSG00000142661	MYOM3	0.135461803	5.020976939	-5.201425946	7.10E-23
ENSG00000267978	MAGEA9B	0.152263485	5.732289434	-5.204255731	5.10E-17
ENSG00000184371	CSF1	0.038561693	1.478884982	-5.220836224	2.00E-15
ENSG00000285106	AC016831.6	0.007537058	0.302780857	-5.235649401	1.06E-10
ENSG00000246640	PICART1	0.082482137	3.216300117	-5.244799036	1.45E-15
ENSG00000271461	AL109809.4	0.019123776	0.819462187	-5.245857379	1.33E-07
ENSG00000276649	AL117335.1	0.026002147	1.114203409	-5.245857379	1.33E-07
ENSG00000228741	SPATA13	0.024279751	1.040397954	-5.245857379	1.33E-07
ENSG00000146592	CREB5	0.175388649	6.710250422	-5.249485896	1.99E-24
ENSG00000171303	KCNK3	0.040288077	1.58969146	-5.267768551	1.24E-16
ENSG00000148677	ANKRD1	0.211144269	8.278576371	-5.27049477	1.26E-19
ENSG00000128510	CPA4	0.010557213	0.463690753	-5.281375254	9.80E-08
ENSG00000172260	NEGR1	0.003116922	0.136900492	-5.281375254	9.80E-08
ENSG00000260328	AC104024.2	0.06789966	2.720410727	-5.283873248	8.59E-16
ENSG00000180730	SHISA2	0.029220595	1.236463858	-5.310493893	4.63E-11
ENSG00000284948	AC107959.4	0.03364368	1.513728762	-5.316039679	7.22E-08
ENSG00000187583	PLEKHN1	0.014749541	0.663625529	-5.316039679	7.22E-08
ENSG00000279041	AC102945.2	0.117539946	4.942201229	-5.344655746	5.77E-15
ENSG00000237914	SIRPG-AS1	0.028185802	1.222871123	-5.346508717	3.09E-11
ENSG00000137731	FXYD2	0.104556981	4.328301873	-5.353962164	1.54E-21
ENSG00000081842	PCDHA6	0.040879293	1.722500698	-5.362458905	3.05E-17
ENSG00000226380	LINC-PINT	0.006312955	0.297564189	-5.382965641	3.96E-08
ENSG00000229124	VIM-AS1	0.020976632	0.988743658	-5.382965641	3.96E-08
ENSG00000197343	ZNF655	0.13429439	5.658663912	-5.385715844	6.98E-24

ENSG00000168062	BATF2	0.029384986	1.322117526	-5.398899346	1.40E-11
ENSG00000148655	LRMDA	0.012739467	0.573186318	-5.398899346	1.40E-11
ENSG00000237943	PRKCQ-AS1	0.053681206	2.334767153	-5.402241237	1.72E-16
ENSG00000267056	AC005336.1	0.134466455	5.905995174	-5.40753962	2.52E-15
ENSG00000111452	ADGRD1	0.02271257	0.98236774	-5.407587038	5.90E-19
ENSG00000186481	ANKRD20A5P	0.006630506	0.319635114	-5.415299268	2.95E-08
ENSG00000250942	ENPP7P11	0.066643462	3.212664256	-5.415299268	2.95E-08
ENSG00000101000	PROCR	0.320289422	13.90929556	-5.422046617	1.05E-21
ENSG00000136155	SCEL	1.023519959	44.68860547	-5.441973316	6.56E-27
ENSG00000177103	DSCAML1	0.053456867	2.379731004	-5.451640598	8.00E-20
ENSG00000079263	SP140	0.022457247	1.05853316	-5.494718559	3.01E-14
ENSG00000113763	UNC5A	0.009586018	0.492918067	-5.508167056	1.24E-08
ENSG00000279289	AL136164.3	0.042016542	1.980469951	-5.509346773	5.79E-16
ENSG00000181449	SOX2	0.049883149	2.386891501	-5.516393927	2.28E-14
ENSG00000253368	TRNP1	4.5253474	209.113552	-5.524706072	4.79E-28
ENSG00000176692	FOXC2	0.115270209	5.464187991	-5.539754963	8.08E-20
ENSG00000177335	C8orf31	0.071330575	3.423331963	-5.544179738	2.20E-17
ENSG00000187720	THSD4	0.108177274	5.110293901	-5.552940743	2.97E-26
ENSG00000198223	CSF2RA	0.054550197	2.746562314	-5.613305334	8.36E-18
ENSG00000144057	ST6GAL2	0.027105248	1.399573481	-5.649659255	4.60E-18
ENSG00000224649	AF124730.1	0.127784253	7.255184905	-5.65078303	2.40E-09
ENSG00000164220	F2RL2	0.0121859	0.704931055	-5.677689272	1.84E-09
ENSG00000144596	GRIP2	0.007031628	0.384168148	-5.678641323	5.29E-13
ENSG00000168394	TAP1	0.09565868	5.123770464	-5.722092464	4.61E-23
ENSG00000231924	PSG1	0.008422788	0.514311218	-5.755522157	8.49E-10
ENSG00000124564	SLC17A3	0.011626447	0.709932652	-5.755522157	8.49E-10
ENSG00000142619	PADI3	0.393089847	22.06573463	-5.800163646	4.42E-27
ENSG00000269707	AC018730.2	0.094323817	5.9616799	-5.805171681	5.13E-10
ENSG00000185404	SP140L	0.02958262	1.727143467	-5.81790867	7.64E-18
ENSG00000165806	CASP7	0.035531846	2.106201317	-5.825093458	3.51E-16
ENSG00000179314	WSCD1	0.333768315	19.27649577	-5.844087857	6.73E-29
ENSG00000234779	BNC2-AS1	0.097629558	6.693550947	-5.922298131	1.52E-10
ENSG00000167601	AXL	2.067382604	126.9156968	-5.934646064	4.29E-31
ENSG00000131910	NR0B2	0.036146584	2.594402397	-5.98826552	7.50E-11
ENSG00000214049	UCA1	0.080511466	5.304300579	-6.014525762	5.19E-23
ENSG00000100033	PRODH	0.033329325	2.26467937	-6.055841031	1.43E-22
ENSG00000168874	ATOH8	0.013754263	0.979837923	-6.061100269	3.77E-15
ENSG00000166897	ELFN2	0.042822712	2.910469442	-6.061932219	1.71E-24
ENSG00000140323	DISP2	0.003276262	0.256210436	-6.111787293	1.94E-11
ENSG00000255441	AC008750.2	0	0.400863779	-6.237329925	0.007243
ENSG00000229618	AC011287.1	0	0.074757487	-6.237329925	0.007243
ENSG00000259476	AC018904.2	0	0.229293169	-6.237329925	0.007243
ENSG00000235240	AC026185.1	0	2.315333895	-6.237329925	0.007243

ENSG00000261527	AC026464.5	0	0.557989055	-6.237329925	0.007243
ENSG00000261634	AC026992.2	0	0.083582593	-6.237329925	0.007243
ENSG00000264187	AC055811.2	0	0.237961074	-6.237329925	0.007243
ENSG00000223711	AC069213.1	0	0.387001054	-6.237329925	0.007243
ENSG00000262136	AC092115.3	0	0.680520435	-6.237329925	0.007243
ENSG00000272787	AC253536.6	0	0.60764419	-6.237329925	0.007243
ENSG00000228412	AL022068.1	0	0.065094215	-6.237329925	0.007243
ENSG00000229771	AL035665.1	0	0.241382922	-6.237329925	0.007243
ENSG00000269846	AL136172.1	0	0.289001505	-6.237329925	0.007243
ENSG00000231187	AL356056.2	0	0.406526839	-6.237329925	0.007243
ENSG00000237781	AL356356.1	0	0.605816688	-6.237329925	0.007243
ENSG00000126353	CCR7	0	0.160633213	-6.237329925	0.007243
ENSG00000237990	CNTN4-AS1	0	0.193873002	-6.237329925	0.007243
ENSG00000240216	CPHL1P	0	0.102406736	-6.237329925	0.007243
ENSG00000080166	DCT	0	0.053409532	-6.237329925	0.007243
ENSG00000154252	GAL3ST2	0	0.294064305	-6.237329925	0.007243
ENSG00000084734	GCKR	0	0.128057247	-6.237329925	0.007243
ENSG00000231948	HS1BP3-IT1	0	0.671446829	-6.237329925	0.007243
ENSG00000164509	IL31RA	0	0.088039357	-6.237329925	0.007243
ENSG00000214659	KRT8P26	0	0.315727349	-6.237329925	0.007243
ENSG00000243795	LINC02044	0	0.161018424	-6.237329925	0.007243
ENSG00000226101	LINC02097	0	0.195282645	-6.237329925	0.007243
ENSG00000225613	LINCMD1	0	0.872008869	-6.237329925	0.007243
ENSG00000162896	PIGR	0	0.092063094	-6.237329925	0.007243
ENSG00000212237	RNA5SP18	0	3.32948841	-6.237329925	0.007243
ENSG00000252516	RNA5SP82	0	4.379001061	-6.237329925	0.007243
ENSG00000196209	SIRPB2	0	0.080029419	-6.237329925	0.007243
ENSG00000154027	AK5	0.006039233	0.549915503	-6.331016879	1.31E-12
ENSG00000261115	TMEM178B	0.054898302	4.557806619	-6.35779509	1.58E-28
ENSG00000184156	KCNQ3	0.006608485	0.584051489	-6.371885923	5.39E-17
ENSG00000225676	AC002378.1	0	1.502118187	-6.387419471	0.004174
ENSG00000267405	AC005180.1	0	0.76257448	-6.387419471	0.004174
ENSG00000234350	AC007405.1	0	0.127967759	-6.387419471	0.004174
ENSG00000249655	AC008434.1	0	0.815357413	-6.387419471	0.004174
ENSG00000261723	AC008870.4	0	0.71392539	-6.387419471	0.004174
ENSG00000259760	AC015660.2	0	2.260763735	-6.387419471	0.004174
ENSG00000248898	AC022126.1	0	0.754858718	-6.387419471	0.004174
ENSG00000284730	AC068987.5	0	0.453527072	-6.387419471	0.004174
ENSG00000280362	AC084759.3	0	0.11349676	-6.387419471	0.004174
ENSG00000279568	AC093525.9	0	0.256375269	-6.387419471	0.004174
ENSG00000244998	AC100803.1	0	0.324370449	-6.387419471	0.004174
ENSG00000282381	AC104073.4	0	0.74854719	-6.387419471	0.004174
ENSG00000264647	AC104564.4	0	1.610184243	-6.387419471	0.004174

ENSG00000274244	AC243585.1	0	0.737448467	-6.387419471	0.004174
ENSG00000277235	AL035420.3	0	1.394489781	-6.387419471	0.004174
ENSG00000232946	AL354707.3	0	1.35236018	-6.387419471	0.004174
ENSG00000255521	AL356215.1	0	0.368724234	-6.387419471	0.004174
ENSG00000273167	AL359736.1	0	0.107681313	-6.387419471	0.004174
ENSG00000213863	AL731661.1	0	0.590542506	-6.387419471	0.004174
ENSG00000234460	AL772337.3	0	0.592104788	-6.387419471	0.004174
ENSG00000273199	AP000692.2	0	0.509250534	-6.387419471	0.004174
ENSG00000271882	AP001330.5	0	1.089127055	-6.387419471	0.004174
ENSG00000165269	AQP7	0	0.085214396	-6.387419471	0.004174
ENSG00000238279	BX470102.1	0	0.842996647	-6.387419471	0.004174
ENSG00000214711	CAPN14	0	0.111517494	-6.387419471	0.004174
ENSG00000186204	CYP4F12	0	0.083653751	-6.387419471	0.004174
ENSG00000198889	DCAF12L1	0	0.132356954	-6.387419471	0.004174
ENSG00000205879	FAM90A2P	0	0.320882595	-6.387419471	0.004174
ENSG00000226564	FTH1P20	0	0.816845291	-6.387419471	0.004174
ENSG00000270604	HCG17	0	0.589764453	-6.387419471	0.004174
ENSG00000185614	INKA1	0	0.430414634	-6.387419471	0.004174
ENSG00000026559	KCNG1	0	0.082330554	-6.387419471	0.004174
ENSG00000183775	KCTD16	0	0.031316022	-6.387419471	0.004174
ENSG00000207696	MIR659	0	4.614754841	-6.387419471	0.004174
ENSG00000199179	MIRLET7I	0	5.328943091	-6.387419471	0.004174
ENSG00000124232	RBPJL	0	0.162009128	-6.387419471	0.004174
ENSG00000283418	RF00026	0	5.086718405	-6.387419471	0.004174
ENSG00000185002	RFX6	0	0.109365067	-6.387419471	0.004174
ENSG00000244486	SCARF2	0	0.133981209	-6.387419471	0.004174
ENSG00000249167	SEMA6A-AS2	0	0.928695476	-6.387419471	0.004174
ENSG00000214510	SPINK13	0	0.38128724	-6.387419471	0.004174
ENSG00000101307	SIRPB1	0.006860195	0.676113482	-6.445031985	3.42E-13
ENSG00000101017	CD40	0.033071192	3.117653132	-6.464929307	1.35E-17
ENSG00000261757	AC005592.1	0	0.300240452	-6.523355476	0.002435
ENSG00000228323	AC008440.1	0	1.121627202	-6.523355476	0.002435
ENSG00000251423	AC016576.1	0	0.135832922	-6.523355476	0.002435
ENSG00000285155	AC092153.1	0	0.225558562	-6.523355476	0.002435
ENSG00000223734	AC098828.2	0	0.859326949	-6.523355476	0.002435
ENSG00000275966	AC110285.6	0	1.0028398	-6.523355476	0.002435
ENSG00000259039	AL161804.1	0	0.344331707	-6.523355476	0.002435
ENSG00000255129	AP000880.1	0	0.513981567	-6.523355476	0.002435
ENSG00000255345	AP002957.1	0	0.20524983	-6.523355476	0.002435
ENSG00000237438	CECR7	0	0.110575868	-6.523355476	0.002435
ENSG00000001626	CFTR	0	0.052701952	-6.523355476	0.002435
ENSG00000184697	CLDN6	0	0.283147983	-6.523355476	0.002435
ENSG00000123977	DAW1	0	0.167880785	-6.523355476	0.002435

ENSG00000243902	ELFN2	0	0.232480803	-6.523355476	0.002435
ENSG00000206262	FOXL2NB	0	0.107815709	-6.523355476	0.002435
ENSG00000270276	HIST2H4B	0	0.25525886	-6.523355476	0.002435
ENSG00000244482	LILRA6	0	0.181160538	-6.523355476	0.002435
ENSG00000280924	LINC00628	0	0.381997162	-6.523355476	0.002435
ENSG00000235597	LINC01102	0	0.096585787	-6.523355476	0.002435
ENSG00000230945	LINC01507	0	1.104023187	-6.523355476	0.002435
ENSG00000188582	PAQR9	0	0.118108501	-6.523355476	0.002435
ENSG00000147223	RIPPLY1	0	0.413776758	-6.523355476	0.002435
ENSG00000243422	RPL23AP49	0	0.432683956	-6.523355476	0.002435
ENSG00000144834	TAGLN3	0	0.274927047	-6.523355476	0.002435
ENSG00000131044	TTLL9	0	0.079739974	-6.523355476	0.002435
ENSG00000237862	Z94160.1	0	0.250582362	-6.523355476	0.002435
ENSG00000261251	Z97055.2	0	0.248558476	-6.523355476	0.002435
ENSG00000180767	CHST13	0.046713752	4.603920872	-6.529016586	5.61E-18
ENSG00000242078	AC084864.1	0	1.761172012	-6.647578789	0.001437
ENSG00000260750	AC092720.1	0	0.396133823	-6.647578789	0.001437
ENSG00000262165	AC233723.1	0	0.825126672	-6.647578789	0.001437
ENSG00000234667	ACTBP13	0	0.135680087	-6.647578789	0.001437
ENSG00000221963	APOL6	0	0.053073556	-6.647578789	0.001437
ENSG00000198312	BMS1P9	0	0.606957586	-6.647578789	0.001437
ENSG00000259845	HERC2P10	0	0.224470315	-6.647578789	0.001437
ENSG00000233155	HMGA1P8	0	1.673387737	-6.647578789	0.001437
ENSG00000248925	HRAT5	0	1.029037286	-6.647578789	0.001437
ENSG00000259293	LIPC-AS1	0	0.240769818	-6.647578789	0.001437
ENSG00000247317	LY6E-DT	0	0.139412786	-6.647578789	0.001437
ENSG00000187045	TMPRSS6	0	0.126927567	-6.647578789	0.001437
ENSG00000233916	ZDHHC20P1	0	1.2698758	-6.647578789	0.001437
ENSG00000284294	AC007326.5	0	0.34971189	-6.761948676	0.000858
ENSG00000249923	AC007663.2	0	0.261185182	-6.761948676	0.000858
ENSG00000232324	AC008440.3	0	1.048505559	-6.761948676	0.000858
ENSG00000257403	AC008740.1	0	2.282041512	-6.761948676	0.000858
ENSG00000272264	AC009686.2	0	1.008527878	-6.761948676	0.000858
ENSG00000258976	AC013451.2	0	0.811604722	-6.761948676	0.000858
ENSG00000272043	AC016405.2	0	1.865130082	-6.761948676	0.000858
ENSG00000259275	AC087477.2	0	0.109734223	-6.761948676	0.000858
ENSG00000253483	AC104561.2	0	1.121234269	-6.761948676	0.000858
ENSG00000238181	AHCYP2	0	0.44763122	-6.761948676	0.000858
ENSG00000229109	AL137847.1	0	1.310631949	-6.761948676	0.000858
ENSG00000258592	AL391152.1	0	0.36900481	-6.761948676	0.000858
ENSG00000232692	AP001596.1	0	0.258976674	-6.761948676	0.000858
ENSG00000157343	ARMC12	0	0.410381231	-6.761948676	0.000858
ENSG00000232237	ASCL5	0	0.28722635	-6.761948676	0.000858

ENSG00000229140	CCDC26	0	0.01922751	-6.761948676	0.000858
ENSG00000170165	CR848007.1	0	0.342507702	-6.761948676	0.000858
ENSG00000131386	GALNT15	0	0.096777081	-6.761948676	0.000858
ENSG00000243694	LINC02027	0	0.976376821	-6.761948676	0.000858
ENSG00000188095	MESP2	0	0.326371613	-6.761948676	0.000858
ENSG00000266634	MIR3972	0	6.688742362	-6.761948676	0.000858
ENSG00000223750	SIRPB3P	0	0.478553113	-6.761948676	0.000858
ENSG00000250366	TUNAR	0	0.167314717	-6.761948676	0.000858
ENSG00000107165	TYRP1	0	0.155552148	-6.761948676	0.000858
ENSG00000261889	AC108134.2	0	0.837812443	-6.867914063	0.000517
ENSG00000159625	DRC7	0	0.121662533	-6.867914063	0.000517
ENSG00000162981	FAM84A	0	0.07474758	-6.867914063	0.000517
ENSG00000229951	FLJ31356	0	0.208824961	-6.867914063	0.000517
ENSG00000112964	GHR	0	0.094808428	-6.867914063	0.000517
ENSG00000253819	LINC01151	0	1.097519628	-6.867914063	0.000517
ENSG00000285373	LINC02478	0	1.020657504	-6.867914063	0.000517
ENSG00000202111	VTRNA1-2	0	7.041389971	-6.867914063	0.000517
ENSG00000176678	FOXL1	0.033499827	4.186823273	-6.900713064	4.48E-23
ENSG00000272944	AC079834.2	0	0.344508378	-6.966626055	0.000315
ENSG00000255202	AL049629.1	0	1.036183379	-6.966626055	0.000315
ENSG00000237945	LINC00649	0	0.046836414	-6.966626055	0.000315
ENSG00000105088	OLFM2	0	0.268578732	-6.966626055	0.000315
ENSG00000223784	LINP1	0.020294051	2.771878606	-6.999544286	5.86E-21
ENSG00000285755	AC132153.1	0	0.362271093	-7.059014353	0.000194
ENSG00000273027	AL844908.2	0	1.556978155	-7.059014353	0.000194
ENSG00000269028	MTRNR2L12	0	0.682754958	-7.059014353	0.000194
ENSG00000112530	PACRG	0	0.274199828	-7.059014353	0.000194
ENSG00000151812	SLC35F4	0	0.168917441	-7.059014353	0.000194
ENSG00000172985	SH3RF3	0.007200664	1.103071935	-7.080665958	9.41E-17
ENSG00000253858	AC008619.1	0	1.342104186	-7.145840624	0.00012
ENSG00000257346	AC011603.1	0	0.907000087	-7.145840624	0.00012
ENSG00000274307	AC023449.2	0	0.317072114	-7.145840624	0.00012
ENSG00000267737	AC087645.2	0	0.664605304	-7.145840624	0.00012
ENSG00000285108	AC103718.1	0	0.070933359	-7.145840624	0.00012
ENSG00000273443	AL645608.8	0	0.917940981	-7.145840624	0.00012
ENSG00000231701	BMS1P13	0	0.85985658	-7.145840624	0.00012
ENSG00000171450	CDK5R2	0	0.303418291	-7.145840624	0.00012
ENSG00000243710	CFAP57	0	0.132021699	-7.145840624	0.00012
ENSG00000175229	GAL3ST3	0	0.306720304	-7.145840624	0.00012
ENSG00000276404	MIR6835	0	11.89020427	-7.145840624	0.00012
ENSG00000187642	PERM1	0	0.222246809	-7.145840624	0.00012
ENSG00000118094	TREH	0	0.2391493	-7.145840624	0.00012
ENSG00000261220	AC103706.1	0	1.186651245	-7.227736708	7.55E-05

ENSG00000243885	AC108751.4	0	0.804931264	-7.227736708	7.55E-05
ENSG00000225472	AL136366.1	0	0.764455593	-7.227736708	7.55E-05
ENSG00000230021	AL669831.3	0	0.146630791	-7.227736708	7.55E-05
ENSG00000258474	LINC02313	0	1.464974901	-7.227736708	7.55E-05
ENSG00000065618	COL17A1	0	0.120365032	-7.305232542	3.05E-05
ENSG00000124915	DKFZP434K028	0	0.294392287	-7.305232542	3.05E-05
ENSG00000245888	FLJ21408	0	0.260410079	-7.305232542	3.05E-05
ENSG00000167083	GNGT2	0	0.503551994	-7.305232542	3.05E-05
ENSG00000091128	LAMB4	0	0.121222822	-7.305232542	3.05E-05
ENSG00000197251	LINC00336	0	0.403654161	-7.305232542	3.05E-05
ENSG00000168631	MUCL3	0	0.14968309	-7.305232542	3.05E-05
ENSG00000169297	NR0B1	0	0.386064148	-7.305232542	3.05E-05
ENSG00000279390	AF127577.6	0	0.408609055	-7.378776962	1.96E-05
ENSG00000259030	FPGT-TNNI3K	0	0.090467102	-7.378776962	1.96E-05
ENSG00000204897	KRT25	0	0.536085293	-7.378776962	1.96E-05
ENSG00000089012	SIRPG	0	0.399314201	-7.378776962	1.96E-05
ENSG00000162520	SYNC	0	0.256742885	-7.378776962	1.96E-05
ENSG00000253771	TPTE2P1	0	0.236217002	-7.378776962	1.96E-05
ENSG00000160886	LY6K	0.020639887	3.692487709	-7.388733428	1.50E-23
ENSG00000263096	AC007638.2	0	0.540244575	-7.448753447	1.27E-05
ENSG00000197550	AL359955.1	0	0.287030706	-7.448753447	1.27E-05
ENSG00000107611	CUBN	0	0.070838399	-7.448753447	1.27E-05
ENSG00000186529	CYP4F3	0	0.170387087	-7.448753447	1.27E-05
ENSG00000102359	SRPX2	0	0.124539687	-7.448753447	1.27E-05
ENSG00000172137	CALB2	0.024536377	5.07297859	-7.512935906	1.98E-19
ENSG00000236098	AC097059.2	0	1.663494397	-7.515492229	8.28E-06
ENSG00000198074	AKR1B10	0	0.56467241	-7.515492229	8.28E-06
ENSG00000231995	AL590399.4	0	1.638583499	-7.515492229	8.28E-06
ENSG00000248409	AP000344.2	0	0.81929175	-7.515492229	8.28E-06
ENSG00000245857	GS1-24F4.2	0	0.687221691	-7.515492229	8.28E-06
ENSG00000254363	AC011379.2	0	0.167679447	-7.579279713	5.45E-06
ENSG00000259347	AC087482.1	0	0.291245207	-7.579279713	5.45E-06
ENSG00000140945	CDH13	0	0.09203109	-7.579279713	5.45E-06
ENSG00000143226	FCGR2A	0	0.193743283	-7.579279713	5.45E-06
ENSG00000223764	LINC02593	0	0.247965271	-7.579279713	5.45E-06
ENSG00000225383	SFTA1P	0	1.249456074	-7.579279713	5.45E-06
ENSG00000257178	AC103702.1	0	0.408484763	-7.640365902	3.61E-06
ENSG00000168070	MAJIN	0	0.466890451	-7.640365902	3.61E-06
ENSG00000189068	VSTM1	0	0.764636959	-7.640365902	3.61E-06
ENSG00000260834	AC009055.2	0	0.764397574	-7.698970325	2.40E-06
ENSG00000271590	AC108463.3	0	0.785317929	-7.755286786	1.61E-06
ENSG00000273102	AP000569.1	0	1.969274401	-7.755286786	1.61E-06
ENSG00000196188	CTSE	0	0.435732374	-7.755286786	1.61E-06

ENSG00000229028	KRT223P	0	0.708858823	-7.809487241	1.09E-06
ENSG00000149256	TENM4	0	0.074011286	-7.809487241	1.09E-06
ENSG00000257126	FOXG1-AS1	0	1.434058827	-7.861724961	7.40E-07
ENSG00000231249	ITPR1-DT	0	1.460801183	-7.861724961	7.40E-07
ENSG00000169554	ZEB2	0	0.050018653	-7.861724961	7.40E-07
ENSG00000231013	AC013275.1	0	0.621412416	-7.912137143	5.06E-07
ENSG00000284648	AC097493.4	0	5.927536698	-7.912137143	5.06E-07
ENSG00000197614	MFAP5	0	0.358012585	-8.007965993	2.40E-07
ENSG00000141293	SKAP1	0	0.52403957	-8.007965993	2.40E-07
ENSG00000283196	AC006453.2	0	0.593380241	-8.053594531	1.67E-07
ENSG00000257849	AC012038.2	0	5.77024619	-8.097824095	1.17E-07
ENSG00000159387	IRX6	0	0.434593417	-8.097824095	1.17E-07
ENSG00000242759	LINC00882	0	0.380521129	-8.097824095	1.17E-07
ENSG00000187783	TMEM72	0	0.467907198	-8.097824095	1.17E-07
ENSG00000137968	SLC44A5	0	0.241617105	-8.140737924	8.20E-08
ENSG00000149654	CDH22	0	0.3178554	-8.182412043	5.79E-08
ENSG00000227764	LINC01693	0	1.301253545	-8.182412043	5.79E-08
ENSG00000258947	TUBB3	0	0.363763519	-8.222916071	4.11E-08
ENSG00000149596	JPH2	0	0.293750927	-8.338021803	1.51E-08
ENSG00000092096	SLC22A17	0	0.408078952	-8.338021803	1.51E-08
ENSG00000012124	CD22	0.006784454	2.492896709	-8.342148618	7.08E-25
ENSG00000105695	MAG	0	0.605520757	-8.374436214	1.09E-08
ENSG00000247809	NR2F2-AS1	0	0.302702952	-8.409954089	7.89E-09
ENSG00000162722	TRIM58	0	0.610721664	-8.511544476	3.09E-09
ENSG00000167644	C19orf33	0	3.264733368	-8.543878103	2.27E-09
ENSG00000212479	RF00012	0	9.785426661	-8.606449358	1.25E-09
ENSG00000258586	LINC02274	0	2.020822371	-8.779361865	1.70E-10
ENSG00000233559	LINC00513	0	1.551197296	-8.858620425	7.60E-11
ENSG00000230699	AL645608.2	0	0.853191283	-8.909139326	4.50E-11
ENSG00000230148	HOXB-AS1	0	1.751475587	-8.981748074	2.09E-11
ENSG00000154319	FAM167A	0	0.582026408	-9.095188655	6.14E-12
ENSG00000080031	PTPRH	0	0.690407268	-9.116844355	4.84E-12
ENSG00000267365	KCNJ2-AS1	0	1.246475141	-9.138179791	3.82E-12
ENSG00000139915	MDGA2	0	0.536949612	-9.589187316	1.63E-14
ENSG00000107242	PIP5K1B	0	0.86468007	-9.619844681	1.12E-14
ENSG00000157005	SST	0	14.70374395	-10.71434913	3.34E-21

Table S7: MeRIP-seq shows the changes of m6A peak in PEG10 mRNA after ALKBH5 knockdown

MeRIP-seq	Value
Overlap_Symbol	PEG10
Overlap_Gene	ENSG00000242265
Chromosome	7
Peak Start	94668005
Peak End	94668181
Summit Positon	94668085
RPM_ALKBH5_shRNA_NC_input	2.24046
RPM_ALKBH5_shRNA_NC_IP	2.91276
RPM_ALKBH5_shRNA_input	8.2782
RPM_ALKBH5_shRNA_IP	15.709
Fold Enrich shRNA NC	1.300072307
Fold Enrich shRNA	1.897634752
fold change(Log2)	-0.545610471
Overlap Begin	UTR3
Overlap Center	UTR3
Overlap End	UTR3

Table S8: Data of Flag-IGF2BP1 CoIP-MS.

Accession	Protein ID	Score
Q9NZI8	IGF2BP1	266227343.7
P68133	ACTA1	211029463.4
P08670	VIM	187443931.4
Q5EBM2	-	142157104.2
Q96A32	MYLPF	132470701.8
P02533	KRT14	119135780.8
A0A0S2Z4G6	TPM1	92864850.08
P13647	KRT5	79768120.41
P05976	MYL1	77303131.61
B2R892	-	70799899.93
P11940	PABPC1	59442062.85
S6B294	-	58672703.4
J3KPS3	ALDOA	57793514
P13929	ENO3	50718530.12
Q53G85	-	37755230.82
Q549M8	C14orf166	29071237.72
B4DN72	-	26939263.03
P04406	GAPDH	25613319.8

B2R853	-	24152144.44
Q04695	KRT17	22415461.59
P16402	HIST1H1D	21305724.07
F5H5D3	TUBA1C	20493315.56
A0A024R9D2	MTDH	20062772.39
P12882	MYH1	19989743.88
C9JZN9	TNNT3	19774522.96
Q15323	KRT31	18556285.34
V9HW22	HEL-S-72p	18474943.52
P04259	KRT6B	18437271.91
A0A384N5Z8	-	18049642.17
A0A024R5Z9	PKM2	17174833.47
Q09161	NCBP1	16894500.9
B4DPP6	-	16742896.2
P60709	ACTB	16271137.88
P42166	TMPO	15687785.8
P07951	TPM2	15385285.92
J3KTL2	SRSF1	14298171.06
Q59F66	-	14064518.31
P68371	TUBB4B	12912778.92
A8K6N9	-	12856563.55
P67809	YBX1	12437537.6
A0A0S2Z4Z0	RBM14	11568074.17
P00338	LDHA	11508668
A0A075B6S5	IGKV1-27	11321483.7
P22626	HNRNPA2B1	10907405.88
Q6IQ30	PABPC4	10644704.99
P26368	U2AF2	10140088.4
A0A3B3ITK7	PGM1	9923664.141
A0A0S2Z4Z9	NONO	9693852.434
Q9Y2W1	THRAP3	9638929.275
A0A024RD80	HSP90AB1	9470380.404
P60174	TPI1	9153859.864
A0A0M4FEM1	PYGM	8744292.26
B2RDW1	RPS27A	8378415.171
P15924	DSP	8318307.582
V9HWB4	HEL-S-89n	8229882.382
P27694	RPA1	8217233.703
Q5T9B7	AK1	8187904.127
P19474	TRIM21	7928308.633
D3GKD9	HBG2	7756099.743
P81605	DCD	7658490.769
P04792	HSPB1	7205952.241
A0A024RDE5	G3BP2	7041190.337

B2R491	RPS4X	6970370.529
B2R8Y4	-	6963600.217
Q8WWM7	ATXN2L	6894786.825
B4DUQ1	-	6693627.74
J3QQ67	RPL18	6561300.219
A6XMV9	PRSS2	6442642.161
Q13344	-	6404849.481
P02585	TNNC2	6383729.849
P23396	RPS3	5950562.764
P26373	RPL13	5945875.001
P15259	PGAM2	5869078.251
Q12906	ILF3	5403550.144
A0A0F7KYT8	FXR1	5384325.722
Q9HBB3	-	5191525.952
B7Z2B0	-	5073516.183
B2R6F3	SFRS3	4839077.007
F8W930	IGF2BP2	4753011.841
Q8WVJ2	NUDCD2	4735683.197
A8MUS3	RPL23A	4728429.057
A0A087X0X3	HNRNPM	4700735.256
A0A2U3TZU2	GPI	4681294.858
A0A024R6W2	NUDT21	4495515.997
Q02413	DSG1	4273103.208
Q9UKX2	MYH2	4111078.03
P07437	TUBB	4110505.245
V9HW31	HEL-S-271	3962163.448
P12956	XRCC6	3875685.349
Q9HCE1	MOV10	3873705.3
Q8IVF2	AHNAK2	3823075.892
V9HWF4	HEL-S-68p	3822906.267
Q9NYF8	BCLAF1	3766240.203
Q15717	ELAVL1	3717293.412
A0A024R825	CA3	3658595.81
A8MXP9	MATR3	3655031.113
Q6PKG0	LARP1	3625335.919
Q9Y3I0	RTCB	3586710.481
Q7Z417	NUFIP2	3577377.406
A0A0U1RRH7	-	3516156.356
H0Y8C2	RPL22L1	3419346.965
P50914	RPL14	3408642.326
C8C504	HBB	3329009.1
Q5JR94	RPS8	3292656.077
Q16629	SRSF7	3275236.01
Q8N1C8	HSPA9	3256014.028

A0A1K0GXZ1	GLNC1	3210026.097
P62917	RPL8	3205721.185
P62891	RPL39	3193124.215
P46776	RPL27A	3180937.286
P51991	HNRNPA3	3161771.351
B2R4P2	-	3076058.862
A3RJH1	DDX1	3061985.322
F8W8D3	SLBP	3010291.412
Q8N1N4	KRT78	2973646.6
P62263	RPS14	2967480.481
Q6NVC0	SLC25A5	2962614.163
A0A024RAZ7	HNRPA1	2925236.844
P62424	RPL7A	2918015.084
O00425	IGF2BP3	2912089.876
A8K4W0	RPS3A	2860216.736
P62280	RPS11	2797227.404
B2R4R0	HIST1H4L	2795149.322
A2A3R6	RPS6	2784259.796
A0A024R4A0	NCL	2723530.695
Q14444	CAPRIN1	2719497.083
A0A0D9SF53	DDX3X	2653067.355
Q5EB52	MEST	2621100.255
P41219	PRPH	2620886.881
A0A024R4M0	RPS9	2605327.184
P15880	RPS2	2516321.427
A0A2R8Y5Y7	RPL9	2440208.032
P50454	SERPINH1	2413250.212
A0A024R7L8	UPF1	2412372.533
A0A3B3ITT5	RPL29	2391324.182
A0A024QZE0	TDRD3	2375337.433
P39023	RPL3	2360007.288
G8JLB6	HNRNPH1	2349594.916
A0A0U1RRM4	PTBP1	2338757.383
Q59FI9	-	2314581.064
P07355	ANXA2	2269903.422
B4E3P1	-	2253388.716
A0A384N6B9	-	2245253.526
B2R7W4	-	2195282.568
Q17RB8	LONRF1	2145836.727
Q14525	KRT33B	2112265.36
P62913	RPL11	2077118.125
A0A0A6YYL6	RPL17-C18orf32	2062246.102
P84098	RPL19	2035692.714
Q00325	SLC25A3	2032788.564

A0A384MQX1	-	2027170.665
A8K588	-	1992115.717
A0A087X1N7	NEB	1980532.457
P78385	KRT83	1968077.288
Q08554	DSC1	1913876.805
M0QYS1	RPL13A	1908277.678
Q08211	DHX9	1890553.053
B5BUE6	DDX5	1843580.488
A0A024R1X8	JUP	1807076.761
Q00839	HNRNPU	1799665.894
Q12905	ILF2	1793075.336
A8K9U0	-	1789866.996
P38159	RBMX	1769000.497
Q01844	EWSR1	1766183.31
A0A384N6C7	-	1751123.754
Q9NUD5	ZCCHC3	1741898.9
E9PB61	ALYREF	1738034.06
P02511	CRYAB	1696019.809
A0A384NPX1	-	1652722.821
K7EL05	HKR1	1587345.903
A0A2R8Y6G6	ENO1	1584422.691
A8K7N0	-	1533225.133
A0A1U9X972	-	1523286.318
A0A024R2Q4	RPL15	1518192.303
Q5TEC6	HIST2H3PS2	1458480.152
Q59HH7	-	1452521.995
A8K6U7	-	1439395.26
B7Z3K9	-	1438590.296
Q5T749	KPRP	1436808.367
A8K4Z4	-	1436135.056
Q0IIN1	KRT77	1422864.944
Q9UNX3	RPL26L1	1404413.221
A8K3B0	-	1381232.032
Q8NCA5	FAM98A	1360074.571
A0A161I202	LTF	1357402.555
Q9NZB2	FAM120A	1338453.506
P25705	ATP5F1A	1326072.534
Q8N684	CPSF7	1303508.038
P62081	RPS7	1290775.282
P78386	KRT85	1256959.2
Q16630	CPSF6	1251646.388
K7ELG9	LSM12	1242140.512
P42766	RPL35	1222519.566
Q9NR12	PDLIM7	1199068.124

A0A024R8A7	hCG_31253	1166452.923
P35609	ACTN2	1148817.022
Q59GY2	-	1139038.078
Q5D862	FLG2	1136327.536
M0R0R2	RPS5	1128752.376
Q32P45	G3BP1	1107033.414
A8K9A4	-	1061526.651
P62906	RPL10A	1052986.21
Q96HU8	DIRAS2	1037919.863
K9JA46	EL52	1009338.145
B7Z4C8	RPL31	1009126.095
Q9Y5A9	YTHDF2	991193.0854
K7ELC7	RPL27	990617.0469
Q92522	H1FX	968838.9857
A0A087WXS0	TNNI2	946549.9415
A0A1U9X8X6	-	943594.6581
M1VPF4	TPM3-ROS1	904211.9847
Q01130	SRSF2	897102.8224
P60866	RPS20	891192.8107
A0A024R814	RPL7	888404.4412
A8K5C4	-	886716.6079
H0YKD8	RPL28	884847.3488
Q14324	MYBPC2	870329.4881
Q5JSZ5	PRRC2B	848568.384
Q5T9A4	ATAD3B	843802.6642
P00441	SOD1	837009.0861
Q9UBB4	ATXN10	826544.9665
Q86YZ3	HRNR	824249.0624
P62851	RPS25	802049.5771
A8K7H3	-	800527.9863
P49916	LIG3	780251.3667
B2R7B5	-	779616.1956
Q9Y4C2	TCAF1	767631.7698
Q86TD4	SRL	764950.2971
Q9BRZ2	TRIM56	748859.0723
B3KTJ9	-	746117.0275
Q5SSJ5	HP1BP3	744884.024
B2R8Z8	-	737468.6507
Q53F64	-	737360.8436
P35030	PRSS3	737221.7402
P23284	PPIB	718017.1236
P13010	XRCC5	712027.6962
P10599	TXN	707474.0529
Q86TJ2	TADA2B	688545.8099

Q6KB66	KRT80	675960.2916
P62277	RPS13	664672.8484
A0A024R3X4	HSPD1	659031.5024
A0A2R8Y891	PFKM	644191.6406
Q9Y520	PRRC2C	642582.0546
P05109	S100A8	638691.5155
Q96P63	SERPINB12	636398.7746
Q71RC2	LARP4	634124.5223
P63244	RACK1	627804.8307
B2R4C0	-	627467.0265
A0A087WUZ3	SPTBN1	623288.6553
Q01469	FABP5	620377.4244
P13535	MYH8	618266.006
A0A024R3T8	PARP1	611948.9999
P50402	EMD	603695.0035
A0A024RDB2	G3BP2	603506.1151
P13639	EEF2	601660.1217
V9HW01	HEL-S-310	595390.7186
G9K388	YWHAE/FAM22A	582553.6805
Q9BYJ9	YTHDF1	577019.5284
Q86TB9	PATL1	566924.774
O76009	KRT33A	561154.9633
A0A0X9SZU9	-	559017.4388
O94916	NFAT5	557489.8572
O75494	SRSF10	544371.4361
E5KSU5	TFAM	536902.34
Q549N0	CFL2	536882.9735
B2R6C0	-	533012.5562
A0A024R2Q5	MYL3	526888.6475
A0A024RDR0	HMGB1	518835.3143
E9PAV3	NACA	518270.5342
Q1KMD3	HNRNPUL2	516732.5205
A8K6H6	-	504749.2502
Q7Z4S6	KIF21A	491593.0902
Q59EJ3	-	490159.7075
H3BNC9	-	489679.3396
Q08188	TGM3	486682.4956
B2R7E8	-	481601.9145
P52597	HNRNPF	470903.8857
Q9BXP5	SRRT	466772.7172
A0A140VK53	-	455027.4101
A8KAN5	-	454429.4615
A0A024QZ86	TBX2	451761.3195

Q9Y3U8	RPL36	449875.9474
Q9BX40	LSM14B	431693.5746
Q9Y623	MYH4	420080.3344
V9HVY3	HEL-S-269	414816.9718
Q13895	BYSL	408596.7985
Q9NQ39	RPS10P5	406289.4681
Q9BUF5	TUBB6	403951.2545
Q969X5	ERGIC1	391915.1781
Q7Z6E5	DKFZp779G2251	391823.2516
A8K486	-	364293.8344
B4DR52	-	360273.9203
P29508	SERPINB3	354106.3594
A0A024R7X7	STAU2	344728.2899
A0A140VK00	-	343421.2675
R4GMX3	COMMD3-BMI1	338954.4122
A0A0U1RQQ9	SCYL2	338531.6276
A0A024RAD5	DDOST	330651.9994
Q96HS1	PGAM5	326538.1834
P61626	LYZ	326012.3945
A0A024R4K3	MDH2	314923.3164
P01040	CSTA	308206.2492
B2CIS9	CASP14	307927.7066
Q9NX05	FAM120C	301319.4212
A8KAF9	RP11-311P8.3	299002.0783
P20930	FLG	290050.8604
Q9C0J8	WDR33	278998.4496
Q15365	PCBP1	274258.2678
P51116	FXR2	273548.174
P26641	EEF1G	273303.0322
Q9P0L0	VAPA	271234.7109
Q13151	HNRNPA0	265868.3182
Q9UKV3	ACIN1	252498.7905
P07305	H1F0	246412.5046
P42677	RPS27	240090.9022
P62829	RPL23	236837.6064
Q9BYE4	SPRR2G	236675.4438
A0A3F2YNY6	PRPF40A	236047.7353
P62269	RPS18	235165.7828
O14893	GEMIN2	226861.0976
V9GYM8	ARHGEF2	224731.5263
Q86US8	SMG6	219533.5387
B4DWA6	-	217393.5417
G3V1L9	TJP1	215577.8376
Q96GA3	LTV1	207389.2485

Q13242	SRSF9	204784.0412
P04843	RPN1	192853.6868
Q59H77	-	189398.6561
A5YM43	PHC3	188805.1102
D9HTE9	SLC25A1	184631.3999
Q562T9	ACT	182777.1821
Q9UHX1	PUF60	182776.9239
Q9UPQ9	TNRC6B	180560.7066
Q9HA47	UCK1	179319.4272
A0A024RDF3	HNRPD	177309.6186
Q9Y3Z3	SAMHD1	175975.3281
Q9NP98	MYOZ1	167722.2484
P38919	EIF4A3	167078.4785
Q6UN15	FIP1L1	167043.8899
D3DTX6	PPP1R9B	166853.3618
Q59F99	-	166237.6734
E5KNY5	LRPPRC	165192.1123
B2R8R5	-	158119.2945
Q8WVX7	-	156200.5603
A0A024R4E2	TARDBP	155239.8892
A0A0A0MTI1	DHCR24	149577.0722
Q00688	FKBP3	141014.4376
A0A0J9YX62	DNAJB6	137946.712
Q00577	PURA	134776.3696
Q8TB72	PUM2	134265.351
A8K9U6	-	132186.3522
A0A024R572	SF1	129026.4935
Q13595	TRA2A	119607.9866
B5BUB5	SSB	115079.1197
Q53GL6	RALY	109504.33
F6VRR5	POLDIP3	107162.0301
B5BU08	U2AF1	102486.8457
P35580	MYH10	99851.772
Q96QD9	FYTTD1	98168.36074
B2RNR6	ZFR	97754.83332
O75533	SF3B1	97278.16451
Q96QR8	PURB	94598.40897
Q14011	CIRBP	92811.08328
B2RDP6	-	88403.36698
Q5W009	RBM17	83845.8698
Q5VW26	NFIB	82321.67092
Q06787	FMR1	81402.88321
B3KMV8	-	80633.27601
D9IAI1	HEL-S-34	80527.40555

Q96T37	RBM15	77591.88249
A8K4Z6	-	69558.68407
B0AZQ4	-	58375.64593
Q9P2N5	RBM27	56815.15414
P35998	PSMC2	54813.48597
Q53F19	NCBP3	50890.36262
A8K4B4	-	44204.53683
B2RBA6	MCM7	37622.7223
A0A024R1C2	TOP3B	35669.61896
Q8IUH3	RBM45	14515.95952
Q99700	ATXN2	13829.56875
Q6PJV4	THRAP3	2494.25004

Table S9: CUT&Tag-seq detection of DNA sequences binding to PEG10 protein (cell cycle regulation related genes only)

Chr	Start	End	Summit	Annotation	Fold enrichment	X.log10.qvalue.	SYMBOL
chr1	1721406	1721683	1721430	Promoter (<=1kb)	8.79979	38.51482	CDK11A
chr17	39461727	39461961	39461902	Promoter (<=1kb)	7.66519	24.49354	CDK12
chr7	39949429	39950651	39949741	Promoter (<=1kb)	7.80525	45.04142	CDK13
chr7	90824279	90824500	90824312	Promoter (<=1kb)	5.05992	14.79867	CDK14
chrX	47217901	47218779	47218401	Promoter (<=1kb)	7.36808	66.61408	CDK16
chr12	96400036	96400451	96400320	Promoter (<=1kb)	9.87958	37.26735	CDK17
chr1	205528697	205529090	205528918	Promoter (<=1kb)	5.6819	20.32988	CDK18
chr6	110815423	110816203	110815693	Promoter (<=1kb)	14.51497	138.57053	CDK19
chr12	55967861	55968128	55967924	Promoter (<=1kb)	4.44028	10.51722	CDK2
chr9	87973894	87974812	87974220	Promoter (<=1kb)	7.56146	35.6978	CDK20
chr12	123267294	123267607	123267436	Promoter (<=1kb)	4.70187	19.94539	CDK2AP1
chr11	67507798	67509150	67508440	Promoter (<=1kb)	7.8708	108.60513	CDK2AP2
chr17	76000610	76000875	76000835	Promoter (<=1kb)	5.00609	21.9453	CDK3
chr12	57751200	57752785	57752542	Promoter (<=1kb)	7.28541	34.6412	CDK4
chr7	151054878	151055366	151054960	Promoter (<=1kb)	3.85937	14.90605	CDK5
chr17	32486259	32486810	32486545	Promoter (<=1kb)	6.7271	21.52808	CDK5R1
chr20	33402016	33402414	33402066	Promoter (<=1kb)	4.76945	10.44488	CDK5RAP1
chr9	120448618	120448976	120448741	Promoter (<=1kb)	12.77532	56.04292	CDK5RAP2
chr17	47982055	47982595	47982323	Promoter (<=1kb)	10.6041	46.48113	CDK5RAP3
chr7	92834965	92835188	92834990	Promoter (<=1kb)	5.95184	25.94629	CDK6
chr5	69234513	69234943	69234903	Promoter (<=1kb)	5.06541	12.80028	CDK7
chr9	127788974	127789351	127789059	Promoter (<=1kb)	5.92928	25.45385	CDK9
chr14	50340878	50341115	50341054	Promoter (<=1kb)	5.60566	14.54599	CDKL1
chr4	75631022	75631259	75631065	Promoter (<=1kb)	3.23642	4.68413	CDKL2
chr5	134370827	134371418	134371212	Promoter (<=1kb)	11.05854	99.51375	CDKL3
chrX	18441163	18441388	18441317	Promoter (<=1kb)	7.75811	26.67406	CDKL5
chr6	36679194	36679742	36679503	Promoter (<=1kb)	7.02786	35.44812	CDKN1A
chr12	12717869	12718163	12718077	Promoter (<=1kb)	9.00577	39.73072	CDKN1B
chr11	2885247	2885857	2885818	Promoter (<=1kb)	4.46234	18.53504	CDKN1C
chr9	21995875	21996517	21996284	Promoter (<=1kb)	9.41684	42.18028	CDKN2A
chr4	183444473	183445673	183444730	Promoter (<=1kb)	11.76198	53.11134	CDKN2AIP
chr5	134411664	134412365	134412018	Promoter (<=1kb)	6.10236	33.81908	CDKN2AIPNL
chr9	21994690	21995365	21995091	Promoter (<=1kb)	6.02115	21.8486	CDKN2B-AS1
chr1	50968225	50968671	50968299	Promoter (<=1kb)	9.19823	33.17646	CDKN2C
chr19	10568614	10569361	10568971	Promoter (<=1kb)	7.406	54.2689	CDKN2D
chr14	54396793	54397728	54396978	Promoter (<=1kb)	9.54305	52.89744	CDKN3
chr13	36431480	36432080	36431823	Promoter (<=1kb)	11.07195	44.73887	CCNA1
chr4	121823487	121824035	121823844	Promoter (<=1kb)	15.50072	75.45017	CCNA2
chr5	69173211	69173390	69173277	Promoter (<=1kb)	9.60764	40.50388	CCNB1
chr14	20332932	20333420	20333033	Promoter (<=1kb)	6.47283	18.29481	CCNB1IP1
chr15	59105053	59105661	59105495	Promoter (<=1kb)	9.96409	41.5736	CCNB2
chrX	50204112	50204411	50204129	Promoter (<=1kb)	5.11013	11.90867	CCNB3
chr6	99568260	99568946	99568629	Promoter (<=1kb)	9.12877	34.09392	CCNC

chr11	69644362	69644753	69644508	Promoter (<=1kb)	8.9011	62.99952	CCND1
chr6	42050270	42050806	42050738	Promoter (<=1kb)	10.58614	74.83663	CCND3
chr15	43185934	43186263	43185933	Promoter (<=1kb)	6.30249	17.45304	CCNDBP1
chr19	29811677	29812277	29812196	Promoter (<=1kb)	8.43252	29.5218	CCNE1
chr8	94894930	94895832	94895225	Promoter (<=1kb)	9.30527	39.02406	CCNE2
chr16	2428358	2429490	2428761	Promoter (<=1kb)	6.72375	37.68261	CCNF
chr5	163437322	163437815	163437612	Promoter (<=1kb)	9.22506	39.8853	CCNG1
chr4	77157556	77158311	77157641	Promoter (<=1kb)	7.34584	29.23553	CCNG2
chr5	87412225	87413238	87412899	Promoter (<=1kb)	10.56093	41.48962	CCNH
chr4	77076007	77076313	77076194	Promoter (<=1kb)	8.51688	29.22655	CCNI
chr10	96043377	96043975	96043599	Promoter (<=1kb)	13.95327	96.42113	CCNJ
chr3	157160558	157160814	157160650	Promoter (<=1kb)	6.96434	21.28909	CCNL1
chr1	1399007	1399823	1399732	Promoter (<=1kb)	6.88977	39.36238	CCNL2
chr12	48716657	48717228	48716784	Promoter (<=1kb)	13.116	58.38448	CCNT1
chr10	35336393	35336726	35336689	Promoter (<=1kb)	8.14606	27.24198	CCNY
chr2	207711835	207712612	207712085	Promoter (<=1kb)	9.06282	45.97808	CCNYL1
